# Suramin exposure alters cellular metabolism and mitochondrial energy production in African trypanosomes

**DOI:** 10.1074/jbc.RA120.012355

**Published:** 2020-04-30

**Authors:** Martin Zoltner, Gustavo D. Campagnaro, Gergana Taleva, Alana Burrell, Michela Cerone, Ka-Fai Leung, Fiona Achcar, David Horn, Sue Vaughan, Catarina Gadelha, Alena Zíková, Michael P. Barrett, Harry P. de Koning, Mark C. Field

**Affiliations:** 1 School of Life Sciences, University of Dundee, Dundee, Scotland, United Kingdom; 2 Institute for Infection, Immunity, and Inflammation, College of Medical, Veterinary and Life Sciences, University of Glasgow, Glasgow, United Kingdom; 3 Institute of Parasitology, Biology Centre, Czech Academy of Sciences, Institute of Parasitology, Faculty of Science, University of South Bohemia, České Budějovice, Czech Republic; 4 Department of Biological and Medical Sciences, Oxford Brookes University, Oxford, United Kingdom; 5 Department of Pathology, University of Cambridge, Cambridge, United Kingdom; 6 Wellcome Centre for Integrative Parasitology and Glasgow Polyomics, University of Glasgow, Glasgow, United Kingdom; 7 School of Life Sciences, University of Nottingham, Nottingham, United Kingdom

**Keywords:** drug action, trypanosome, Trypanosoma brucei, parasite metabolism, metabolomics, suramin, glycosomes, polypharmacology, proteomics, differentiation, drug mechanisms, energy homeostasis, sleeping sickness

## Abstract

Introduced about a century ago, suramin remains a frontline drug for the management of early-stage East African trypanosomiasis (sleeping sickness). Cellular entry into the causative agent, the protozoan parasite *Trypanosoma brucei*, occurs through receptor-mediated endocytosis involving the parasite's invariant surface glycoprotein 75 (ISG75), followed by transport into the cytosol via a lysosomal transporter. The molecular basis of the trypanocidal activity of suramin remains unclear, but some evidence suggests broad, but specific, impacts on trypanosome metabolism (*i.e.* polypharmacology). Here we observed that suramin is rapidly accumulated in trypanosome cells proportionally to ISG75 abundance. Although we found little evidence that suramin disrupts glycolytic or glycosomal pathways, we noted increased mitochondrial ATP production, but a net decrease in cellular ATP levels. Metabolomics highlighted additional impacts on mitochondrial metabolism, including partial Krebs' cycle activation and significant accumulation of pyruvate, corroborated by increased expression of mitochondrial enzymes and transporters. Significantly, the vast majority of suramin-induced proteins were normally more abundant in the insect forms compared with the blood stage of the parasite, including several proteins associated with differentiation. We conclude that suramin has multiple and complex effects on trypanosomes, but unexpectedly partially activates mitochondrial ATP-generating activity. We propose that despite apparent compensatory mechanisms in drug-challenged cells, the suramin-induced collapse of cellular ATP ultimately leads to trypanosome cell death.

## Introduction

*Trypanosoma brucei* is the causative agent of human and animal African trypanosomiasis (HAT and AAT, respectively) and has exerted significant impact on African economics, ecosystems, and public health for centuries. Whereas there are currently five drugs for HAT, their applicability depends on disease stage and causative subspecies. Adverse toxicity, complex administration, and emerging resistance all demand new treatments (1–3). Concurrent is the agricultural impact of *T. brucei* and the related species *Trypanosoma congolense*, *Trypanosoma equiperdum*, *Trypanosoma evansi*, and *Trypanosoma vivax*, which are major threats to livestock across sub-Saharan Africa, much of Asia, and parts of South America (4).

The drug development pipeline for HAT is currently better stocked than it has been for decades, with two new compounds, fexinidazole and acoziborole (a benzoxaborole), in approval and late stage clinical development, respectively (www.dndi.org/diseases-projects/portfolio/scyx-7158/; Accessed date: March 12, 2019) (6). A second benzoxaborole is under development for AAT (7). These drugs have both well-defined modes of action and, for the benzoxaboroles, defined structure-specific activation pathways (8) together with a target in central mRNA metabolism (9–11). Identification of the drug target, together with cellular entry pathways and metabolic interconversions, is of high value for exploitation of additional chemical space, for medicinal chemistry and for assessment of resistance mechanisms.

Of current trypanocidal drugs, suramin is by far the oldest and also presents the most complex genetic interaction with trypanosomes (12). Suramin is a polysulfonated trypan blue derivative and the negative charge prevents passive diffusion across membranes and access to the central nervous system, negating utility for late stage disease. The action of other drugs is comparatively simple, with mechanisms of cell entry, activation, and likely target at least partly characterized (12, 13). *T. brucei* suramin resistance has been challenging to obtain both in the laboratory or the field beyond a few interesting examples (14–16), including one dependent on expression of a specific variant surface glycoprotein, VSG^Sur^ (17, 18).

Suramin has high affinity for many proteins, including serum albumin and low-density lipoprotein (LDL), and *in vitro* EC_50_ varies depending on the composition and concentration of serum in the culture medium. The influence of LDL on suramin uptake and accumulation suggested an LDL receptor–mediated pathway for suramin internalization (19), but demonstration that altering abundance of LDL-binding sites in parasites does not impact the EC_50_ suggested that this was unlikely (20). Subsequently, the invariant surface glycoprotein ISG75 was identified as a major surface molecule involved in suramin sensitivity, together with the lysosomal MFST for cytosolic delivery (12, 21). Together, these data support a model for suramin entry mediated by endocytosis and delivery to the lysosome and explain the selective sensitivity of trypanosomes.

In many organisms, suramin has complex effects, including interactions with phosphatases (22), the cystic fibrosis chloride channel (23), and signaling pathway components (24), as well as acting as an immunosuppressant and chromatin modulator through sirtuins (25). Suramin inhibits Zika virus replication (26, 27) and has a beneficial impact on autism spectrum disorder (28). However, whereas many of these examples likely represent charge-mediated and nonspecific interactions between suramin and protein, there are important and specific interactions with biological systems (24, 29). The potential for suramin repurposing into these pharmacological spaces has been dampened by possible polypharmacology, toxicity, and an absence of a clear understanding of biochemical impact in any system.

Similarly, the mechanism of suramin trypanocidal activity remains unresolved. Whereas suramin inhibits the activity of cytosolic pyruvate kinase (cPYK) and all seven glycolytic enzymes compartmentalized in glycosomes (30) with IC_50_ values of 3–100 μm (31, 32), suramin inhibits trypanosome replication at ∼35 nm (12), indicating that, in the absence of a mechanism for significant concentration, glycolytic enzymes are unlikely to be the primary target.

Here, we analyzed the interactions of suramin with bloodstream form (BSF) *T. brucei* using metabolomics, genetics, and proteomics. We observed little impact on glycosome morphology or composition but found that suramin induces highly specific changes to metabolism. Specifically, decreased cellular ATP levels are accompanied by partial activation of the Krebs' cycle and increased expression of many proteins normally repressed in the bloodstream form.

## Results

### Suramin rapidly accumulates in cells proportional to ISG75 abundance

Previous work indicated that the abundant invariant surface glycoprotein ISG75 is involved in suramin sensitivity and that knockdown increased the EC_50_ ∼3-fold (12). Manipulation of ISG75 copy number via altering ubiquitylation efficiency also impacts suramin sensitivity (21). As a first step to understanding how suramin kills trypanosomes, we further validated the role of ISG75 and asked whether suramin accumulates within the cell.

**Figure 1.**
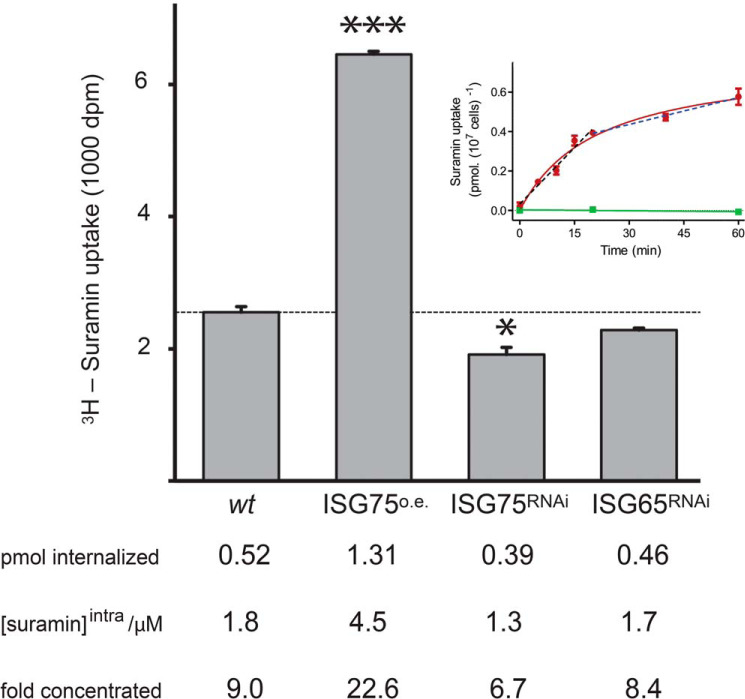
**Suramin is taken up rapidly and accumulates to high intracellular concentrations.** Shown are scintillation counts from 1 × 10^7^ cells incubated in 200 nm [^3^H]suramin over 15 min and surface binding cold-chased. *Bars*, mean of three independent experiments for WT cells (*wt*), ISG75-overexpressing cells (*ISG75O.E.*), and ISG75- and ISG65-silenced cells (*ISG75RNAi* and *ISG65RNAi*), respectively, with the S.E. (*error bars*) and significance intervals from Student's unpaired *t* test (*, *p* < 0.05; ***, *p* < 0.001) indicated. The intracellular suramin concentration was calculated using a volume of 29 μm^3^ for *T. brucei* BSF (33). *Inset*, suramin uptake in the presence of 250 nm suramin monitored over 1 h (*red line*). Suramin is taken up in two phases, a rapid initial phase over 20 min with 19.0 pmol/min (linear regression shown as a *black dashed line*; *r*^2^ = 0.97; *p* = 0.002, nonzero slope) and a slower phase (20–60 min) with 4.5 pmol/min (linear regression shown as a *dark blue dashed line*; *r*^2^ = 0.99; *p* = 0.048, nonzero slope). Saturability of uptake was determined by incubation of cells with 250 nm [^3^H]suramin in the presence of 100 μm unlabeled suramin (*p* = 0.48, zero slope) (*green line*).

Uptake of [^3^H]suramin was biphasic, with a rapid initial phase over 20 min and a longer, ∼4-fold slower, linear phase lasting at least 40 min (Fig. 1). Within 15 min, 0.5 pmol of suramin accumulated, whereas in cells overexpressing ISG75, 1.3 pmol was internalized (*p* < 0.001). Silencing reduced accumulation to 0.4 pmol (*p* < 0.05). ISG65 silencing had no significant effect on suramin uptake compared with WT cells (Fig. 1). Assuming a cell volume of ∼30 μm^3^ (33) (this study; see below) and uniform cellular distribution, this corresponds to intracellular suramin concentrations of 1.8 μm in WT, 4.5 μm in overexpressers, and 1.3 μm in silenced cells. As ISG75 expression levels in overexpressing or silenced cells were ∼2.5- and 0.2-fold (mRNA levels) of the parental cells, respectively (18, 34), there is a clear correlation between suramin accumulation and ISG75 level. This is consistent with a receptor role for ISG75 as suggested previously (35, 36), but rapid accumulation contrasts with the slow rate of parasite killing, with the latter requiring hours to days and suggestive of a protracted impact on cellular function, rather than a rapid and complete blockade to a specific and vital process.

### Suramin has no major impact on glycosome morphology

In trypanosomes, most enzymes of the glycolytic pathways are located within peroxisome-derived organelles or glycosomes (30). Suramin toxicity has been suggested to involve glycosomal activity, as it inhibits purified glycolytic enzymes (32), but penetration of the highly charged suramin molecule into the glycosome of live BSF trypanosomes has not been convincingly demonstrated, leaving doubt about an ability to disrupt trypanosomal glycolysis *in vivo*.

**Figure 2.**
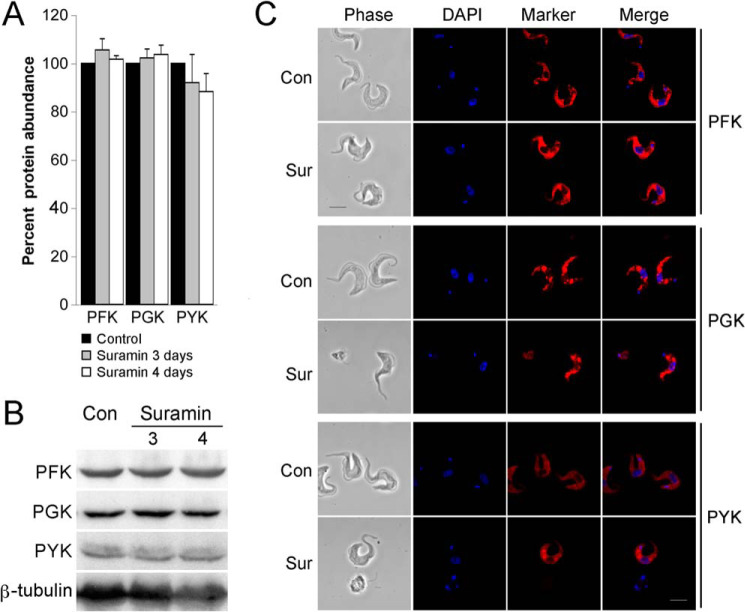
**Suramin has no significant impact on abundance or location of selected enzymes of the glycosomal pathway.** *A* and *B*, steady-state levels of PFK, PGK, and PYK were analyzed by immunoblotting at 0 (*CON*), 3, or 4 days post-suramin treatment (1× EC_50_). β-tubulin was used as loading control. Graphs represent the mean of three independent experiments, with the S.E. indicated. *C*, cells were treated with 1× EC_50_ suramin for 0 or 4 days, and the subcellular localizations of PFK, PGK, and PYK were detected using antibodies specific to each protein. Cells were stained with 4′,6-diamidino-2-phenylindole to visualize nuclear and mitochondrial DNA. *Scale bar*, 2 μm.

Suramin-treated cells were initially analyzed by immunofluorescence microscopy and immunoblotting. We selected three glycolytic pathway enzymes, two localized within the glycosome and one in the cytosol: phosphofructokinase (PFK; glycosome (g)) acting before the glycolytic branch point and the most suramin-sensitive of the glycolytic enzymes (32), phosphoglycerate kinase (PGK) acting after the branch point, and pyruvate kinase (PYK) producing pyruvate in the cytosol (c). Suramin treatment (1× EC_50_) did not significantly affect the abundance of these enzymes (Fig. 2, *A* and *B*). gPFK and gPGK remain localized to vesicular structures, typical of glycosome staining (Fig. 2*C*), and following 3–4 days of exposure to suramin, there was no significant alteration. Similarly, there were no changes to cPYK localization.

**Figure 3.**
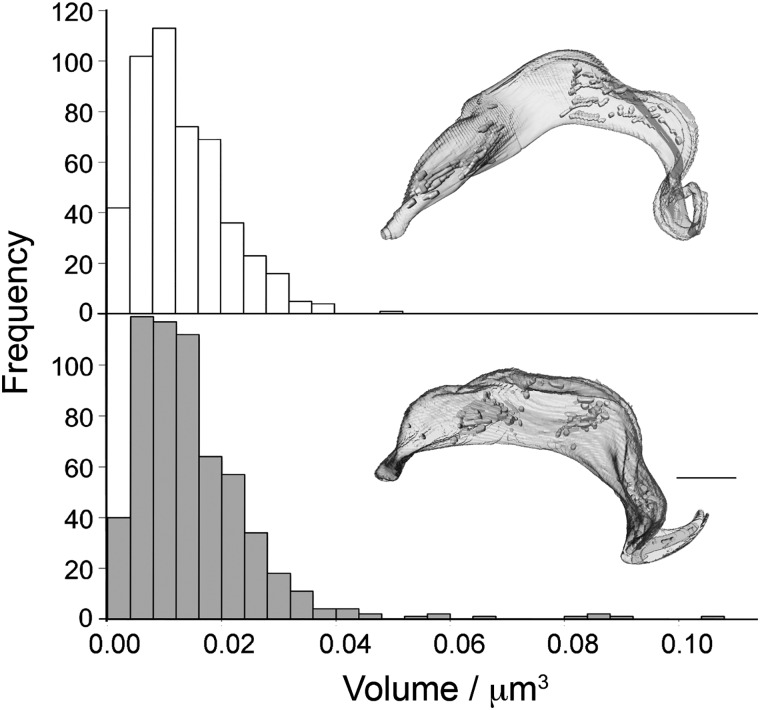
**Suramin has minimal impact on glycosome cellular frequency.** Cells were cultured in the presence of suramin for either 0 h (*top*) or 72 h (*bottom*). The respective distribution of glycosome volumes and for cells treated for 72 h with suramin is shown as a bar graph. Upon treatment with suramin, median individual glycosome volume increased from 0.0113 to 0.0127 μm^3^ (*p* < 0.037). Example segmentations of whole *T. brucei* cell are shown for both cases. *Scale bar*, 2 μm.

**Figure 4.**
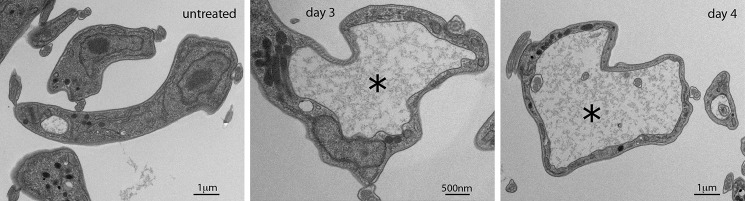
**Suramin leads to an enlarged flagellar pocket.** Cells were grown in the presence or absence of suramin (35 nm) over 4 days and analyzed by thin-section transmission EM. Images show ultrastructure in untreated cells or suramin-treated cells after 3 or 4 days. Enlarged flagellar pockets are marked by an *asterisk*. The day 3 micrograph is representative of 7 of 15 thin sections across the flagellar pocket, whereas day 4 is representative of 14 of 22 flagellar pocket cross-sections. In untreated cells, a large pocket is observed in 2 of 63.

The number and volume of glycosomes per cell was measured by three-dimensional EM (Movie S1 and Fig. 3). Following treatment with suramin, the median number of glycosomes per cell increased from 61 to 73 (*p* = 0.009, *n* = 10 cells). Total glycosomal volume increased from 0.81 ± 0.22 to 1.079 ± 0.27 μm^3^ (*p* = 0.024, *n* = 10 cells). The volume of individual glycosomes expanded by 12% following 3 days of suramin treatment, with a median volume of 0.0113 μm^3^ for untreated *versus* 0.0127 μm^3^ for treated cells (*p* < 0.037). To compare individual glycosome volumes, eight untreated and treated cells were analyzed, representing 473 and 592 glycosomes, respectively. Despite most glycosomes retaining volumes within the range of untreated cells, there was a small increase in exceptionally large glycosomes in suramin-treated cells (Fig. 3). However, a significant morphological abnormality, specifically enlargement of the flagellar pocket, was observed (Fig. 4). This so-called “Big Eye” phenotype is likely indicating disruption to endocytosis, possibly as a result of decreased cellular ATP levels (37). Therefore, immunoblotting, imaging, and ultrastructural morphometry failed to indicate substantial changes to the glycosome but did suggest abnormalities to energy production.

### Suramin perturbs mitochondrial membrane potential and ATP levels

**Figure 5.**
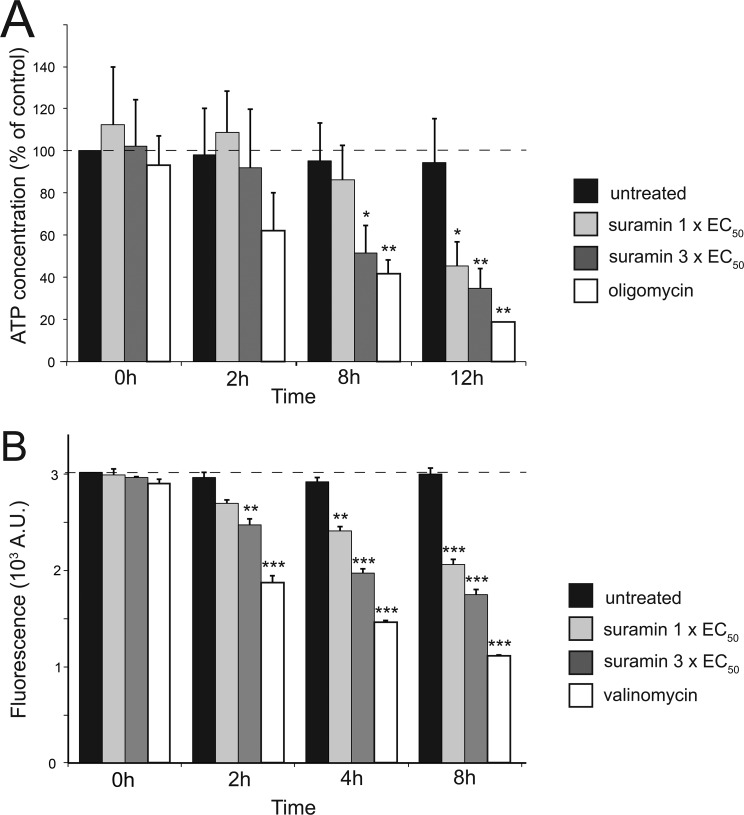
**Suramin impacts both cellular ATP production and mitochondrial membrane potential.** Cells were exposed to 35 nm (1× EC_50_) and 105 nm (3× EC_50_) suramin and analyzed at the indicated time points. *A*, the intracellular ATP concentration was measured by a bioluminescent assay with oligomycin (2 μg/ml) as positive control. Values are presented as percentage *versus* untreated control. *B*, Ψm changes were determined using the accumulation of the fluorescent indicator dye tetramethylrhodamine ethyl ester. Valinomycin (100 nm) was used as a depolarization control. For both *panels*, *bars* show the average and S.E. of three independent determinations, and statistical differences were determined using Student's unpaired two-tailed *t* test: *, *p* < 0.05; **, *p* < 0.01; ***, *p* < 0.001 relative to untreated control.

**Figure 6.**
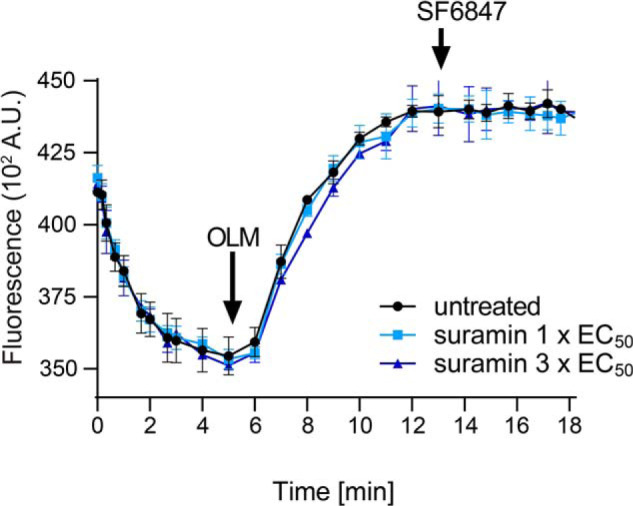
**F_o_F_1_-ATPase activity is not affected by suramin.** *In situ* generation of the mitochondrial membrane potential in the presence of ATP in digitonin-permeabilized cells untreated or pretreated with suramin for 8 h prior to permeabilization. *OLM*, oligomycin (2.5 μg/ml). SF6847 (250 nm) is an uncoupler. The displayed results represent the average activities obtained from three independent measurements.

Treatment with suramin led to dose- and time-dependent reductions in cellular ATP levels, becoming significant after 8 h when the ATP level fell to ∼50% of untreated cells (Fig. 5*A*). Further, after 2 h of incubation with 1× or 3× EC_50_ suramin, mitochondrial membrane potential (MMP) decreased by 10 and 20%, respectively, attaining a ∼50% decrease after 8 h and 3× EC_50_ suramin (Fig. 5*B*). In BSFs, the MMP is maintained by the reverse activity of the F_1_F_o_-ATPase complex, which hydrolyzes cytosolic ATP imported into the mitochondrial lumen (38, 39). We therefore measured the ability of suramin to affect the ability of the F_1_F_o_-ATPase complex to energize mitochondrial membranes. Untreated BSFs were used to establish the rate of safranin O quenching by energized mitochondria in the presence of ATP. A baseline for rapid membrane depolarization was established with oligomycin addition (Fig. 6). Oligomycin led to complete mitochondrial membrane depolarization because the addition of the uncoupler SF_6487_ caused no further increase in fluorescence and was indistinguishable between suramin-treated and -untreated cells (Fig. 6). This confirmed that ATP import and F_1_F_o_-ATPase proton pump activity are unaffected by suramin and that the observed decrease in MMP most likely results from low cellular ATP levels.

**Figure 7.**
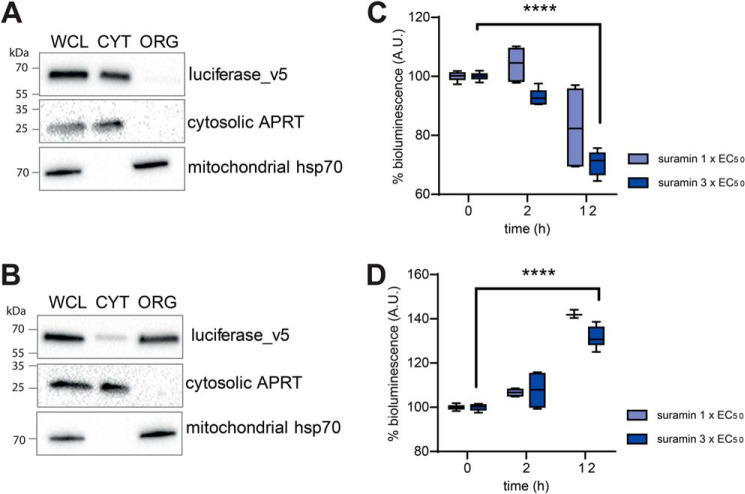
**Suramin affects levels of cytosolic and mitochondrial ATP.** *A* and *B*, subcellular localization of luciferase_v5 (*A*) and MLS_luciferase_v5 (with N-terminal mitochondrial localization signal) (*B*) was determined in BSF whole-cell lysates (*WCL*) and the corresponding organellar (*ORG*) and cytosolic (*CYT*) fraction separated by digitonin extraction. Purified fractions were analyzed by Western blotting with the following antibodies: anti-luciferase, anti-mt Hsp70 (mitochondrial marker), and anti-adenosine phosphoribosyltransferase (*APRT*) (cytosolic marker). The relevant sizes of the protein marker are indicated on the *left. C* and *D*, the cytosolic and mitochondrial ATP content was measured by a TECAN M200 plate reader upon the addition of luciferin to living cells. The *y* axis represents the percentage of the measured light units of cells treated with suramin to BSF cells, relative to those that were not treated with suramin (0-h treatment). The displayed results represent the average maximal signals obtained from three independent measurements. *Error bars*, S.E.; ****, *p* < 0.001.

To report specifically on mitochondrial and cytosolic ATP levels, BSFs expressing firefly luciferase fused with three C-terminal v5 tags in tandem with or without an N-terminal mitochondrial localization signal (MLS) were generated. The proportions of luciferase present in the cytosol or mitochondrion were verified by immunoblotting (Fig. 7). Luciferin luminescence indicates that suramin causes a decrease in cytosolic ATP levels as expected but that this is concomitant with an increase in mitochondrial ATP levels (Fig. 7, *C* and *D*, respectively).

### Suramin triggers pyruvate accumulation and proline catabolism

**Figure 8.**
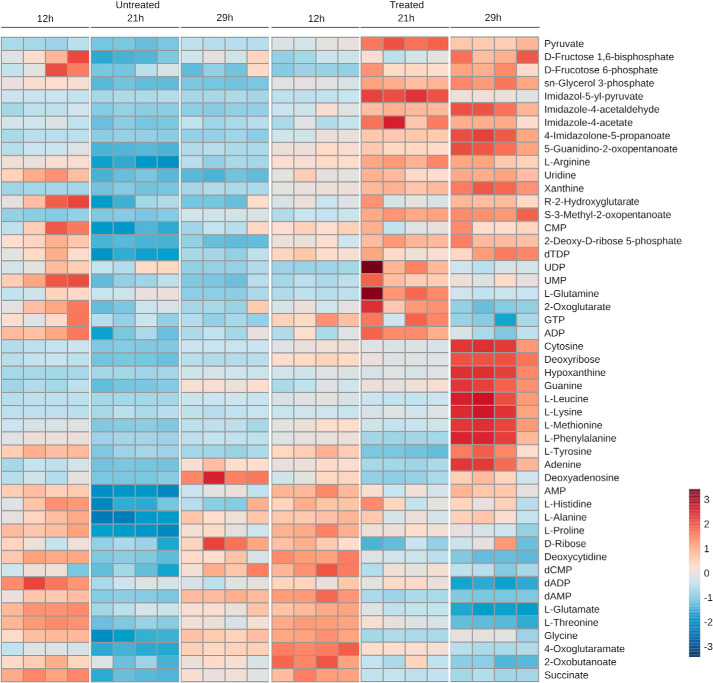
**Metabolite changes upon suramin treatment.** Heat map of selected metabolites quantified at the indicated time points in cells after suramin treatment and nontreated controls. *Colors* in the *scale bar* (*right*) correspond to intensities normalized to the 0-h point.

Global metabolomics analysis, labeling cells with [^13^C]glucose after 24-h suramin treatment at the EC_50_, indicated that 85 metabolites were significantly changed in abundance (Fig. S1 and Table S1) and that several essential pathways were impacted. Given that ATP levels and MMP were significantly altered, analysis at 12, 21, and 29 h of suramin treatment was performed to identify and track primary metabolic effects. Both cells and spent culture medium were analyzed, the latter to determine changes to uptake or secretion of specific metabolites. In this second series of experiments, glucose labeling was omitted as ^13^C isotope distributions were essentially unchanged between suramin-treated and -untreated cell extracts at 24 h, indicating no rapid severe changes to glucose metabolism. From this data set, 48 metabolites, identified against an authentic standard and/or by MS fragmentation pattern, were identified as significantly changed in the cell extracts (Fig. 8 and Tables S2 and S3).

Notably, levels of the oxidative stress sentinel metabolite ribose 5-phosphate (40) were not significantly altered. Among the most striking changes was accumulation of pyruvate (Fig. 8). Pyruvate was only slightly increased after 12 h, but this increased nearly 5-fold after 21 h. This was highly unexpected, as intracellular pyruvate is in the millimolar range in untreated trypanosomes (41). Increasing pyruvate concentration by inhibition or knockdown of the plasma membrane pyruvate transporter TbPT is sufficient to cause cytosolic acidification and death (41–43), as the transporter extrudes one proton per pyruvate as is common for other monocarboxylate transporters (44). We tested whether overexpression of TbPT1 could protect trypanosomes from suramin toxicity but found no shift in suramin potency (Fig. S7).

Concomitant with this increase in intracellular pyruvate, phosphoenolpyruvate (PEP) levels dropped by 50% at 21 h, which may suggest increased pyruvate kinase activity or, alternatively, pyruvate phosphate dikinase (PPDK) conversion of PEP to pyruvate. Lactate levels also appear elevated, but identification of this metabolite is low-confidence (Table S2). Glycerol 3-phosphate, a late glycolytic intermediate, is raised at 21 h, as are the upstream glycolytic intermediates fructose 1,6-bisphosphate and fructose 6-phosphate. However, the latter metabolite can also be produced through the pentose phosphate pathway (PPP) via transketolase and glycerol 3-phosphate from dihydroxyacetone phosphate, which itself is downstream of fructose 1,6-bisphosphate and fructose 6-phosphate. Any of these interpretations would suggest increased glucose uptake, but metabolomics evidence is insufficient to discriminate between these possibilities.

**Figure 9.**
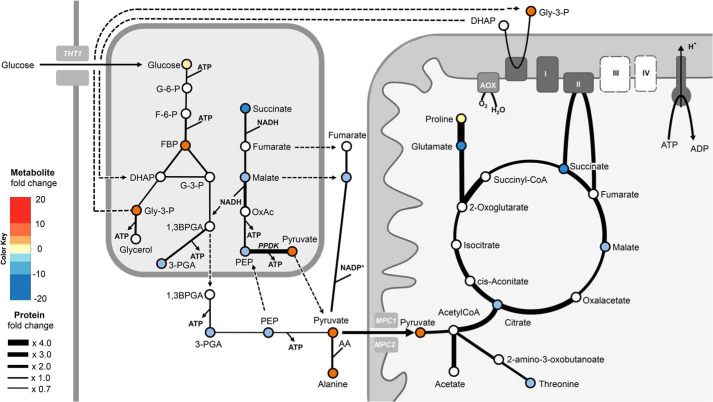
**Suramin impact on glycosomal and mitochondrial pathways.** Enzymatic steps and transport processes are represented by *black arrows* with *different thickness* dependent on the observed abundance change of the respective protein as a result of 48-h suramin treatment (for ratio changes, see the *key* on the *left*). The respective metabolites changes upon 29 h of suramin treatment are *color-coded* (*white circles*, not determined). *Dashed lines* indicate transport processes, where the transport proteins are unknown.

An early and transient change was observed in the levels of some intracellular amino acids and Krebs' cycle intermediates, suggesting a primary effect (Fig. S2). This cohort includes glutamate, threonine, proline, alanine, and succinate. A decrease in serine, threonine, and glutamate is consistent with up-regulation of proline and threonine catabolism. Glutamate is an intermediate in proline degradation (45) (Fig. 9) and the ultimate end product of proline catabolism is alanine, which is excreted (46). Alanine levels were unchanged in culture supernatants, but, by contrast, proline was decreased ∼10%, consistent with activated proline catabolism. Notably, elevated ornithine levels are accompanied by elevated *N*-acetylputrescine, indicative of increased putrescine, the product of ornithine decarboxylase and entry into the polyamine pathway.

### Proteome changes induced by suramin indicate metabolic reprogramming

**Figure 10.**
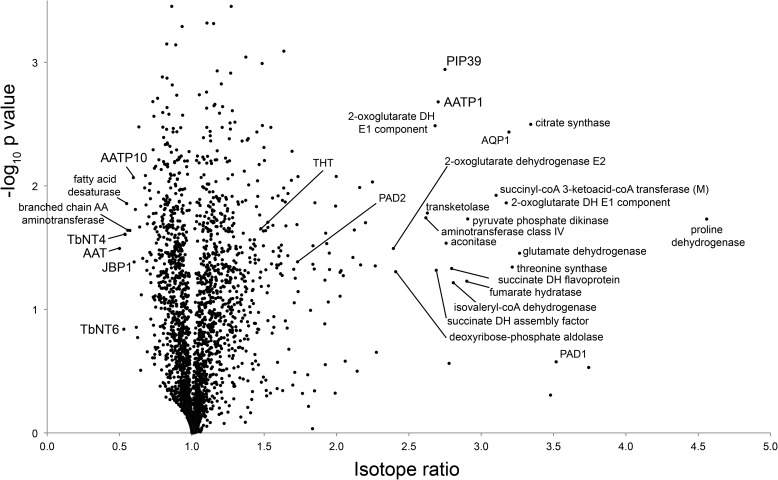
**Global proteome changes upon 48 h of suramin exposure.** Shown is a volcano plot of normalized SILAC ratios, averaged from triplicate experiments, plotted against the respective −log_10_-transformed *p* values. Selected data points are annotated. For complete annotation, see Table S4. *DH*, dehydrogenase; *AQP*, aquaporin; *AA*, amino acid; *AATP1*, amino acid transporter 1 Tb927.8.7610; *AATP10*, amino acid transporter 10 Tb927.4.4820; *NT4*, adenosine transporter Tb927.2.6220; *AAT*, amino acid transporter Tb927.4.4830; *JBP1*, J-binding protein 1 Tb927.11.13640; *PAD*, protein associated with differentiation; *PIP39*, PTP1-interacting protein, 39 kDa.

Stable-isotope labeling by amino acids in culture (SILAC) was used to compare untreated cells with cells exposed to suramin at the EC_50_ after 1 and 2 days of exposure to provide a kinetic context, 1 day being considered to allow proteome changes to become significant and 2 days to capture additional changes that may correlate with later changes to the metabolome. 4019 protein groups were identified, representing ∼50% of the *T. brucei* proteome, 2577 of which could be quantified in two replicates (Table S4 and Fig. 10). Surprisingly, after 1 day, the proteome was essentially unchanged (Table S4 and Fig. S3), which indicated that significant metabolic changes precede observable proteome alterations. However, at 2 days, significant alterations to protein abundance were detected.

A 1.5-fold increase in the trypanosome BSF-specific hexose transporter THT1 (47) indicates an increased glucose uptake capacity, which was expected from the alterations in PEP/glycolytic intermediates. However, with the exception of a 3-fold increase in PPDK and, to a lesser extent, those enzymes involved in the succinate malate shunt, the abundance of proteins engaged in glycolysis was unaltered (Fig. 9 and Fig. S4). PPDK reversibly converts PEP into pyruvate in procyclic form (PCF) cells grown in the presence of glucose, functioning in the glycolytic direction, generating ATP in the glycosome (48). Whereas the glycosomal content of glycolytic enzymes is globally reduced in the PCF (from ∼90% of the organellar protein content in BSF to under 50% in PCF (49)), exceptionally PPDK and succinate malate shunt enzymes have a much higher abundance in PCF (50). This suggests that suramin-treated cells might partially maintain the glycolytic ATP/ADP and NADH/NAD ratio using PCF-like pathways. Transketolase, a central enzyme in the nonoxidative branch of the PPP (51) with glycosomal and cytosolic localizations (52), increased ∼2.6-fold, also consistent with the observed increases in fructose 6-phosphate and also, indirectly, fructose 1,6-bisphosphate, and likewise correlates with a partial induction of a PCF-like expression profile.

Further, suramin significantly increased the abundance of multiple mitochondrial proteins mostly associated with functions in PCFs (Fig. 9 and Fig. S4). All enzymes of the Krebs' cycle were up-regulated between 2- and 3-fold (Table S4 and Fig. S4). A >4-fold increased abundance of proline dehydrogenase, along with ∼2-fold up-regulation of Δ^1^-pyrroline-5-carboxylate dehydrogenase, which converts γ-glutamate semialdehyde into glutamate (53), together with 3-fold increased glutamate dehydrogenase suggests increased proline catabolism, a further feature of PCF metabolism and concordant with the metabolomics findings here (53). Threonine synthase, generating threonine from *O*-phosphohomoserine, is also up-regulated (54). Threonine metabolism is a major source of acetyl-CoA in both the PCF and BSF stages (55, 56), which is further converted to acetate by a coupled ATP-generating reaction of two enzymes (57), succinyl-CoA:3-ketoacid CoA transferase and succinyl-CoA synthase, which are also both increased (Fig. 9 and Fig. S4). Acetate is, upon transport to the cytosol, converted back to acetyl-CoA by cytosolic acetyl-CoA synthetase and feeds into lipid synthesis (58). Altogether, this suggests that mitochondrial activity, and in particular ATP generation pathways, is significantly increased (Fig. 9 and Fig. S4).

Moreover, the abundance of the mitochondrial inner membrane pyruvate carriers, TbMPC1 and -2, that shuttle cytosolic pyruvate for oxidation to acetyl-CoA and possibly fuel the Krebs' cycle due to higher levels of citrate synthase (59), was increased 2-fold, consistent with the rapid increase of intracellular pyruvate (see below). Of 11 further carrier proteins in the inner mitochondrial membrane detected, two were significantly up-regulated: MCP20 (1.6-fold), a putative SAM transporter (60), and MCP12 (1.8-fold), a 2-oxoglutarate/dicarboxylate transporter homologue (61) that transports dicarboxylates and tricarboxylates across the mitochondrial inner membrane (62). The up-regulation of these mitochondrial importers indicates routing of substrates to mitochondrial metabolism and is further corroboration of this effect (Fig. 9).

### Proteome alterations are similar to differentiation after suramin exposure

Significantly, a cohort of proteins associated with differentiation from long slender (LS) to short stumpy (SS) forms were detected with increased abundance, including PAD (protein associated with differentiation) 1 and 2, which increased >3-fold and ∼2-fold, respectively; PIP39 increased ∼3-fold (TbPTP1-interacting protein, 39 kDa), a phosphatase that upon phosphorylation translocates into glycosomes and promotes the differentiation pathway (63); and protein kinases NRKA/B (key regulators of differentiation and established markers of BSF-SS cells) increased 2-fold (64).

**Figure 11.**
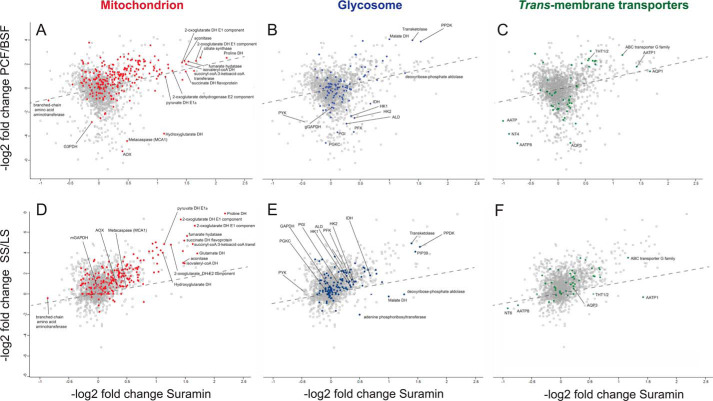
**Correlation of proteome changes upon suramin treatment with proteome differences between BSF and PCF and BSF-LS and BS-SS.** −log_2_-transformed abundance shifts after 48 h of suramin (35 nm) exposure were plotted against corresponding −log_2_-transformed abundance differences between BSF and PCF (*A–C*) (50) and between BSF-LS and BSF-SS (*D–F*) (65). Selected GO terms are indicated by *color*: *red*, mitochondrion; *blue*, glycosome; *green*, transmembrane transporter activity. Infinite changes were omitted for clarity. The BSF-SS data are derived from the pleomorphic strain EATRO1125 (65). *DH*, dehydrogenase; *PPDK*, pyruvate phosphate dikinase; *PYK*, cytosolic pyruvate kinase; gGAPDH, glycosomal glyceraldehyde 3-phosphate dehydrogenase; *PGI*, phosphoglucose isomerase; *HK*, hexokinase; *IDH*, isocitrate dehydrogenase; *PGKC*, phosphoglycerate kinase; *PFK*, phosphofructokinase; *ALD*, aldolase; *G3PDH*, mitochondrial glycerol-3-phosphate dehydrogenase; *AOX*, alternative oxidase; NT4, adenosine transporter Tb927.2.6220; *AATP*, amino acid transporter; *AQP*, aquaporin.

**Figure 12.**
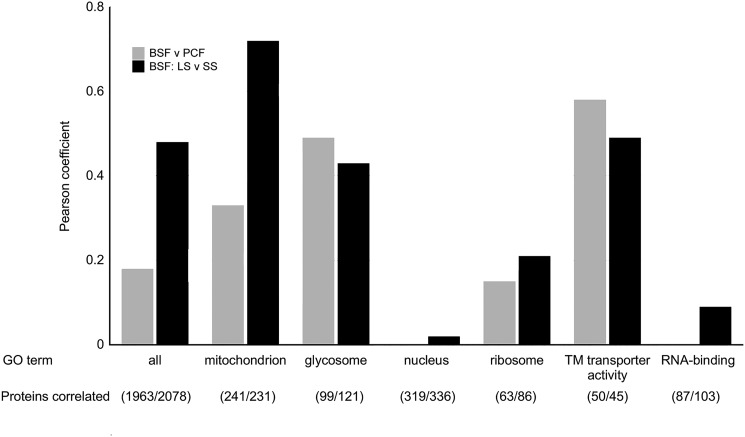
**Correlation of proteome changes upon suramin treatment with proteome differences between BSF and PCF and between BSF-LS and BS-SS.** Shown is a bar graph of Pearson coefficients from correlations of proteome changes upon suramin treatment between BSF and PCF (*gray*) (50) and between BSF-LS and BSF-SS (*black*) for all shared, finite quantified protein groups and selected GO terms. The BSF-SS data are derived from the pleomorphic strain EATRO1125 (65). Corresponding correlation plots are shown in Fig. 11 (*BSF-SS*) and Fig. S5 (*PCF*).

The similarities between these changes and the PCF proteome were fully revealed when the changes observed upon suramin treatment were correlated with those associated with differentiation. Proteome differences between BSF and PCF (50) (Fig. S5) and BSF-LS and BSF-SS forms (65) (Fig. 11) were used, where the former comparisons used monomorphic Lister 427 and the latter used the pleomorphic EATRO1125 strain. In both cases, data in the correlation plots were elliptically distributed, with Pearson coefficients of 0.18 (BSF/PCF, 1963 proteins) and 0.49 (BSF-LS/BSF-SS, 2078 proteins), respectively, for all finite data. These Pearson coefficients, when restricted to mitochondrial proteins only, increase to 0.33 (BSF/PCF, 241 proteins) and to 0.72 (BSF-LS/BSF-SS, 231 proteins), respectively (Fig. 12). Similarly, GO term categories “glycosome” and “transmembrane transporter activity” were enriched, whereas GO categories such as “ribosome,” “nucleus,” and “RNA-binding” display poor correlation (Fig. 12 and Fig. S5). Notably, enzymes associated with PCF metabolism were up-regulated, whereas levels of those associated with BSF-LS metabolism were unaltered (Fig. S5). This is most obvious for the glycosome, where glycolytic enzymes (*e.g.* hexokinase, glyceraldehyde and 3-phosphate dehydrogenase; Fig. S11) were unaffected, as is the case in BSF-SS, but distinct from PCF, where this protein cohort is dramatically down-regulated. Similarly, levels of mitochondrial FAD-dependent glycerol-3-phosphate dehydrogenase and alternative oxidase, the cytochrome-independent terminal oxidase for BSF respiration (66), correlated in suramin-treated and BSF-SS cells but are massively down-regulated in PCF cells.

In summary, prolonged suramin exposure leads to an activated mitochondrion, quite probably in response to falling cellular ATP levels. It appears there may also be an attempt to generate ATP in the glycosome via PEP metabolism. Proteome alterations closely resemble the BSF-SS form, certainly as far as energy metabolism and expression of differentiation-associated proteins is concerned, whereas there is no indication of further differentiation into true PCF forms.

## Discussion

Understanding mechanism of action is crucial for connecting interactions between biological systems and xenobiotics. Recent rapid progress in determining the mechanisms of action for trypanocidal drugs currently in the clinic or moving through development pipelines has clarified how many of these interactions operate. Frequently, these advances have revealed exquisite subtlety behind the provision of drug specificity (9, 12). For suramin the mechanism for cellular entry, exploiting a trypanosome-specific surface protein as receptor, has been proposed, but subsequent events within the cell have remained poorly defined (12, 21). Here, we examined multiple facets of suramin interactions with trypanosomes to uncover significant and specific impacts on energy production, together with potential activation of compensatory mechanisms.

Suramin consists of two symmetric trisulfonated naphthylamine groups with six negative charges at physiological pH, and thus once internalized, it is trapped within the cell. Uptake through ISG75 endocytosis enables selectivity as well as rapid accumulation (12, 21). Size-exclusion chromatograph of [^3^H]suramin incubated with serum revealed promiscuous protein binding (Fig. S6), likely explaining the extraordinarily >40-day plasma half-life, (67), and indicating that intracellular accumulation of suramin *in vivo* could become considerable, with likely only a modest proportion of the compound remaining unbound.

Suramin apparently inhibits the functions of a wide variety of proteins (22, 24, 31, 68–70), but, as there is no obvious structural similarity uniting these targets, a low-specificity mechanism likely operates, and efficient cellular accumulation is probably also key to efficacy. Notably, multiple cellular pathways are involved in suramin sensitivity in trypanosomes, and the genetic sensitivity profile is significantly more complex than for other trypanocidal drugs (12). Whereas suramin inhibits isolated glycosomal enzymes in the micromolar range (32), which are certainly attainable within (some parts of) the cell (Fig. 1), an increase in glycolytic enzyme levels or metabolites unique to the glycolytic pathway is not observed. However, a fall in cytosolic ATP is substantial and significant, with a rapid onset, and, as there is no other significant cytoplasmic ATP-generating mechanism, argues for impediment of glycolytic ATP production. However, despite a thorough interrogation of glycolysis and glycosomal number and composition, the exact molecular mechanism responsible for this inferred glycolytic disruption remains elusive (see below).

Metabolomics identified increased PPP metabolites as well as possible late glycolytic intermediates and the overproduction of pyruvate. Significantly, the only elevated glycolytic intermediates are common with the PPP, and their production via this pathway is supported by increased abundance of transketolase and other enzymes. Thus, increased production of pyruvate is inferred to be from PEP by gPPDK, another potential ATP-generating route normally only active in the insect stage. An increase in Krebs' cycle activity is supported by proteomics and occurs considerably later, following suramin administration, and we suggest that the Krebs' cycle and PPP activation are both likely to be secondary impacts, representing an (ultimately futile) attempt by the trypanosome to generate energy following the collapse of cytosolic ATP. An increase in abundance of the glucose transporter THT1 also attests to an increased draw on glucose requirements. Regardless, from the rather diverse impact of suramin on trypanosome metabolism, we also cannot exclude polypharmacology as a mechanism (*i.e.* the inhibition of multiple enzymes and hence metabolic pathways).

Many enzymes classically believed to be exclusively associated with procyclic metabolism are detectable in BSF cells by high-sensitivity LC-MS/MS, albeit at low abundance (71). This may enable rapid adaptation to multiple extravascular niches in the host, such as cerebrospinal fluid, subcutaneous areas, and adipose tissue, which offer nutrient opportunities distinct from peripheral blood, or reflect the fact that most genes are not regulated by heterochromatinization and hence differential expression is restricted to 2 or 3 orders of magnitude. Suramin increased expression of many of these proteins, indicating an expression profile that has some resemblance to the procyclic form. These changes are far removed from a complete differentiation, however, and include an absence of down-regulated glycolysis or a full mitochondrial activation. Expression of stumpy stage transitional markers, including PAD1 and -2, is also suggestive of a stumpy-like expression profile. The differentiation to cell cycle–arrested stumpy forms has been suggested as having multiple roles in enhancing transmission, including limiting parasitemia and attuning parasites for their new environment in the tsetse fly (72, 73). Multiple manipulations of culture conditions, as well as the creation of specific genetic lesions, invoke partial stumpy pathway activation and/or expression of protein markers, including PAD1. Significantly, AMPKα1, implicated in the initiation of this differentiation process (74–76), is up-regulated following suramin treatment, and the changes observed here are consistent with AMPKα1 activation. However, whether this is cause or effect is unclear.

**Figure 13.**
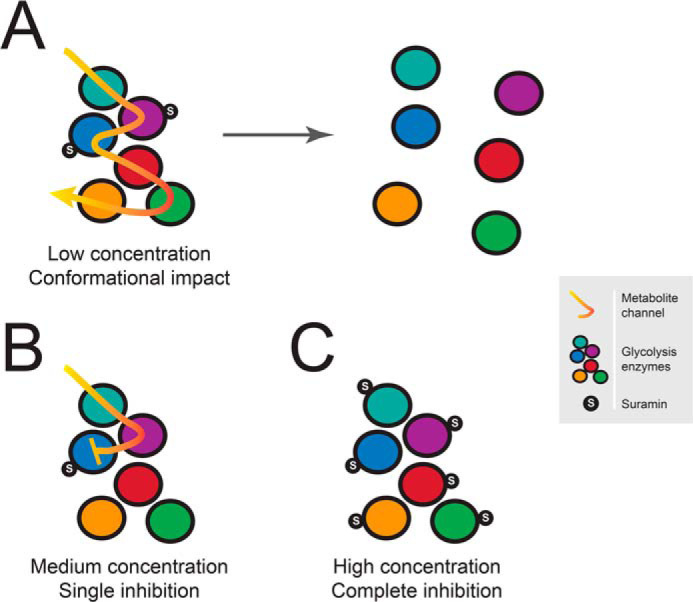
**Schematic view of potential impact of suramin on glycolytic complexes.** There are several lines of evidence that suramin is a promiscuous binder, and multiple diverse low-affinity targets have been described. It is possible that suramin binding disrupts glycolytic protein complexes even at low concentration through conformational impact (*A*). Due to rapid accumulation of suramin by endocytic uptake, higher concentrations could be reached, potentially resulting in complete inhibition of single (*B*) or multiple protein targets (*C*) within a complex. All of these scenarios would ultimately lead to a decrease in glycolytic flux and hence ATP production.

To conclude, suramin has many effects on the trypanosome cell, and many of these are likely the consequence of a central collapse to ATP production, presumably by the glycolytic pathway. Activation of the pentose phosphate and mitochondrial ATP production pathways, together with changes to protein abundance normally associated with differentiation, are possible consequences of this decrease in ATP production. Whereas the concentration of suramin required to inhibit glycolytic enzymes is significantly greater than that required to kill trypanosomes, it is possible that this concentration is achieved by cellular accumulation though endocytosis. It is also a possibility that the glycolytic system, suggested to use substrate channeling and thus to have physical interactions between the enzymes, can be disrupted by lower levels of suramin than required to inhibit the individual enzymes, leading to decreased flux and hence ATP production, but without obvious changes to protein abundance (Fig. 13). Finally, the concept of leveraging induction of differentiation by pharmacological intervention was proposed as a therapeutic opportunity (77, 78); ironically, suramin has likely been exploiting such a strategy for more than a century.

## Materials and methods

### T. brucei brucei culturing and transfection

BSF Molteno Institute Trypanosomal antigen type (MITat) 1.2, derived from Lister strain 427, was cultured in HMI-11 complete medium (HMI-11 supplemented with 10% fetal bovine serum non-heat-inactivated, 100 units/ml penicillin, 100 units/ml streptomycin) (79) at 37 °C with 5% CO_2_ in a humid atmosphere, in culture flasks with vented caps. 2T1 cells, a variant of Lister 427 (80), were maintained in HMI-11 complete medium in the presence of phleomycin (0.5 μg/ml) and puromycin (1 μg/ml). Following transfection with stem-loop RNAi plasmids or inducible overexpression plasmids, 2T1 cells were maintained in phleomycin (0.5 μg/ml) and hygromycin (2.5 μg/ml) (80, 81). Experiments were performed following 48-h induction with tetracycline (1 μg/ml). Cells were maintained at densities between 1 × 10^5^ and 2.5 × 10^6^ cells/ml. The suramin EC_50_ was assessed following 3 days of TbPT1 overexpression. Cells were plated at 1 × 10^4^ cells/ml in 96-well plates in a 2-fold dilution series of suramin, starting from 1 μm. After 3 days of growth, 20 μl of resazurin (Sigma) at 125 μg/ml in PBS was added to each well and incubated for a further 6 h at 37 °C. Fluorescence was determined using a Gemini Fluorescent Plate Reader (Molecular Devices) with the following settings: excitation, 530 nm; emission, 585 nm; filter cutoff, 570 nm. Data were processed in Excel; nonlinear regression analysis was carried out in GraphPad Prism using an equation for a sigmoid curve with variable slope.

### Plasmid constructs

ORF-specific RNAi fragments of 400–600 bp were amplified with PCR primers designed using RNAit (82) and cloned into pRPa^iSL^ to generate stem-loop, “hairpin” dsRNA to induce RNAi knockdown (80). All constructs were verified by Sanger sequencing. ISG75 was overexpressed in pXS5ISG75HA (34). The following primers were used to amplify the ORF of TbPT1 for cloning into pRPa^TAG^: 5TbPT1Pac1 (5′-GCGCGCTTAATTAATGGTTTACCTTGTCGATGACCTTGC-3′) and 3TbPT15XbaR (5′-GCGCGCTCTAGACTGCTGCGCTGCTCCGCCACTGTG-3′). Prior to introduction into trypanosomes, pRPa^iSL^ and pRPa^TAG^ constructs were linearized with AscI, and pXS5 constructs were linearized with XhoI.

### Ultrastructural analysis

Bloodstream-form *T. brucei* strain Lister 427, exposed or not to 35 nm suramin (1× EC_50_) in culture medium for 4 days, were prepared for EM as described previously (83). Briefly, cells were fixed isothermally in culture with 2.5% (v/v) glutaraldehyde at 37 °C prior to harvesting by centrifugation (800 × *g* for 15 min). Following fixation, samples were post-fixed in 1% (w/v) osmium tetroxide in PBS for 30 min at room temperature and *en bloc* stained with 1% (w/v) aqueous uranyl acetate. Following dehydration through an acetone series, samples were embedded in epoxy resin. Ultrathin (70-nm) sections were post-stained with 2% uranyl acetate and lead citrate and imaged on a Tecnai G2 transmission electron microscope (FEI, Hillsboro, OR, USA). Resin blocks were also imaged on a Zeiss Gemini/Merlin scanning electron microscope, and serial sections were generated using a Gatan 3view serial sectioning system. Image resolution was 8000 × 8000 pixels with a pixel size of 3 nm^2^ and a *z*-resolution of 100 nm. Initial data processing was performed using IMOD (84).

Ten cells in G_1_ phase of the cell cycle were selected from each sample. Each cell was then segmented and reconstructed using Amira software (versions 5.3.3 to 6.1, FEI, Eindhoven, Netherlands) so that glycosome volume and number could be calculated (Fig. 3) along with whole-cell volume. Individual glycosome volumes were calculated for eight cells from both the untreated and treated samples. Statistical comparisons of these parameters were performed using IBM SPSS Statistics 23. Total glycosome volumes per cell were normally distributed for each group, and hence a *t* test was applied, whereas for the remaining statistical analyses, Mann–Whitney U was the test selected, as none of the variables assessed were normally distributed.

### Protein electrophoresis and immunoblotting

Proteins were separated by electrophoresis on 12.5% SDS-polyacrylamide gels and then transferred to polyvinylidene difluoride membranes (Immobilon, Millipore) using a wet transfer tank (Hoefer Instruments). Nonspecific binding was blocked with TBS with 0.2% Tween 20 (TBST) supplemented with 5% freeze-dried milk, and proteins were detected by incubation with primary antibody diluted in TBST with 1% milk for 1 h at room temperature. Antibodies were used at the following dilutions: mouse monoclonal anti-HA (sc-7392, Santa Cruz Biotechnology, Inc.) at 1:10,000, rabbit monoclonal anti-Myc (7E18, Sigma) at 1:5000, mouse monoclonal anti-v5 (37-7500, Invitrogen) at 1:1000, rabbit polyclonal anti-ISG65 and anti-ISG75 both at 1:10,000 (from M. Carrington (University of Cambridge, Cambridge, UK) and P. Overath (University of Tubingen, Germany), respectively), KMX-1 anti-β-tubulin at 1:2000 (MAB3408, Millipore), rabbit anti-CatL at 1:1000 (85), mouse anti-GLP-1 at 1:1000 (86), rabbit anti-PFK (Do405, 1:50,000), rabbit anti-PGK (Do425, 1:100,000), and rabbit anti-PYK (SB953, 1:100,000) (all from P. Michels, University of Edinburgh, UK). Following three washes of 10 min with TBST, the membrane was incubated in secondary antibody diluted in TBST with 1% milk for 1 h at room temperature. Commercial secondary anti-rabbit peroxidase-conjugated IgG (A0545, Sigma) and anti-mouse peroxidase-conjugated IgG (A9044, Sigma) were used, both at 1:10,000. Detection was by chemiluminescence with luminol (Sigma) on BioMaxMR film (Kodak). Densitometry quantification of relative protein level was achieved using ImageJ software (National Institutes of Health).

### Immunofluorescence

Samples were prepared as described previously (87). Antibodies were used at the following dilutions: mouse and rabbit anti-HA epitope IgG (sc-57594 and sc-7392, Santa Cruz Biotechnology) at 1:1000, mouse 9E10 anti-Myc at 1:1000 (Sigma), rabbit anti-ISG75 (from P. Overath) at 1:1000, rabbit anti-CatL at 1:1000 (85), and mouse anti-GLP-1 at 1:1000 (86). Rabbit anti-PFK (Do405, 1:1000), rabbit anti-PGK (Do425, 1:1000), and rabbit anti-PYK (SB953, 1:500) were all from P. Michels. Secondary antibodies were used at the following dilutions: anti-mouse Oregon Green (Molecular Probes) at 1:1000 and anti-rabbit Cy3 (Sigma) at 1:1000. Cells were examined on a Nikon Eclipse E600 epifluorescence microscope fitted with optically matched filter blocks and a Hamamatsu ORCA CCD camera. Digital images were captured using Metamorph software (Universal Imaging Corp.), and raw images were processed using Adobe Photoshop CS3 (Adobe Systems Inc.).

### Suramin uptake

Parasites from 2T1, ISG65 RNAi, ISG75 RNAi, and ISG75–overexpressing lines were grown in HMI-11 medium supplemented with 10% fetal bovine serum in the presence of 1 μm/ml tetracycline for 60 h at 37 °C in humid atmosphere with 5% CO_2_. Cells were then harvested by centrifugation at 1000 × *g* and washed twice with uptake assay buffer (33 mm HEPES, 98 mm NaCl, 4.6 mm KCl, 0.5 mm CaCl_2_, 0.07 mm MgSO_4_, 5.8 mm NaH_3_PO_4_, 0.03 mm MgCl_2_, 23 mm NaHCO_3_, 14 mm glucose, pH 7.3). To determine the linear phase of uptake of [^3^H]suramin, 1 × 10^7^ cells resuspended in uptake assay buffer were incubated with 250 nm [^3^H]suramin (20 Ci/mmol; American Radiolabeled Chemicals) for preset times up to 60 min. The saturability of uptake was determined by incubation of cells with 250 nm [^3^H]suramin in the presence of 100 μm unlabeled suramin, which yielded a rate of uptake not significantly different from zero (*p* > 0.4; F-test). We observed two phases of suramin uptake: 1) from 0 to 20 min and 2) from 20 to at least 60 min (*r*^2^ > 0.97). To compare the rates of suramin uptake for the four cell lines and avoid any early effect caused by the internalization of the drug, 1 × 10^7^ cells of each cell line were incubated with 200 nm [^3^H]suramin for 15 min, consistently within the first linear phase of transport, in assay buffer only or in the presence of 100 μm unlabeled suramin. The reaction was stopped by the addition of 400 μm unlabeled suramin in uptake assay buffer followed by immediate centrifugation through an oil layer (88). The cell pellet was collected and lysed by the addition of 500 μl of 2% SDS solution and agitated for at least 1 h. Then 3 ml of scintillation fluid (Scintilogic U, Lablogic) was added to each vial and left under gentle agitation (40 rpm) for 16 h, after which time vials were manually vigorously agitated, and scintillation was measured in a 300SL (Hidex) scintillation counter. The comparison between cell lines is based on four independent assays in triplicate.

### Determination of ATP levels

Changes in cellular ATP levels due to the exposure of trypanosomes to suramin at 1× EC_50_ (35 nm) and 3× EC_50_ (105 nm) concentrations were monitored using the Molecular Probes ATP Determination Kit (A22066, Invitrogen Detection Technologies), based on the luciferin-luciferase bioluminescent enzymatic reaction. Bloodstream-form cell cultures of *T. brucei* 2T1 were incubated with and without test compound, and, at each predetermined incubation time, 10^7^ cells of each sample were transferred into a microcentrifuge tube and centrifuged at 2500 × *g* for 10 min at 4 °C. The pellet was washed twice with 1 ml of 50 mm Tris-HCl (pH 7.4) containing 0.1 mm DTT and resuspended in 150 μl of the same buffer. The samples were incubated with digitonin (40 μm) to facilitate cell membrane permeabilization. Cells were lysed by sonication on ice (twice for 10 s separated by 30 s), using a Soniprep 150 (MSE) at 8-μm amplitude. The samples were centrifuged at 10,000 × *g* for 10 min at 4 °C, and the supernatant was collected and instantly frozen in liquid nitrogen and stored at −80 °C. Oligomycin (2.0 μg/ml) was used as positive control. ATP levels were quantified using the kit above following the manufacturer's instructions. 90 μl of standard reaction solution was added to each well of a 96-well plate, and the background luminescence was recorded in a FLUOstar OPTIMA fluorimeter; 10 μl of each sample was then added to each well. The plate was incubated at 28 °C for 15 min, and the luminescence was measured, including a standard curve with an ATP concentration ranging from 1 nm to 1 μm to allow the calculation of the ATP concentrations in each sample. The ATP content was measured at 0, 2, 8, and 12 h of incubation with the test sample.

### MMP fluorimetric detection

Changes in mitochondrial membrane potential (Ψm) after incubation of trypanosomes with the suramin were determined using the indicator dye tetramethylrhodamine ethyl ester (TMRE). Cell suspensions of *T. brucei brucei* 2T1 were incubated at 1× EC_50_ (35 nm) and 3× EC_50_ (105 nm) concentrations of suramin. 3 × 10^6^ cells were harvested at each time point (0, 2, 4, and 8 h) after further incubation with TMRE (100 nm) for 30 min at 37 °C. The cells were transferred into a microcentrifuge tube and centrifuged at 2500 rpm for 10 min at room temperature. The pellet was washed once in 1 ml of PBS (pH 7.4) and resuspended in 1 ml of fresh PBS. 200 μl of cell suspension was added to each well of a 96-well black flat-bottomed plate, and the fluorescence proportional to the amount of TMRE permeated into mitochondria was recorded in a FLUOstar OPTIMA fluorimeter. Valinomycin (Sigma–Aldrich; 100 nm) was used as control for mitochondrial membrane depolarization.

### In situ ΔΨm measurement

Estimation of the ΔΨm *in situ* was done spectrofluorometrically using the indicating dye safranin O (Sigma, S2255). *T. brucei* BSF cells (6 × 10^7^ cells/ml) were resuspended in a reaction buffer containing 8 mm KCl, 110 mm potassium gluconate, 10 mm NaCl, 10 mm HEPES, 10 mm K_2_HPO_4_, 0.015 mm EGTA, 0.5 mg/ml BSA (fatty acid–free), 10 mm mannitol, 1 mm MgCl_2_, 2 mm ATP, and 5 μm safranin O, pH 7.25. The reaction was activated with digitonin (40 μm), whereas oligomycin (3 μm) and SF6847 (250 nm) were injected at specific time points during the assay. Changes in the amount of fluorescence over time were detected on an Infinite M200 microplate reader (TECAN) (λ_ex_, 496 nm; λ_em_, 586 nm).

### In vivo ATP measurement

Cytosolic and mitochondrial ATP was measured *in vivo* using ATP-dependent luciferase bioluminescence. Briefly, an equal number of BSF cells were treated with suramin at the concentration of 1× EC_50_ and 3× EC_50_ for 0, 2, and 12 h, washed with PBS (pH 7.4), and resuspended in HEPES-glu-LUC buffer (20 mm HEPES, 10 mm glucose, 116 mm NaCl, 5.6 mm KCl, 8 mm MgSO_4_, 1.8 mm CaCl_2_, pH 7.4).The emission of light was triggered by the addition of d-luciferin (50 μm; Sigma, L6882) and immediately measured on an Infinite M200 microplate reader (TECAN). The cells exhibit a maximum luminescence signal within ∼1 min after d-luciferin addition, after which the signal decayed rapidly; the measurement was taken at peak intensity.

Expression of luciferase-v5 or MLS_luciferase-v5luciferase in cytosolic and mitochondrial fractions was analyzed, tested by Western blotting using 50 μm luciferin A commercial anti-v5-antibody and antibodies recognizing the cytosolic adenosine phosphoribosyltransferase and mitochondrial Hsp70 protein.

### SILAC labeling

HMI-11 for SILAC was prepared as described previously (21). Either normal l-arginine and l-lysine (HMI11-R0K0) or l-arginine U-^13^C_6_ and l-lysine 4,4,5,5-^2^H_4_ (HMI11-R6K4) (Cambridge Isotope Laboratories) were added at 120 and 240 μm, respectively. Cells treated with 35 nm suramin (1× EC_50_) were grown in parallel with nontreated cells, in the presence of HMI11-R0K0 or HMI11-R6K4, respectively. Cultures in logarithmic growth phase were mixed after 24 and 48 h, respectively, immediately harvested by centrifugation, washed twice with PBS containing Complete Mini Protease Inhibitor Mixture (Roche Applied Science), resuspended in Laemmli buffer containing 1 mm DTT, and stored at −80 °C. Samples were generated in triplicate, and one label swap was performed. Samples were sonicated, and aliquots containing 5 × 10^6^ cells were separated on a NuPAGE bis-tris 4–12% gradient polyacrylamide gel (Invitrogen). The sample lane was divided into eight slices that were excised from the Coomassie-stained gel, destained, and then subjected to tryptic digest and reductive alkylation. Liquid chromatography tandem MS (LC-MS/MS) was performed by the Proteomic Facility at the University of Dundee. The eight fractions obtained from SDS-PAGE were subjected to LC-MS/MS on an UltiMate 3000 RSLCnano System (Thermo Fisher Scientific) coupled to a Q Exactive HF Hybrid Quadrupole-Orbitrap (Thermo Fisher Scientific). The 24-h suramin treatment set were run on an Orbitrap Velos Pro (Thermo Fisher Scientific). Mass spectra were analyzed using MaxQuant version 1.5 (89) searching the *T. brucei brucei* 927 annotated protein database (release 39.0) from TriTrypDB (90). Minimum peptide length was set at six amino acids, isoleucine and leucine were considered indistinguishable, and false discovery rates of 0.01 were calculated at the levels of peptides, proteins, and modification sites based on the number of hits against the reversed sequence database. SILAC ratios were calculated using only peptides that could be uniquely mapped to a given protein. When the identified peptide sequence set of one protein contained the peptide set of another protein, these two proteins were assigned to the same protein group. *p* values were calculated, applying *t* test–based statistics using Perseus (91). Proteomics data have been deposited to the ProteomeXchange Consortium via the PRIDE partner repository (92) with the data set identifier PXD014215.

### Metabolomics

Cultures were grown and treated with 35 nm suramin in parallel with nontreated cells in quadruplicate. For glucose isotopic labeling, the HMI-11 medium was supplemented with 30 mm ^13^C-labeled glucose. At the indicated time points (0, 12, 21, and 29 h), cultures in logarithmic growth phase were quenched by rapid cooling to 10 °C in a dry ice/ethanol bath, and 10^8^ cells/sample were harvested by centrifugation (2000 × *g*, 10 min, 4 °C). Supernatant samples were collected mixed 1:20 with extraction solvent (chloroform/methanol/water, 1:3:1). Cells were resuspended in 1 ml of PBS, transferred to an Eppendorf tube, and washed once with PBS (2000 × *g*, 10 min, 4 °C). The pellet was resuspended in 200 μl of extraction solvent, crushed with a pipette tip, and extracted for 1 h at 4 °C under shaking (1300 rpm). After the removal of cell debris by centrifugation (14,000 × *g*, 10 min, 4 °C), the extract was stored at −80 °C under argon until LC-MS analysis. To assess instrument performance, one pooled quality control sample was prepared by mixing an equal volume of all of the samples.

### LC-MS

Samples and a mixture of 240 standards (in three mixes) were separated by HPLC on a Dionex Ultimate 3000 RSLC system (Thermo Fisher Scientific) using a ZIC-pHILIC (Merck) column and then analyzed on a Q Exactive Orbitrap mass spectrometer (Thermo Fisher Scientific) using the method of Rattigan *et al.* (93), including fragmentation of the pooled samples.

### Data analysis

The standard mixes' raw files where analyzed using ToxID Automated Compound Screening software (Thermo Fisher Scientific). Raw sample data files were converted to mzXML and fragmentation files to mzML using Proteowizard (94). The files were then uploaded into the online metabolomics platform PiMP (95). Data were quality-controlled using the principle component analysis (PCA) and total ion chromatograms generated by PiMP. The metabolites identified by PiMP were filtered to retain only metabolites with retention times matching standards (identified) and/or identified using fragmentation by PiMP's algorithm FrAnK. Double peaks were also excluded. 84 metabolites were then identified for the glucose-labeled experiment and 65 for the time course experiment.

The ^13^C-labeled glucose experiment data set was analyzed using mzmatch-ISO (96). The unlabeled samples were analyzed to find significantly changed metabolites using PiMP (*t* test on log-transformed data, Benjamini and Hochberg correction). The time course data set was then analyzed by implementing a linear model using R, followed by analysis of variance to identify metabolites with differential time-dependent changes between treated and untreated cells (model = lm(*y* ∼ IsTreated*Time), where *y* is the metabolite intensity). The 65 *p* values were then corrected using the Benjamini–Hochberg procedure (false discovery rate <0.05). Data for the 48 significantly changed metabolites where then uploaded to Metaboanalyst (5) to generate a normalized heat map.

## Data availability

All data (except raw 'omics data) are contained within the article. 'Omics data sets for the proteomics and metabolomics analyses are available upon request from the corresponding author. Proteomics data have been deposited to the ProteomeXchange Consortium via the PRIDE partner repository (92) with the data set identifier PXD014215.

## Supplementary Material

Supporting Information
