# Supplementary material for: Suramin exposure alters cellular metabolism and mitochondrial energy production in African trypanosomes
Source: J Biol Chem. 2020 Apr 30;295(24):8331–47. doi: 10.1074/jbc.RA120.012355 (PMC7294092; doi:10.1074/jbc.RA120.012355)
Supplement: Supporting Information [file supp_295_24_8331__index.html]

Suramin exposure alters cellular metabolism and mitochondrial energy production in African trypanosomes — Suramin mode of action — Supporting Information 

# Suramin exposure alters cellular metabolism and mitochondrial energy production in African trypanosomes

## Supporting Information

- Movie S1 - 3Dreconstruction of glycosomes
- Table S3 - Metabolomics dataset
- Table S2 - Dataset
- Table S4 - Dataset
- Table S1 - Dataset
- Supporting Information (to be published online) -

  Supporting figures
