# Supplementary material for: Suramin exposure alters cellular metabolism and mitochondrial energy production in African trypanosomes
Source: J Biol Chem. 2020 Apr 30;295(24):8331–47. doi: 10.1074/jbc.RA120.012355 (PMC7294092; doi:10.1074/jbc.RA120.012355)
Supplement: Supporting Information [file supp_RA120.012355_157778_3_supp_514944_q94r7g.pdf]

Supplementary material for:

Suramin induces mitochondrial activation in bloodstage African trypanosomes

Martin Zoltner[1$], Gustavo Campagnaro[2], Gergana Taleva[3], Alana Burrell[4], Michela Cerone[2], Ka-Fai Leung[5], Fiona Achcar[6], David Horn[1], Sue Vaughan[4], Catarina Gadelha[7], Alena Zíková[3], Michael P. Barrett[6], Harry P. de Koning[2] and Mark C. Field[1*]

Supplementary figure legends

Figure S1: Summary of metabolite changes of cells grown in the presence ^{13}C-labeled glucose one day after suramin exposure.

Figure S2: Amino acid level changes upon suramin treatment. -log10 fold changes of amino acids detected in the metabolomics are plotted versus the 3 time points analyzed.

Figure S3: Suramin induced proteome changes have an onset later than 24 hours. Correlation of proteome changes upon suramin treatment after 24 hours and 48 hours. The correlation plot uses the respective normalised SILAC ratio, averaged from triplicate experiments.

Figure S4: GO term analysis. Volcano plots of normalised SILAC ratio after 48 hours suramin (35 nM) exposure. -log2 transformed average ratios from triplicate experiments, are plotted against the respective -log10 transformed p-values. Data points are colored for indicated GO terms. Selected data points are annotated.

Figure S5: Correlation of proteome changes upon suramin treatment with proteome differences between BSF and PCF. -log2 transformed abundance shifts after 48 hours suramin (35 nM) exposure, were plotted against corresponding -log2 transformed abundance differences between BSF and PCF (50). Selected GO terms are color indicated. Infinite changes were omitted for clarity. (DH = dehydrogenase, PPDK = pyruvate phosphate dikinase).

Figure S6: Suramin binding to serum proteins. Gel filtration chromatogram of 0.5 ml foetal bovine serum pre-incubated with ^{3}H-suramin on a 24 ml Superdex 200 column. The black line indicates the relative absorbance at λ=280 nm and the red line the scintillation count (counts per minute (CPM)) of respective fractions.

Figure S7: Pyruvate transporter overexpression has no effect on suramin sensitivity. Pt1-myc overexpression was detected by western blotting using the myc epitope (A), but did not change potency (EC_{50}) of suramin (B).

Movie S1: 3D segmentation of whole *T. brucei* cell. Plasma membrane and flagellum (purple), glycosomes (orange).

Table S1: High confidence metabolite changes of cells grown in the presence ^{13}C-labeled glucose one day after suramin exposure List of 84 quantified metabolites with the respective log_2 fold change and corresponding *p*-value

Table S2: Metabolomics analysis at different time points of suramin exposure.

Table S3: High confidence metabolite changes at different time points of suramin exposure List of 65 quantified metabolites.

Table S4: SILAC proteomics analysis after 24 hours and 48 hours suramin exposure.

(R)−Lactate

Formula: C3H6O3 Mass: 90.032 Std.RT: 552.6 Ion: NEG

G1

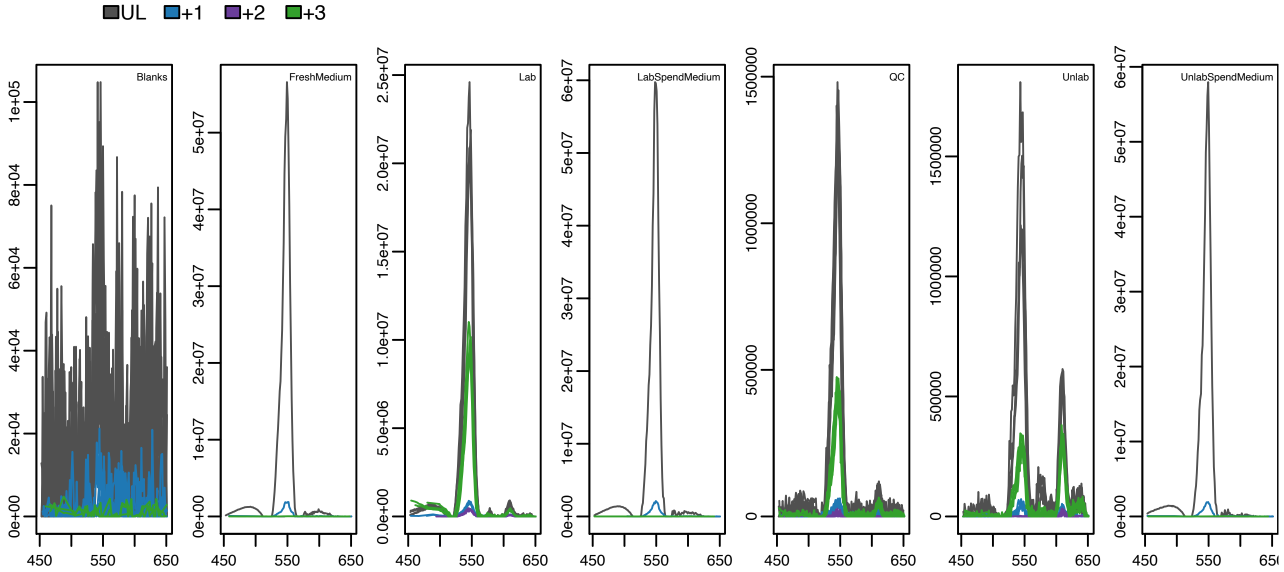

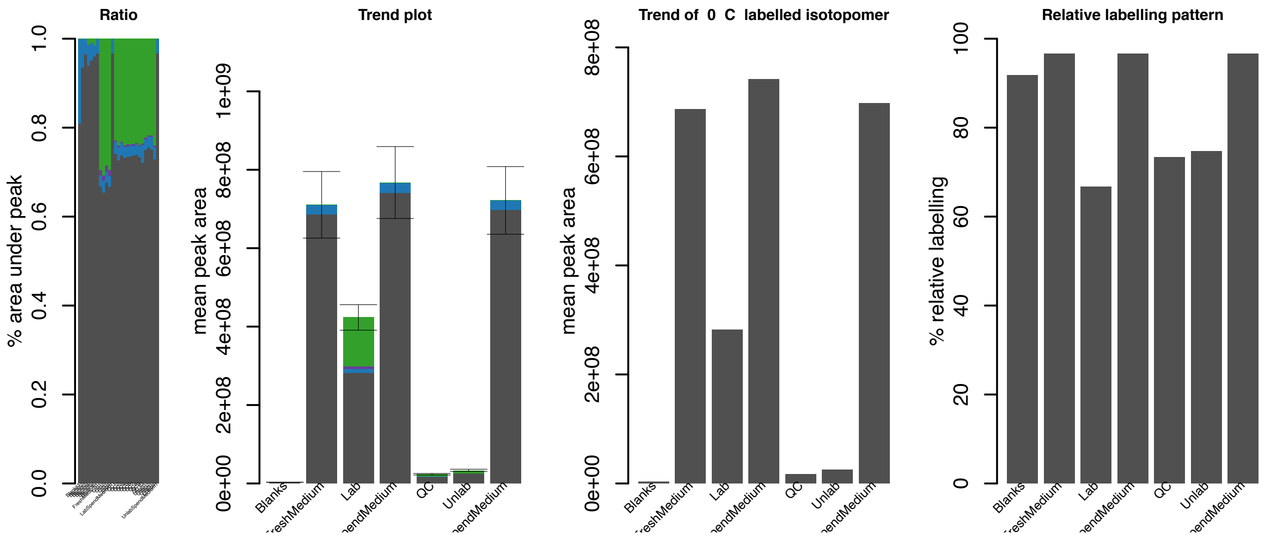

(S)−Malate

Formula: C4H6O5 Mass: 134.022 Std.RT: 777 Ion: NEG

G1

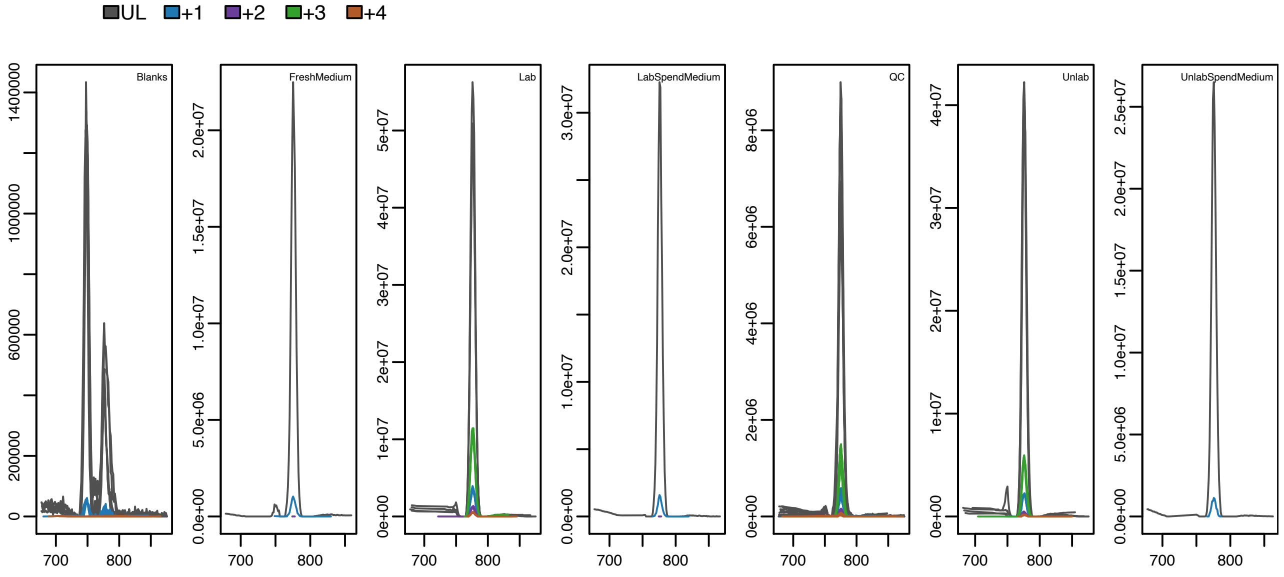

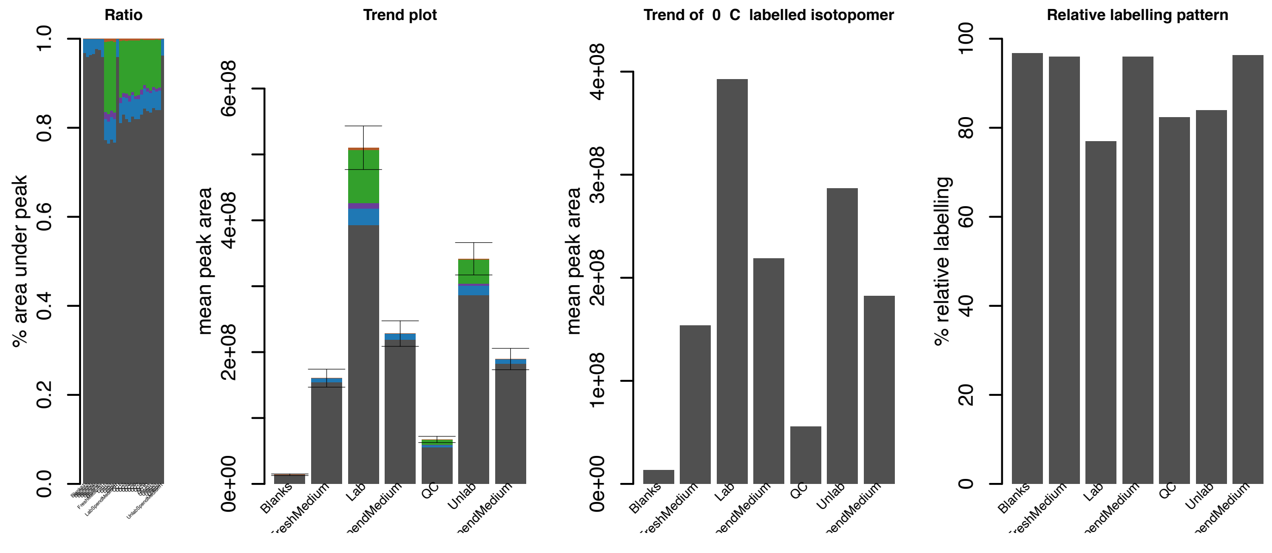

2−phospho−D−glycerate

Formula: C3H7O7P Mass: 185.993 Std.RT: 783 Ion: NEG

G1

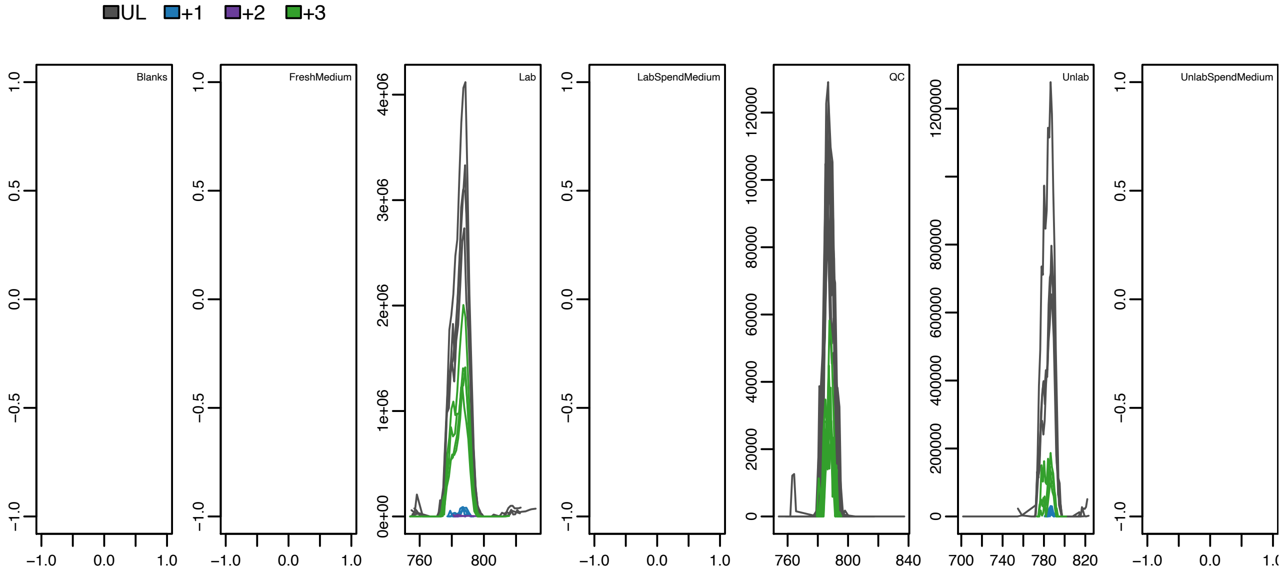

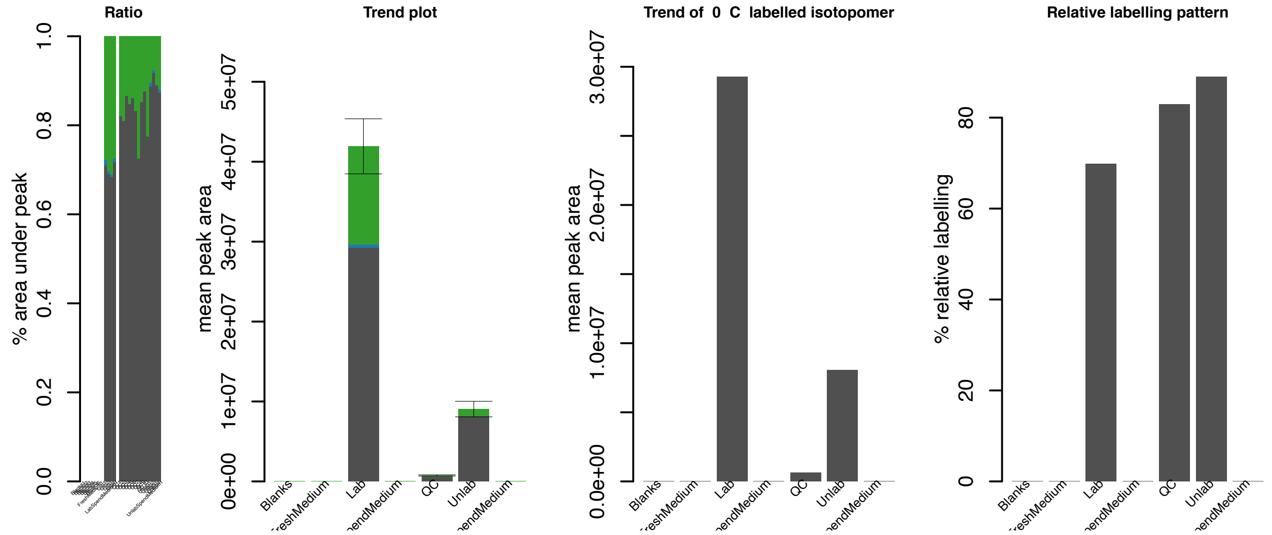

3'_5'–Cyclic AMP

Formula: C10H12N5O6P Mass: 329.053 Std.RT: 526.8 Ion: NEG

G1

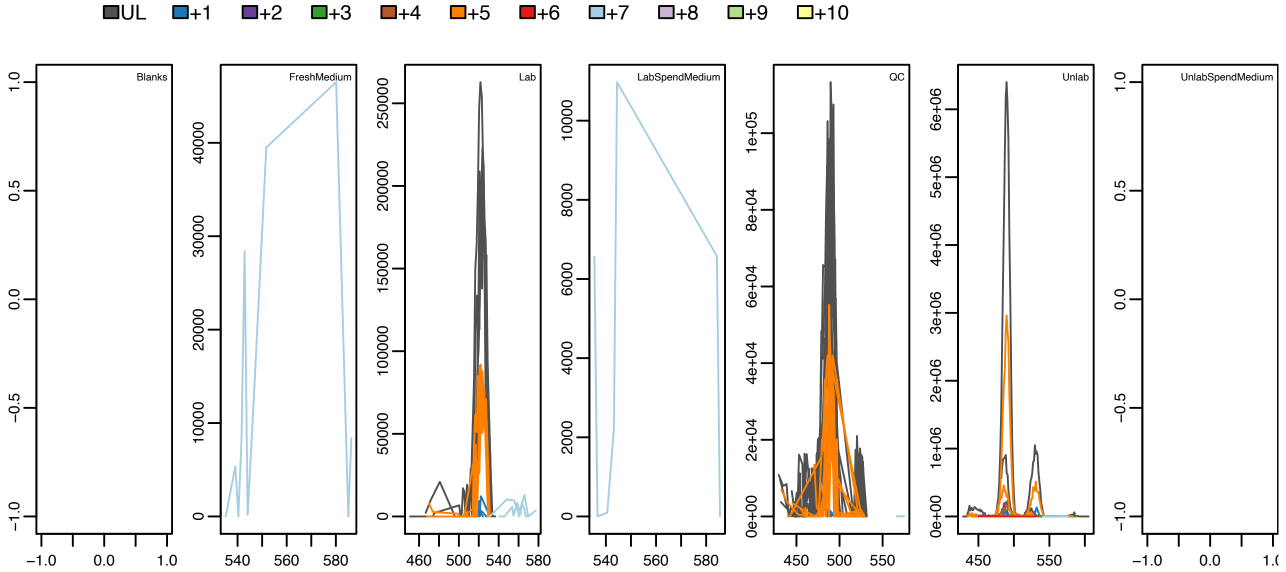

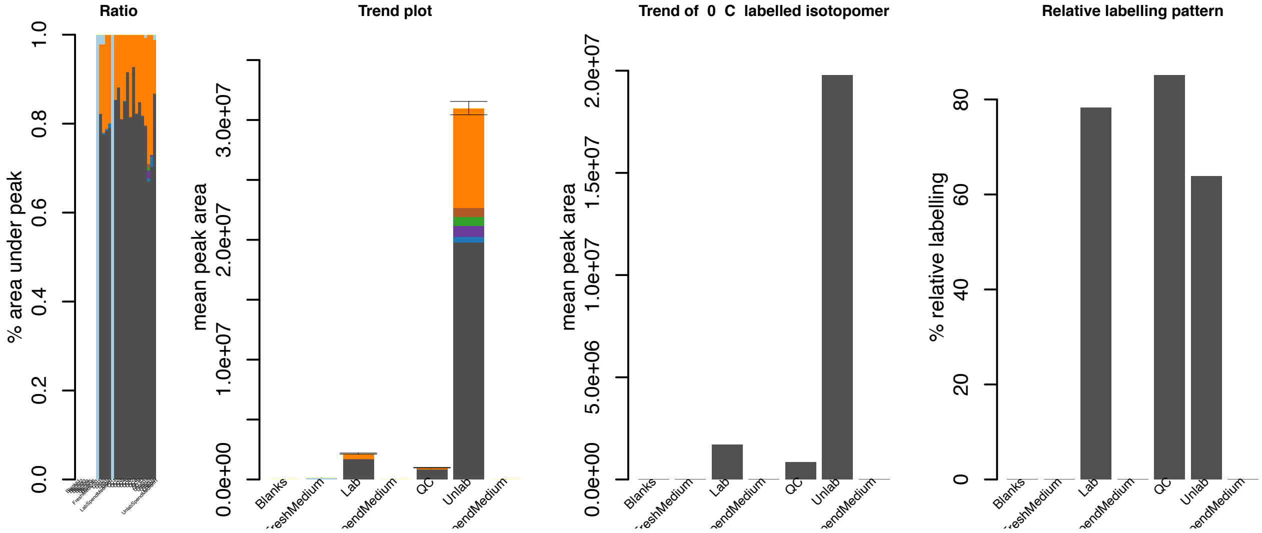

3−Phospho−D−glycerate

Formula: C3H7O7P Mass: 185.993 Std.RT: 788.4 Ion: NEG

G1

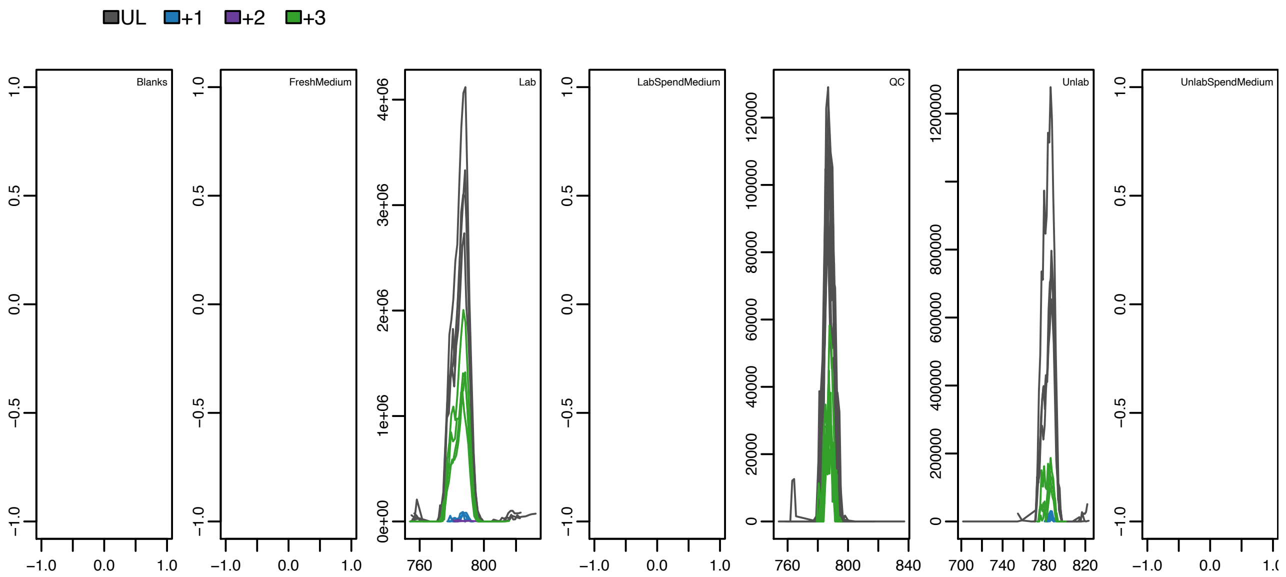

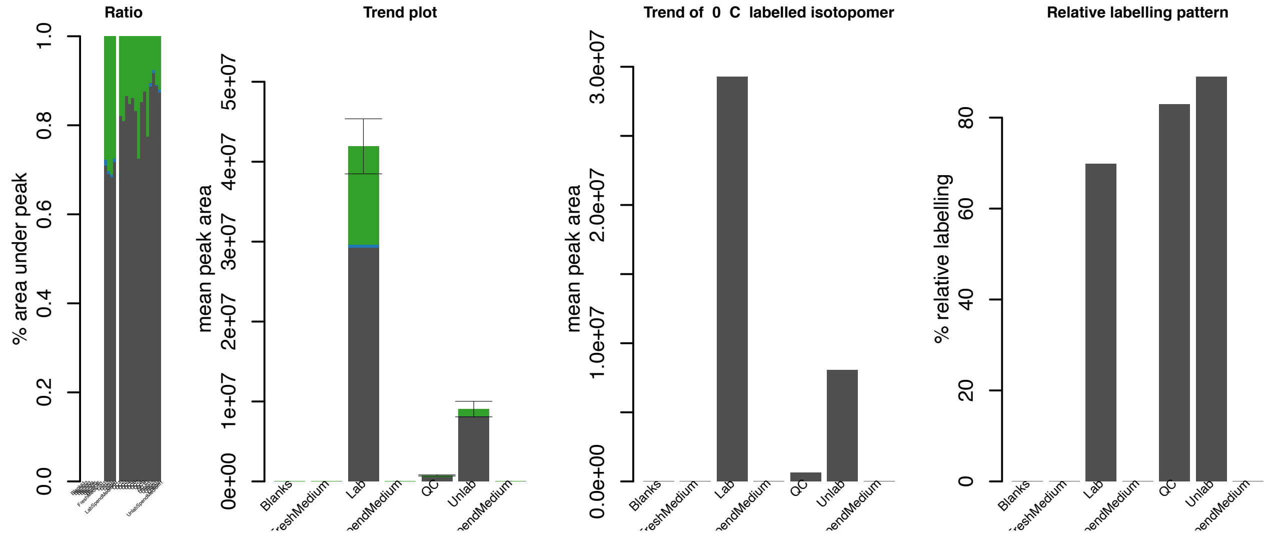

ADP

Formula: C10H15N5O10P2 Mass: 427.029 Std.RT: 714 Ion: NEG

G1

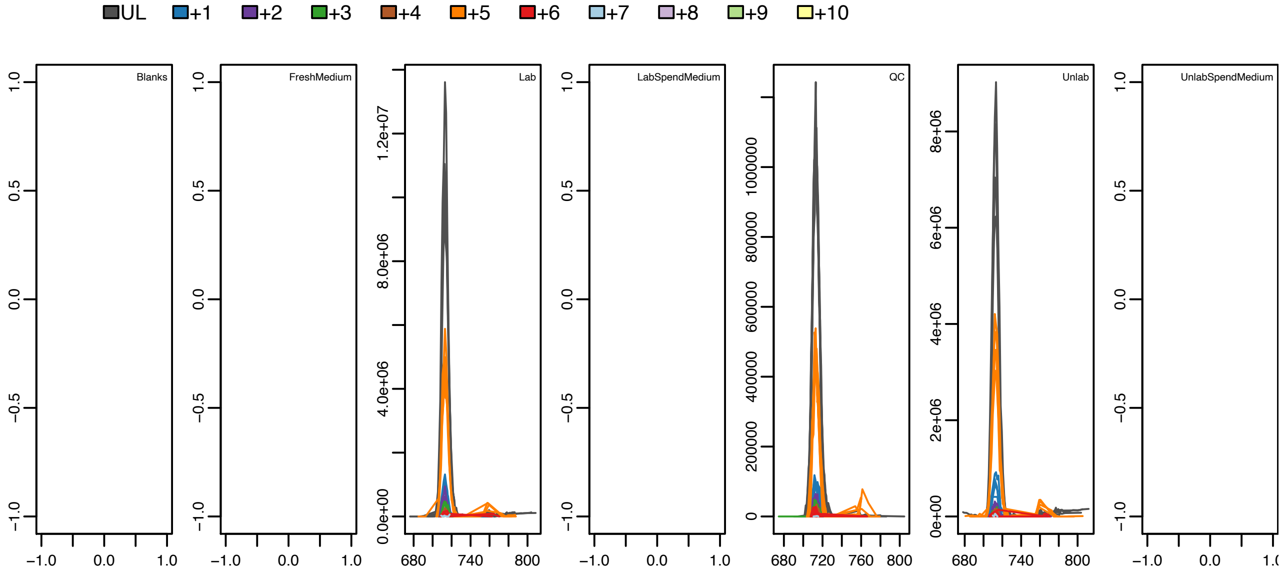

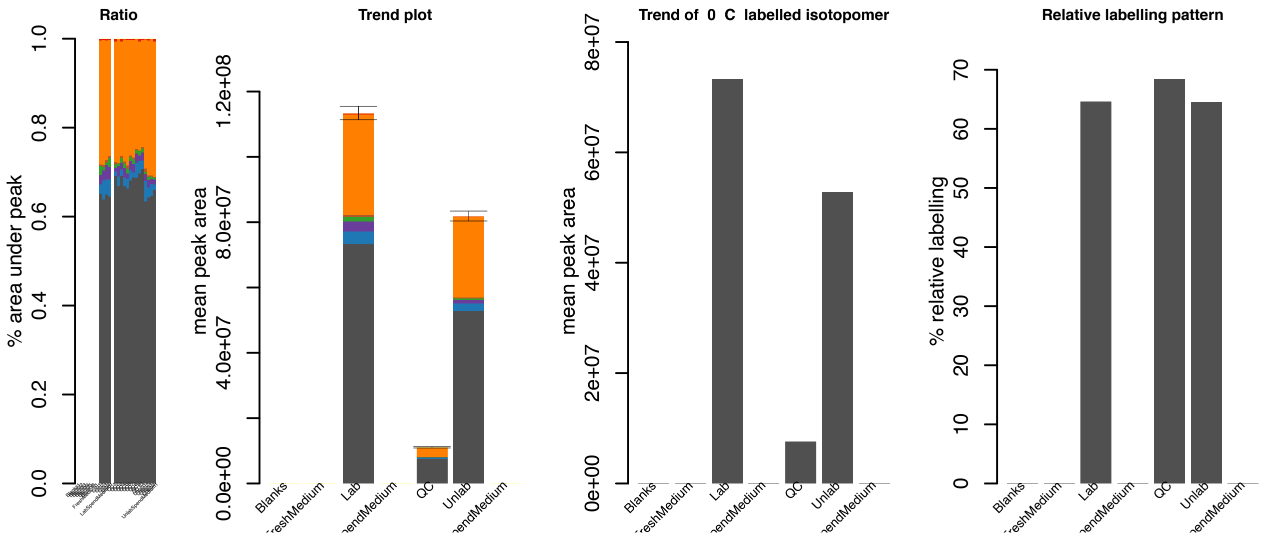

AMP

Formula: C10H14N5O7P Mass: 347.063 Std.RT: 670.8 Ion: NEG

G1

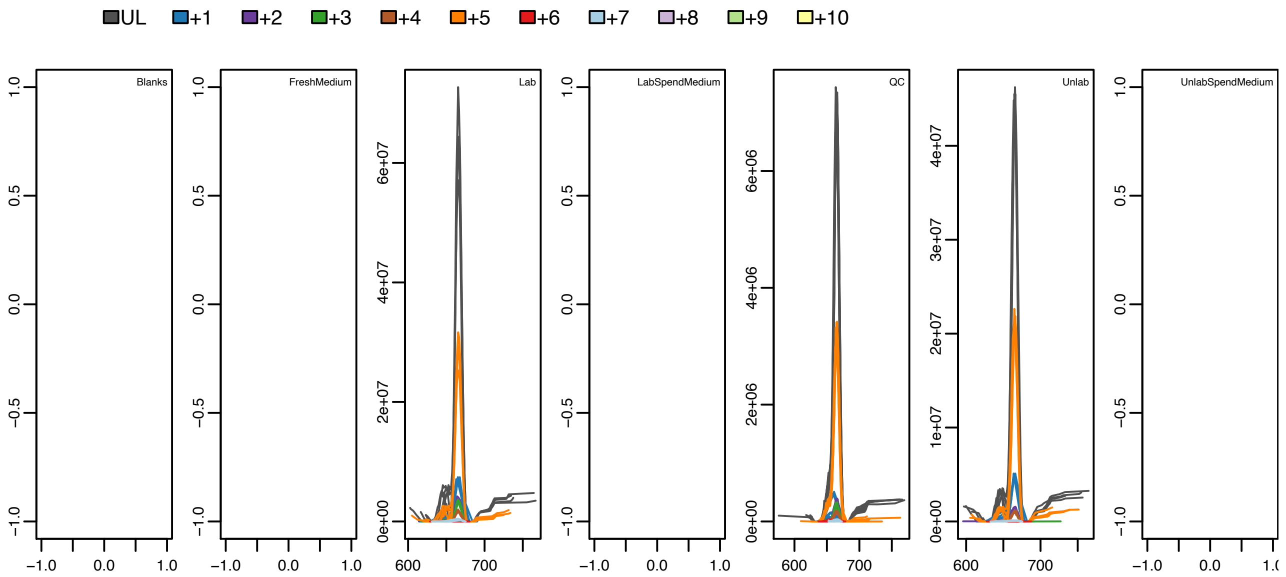

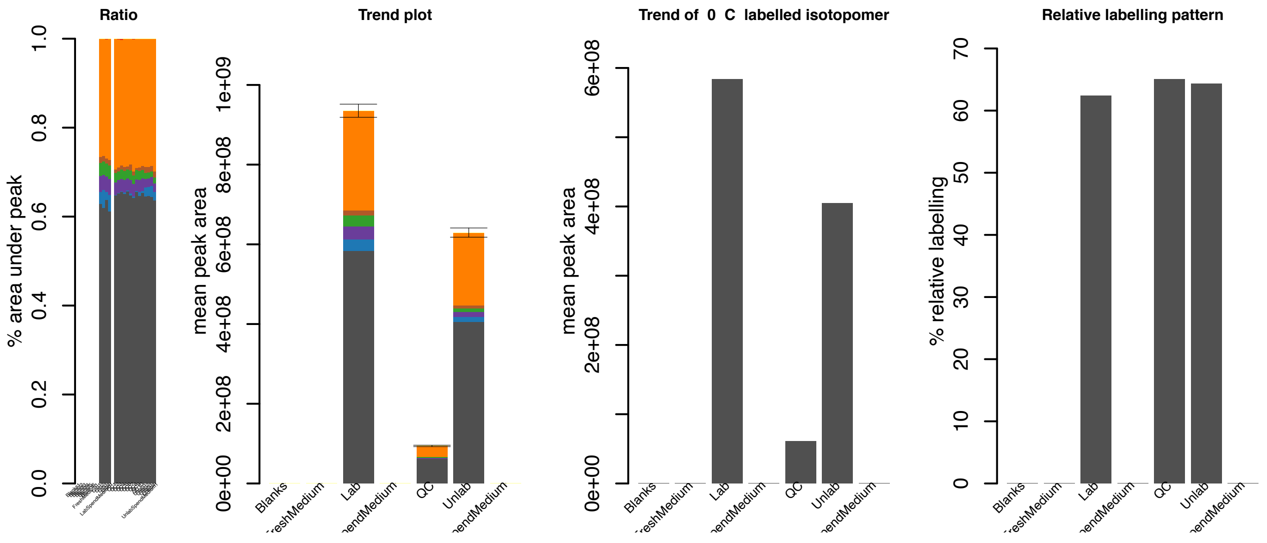

Adenine

G1

Formula: C5H5N5 Mass: 135.054 Std.RT: 523.8 Ion: NEG

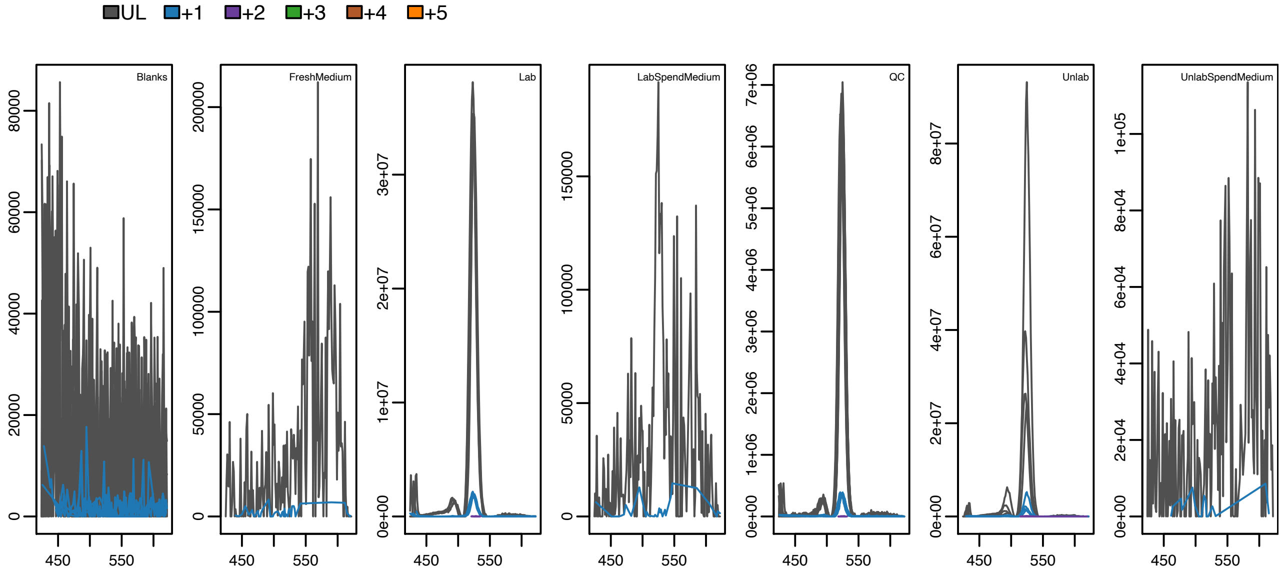

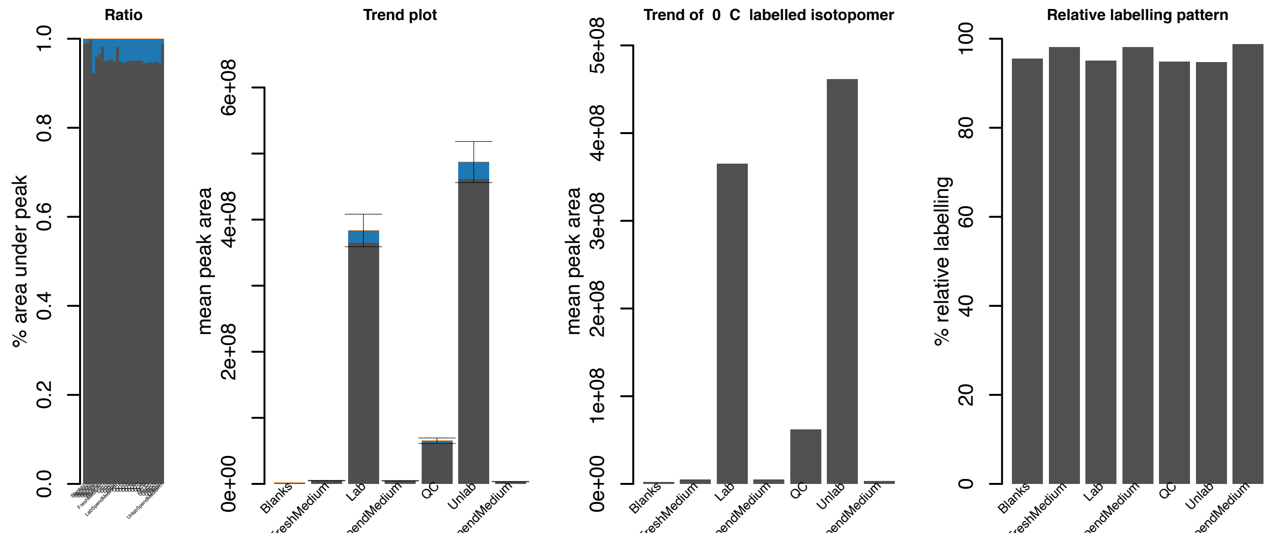

Adenosine

Formula: C10H13N5O4 Mass: 267.097 Std.RT: 494.4 Ion: NEG

G1

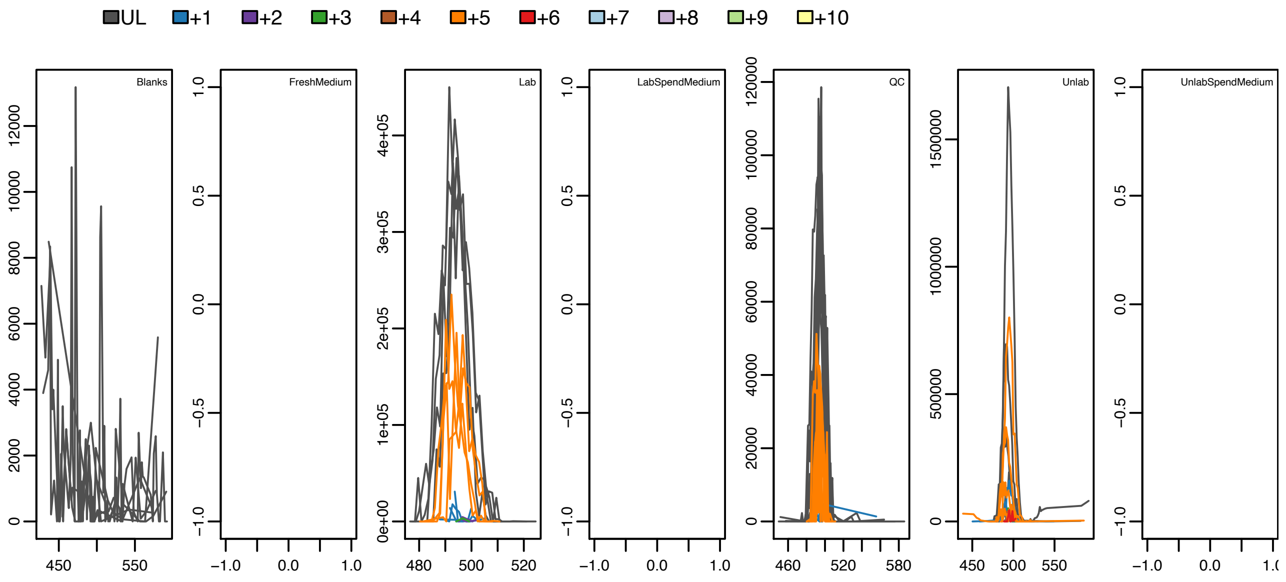

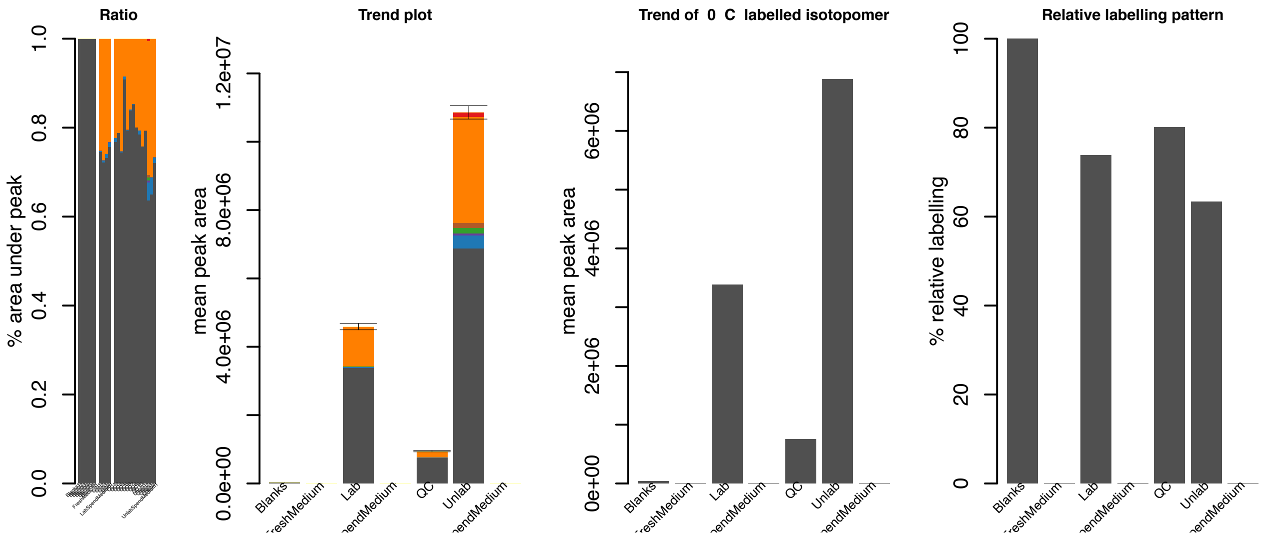

Adenosine 2'_5'–bisphosphate

Formula: C10H15N5O10P2 Mass: 427.029 Std.RT: 761.4 Ion: NEG

G1

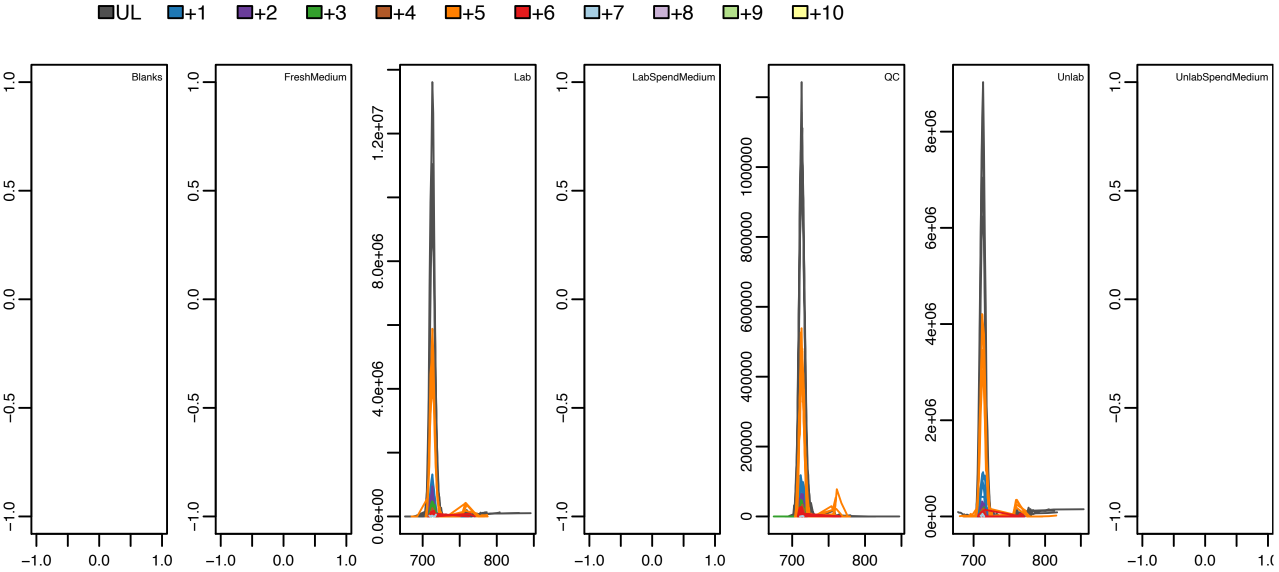

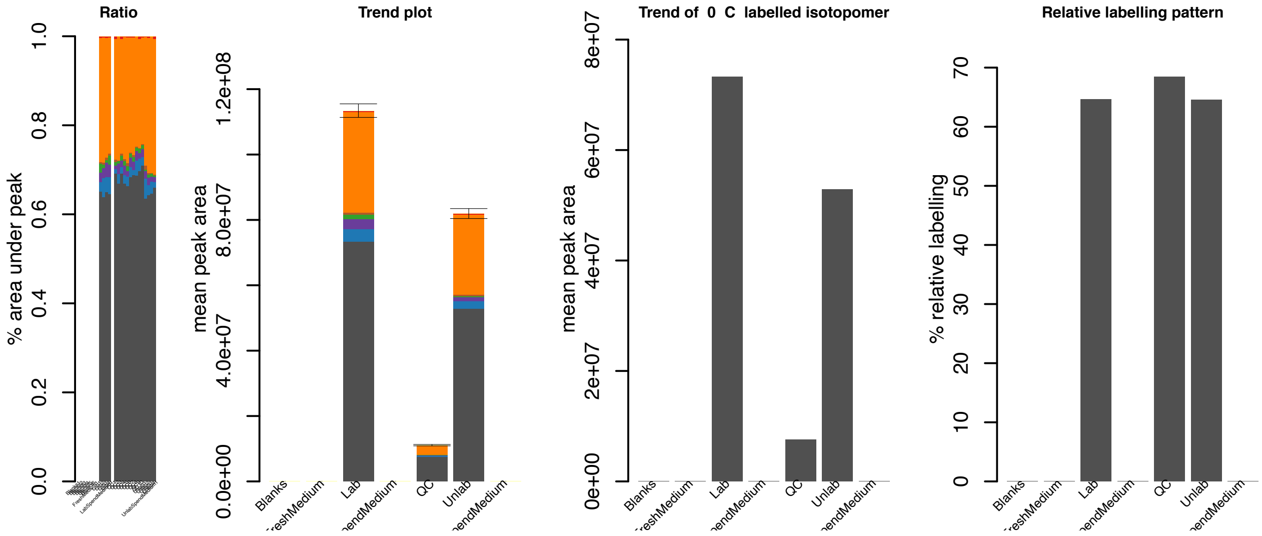

D−Fructose 6−phosphate

Formula: C6H13O9P Mass: 260.03 Std.RT: 754.8 Ion: NEG

G1

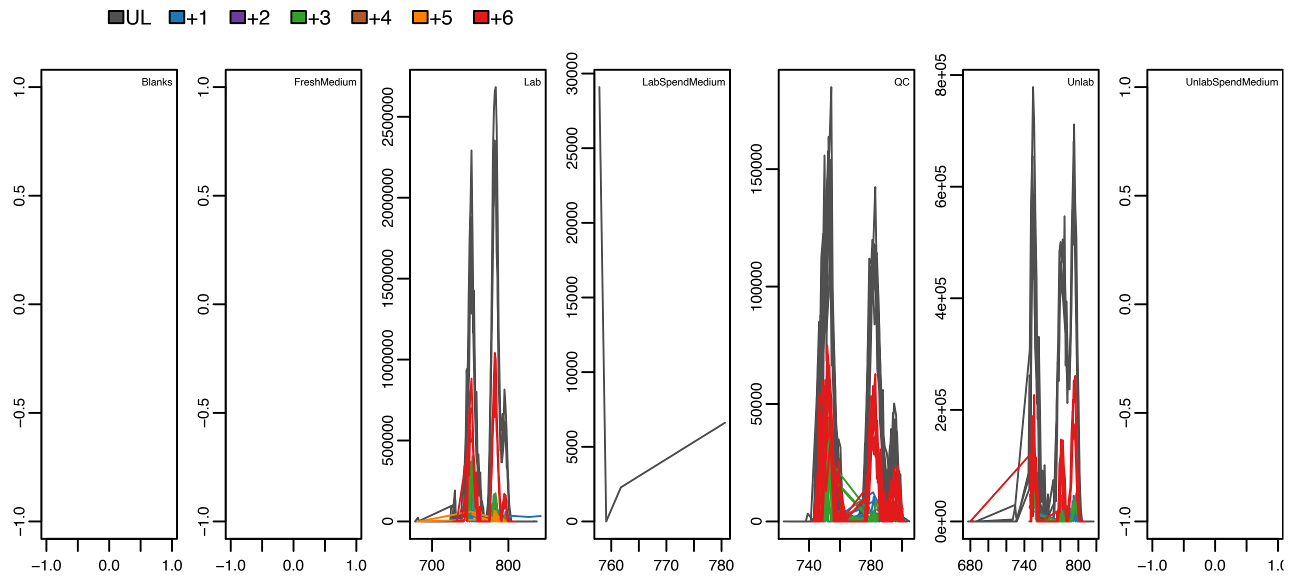

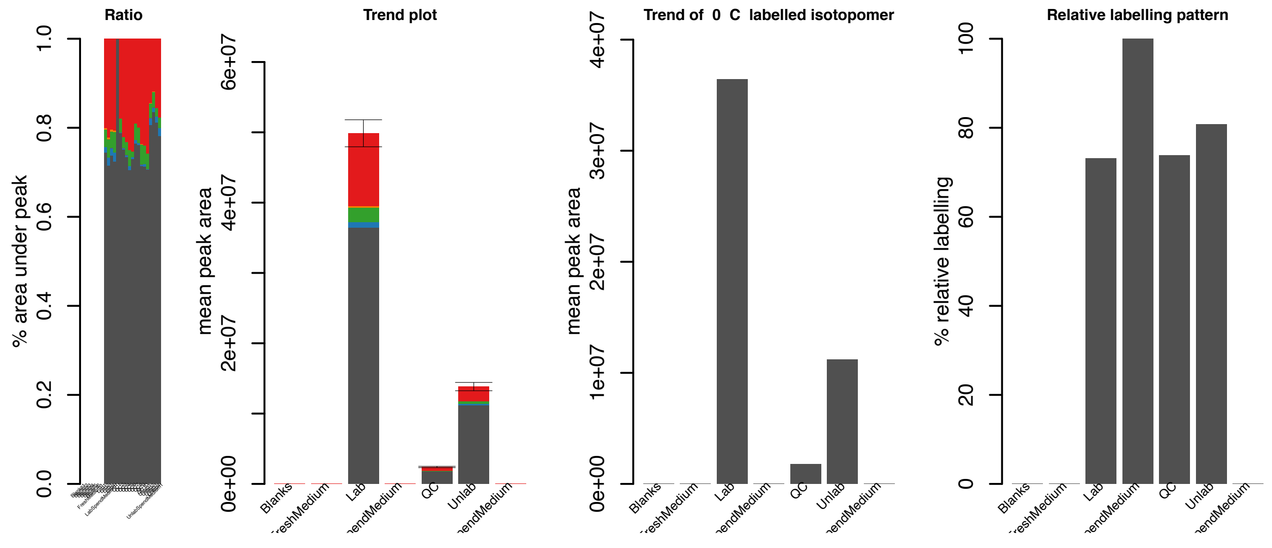

D−Gluconic acid

Formula: $C6H12O7$ Mass: 196.058 Std.RT: 712.8 Ion: NEG

G1

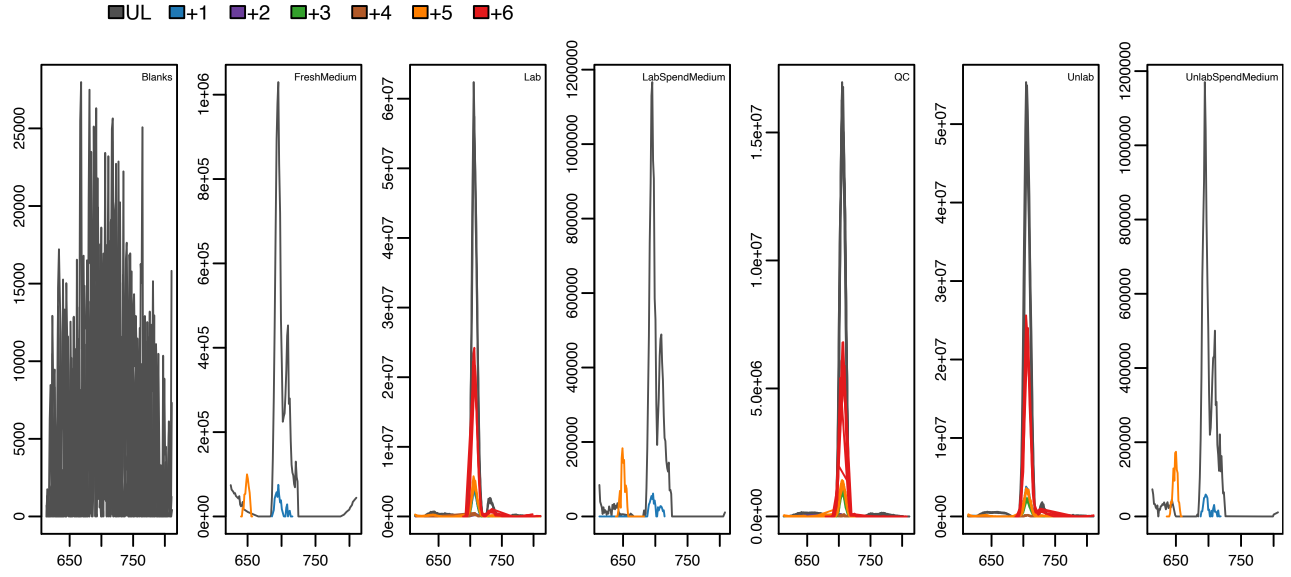

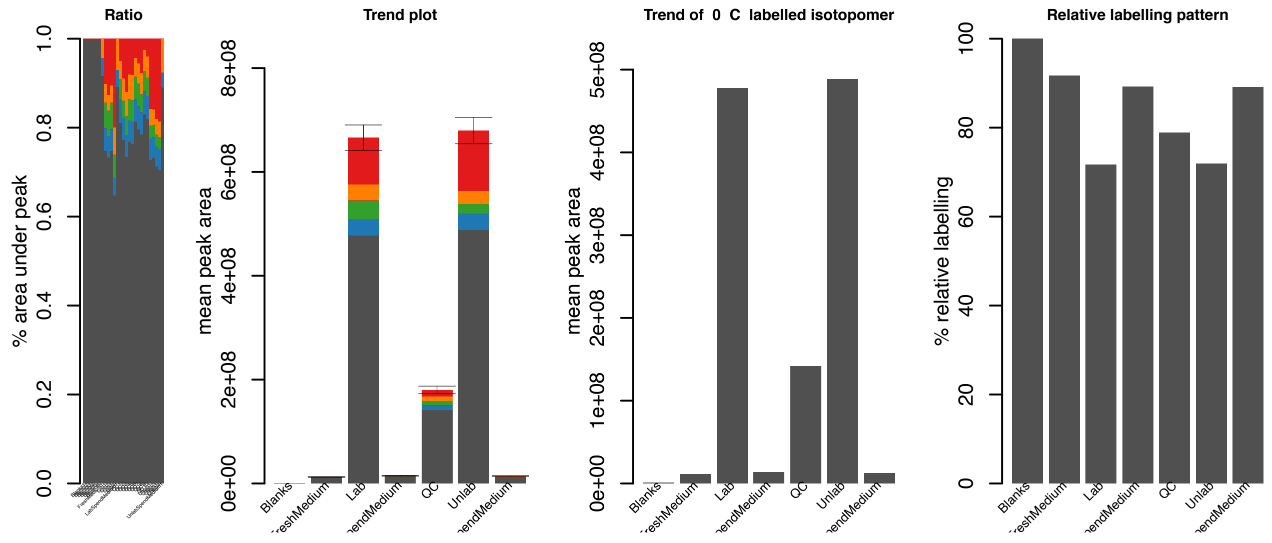

D−Glucosamine−6−phosphate

Formula: C6H14NO8P Mass: 259.046 Std.RT: 759 Ion: NEG

G1

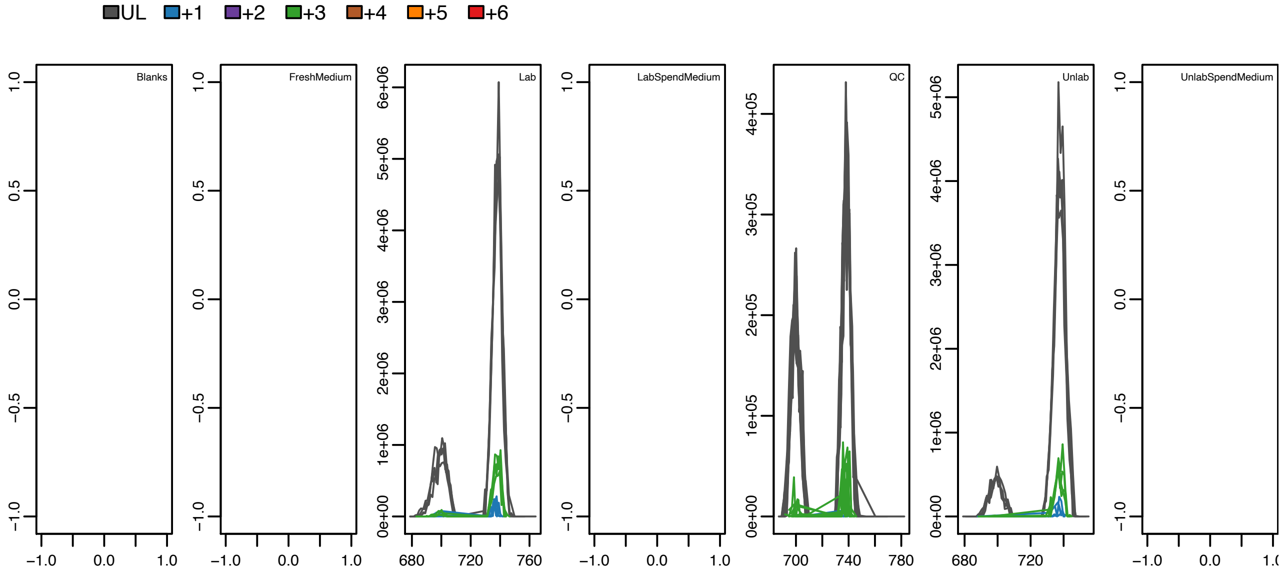

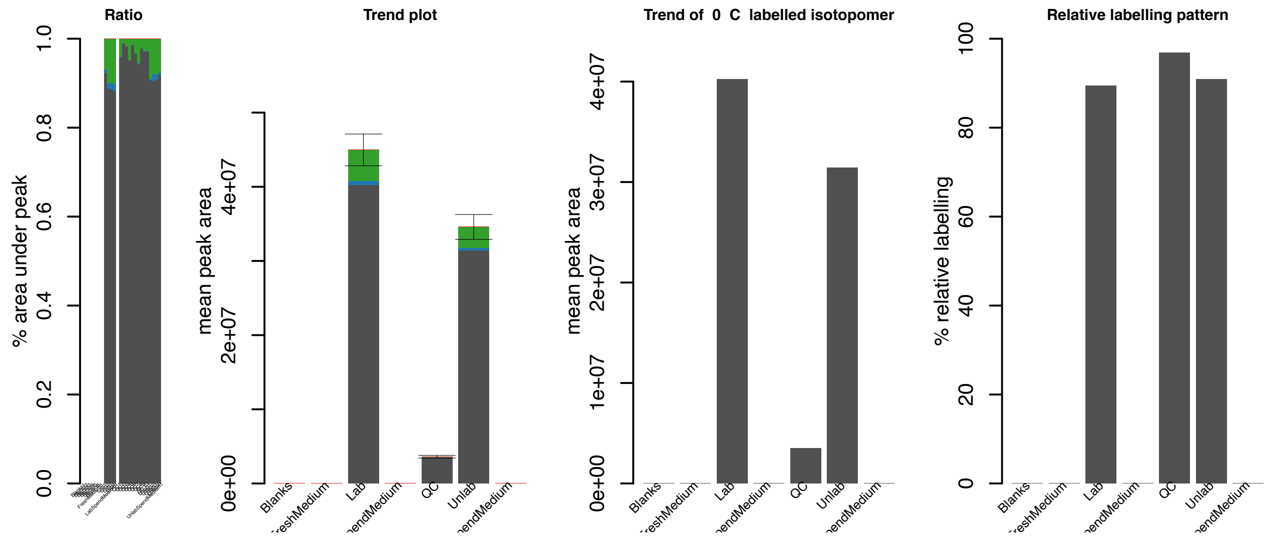

D−glucose 6−phosphate

Formula: C6H13O9P Mass: 260.03 Std.RT: 783 Ion: NEG

G1

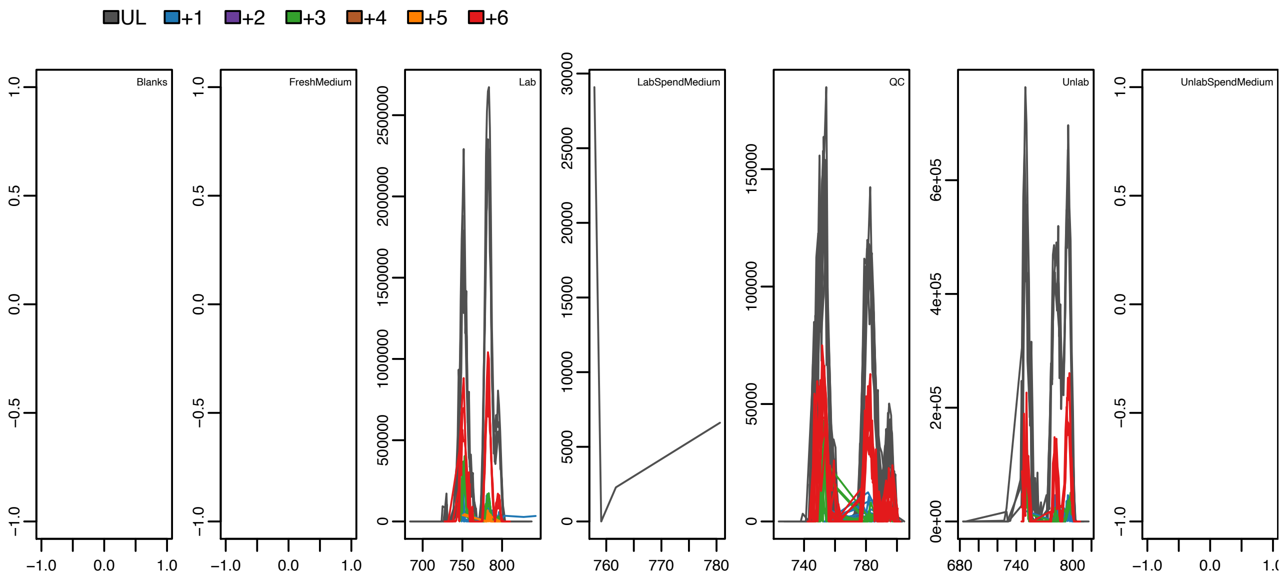

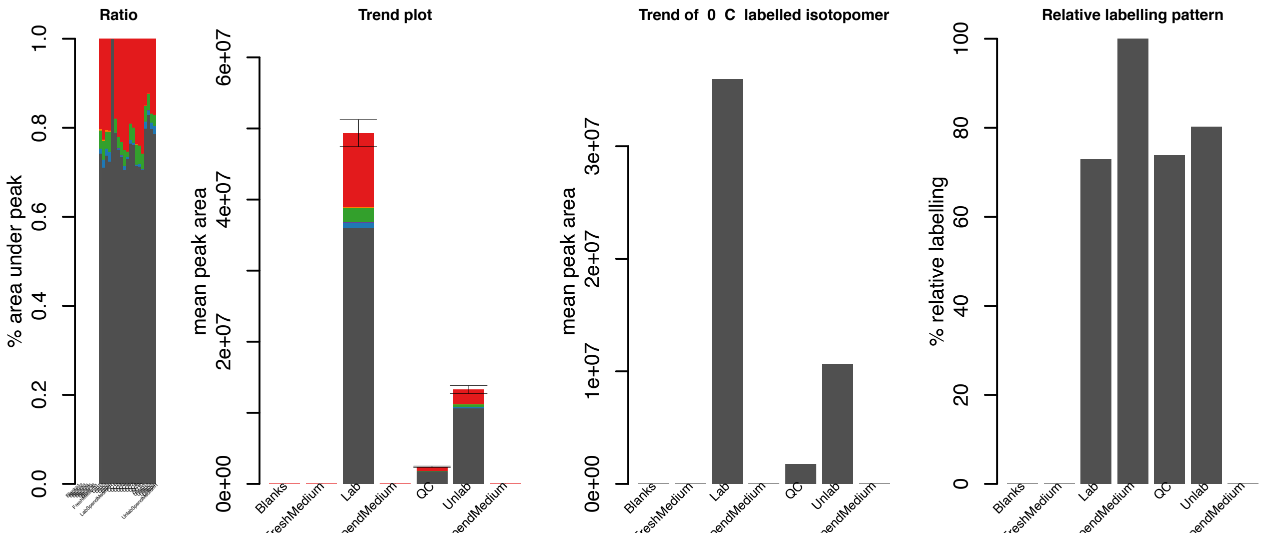

D−ribose 5−phosphate

Formula: C5H11O8P Mass: 230.019 Std.RT: 750 Ion: NEG

G1

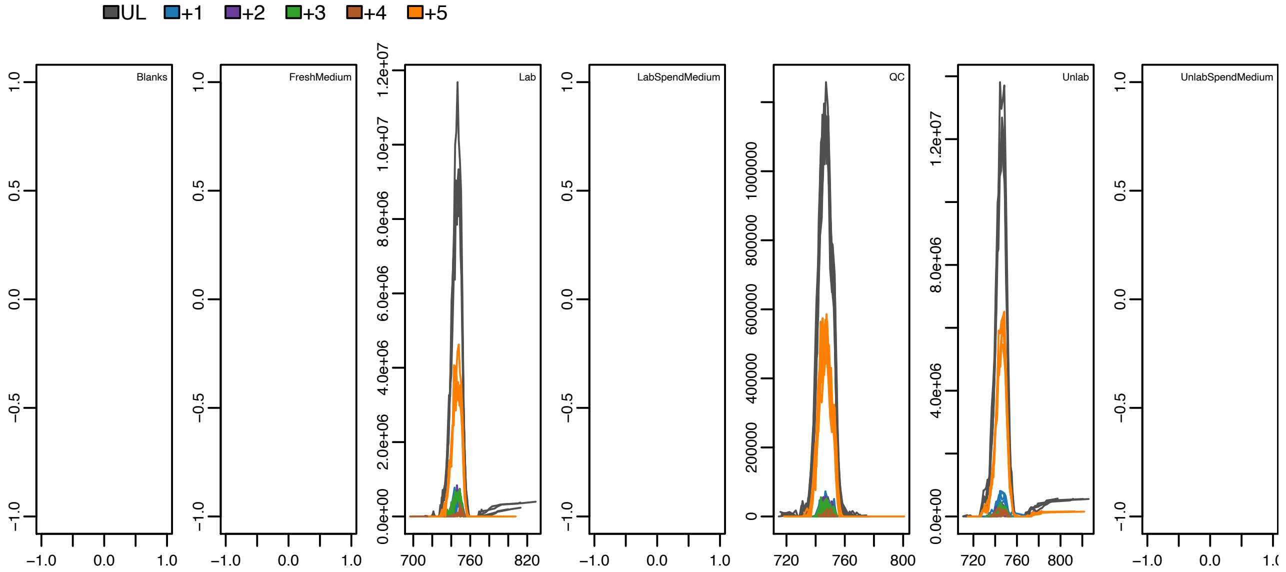

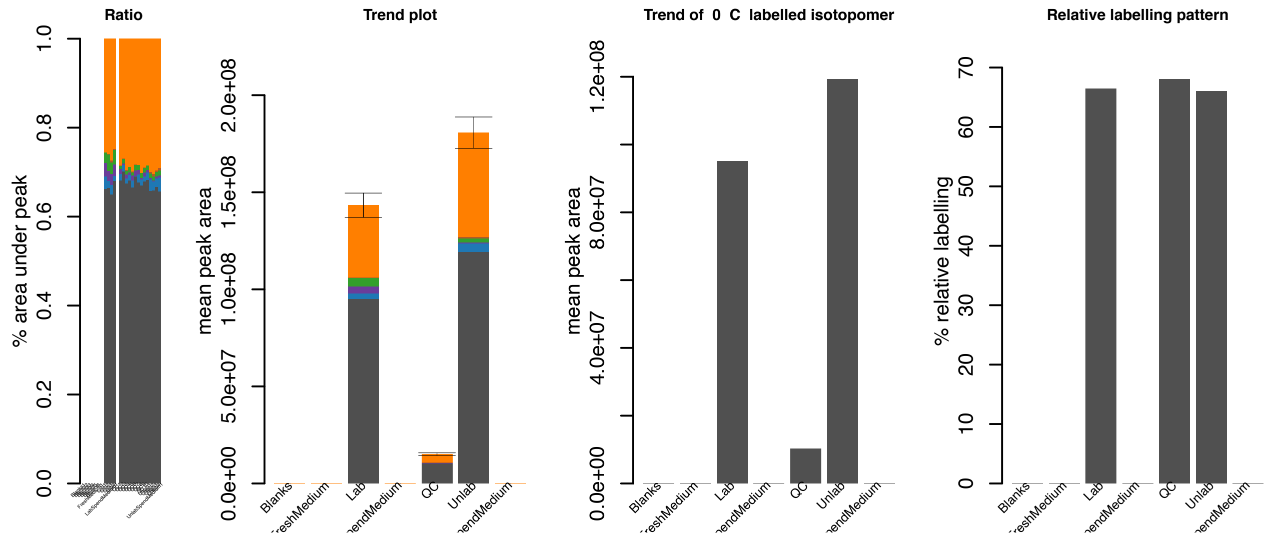

Fumarate

G1

Formula: C4H4O4 Mass: 116.011 Std.RT: 784.2 Ion: NEG

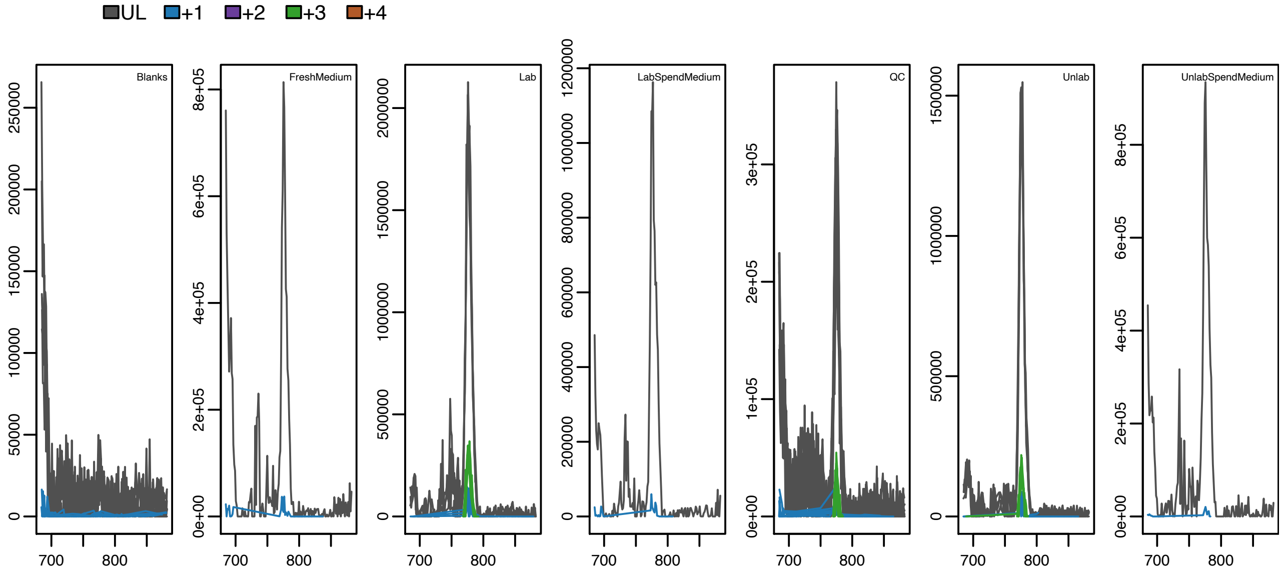

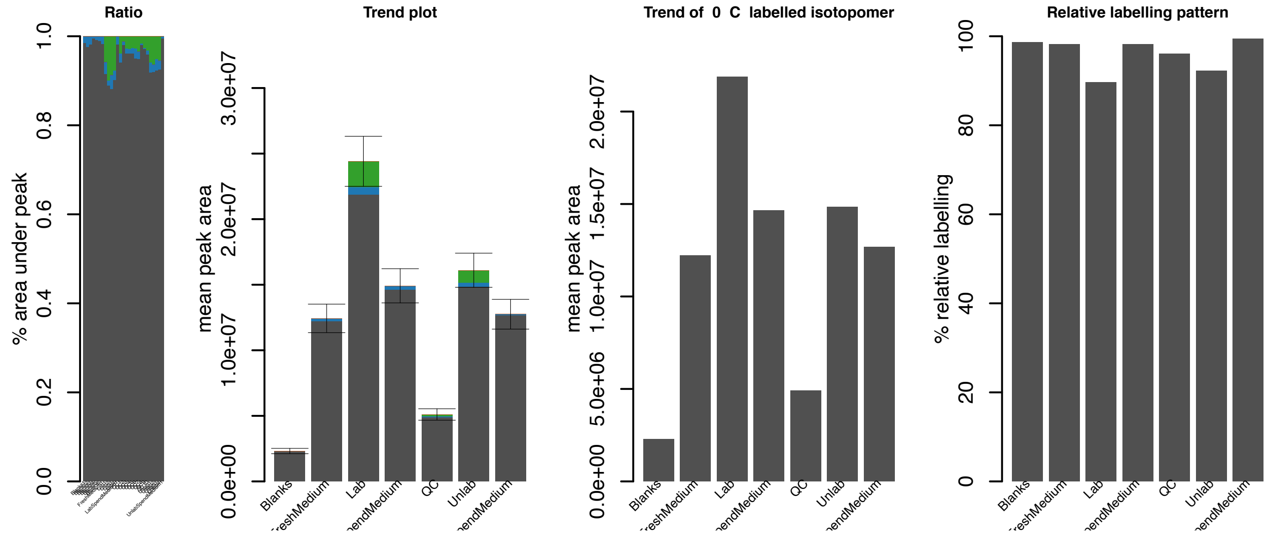

GDP

Formula: C10H15N5O11P2 Mass: 443.024 Std.RT: 816 Ion: NEG

G1

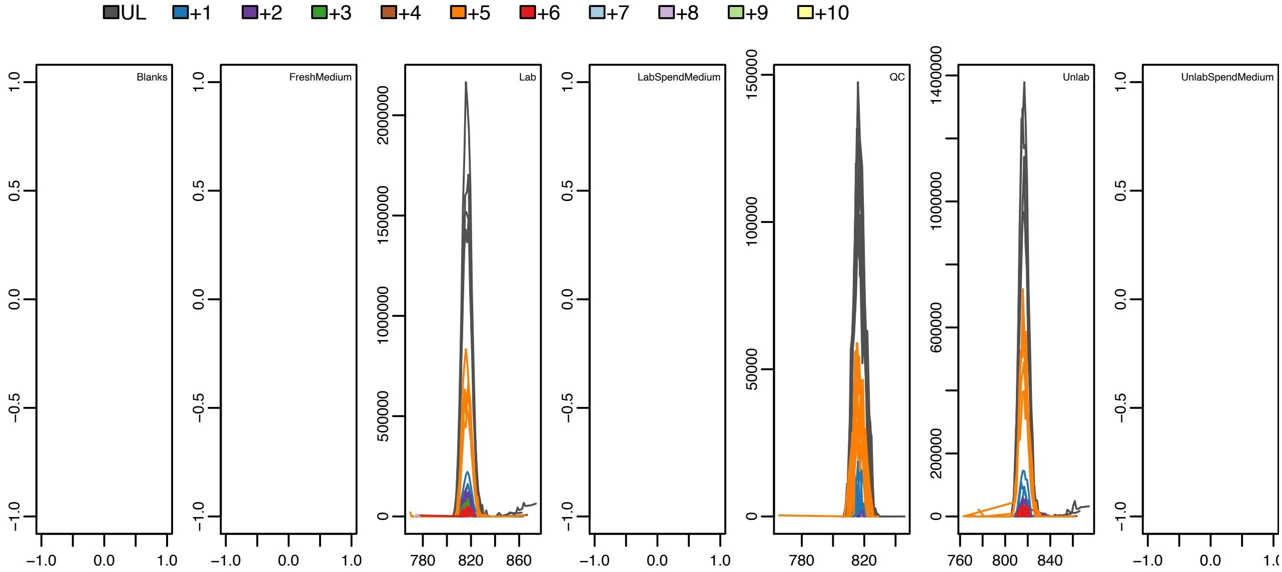

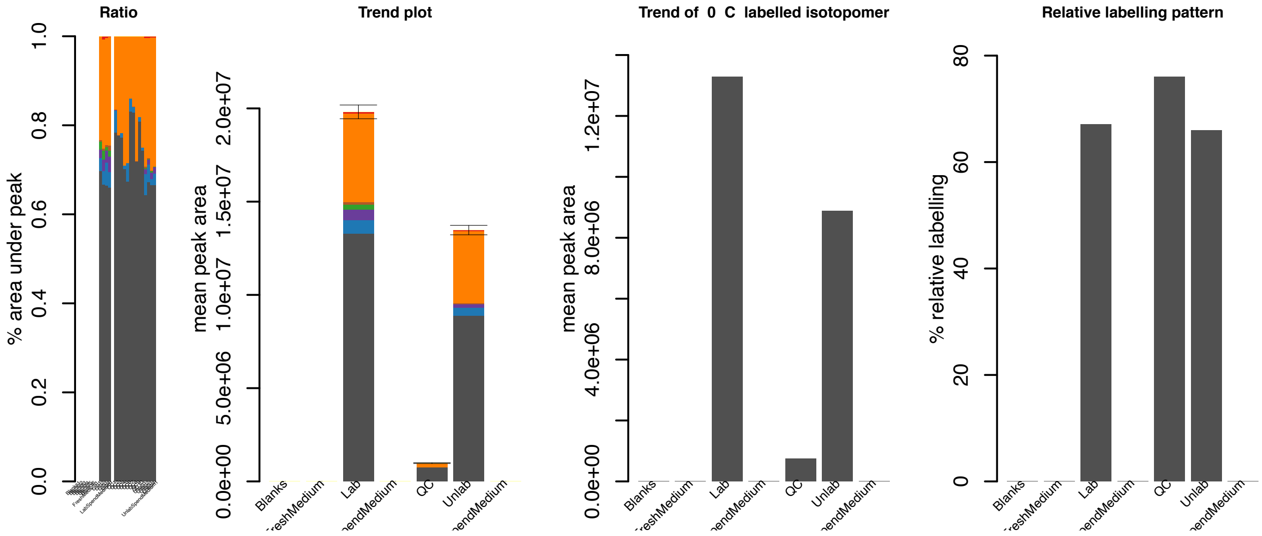

GMP

Formula: C10H14N5O8P Mass: 363.058 Std.RT: 747 Ion: NEG

G1

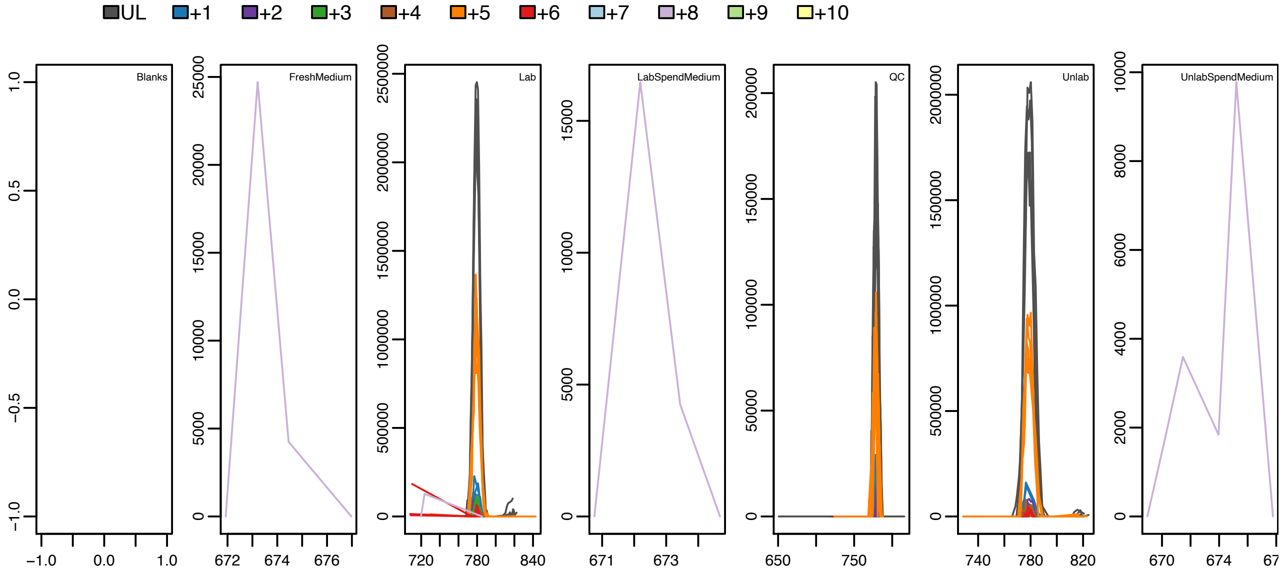

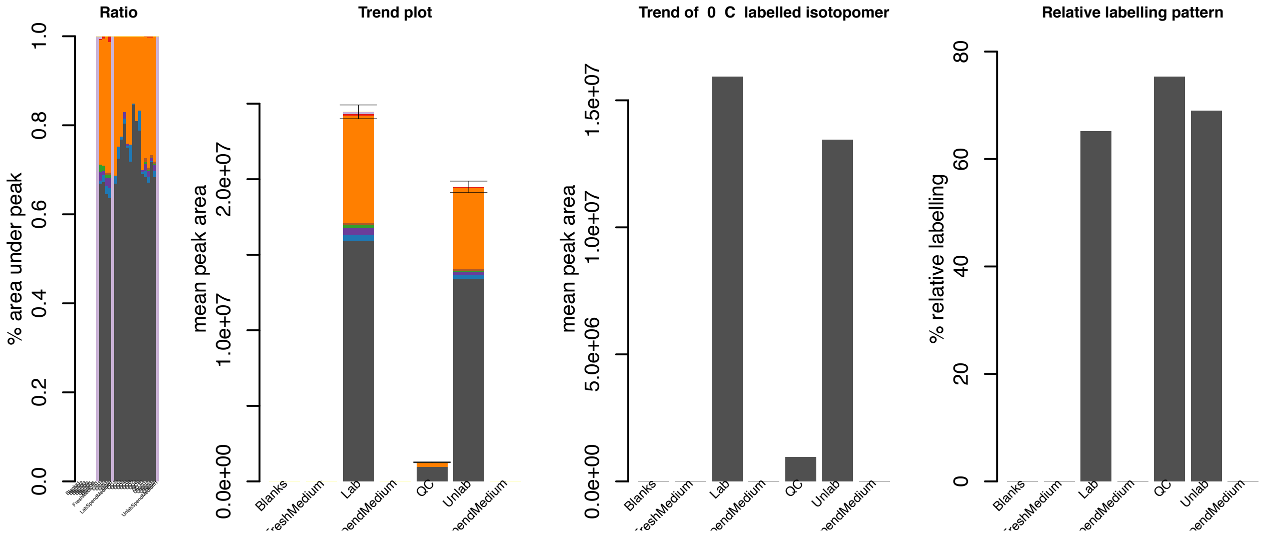

Glycerol

Formula: C3H8O3 Mass: 92.047 Std.RT: 525 Ion: NEG

G1

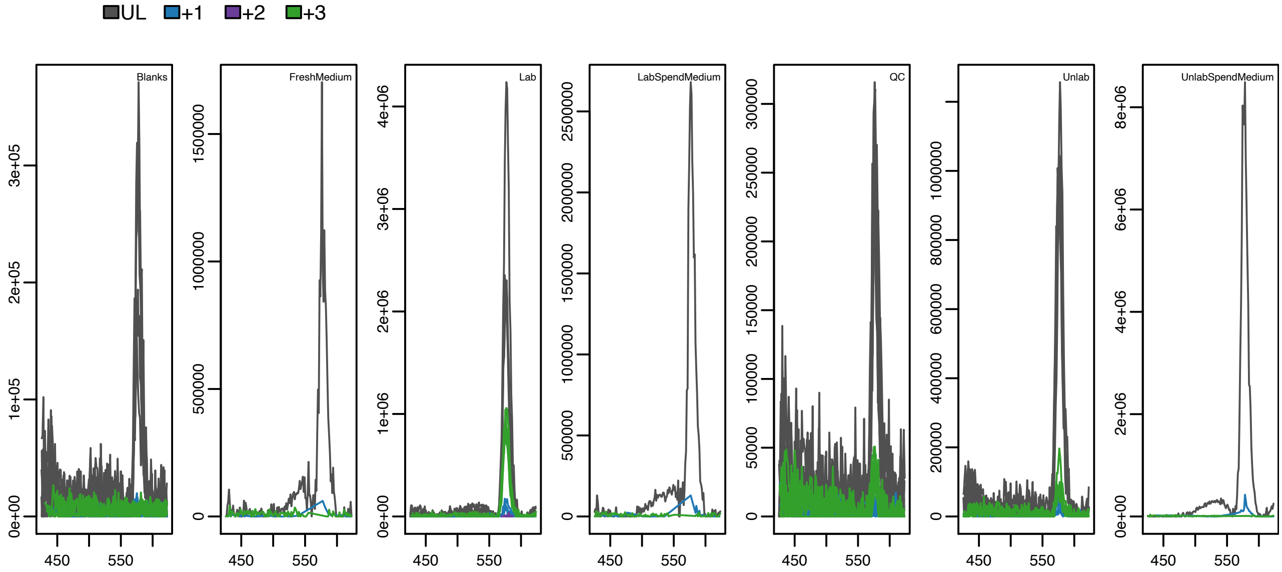

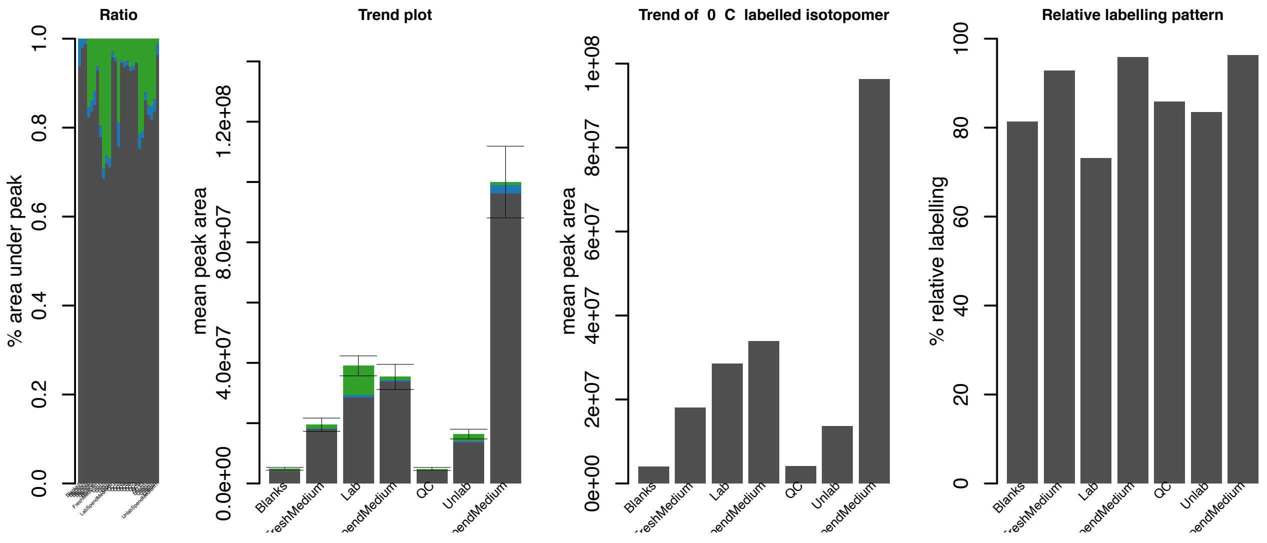

Guanine

Formula: C5H5N5O Mass: 151.049 Std.RT: 664.2 Ion: NEG

G1

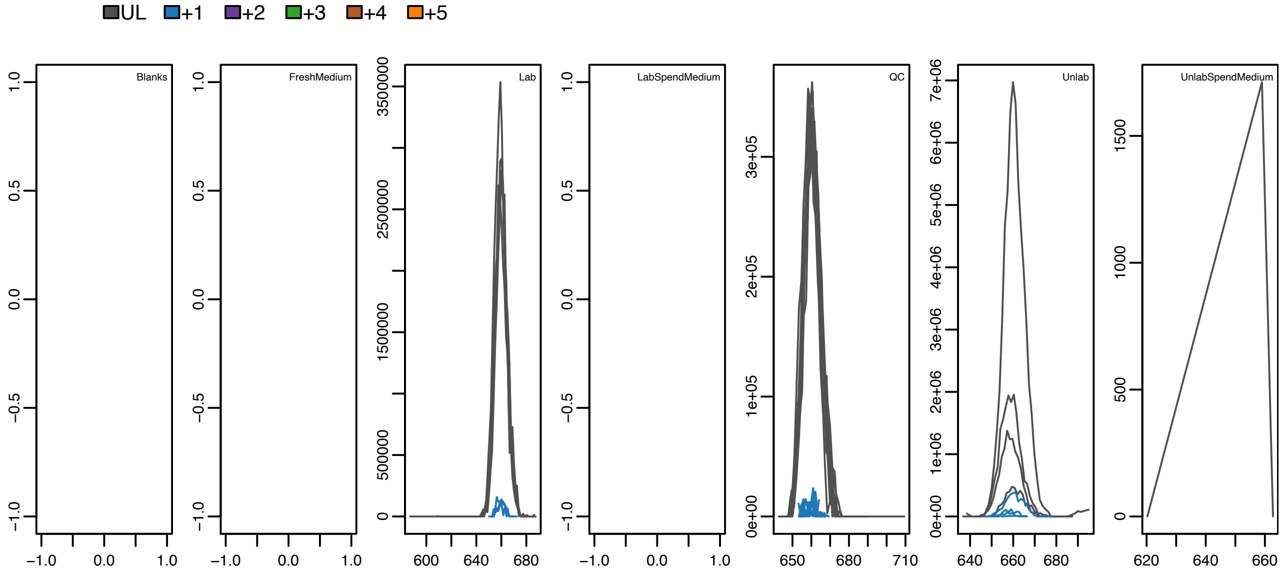

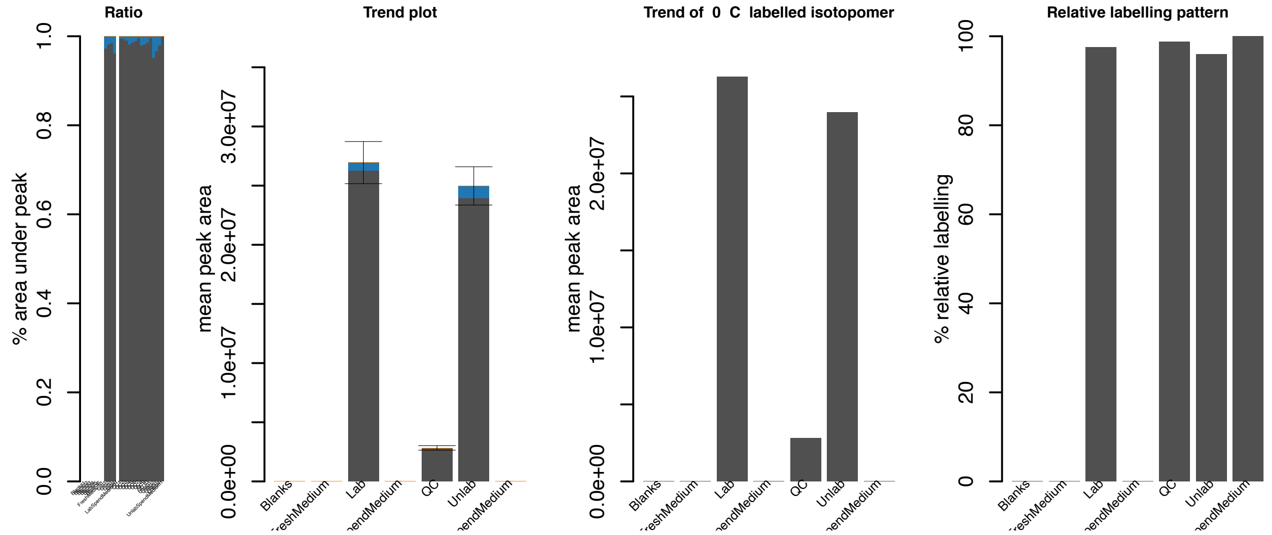

Hypoxanthine

Formula: C5H4N4O Mass: 136.039 Std.RT: 562.2 Ion: NEG

G1

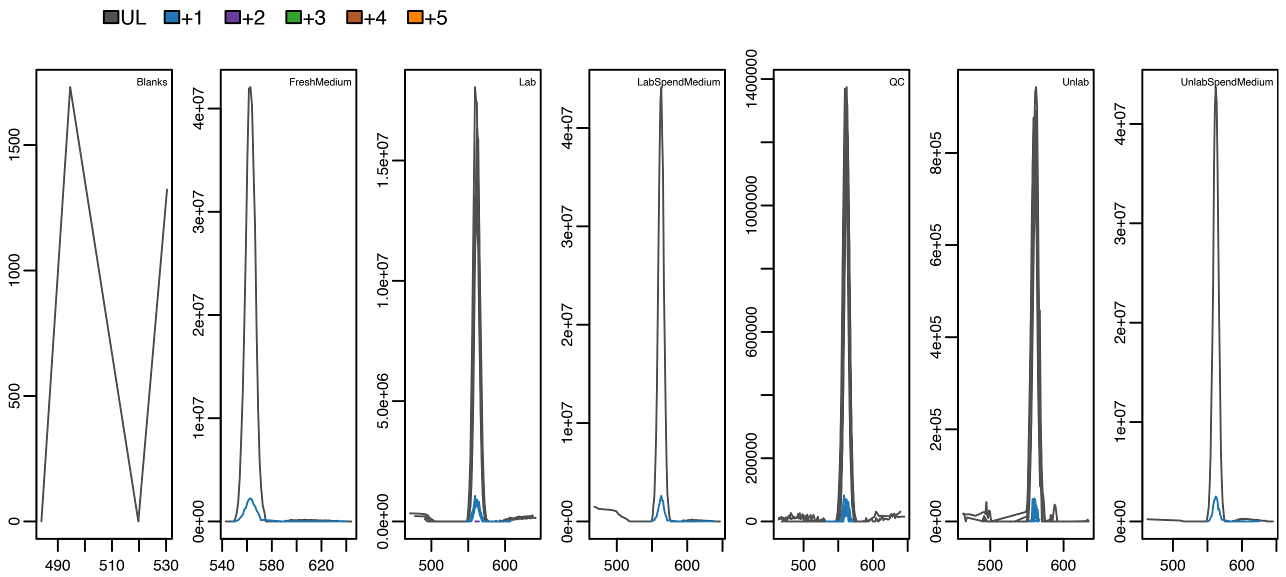

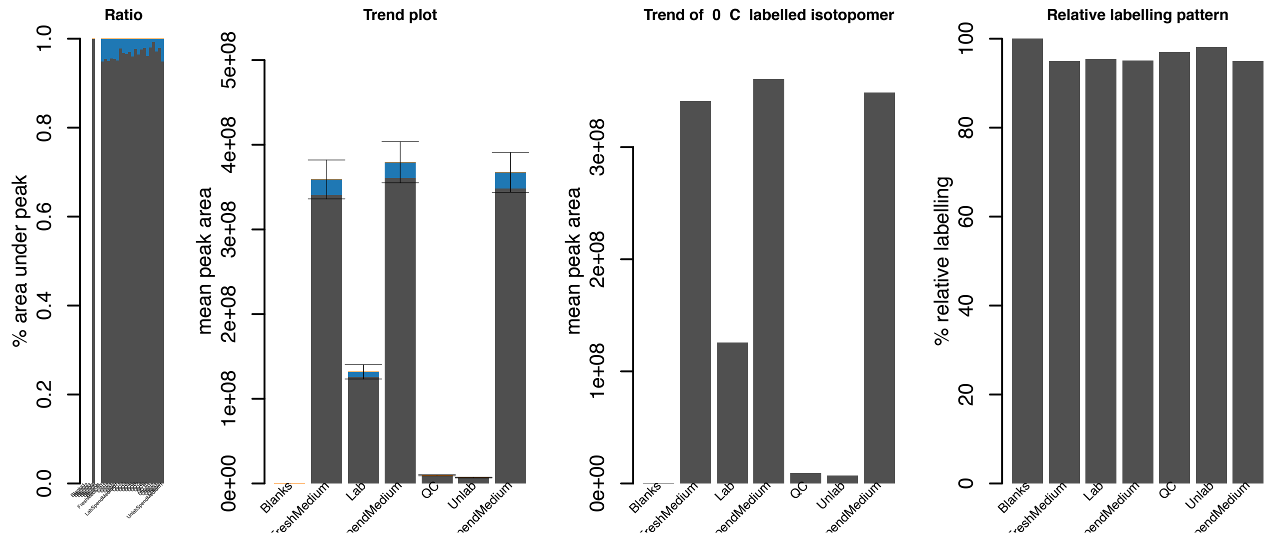

IMP

Formula: C10H13N4O8P Mass: 348.047 Std.RT: 732.6 Ion: NEG

G1

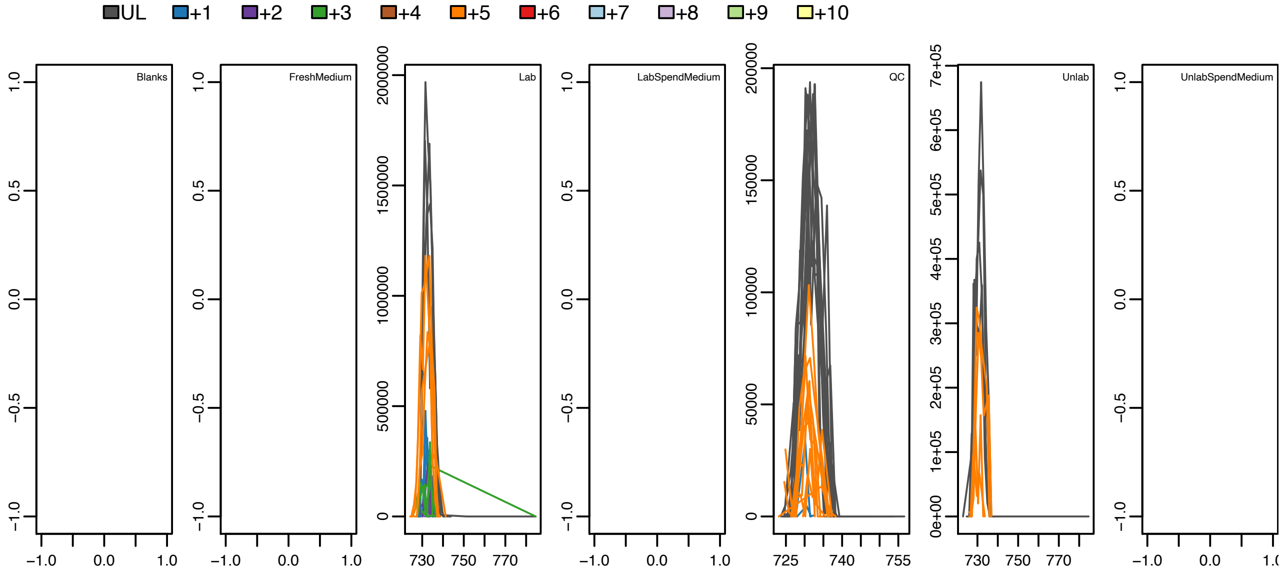

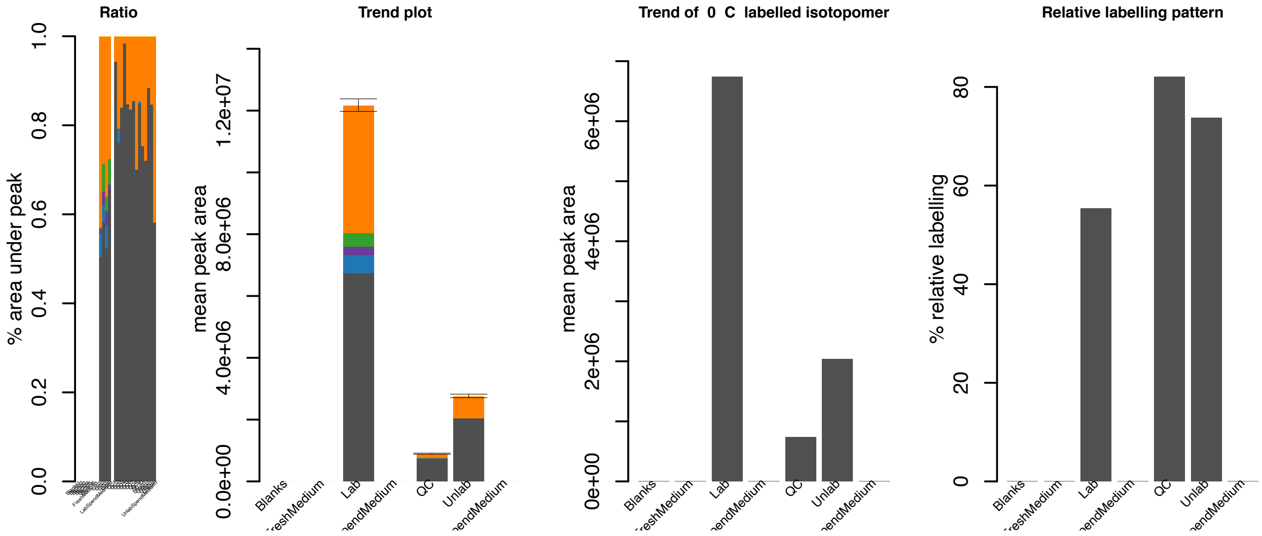

L−Alanine

Formula: C3H7NO2   Mass: 89.048 Std.RT: 737.4 Ion: NEG

G1

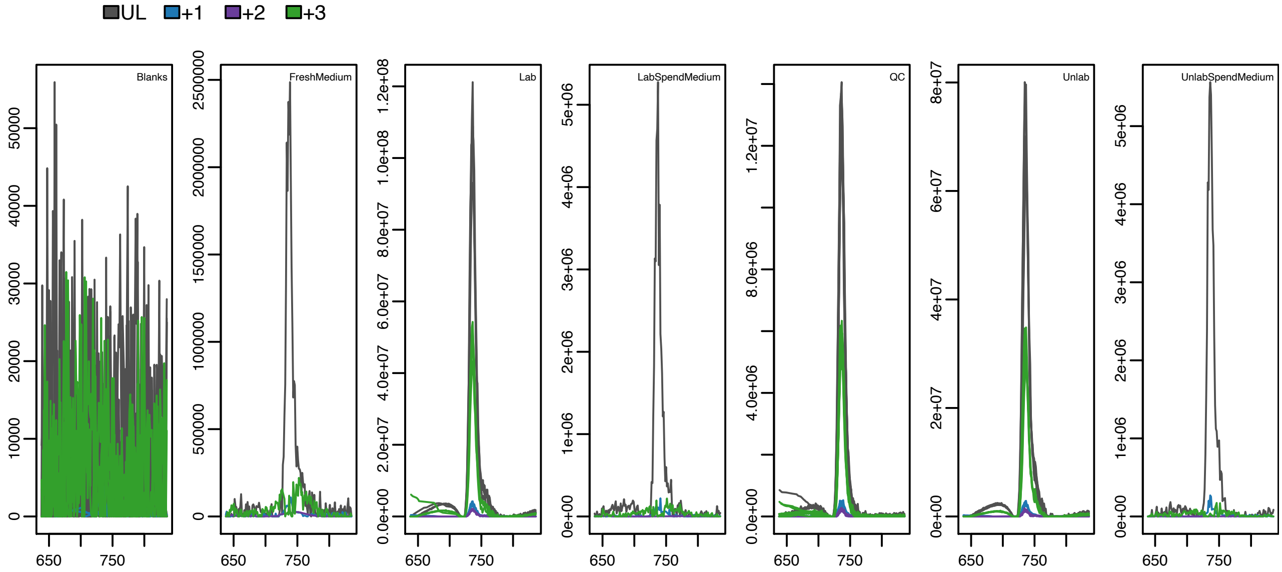

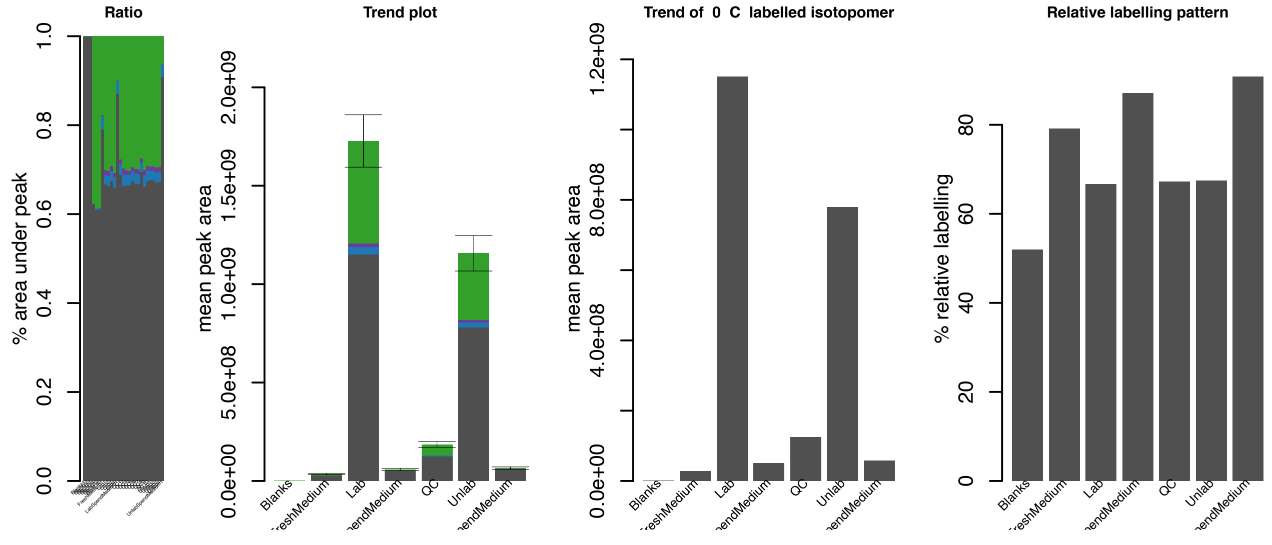

L−Arginine

Formula: C6H14N4O2 Mass: 174.112 Std.RT: 1209.6 Ion: NEG

G1

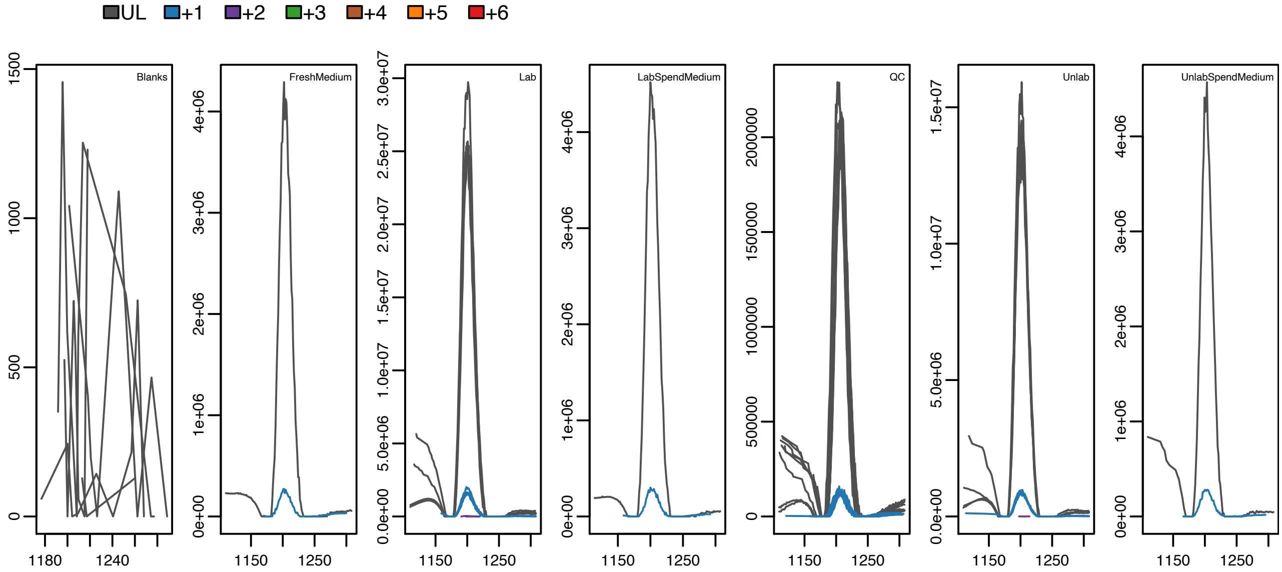

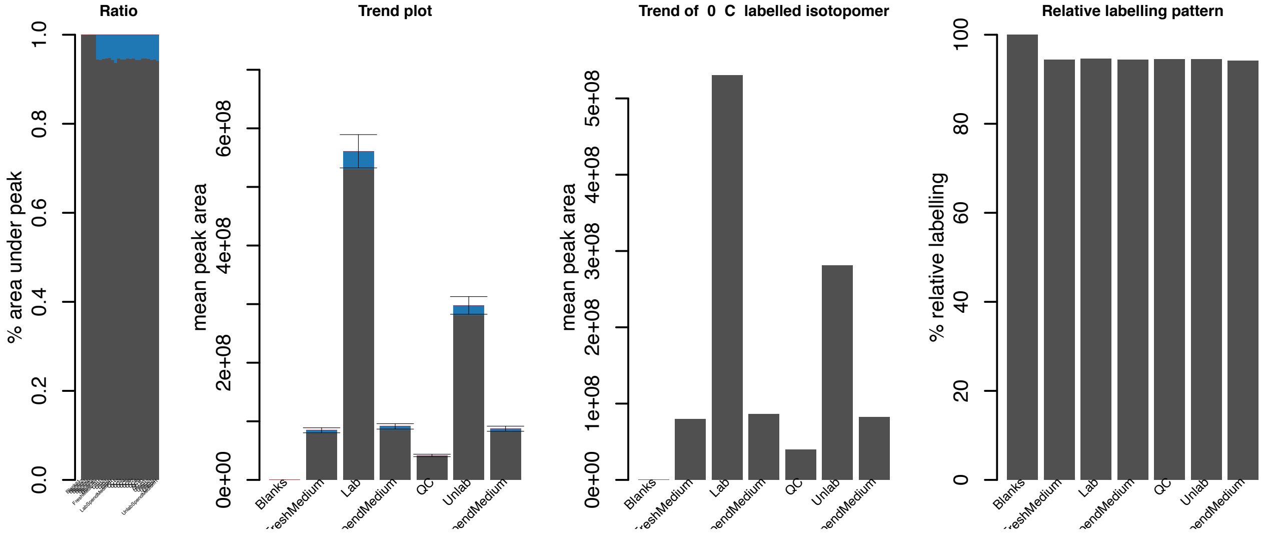

L−Asparagine

Formula: C4H8N2O3 Mass: 132.053 Std.RT: 754.8 Ion: NEG

G1

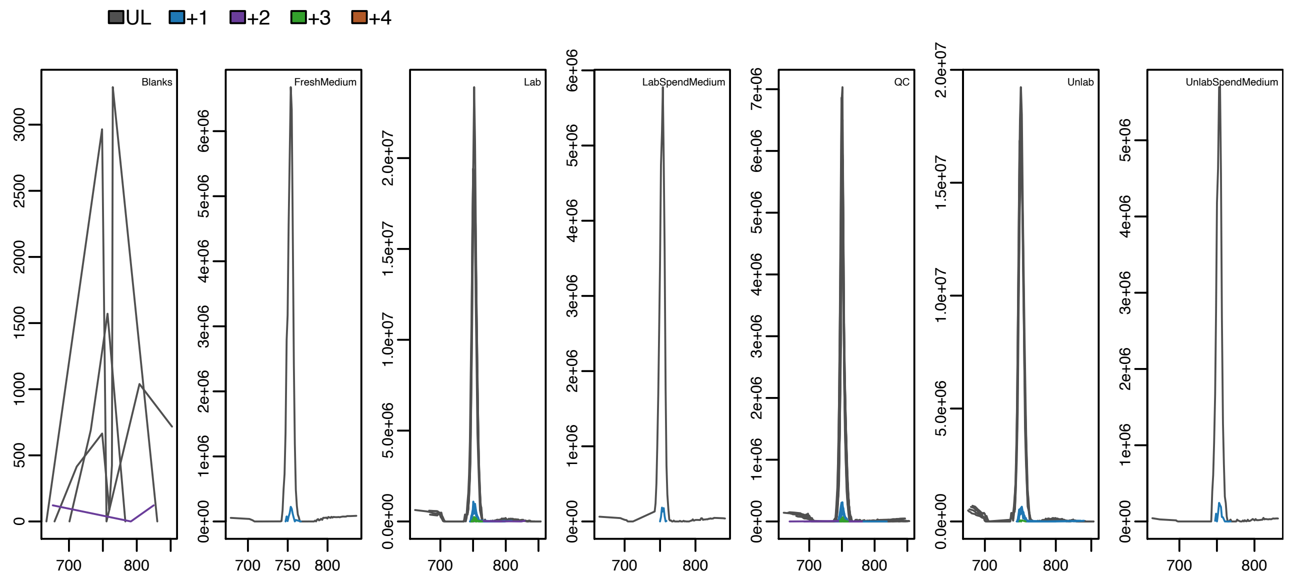

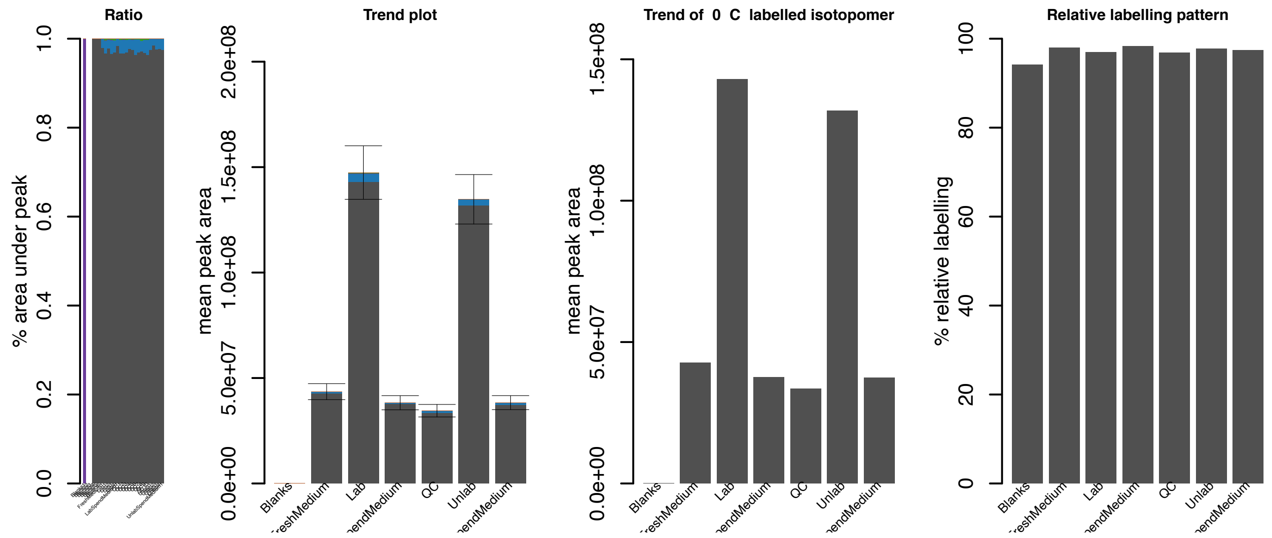

L−Aspartate

Formula: C4H7NO4 Mass: 133.038 Std.RT: 735.6 Ion: NEG

G1

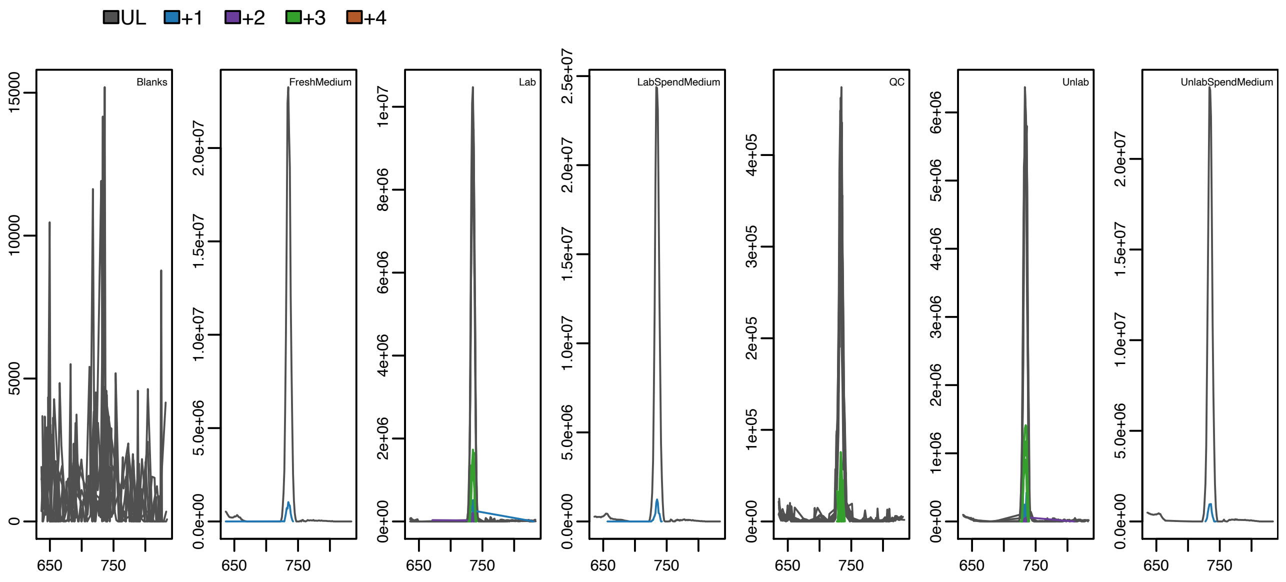

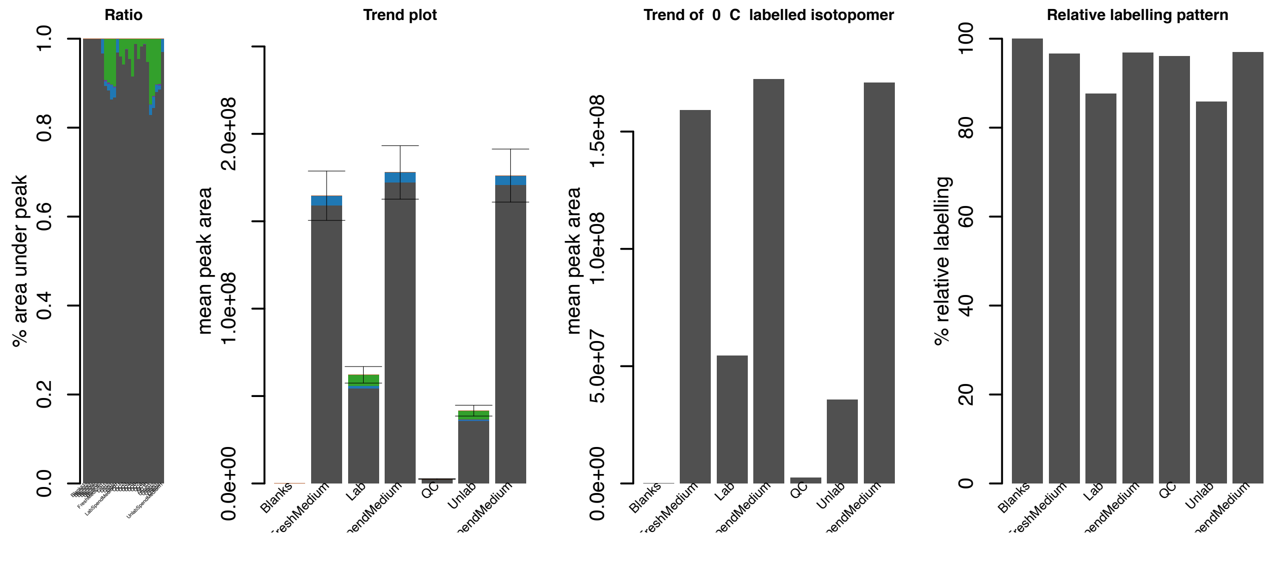

L−Glutamate

Formula: C5H9NO4 Mass: 147.053 Std.RT: 723.6 Ion: NEG

G1

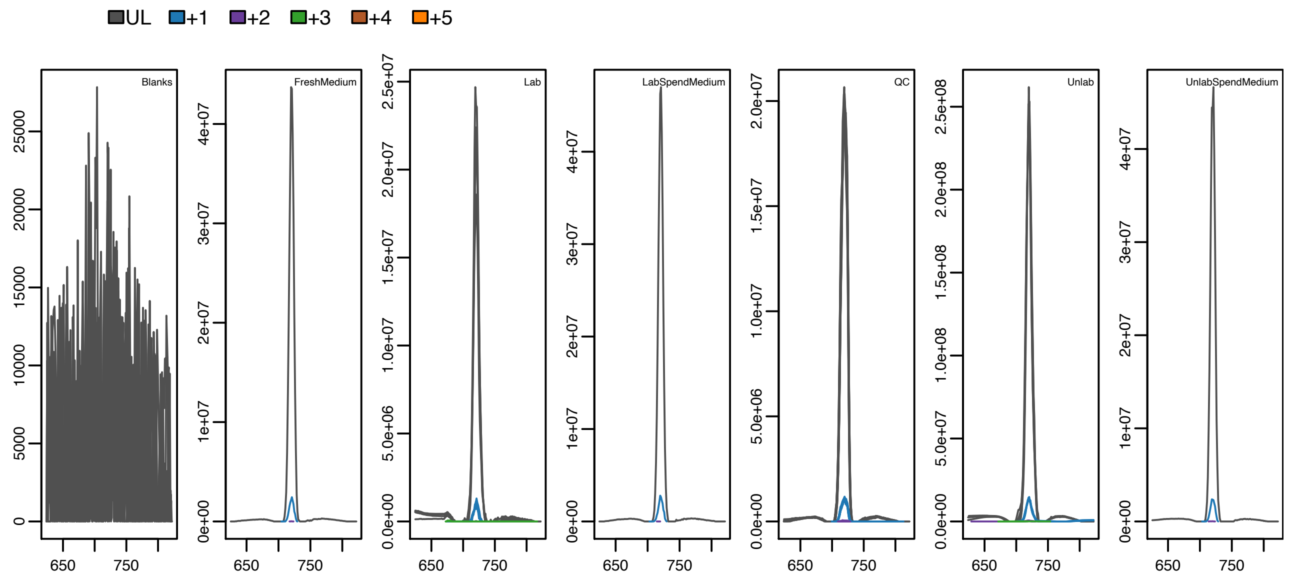

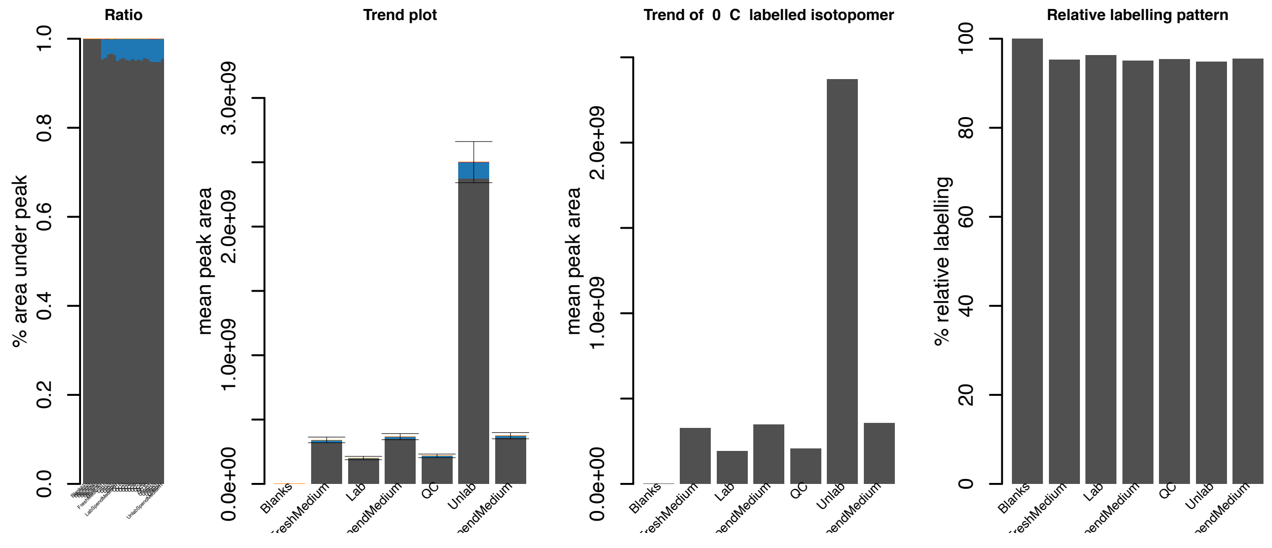

L−Glutamine

Formula: C5H10N2O3 Mass: 146.069 Std.RT: 739.2 Ion: NEG

G1

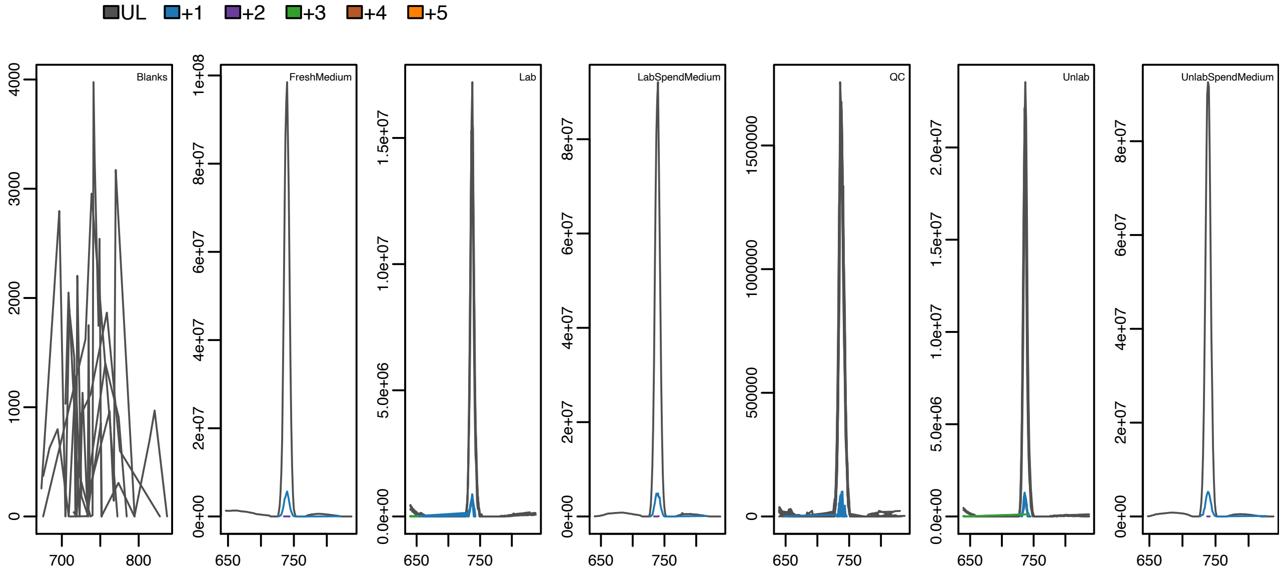

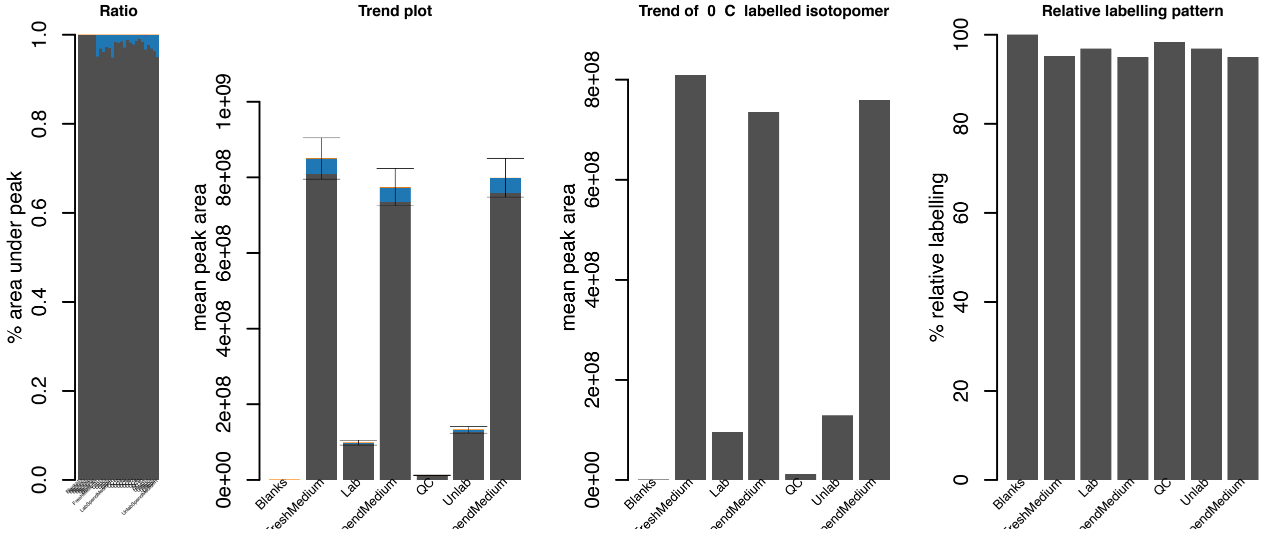

L−Lysine

Formula: $C_6H_{14}N_2O_2$ Mass: 146.106 Std.RT: 1111.8 Ion: NEG

G1

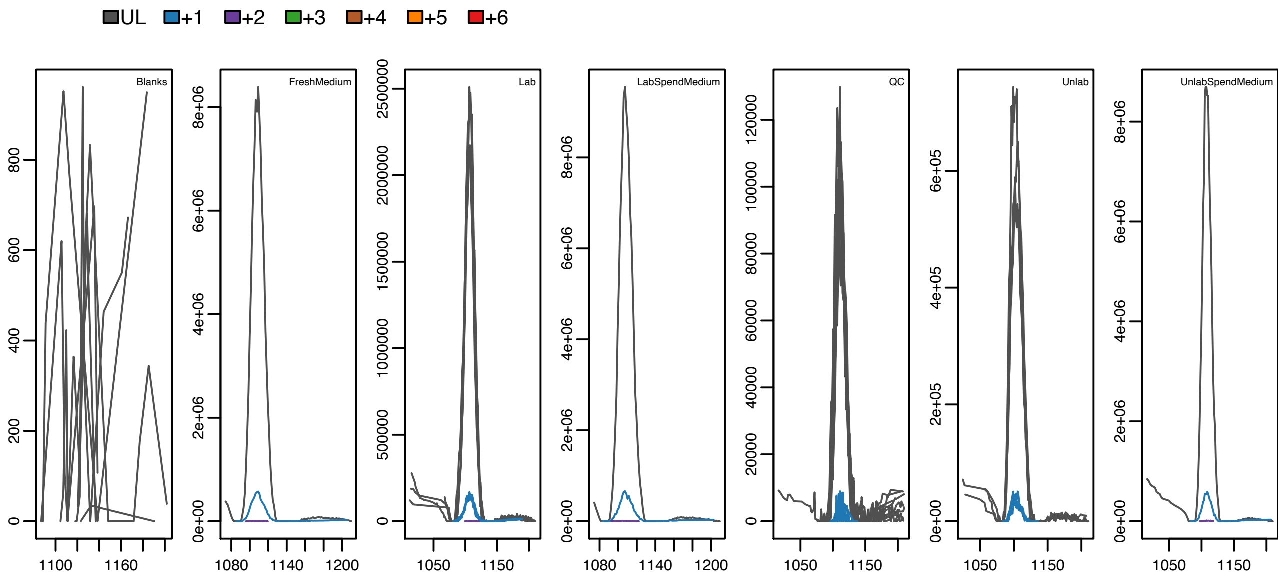

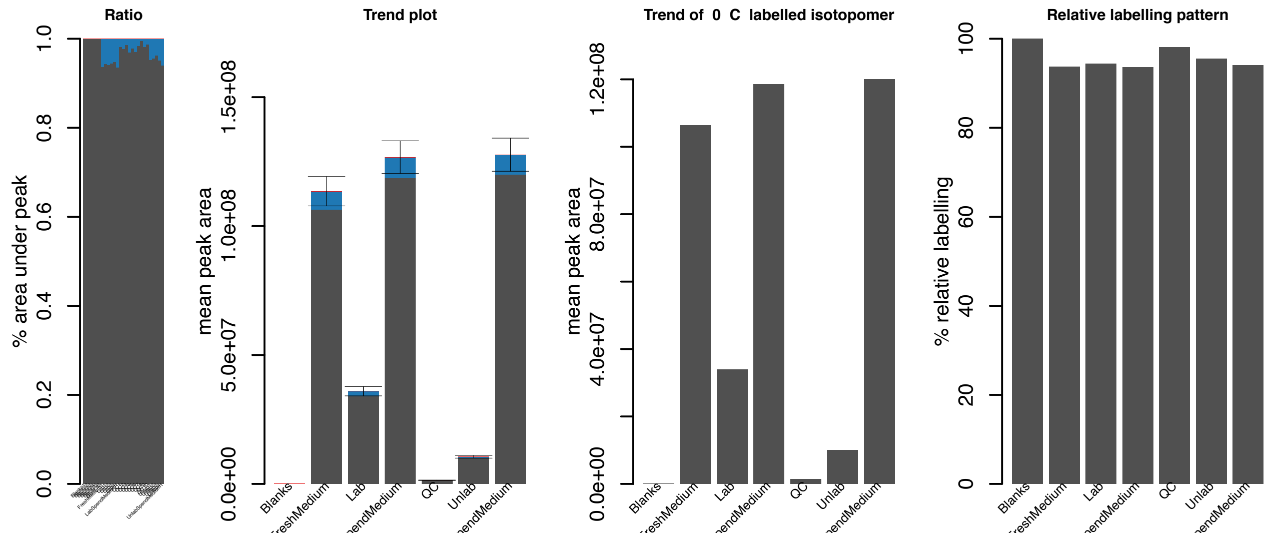

L−Methionine

Formula: C5H11NO2S Mass: 149.051 Std.RT: 613.8 Ion: NEG

G1

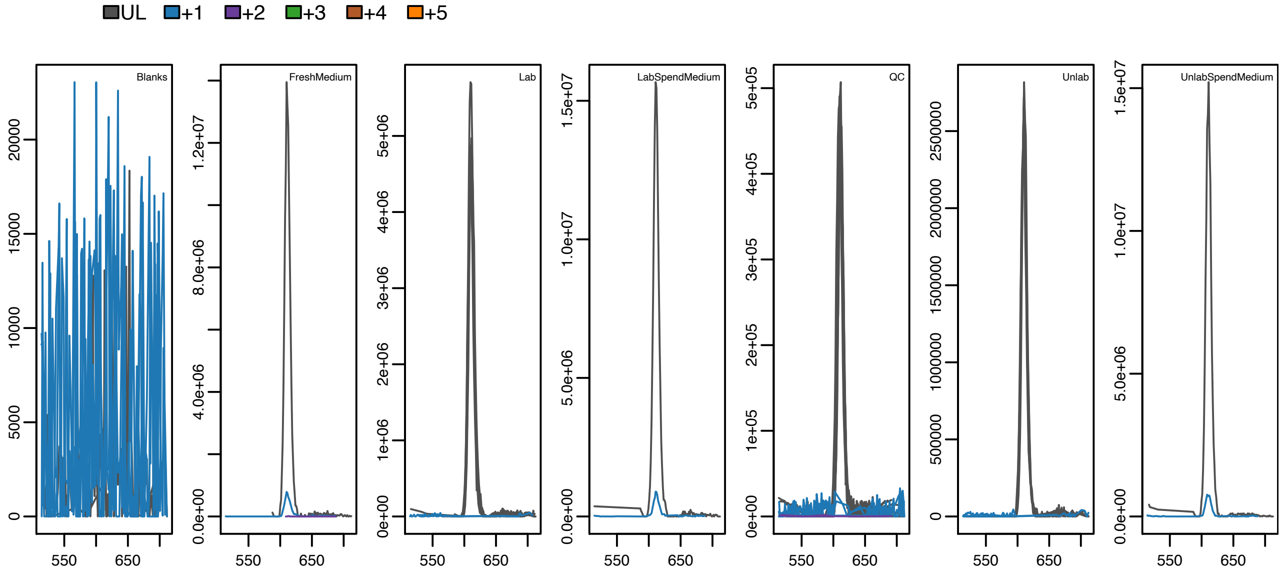

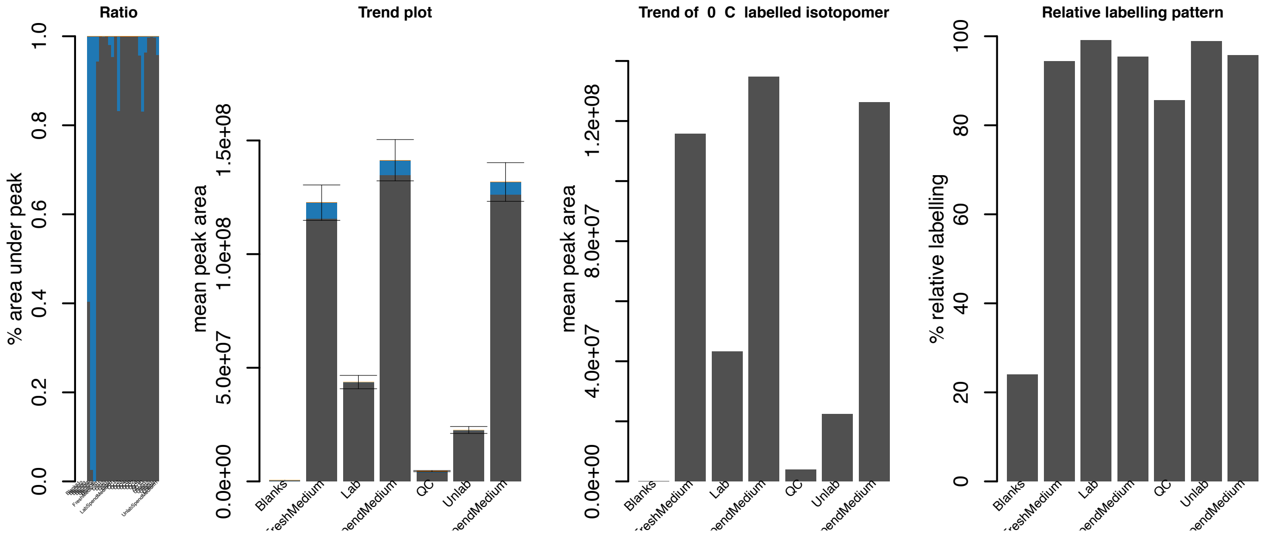

L−Ornithine

Formula: C5H12N2O2   Mass: 132.09 Std.RT: 1044 Ion: NEG

G1

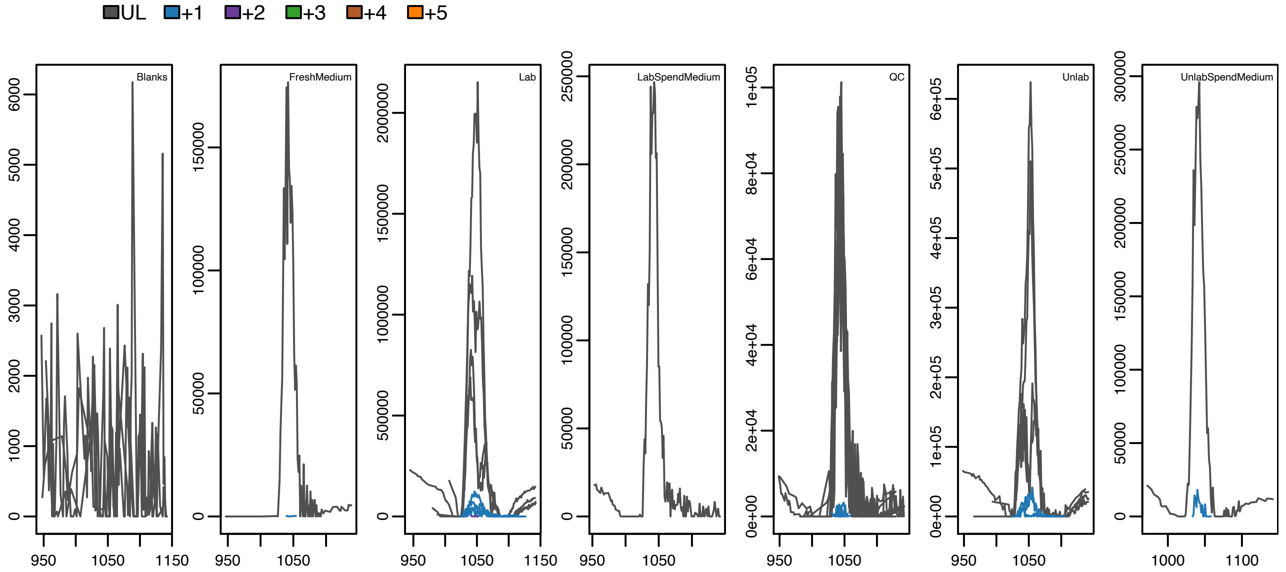

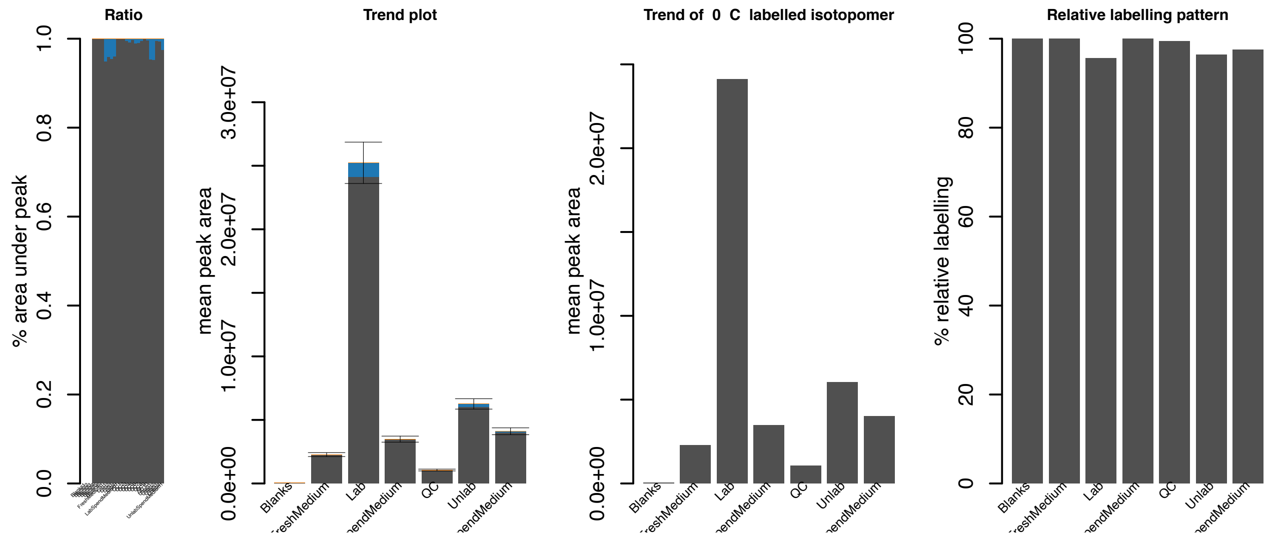

L−Phenylalanine

Formula: $C_9H_{11}NO_2$ Mass: 165.079 Std.RT: 552.6 Ion: NEG

G1

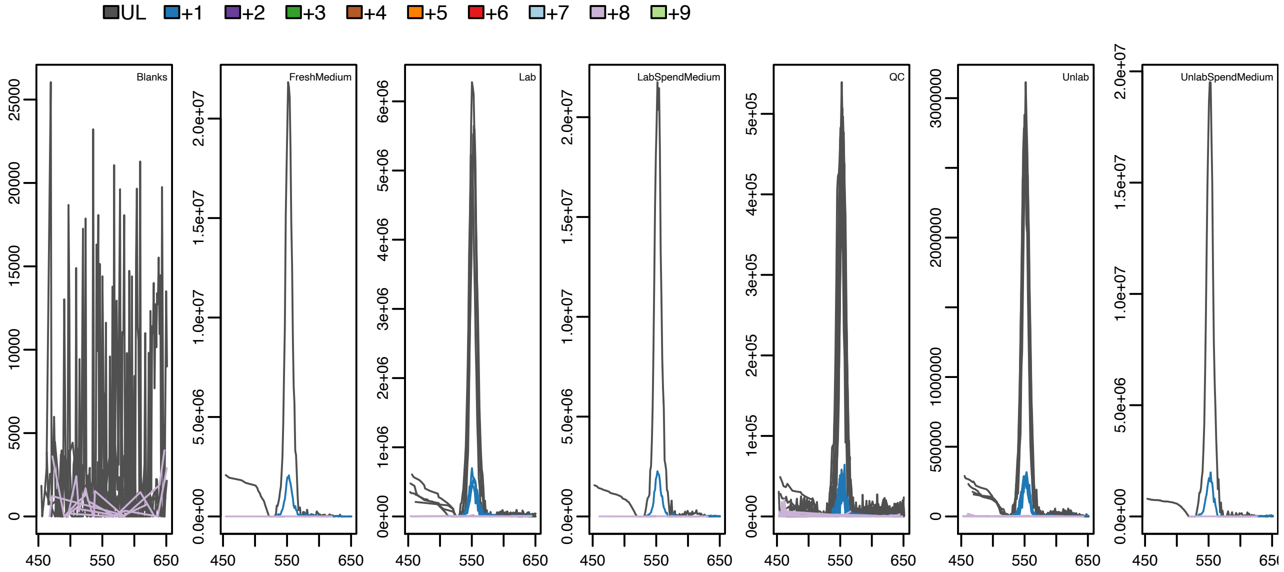

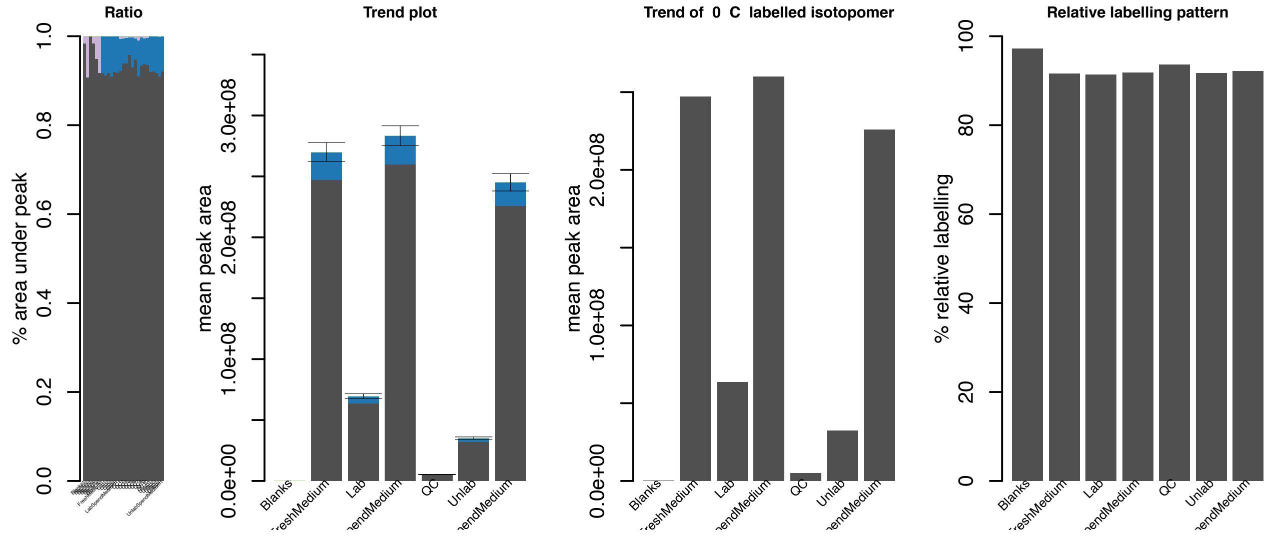

L−Proline

Formula: C5H9NO2 Mass: 115.063 Std.RT: 661.2 Ion: NEG

G1

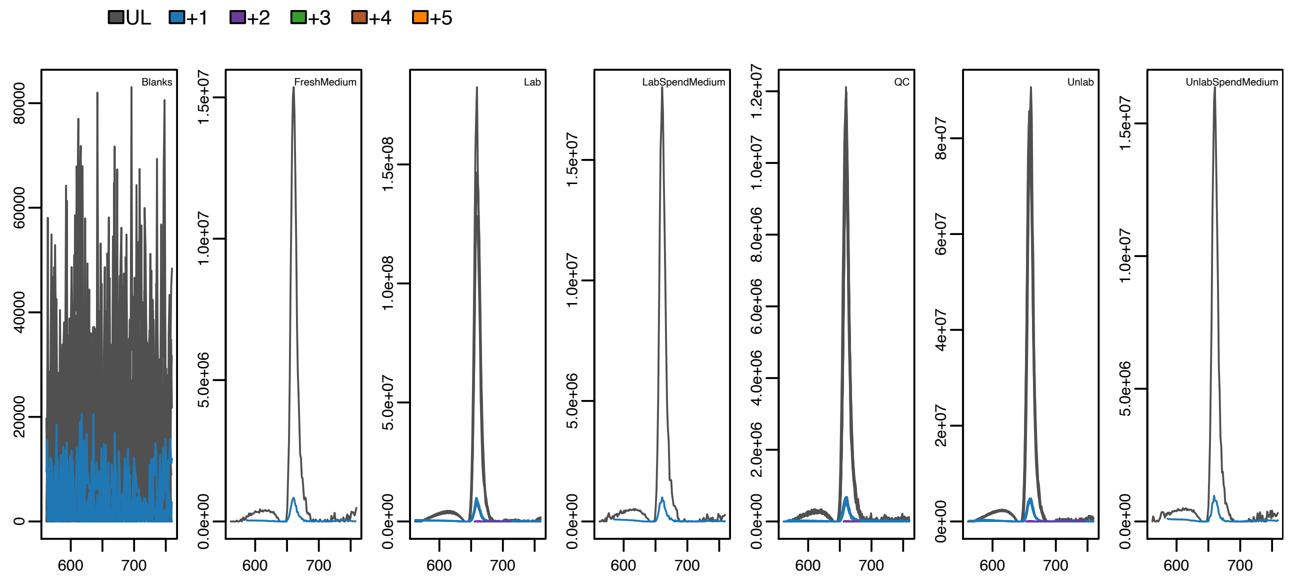

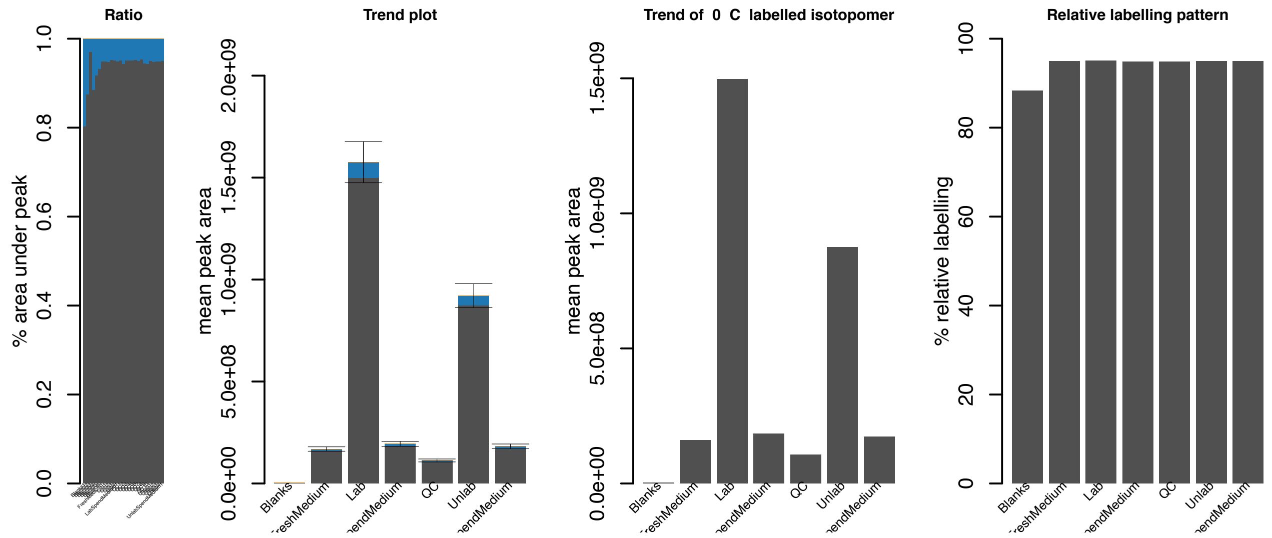

L−Serine

Formula: C3H7NO3 Mass: 105.043 Std.RT: 775.2 Ion: NEG

G1

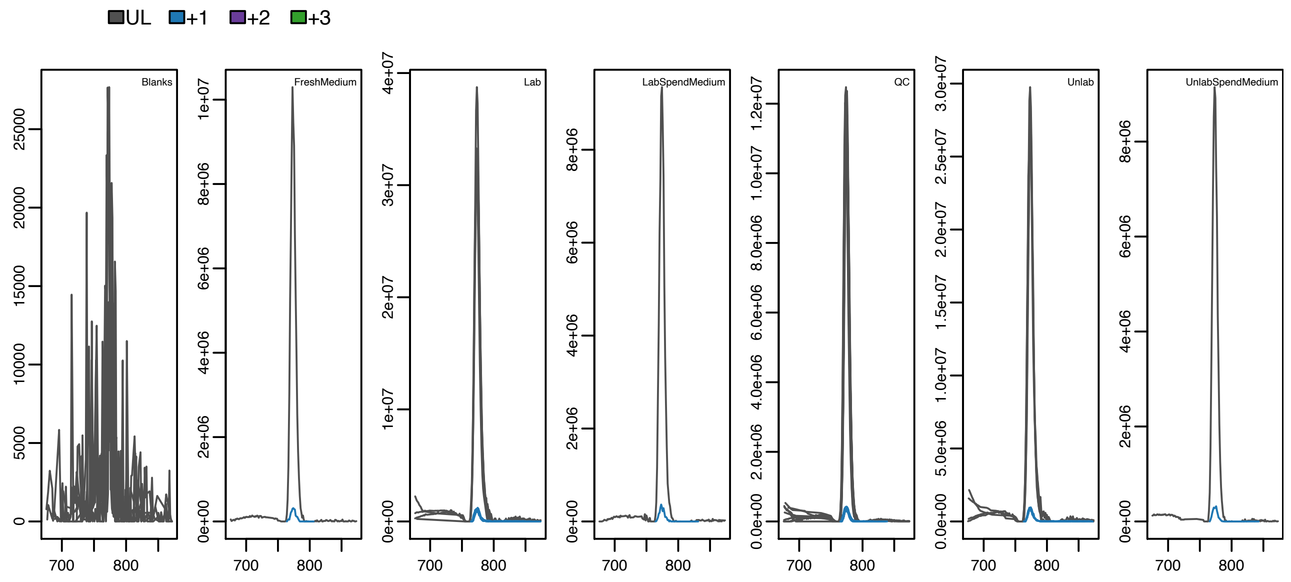

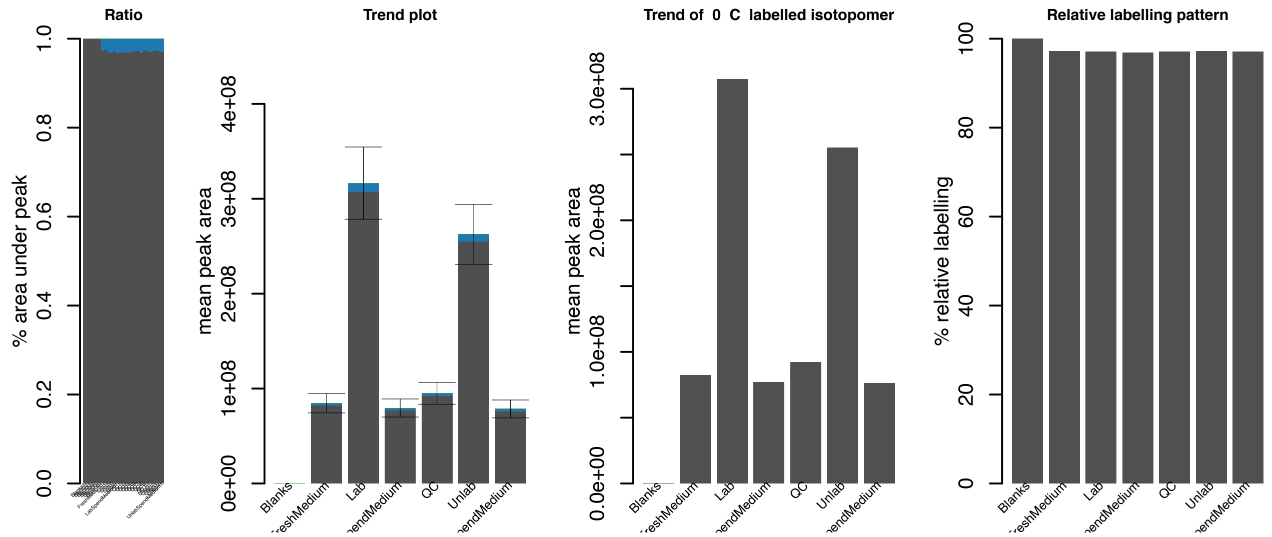

L−Threonine

Formula: C4H9NO3 Mass: 119.058 Std.RT: 728.4 Ion: NEG

G1

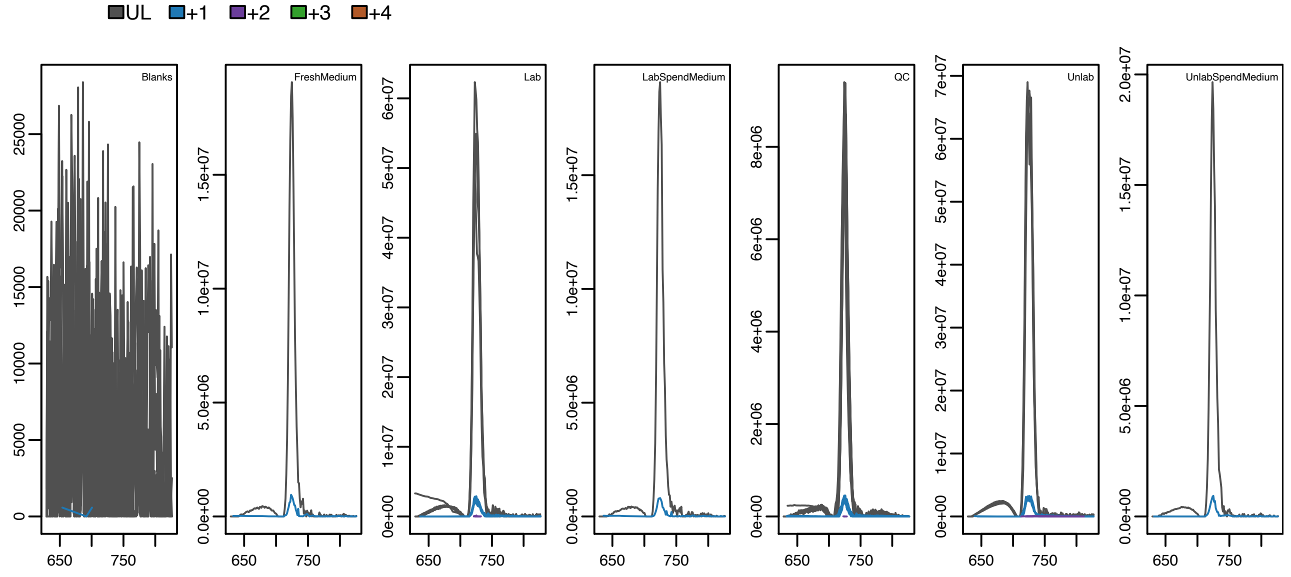

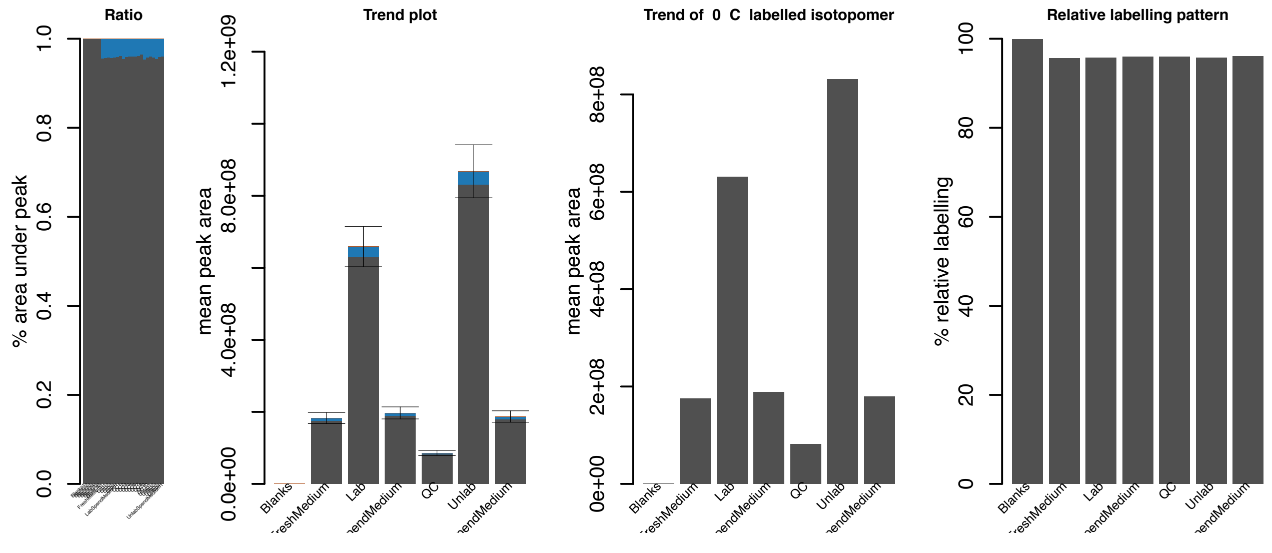

L−Tryptophan

Formula: C11H12N2O2 Mass: 204.09 Std.RT: 630 Ion: NEG

G1

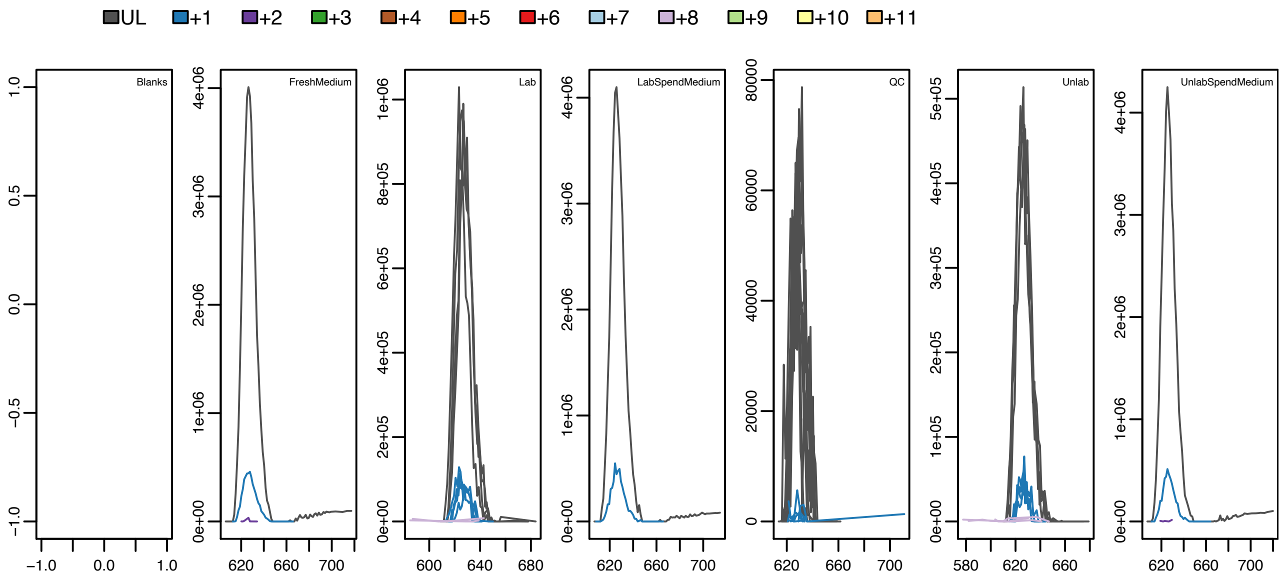

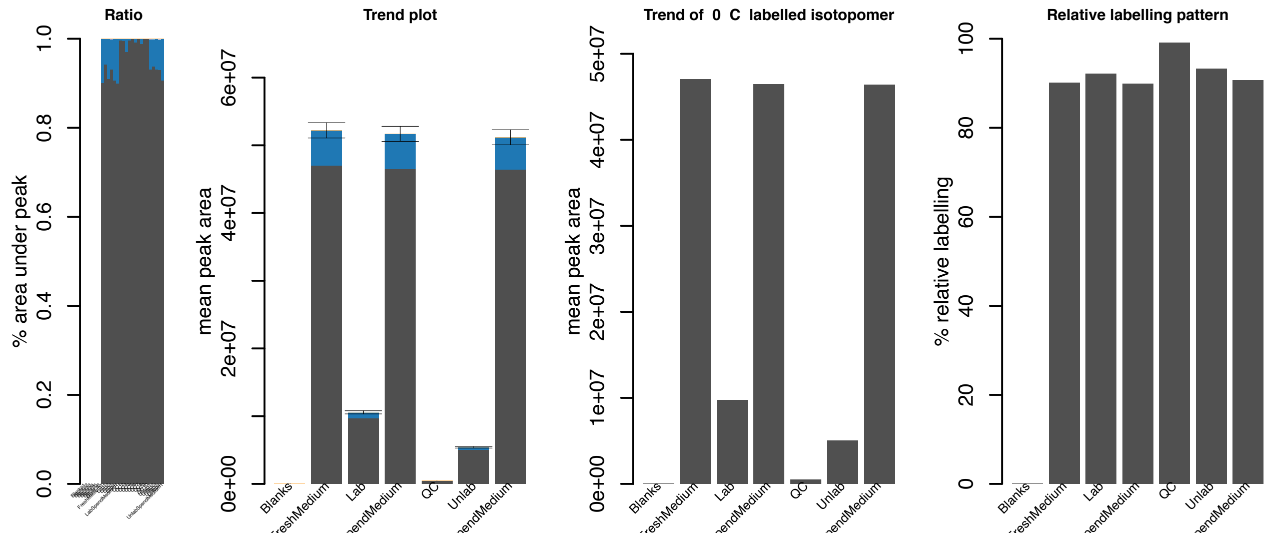

L−Tyrosine

Formula: C9H11NO3 Mass: 181.074 Std.RT: 680.4 Ion: NEG

G1

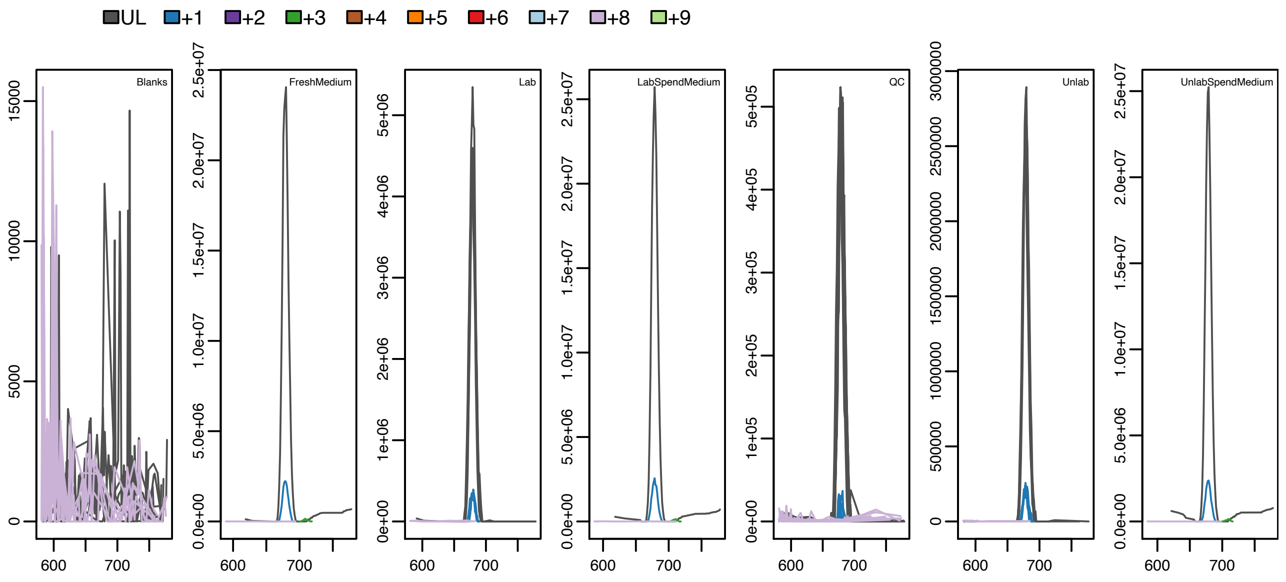

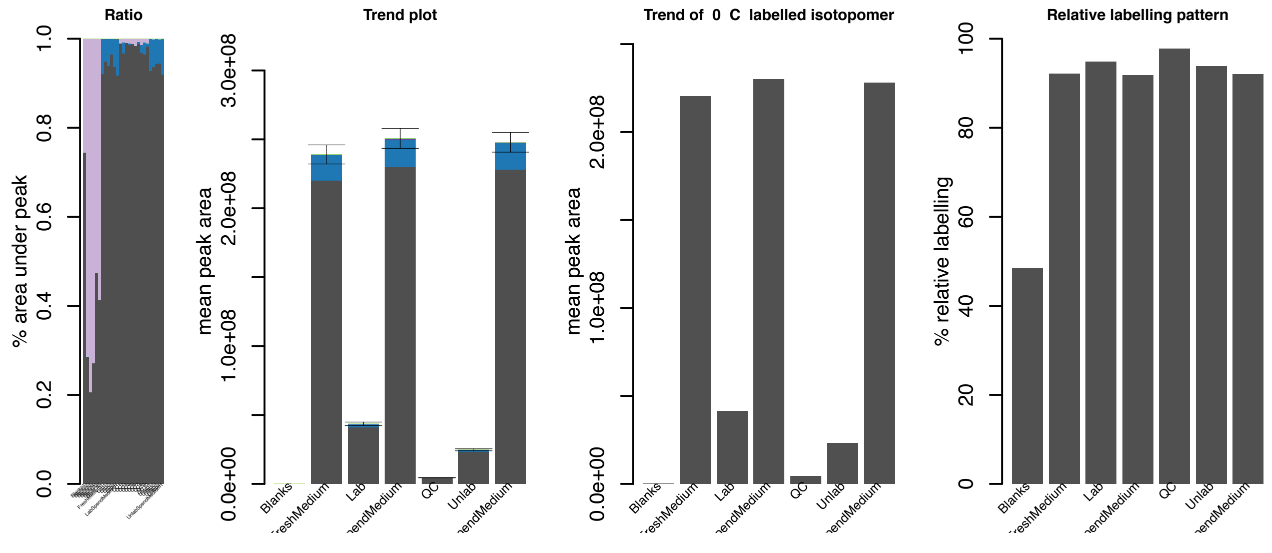

L−Valine

Formula: C5H11NO2 Mass: 117.079 Std.RT: 651 Ion: NEG

G1

UL +1 +2 +3 +4 +5

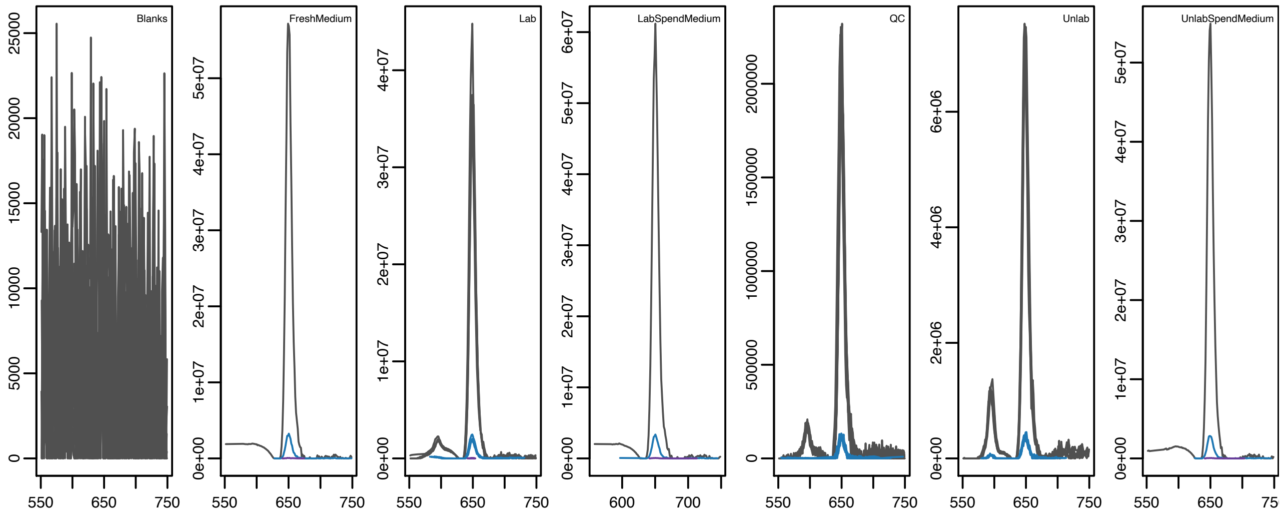

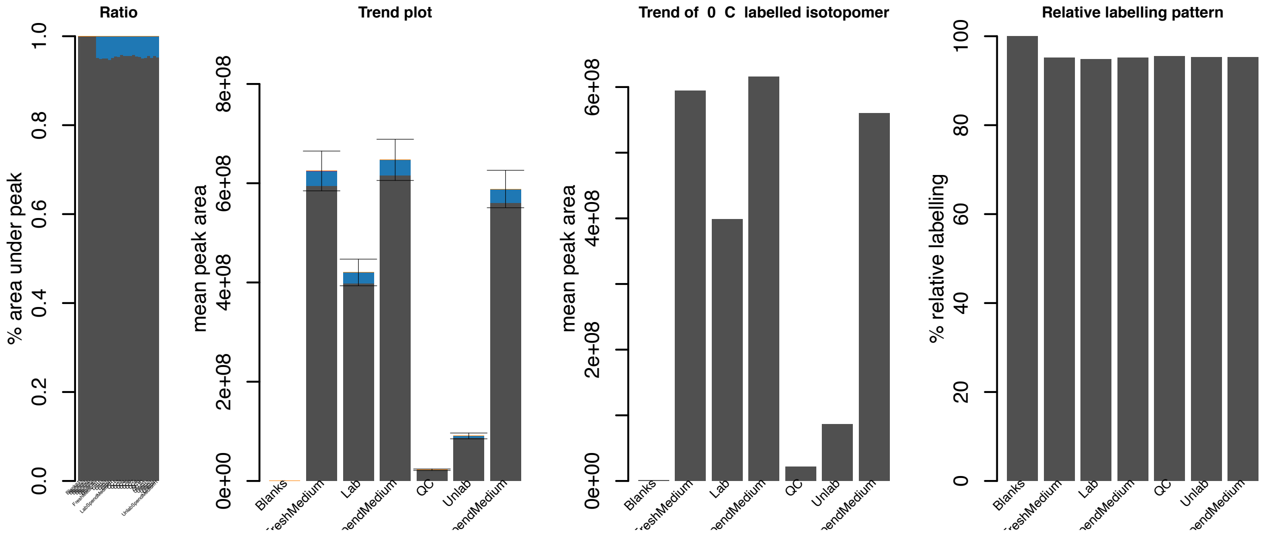

L−isoleucine

Formula: C6H13NO2 Mass: 131.095 Std.RT: 623.4 Ion: NEG

G1

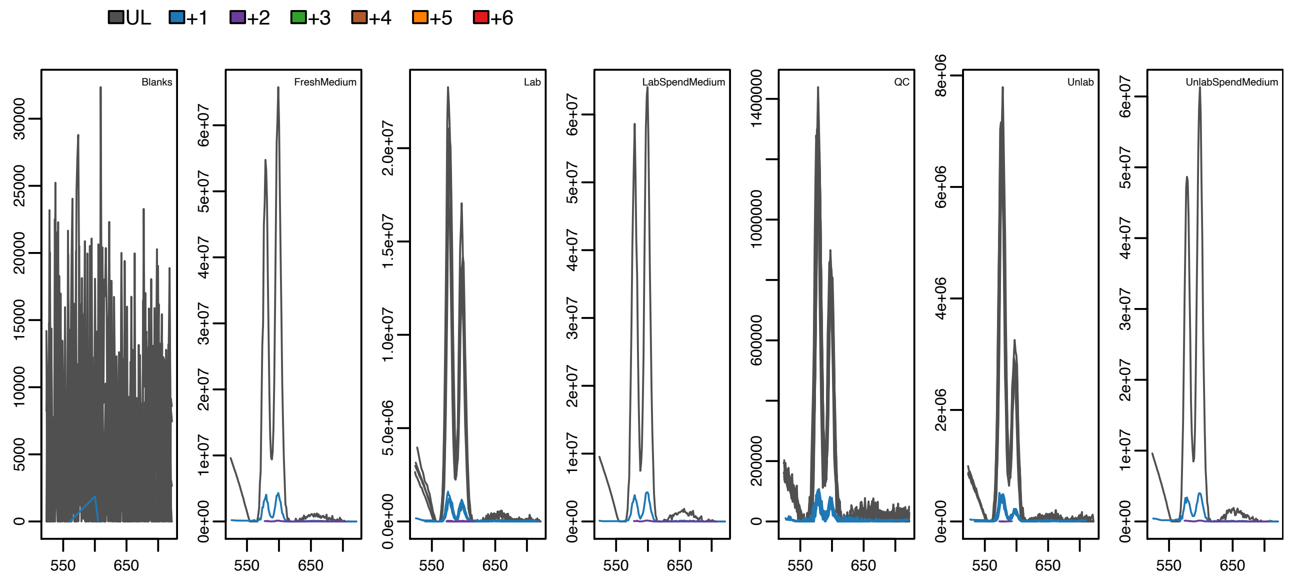

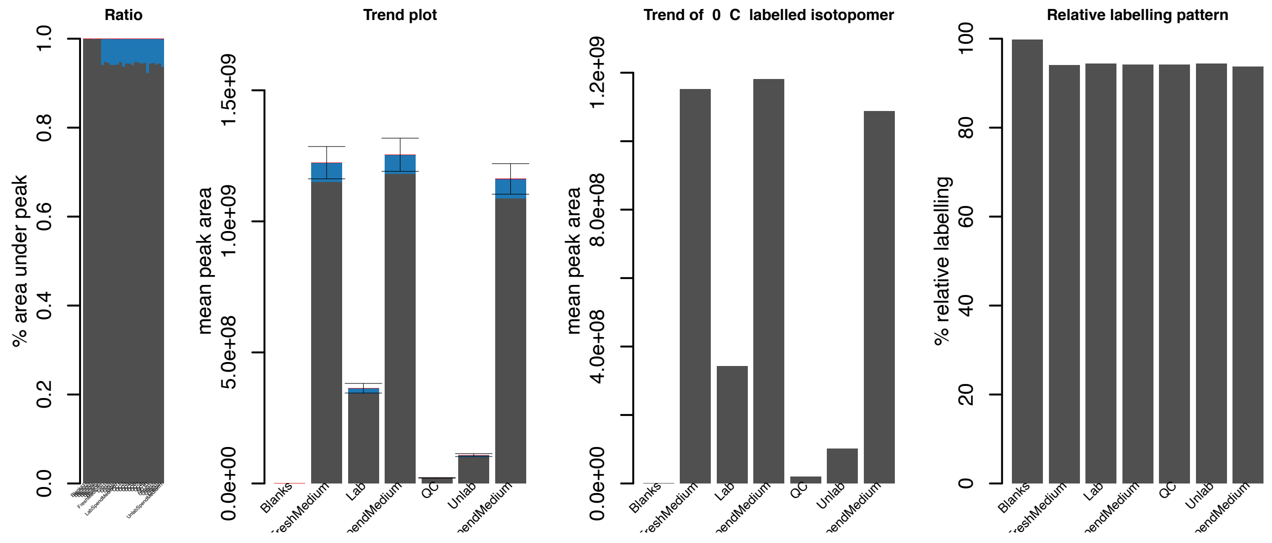

N−Acetylornithine

Formula: C7H14N2O3 Mass: 174.1 Std.RT: 735 Ion: NEG

G1

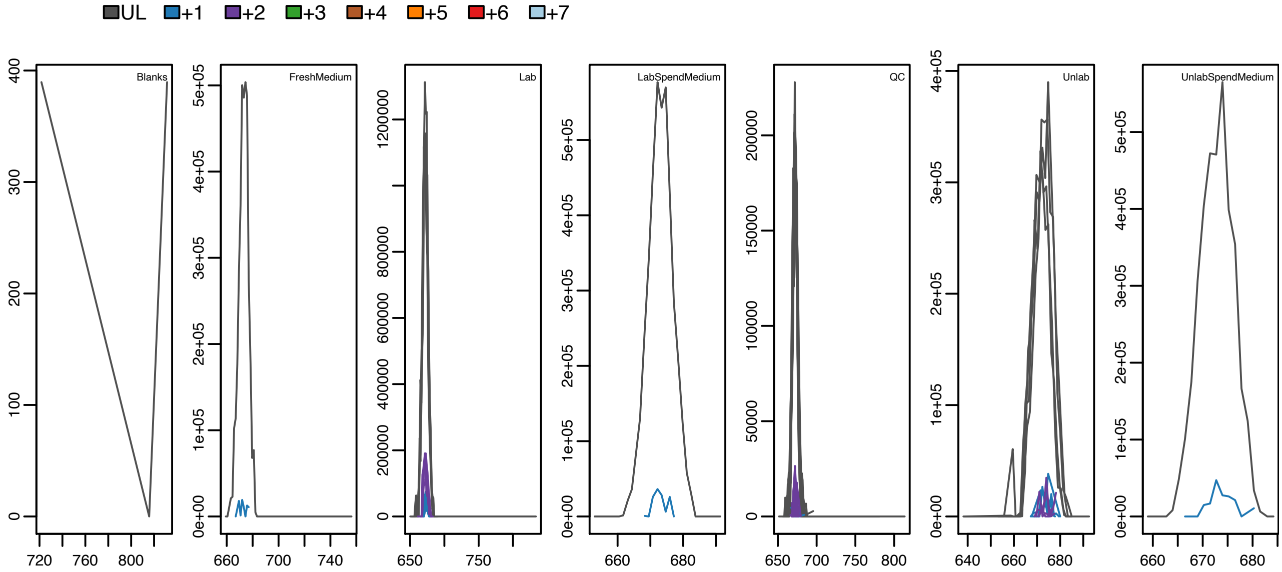

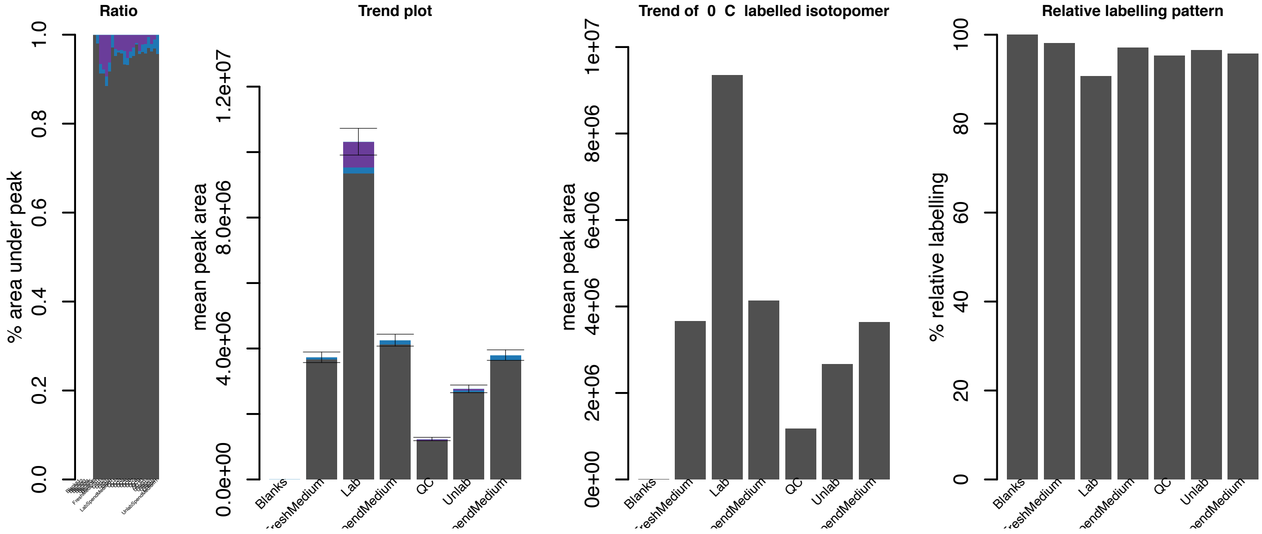

N2−Acetyl−L−lysine

G1

Formula: C8H16N2O3 Mass: 188.116 Std.RT: 727.2 Ion: NEG

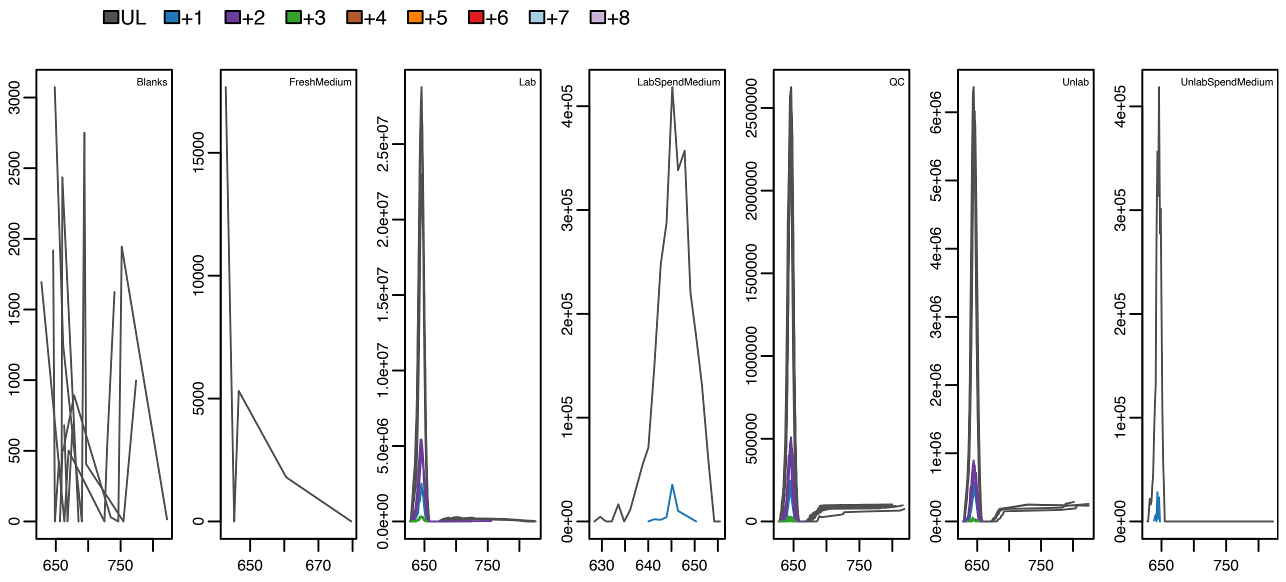

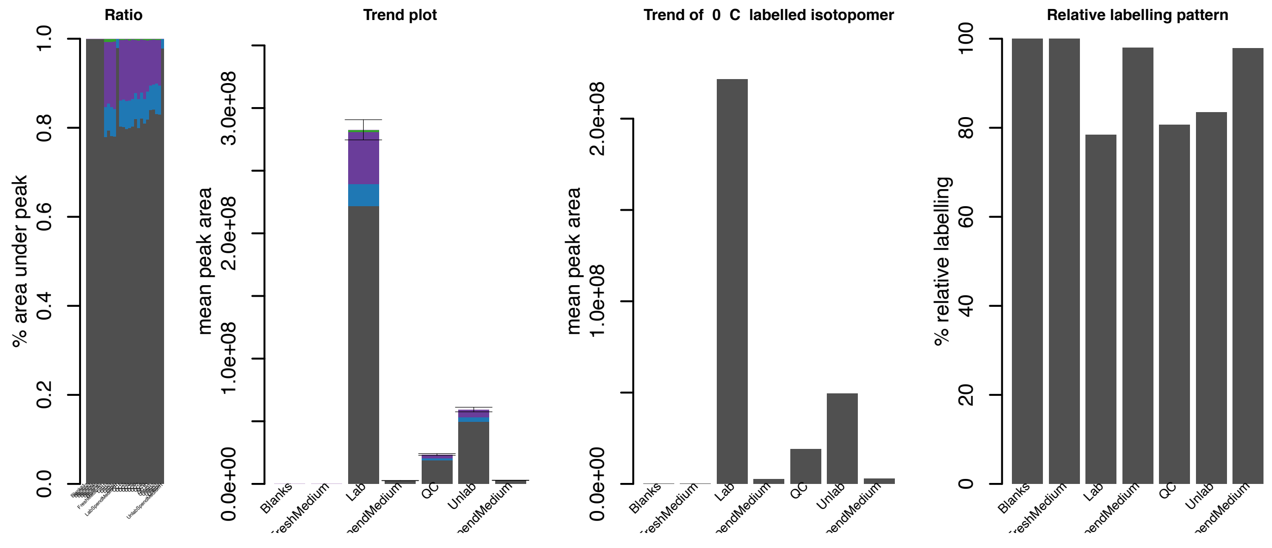

Nicotinamide

Formula: C6H6N2O   Mass: 122.048 Std.RT: 389.4 Ion: NEG

G1

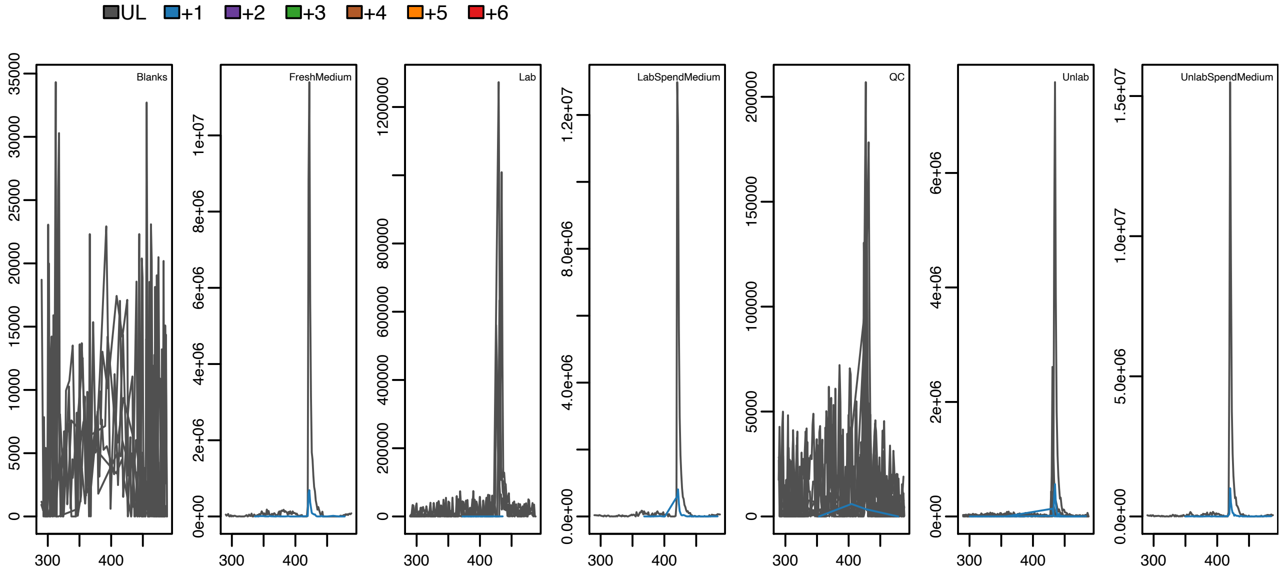

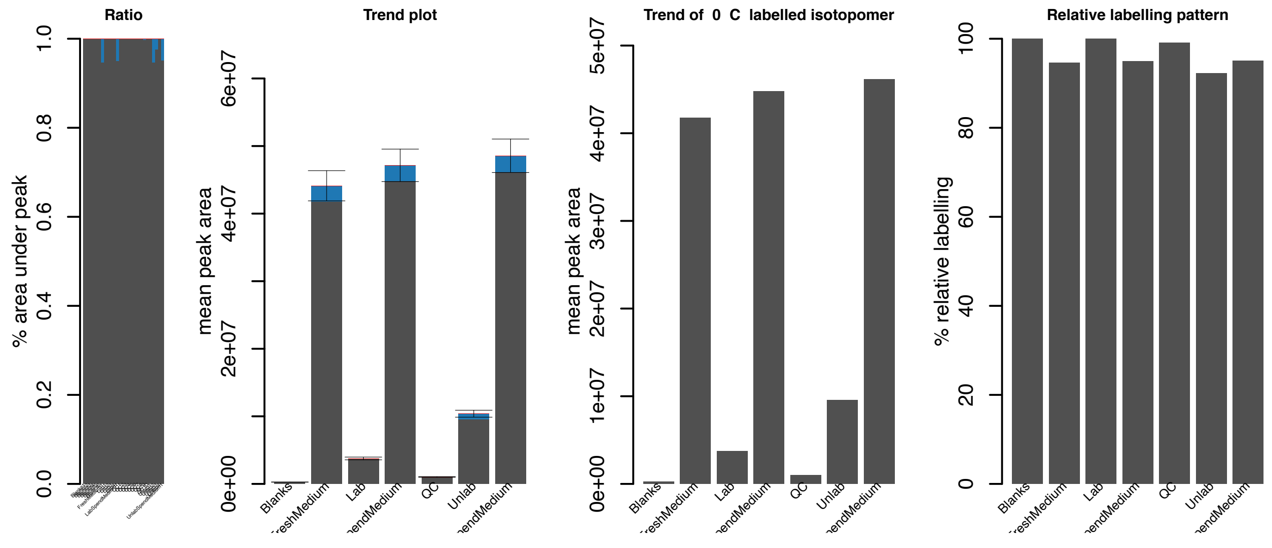

Nicotinate

Formula: C6H5NO2 Mass: 123.032 Std.RT: 435 Ion: NEG

G1

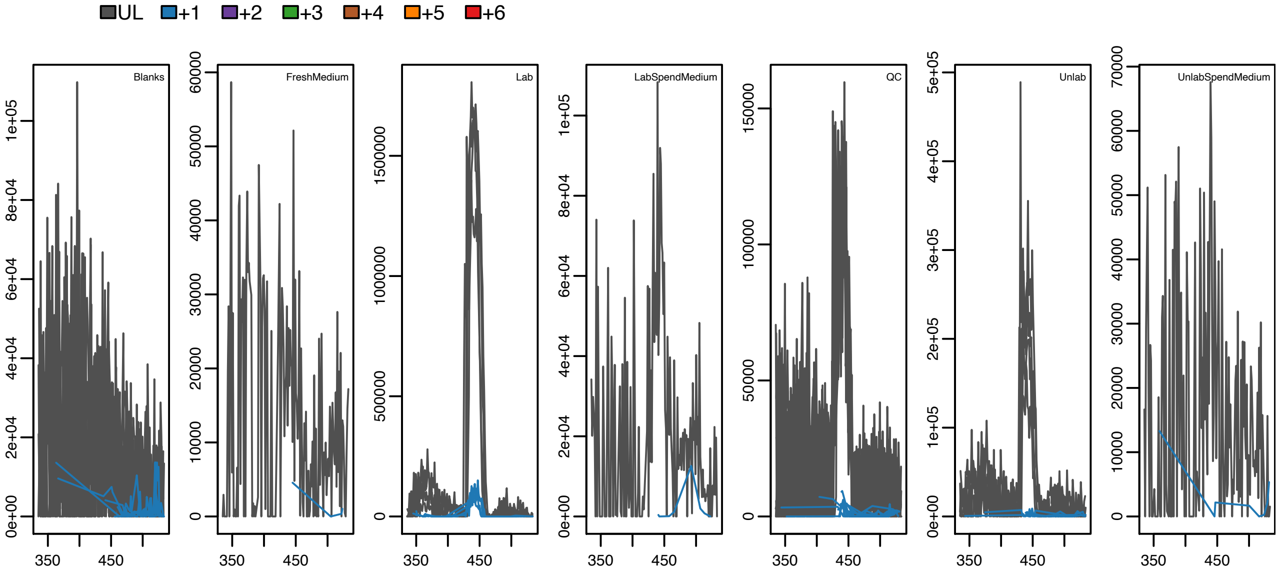

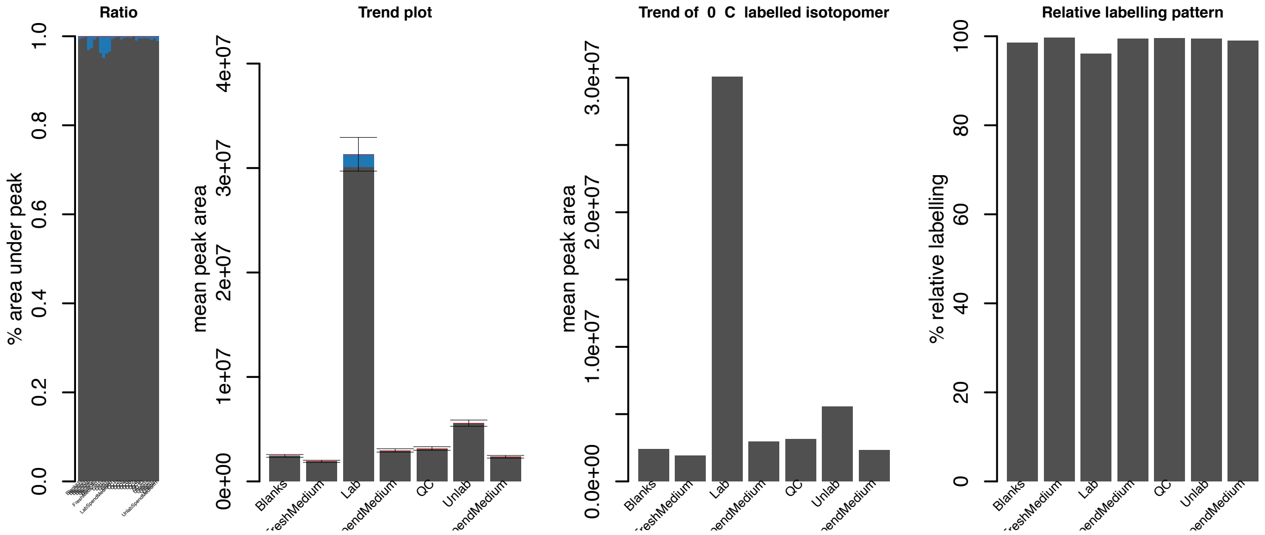

O−Acetyl−L−serine

Formula: C5H9NO4 Mass: 147.053 Std.RT: 701.4 Ion: NEG

G1

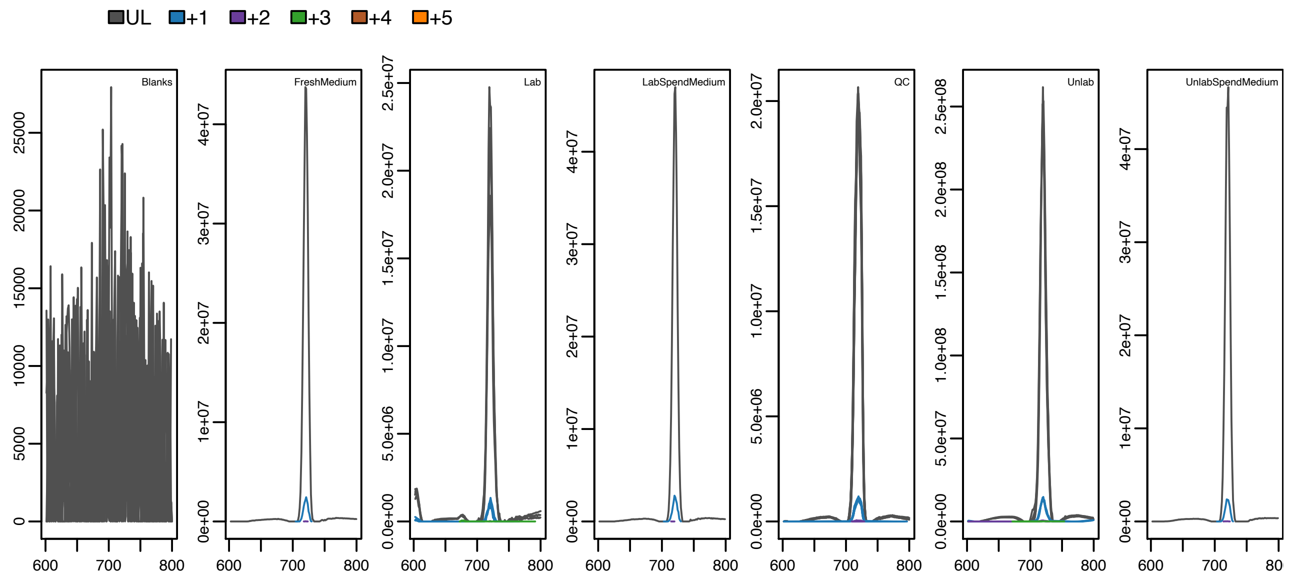

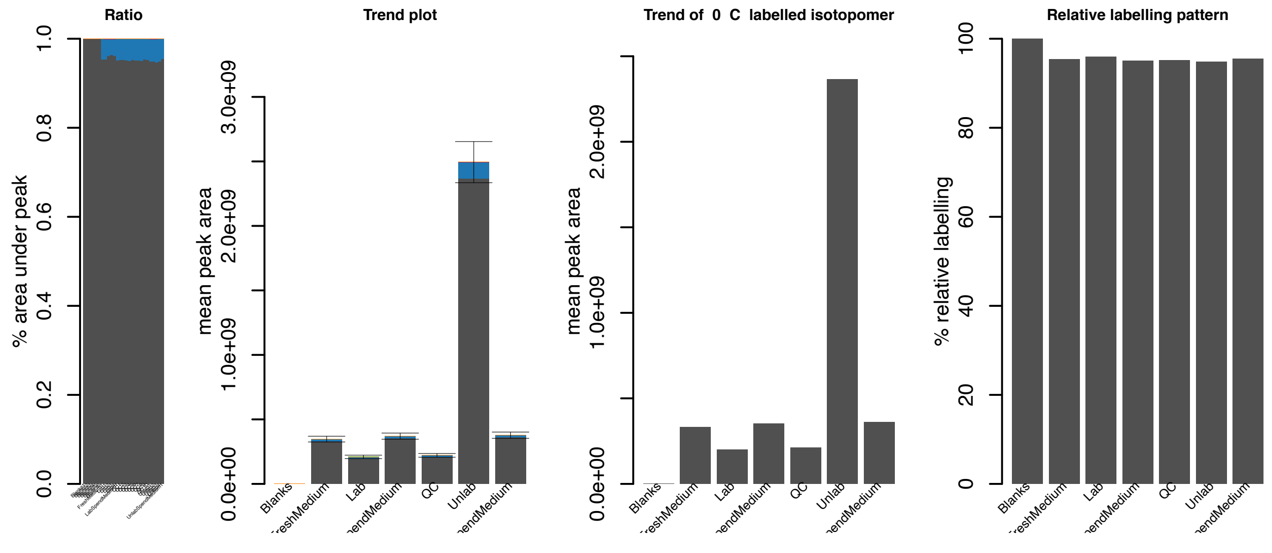

Orotate

G1

Formula: C5H4N2O4 Mass: 156.017 Std.RT: 586.8 Ion: NEG

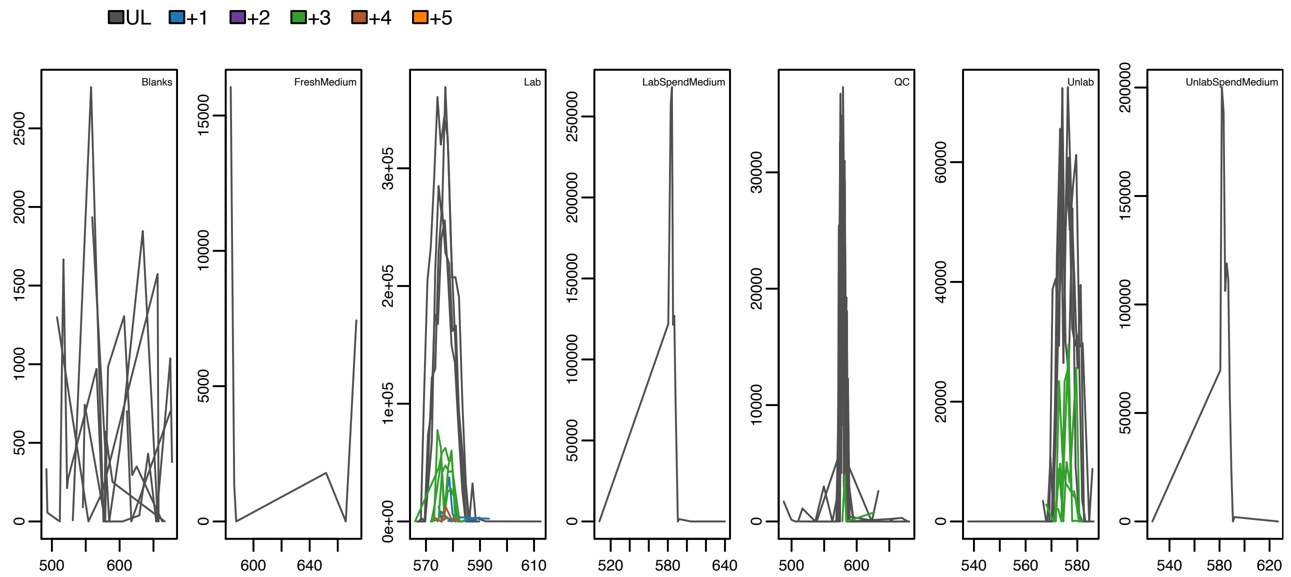

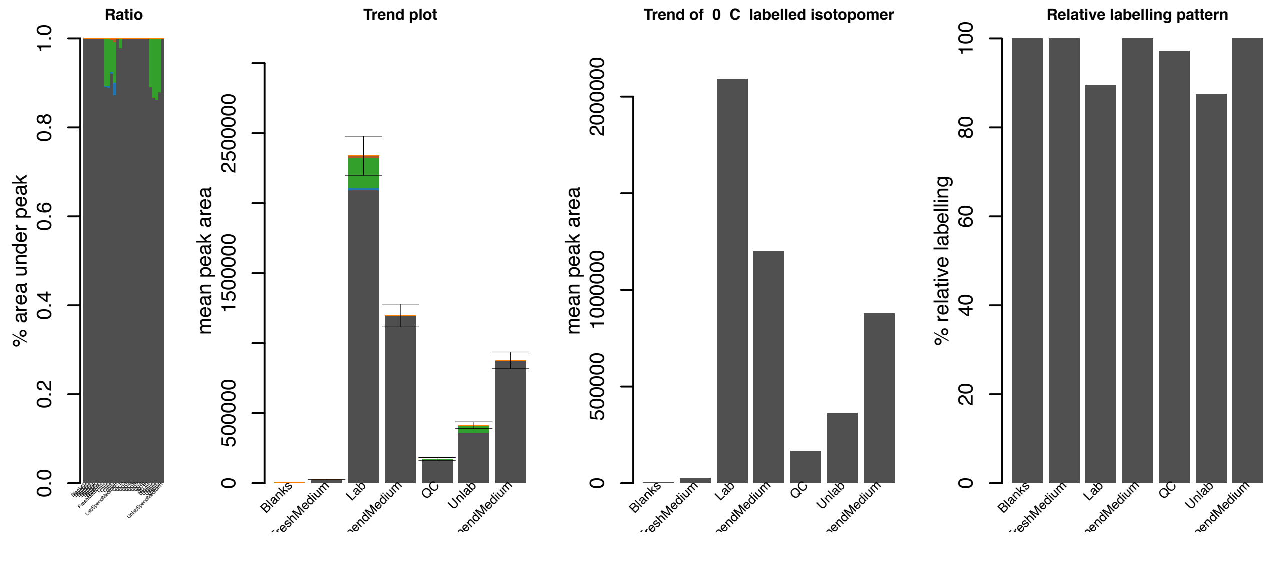

Pantothenate

Formula: $C9H17NO5$ Mass: 219.111 Std.RT: 487.2 Ion: NEG

G1

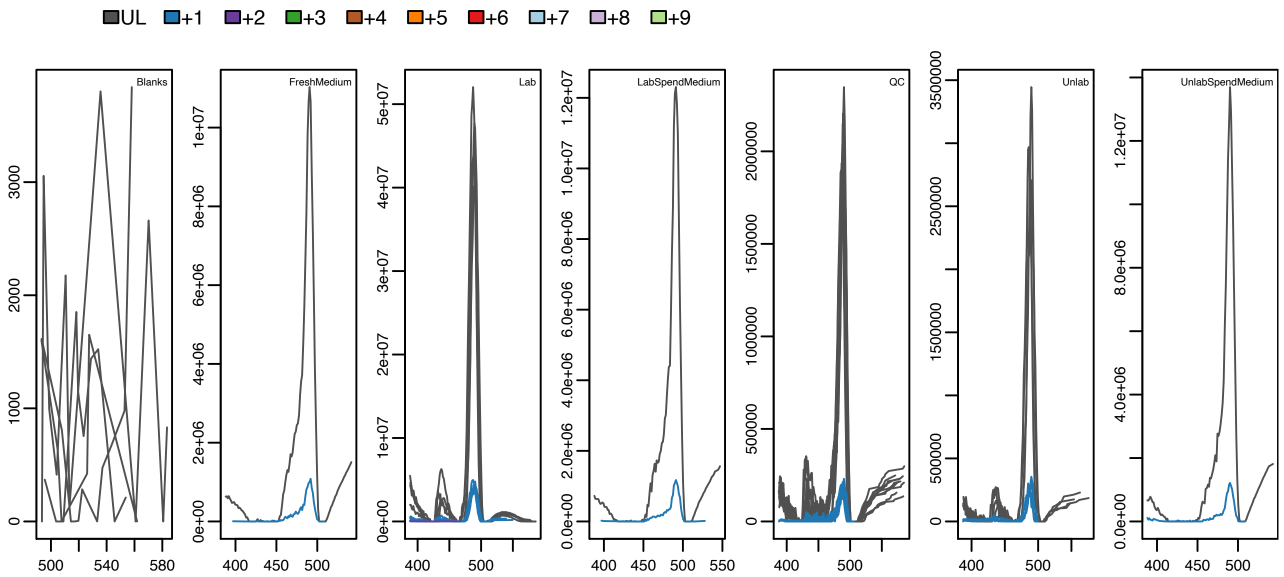

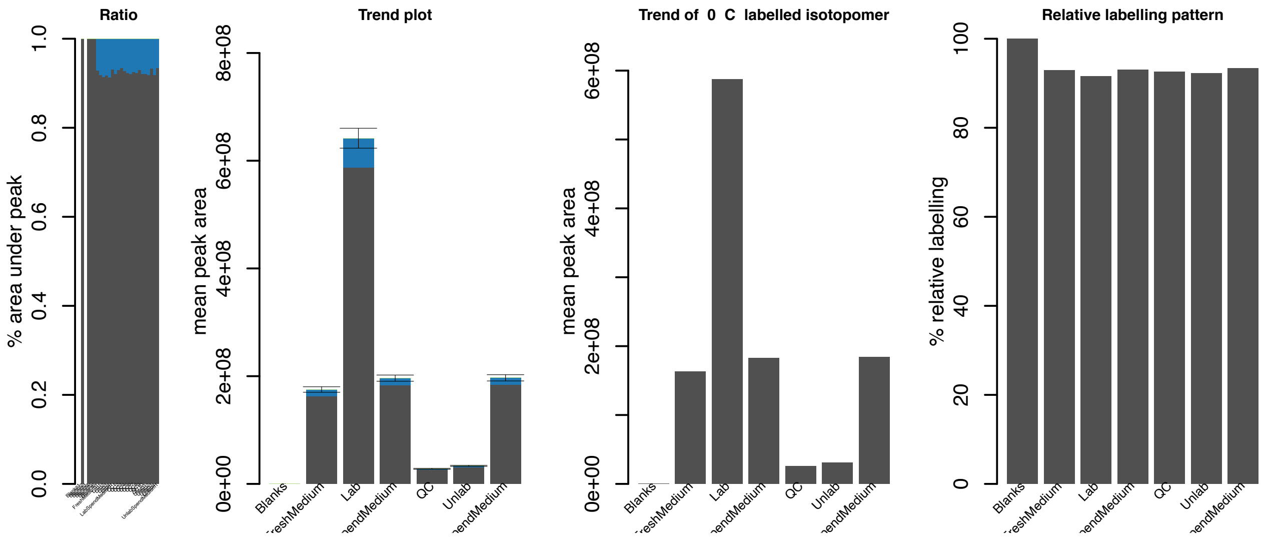

Phenylpyruvate

Formula: C9H8O3 Mass: 164.047 Std.RT: 274.2 Ion: NEG

G1

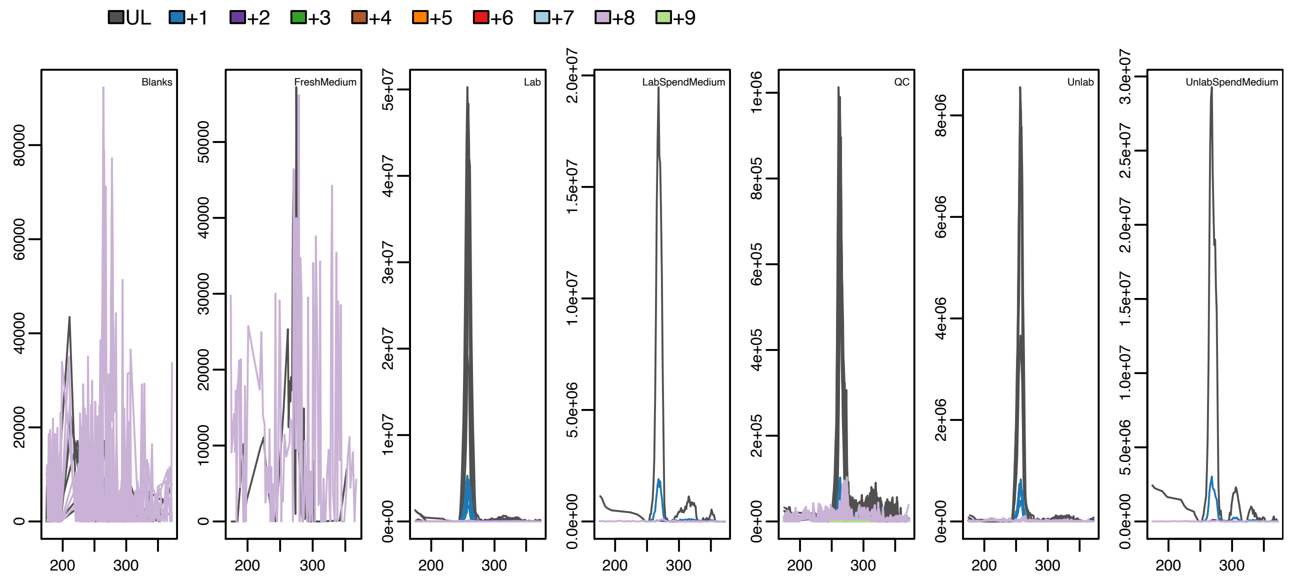

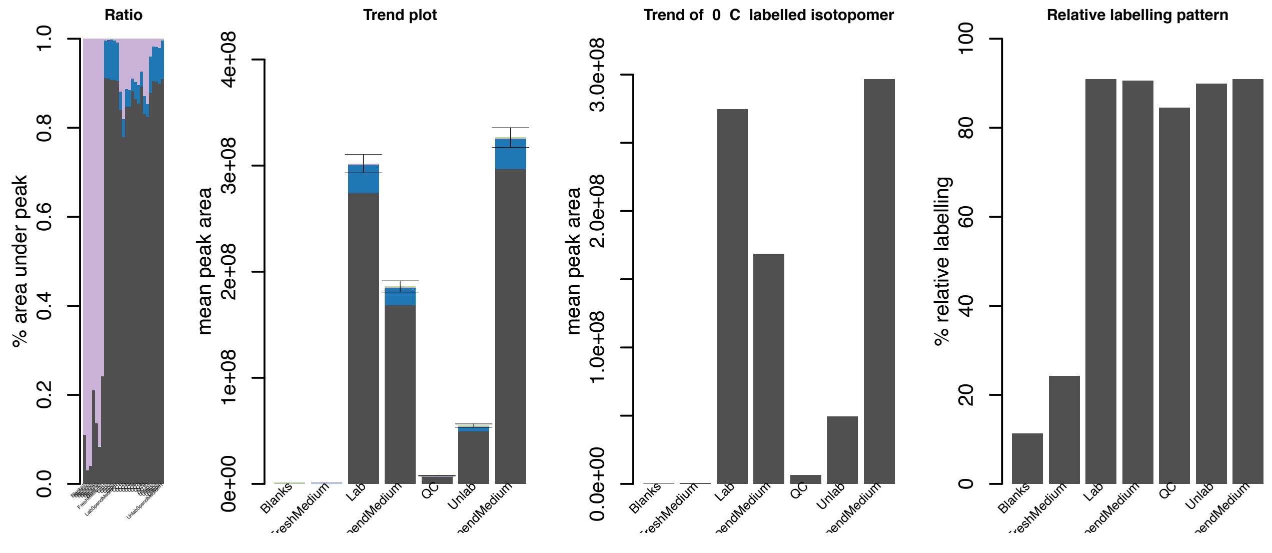

Phosphoenolpyruvate

Formula: C3H5O6P   Mass: 167.982 Std.RT: 811.8 Ion: NEG

G1

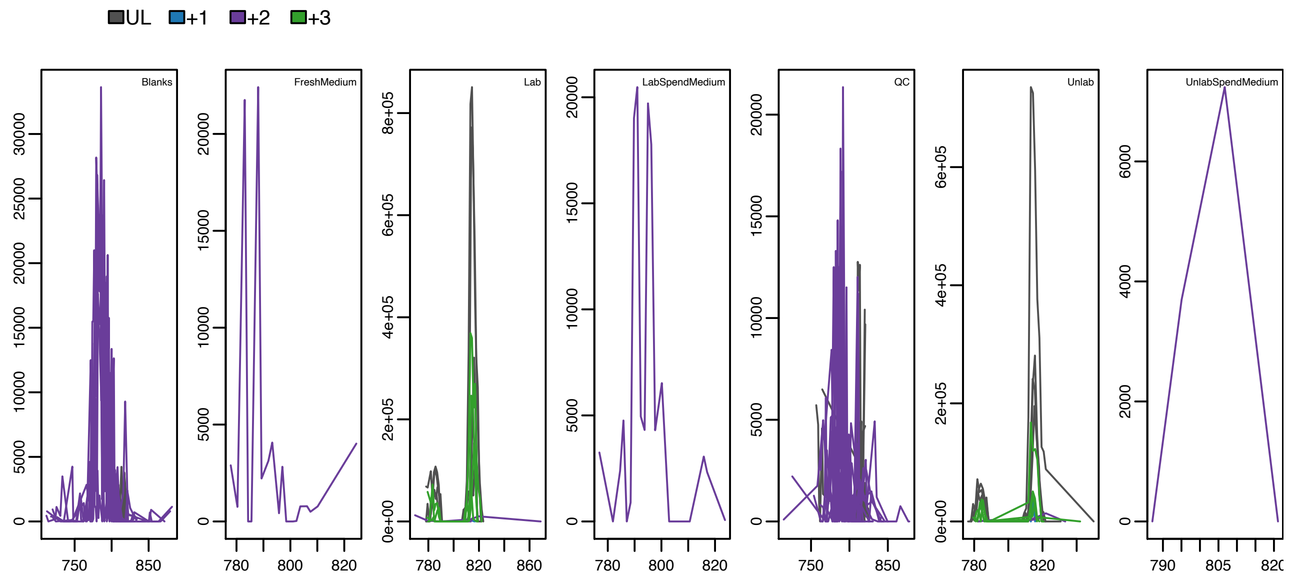

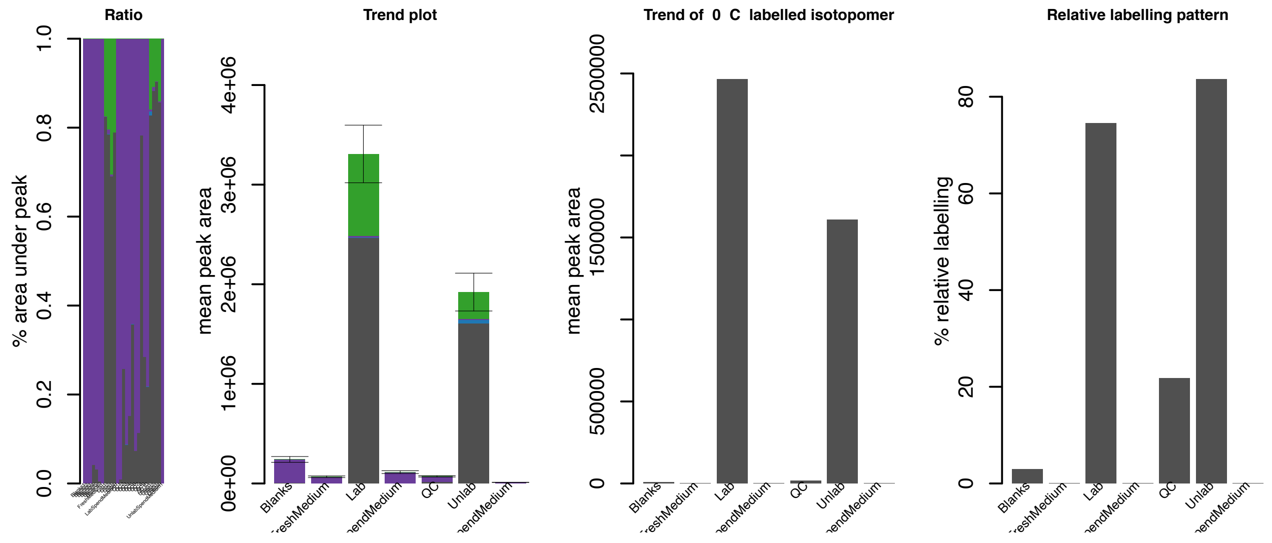

Pyruvate

Formula: C3H4O3   Mass: 88.016 Std.RT: 490.2 Ion: NEG

G1

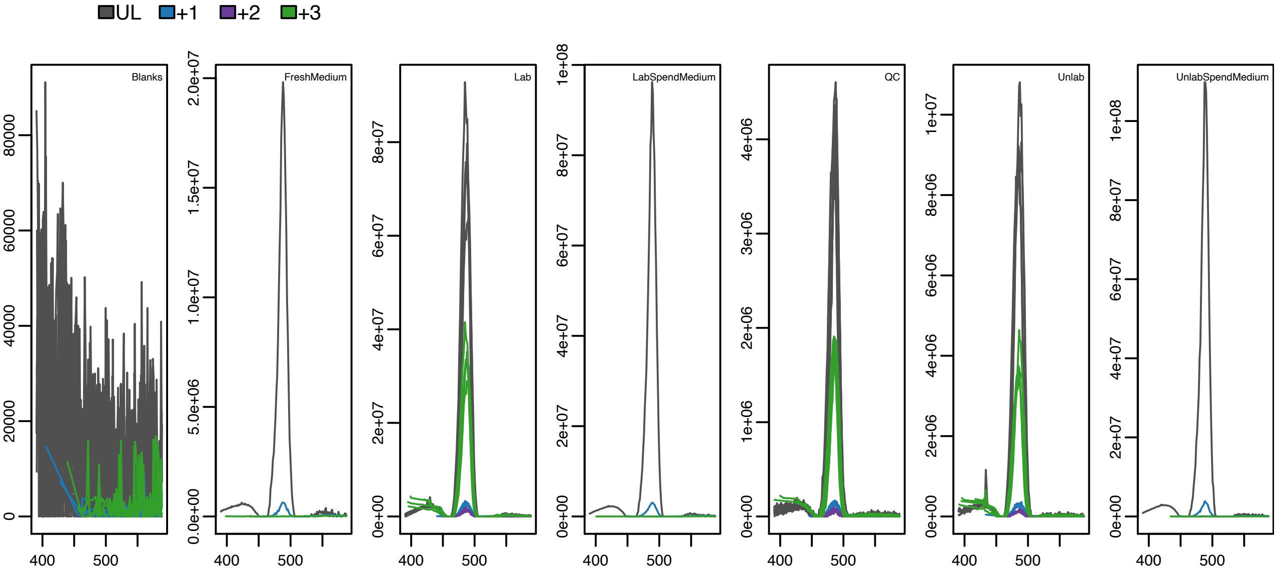

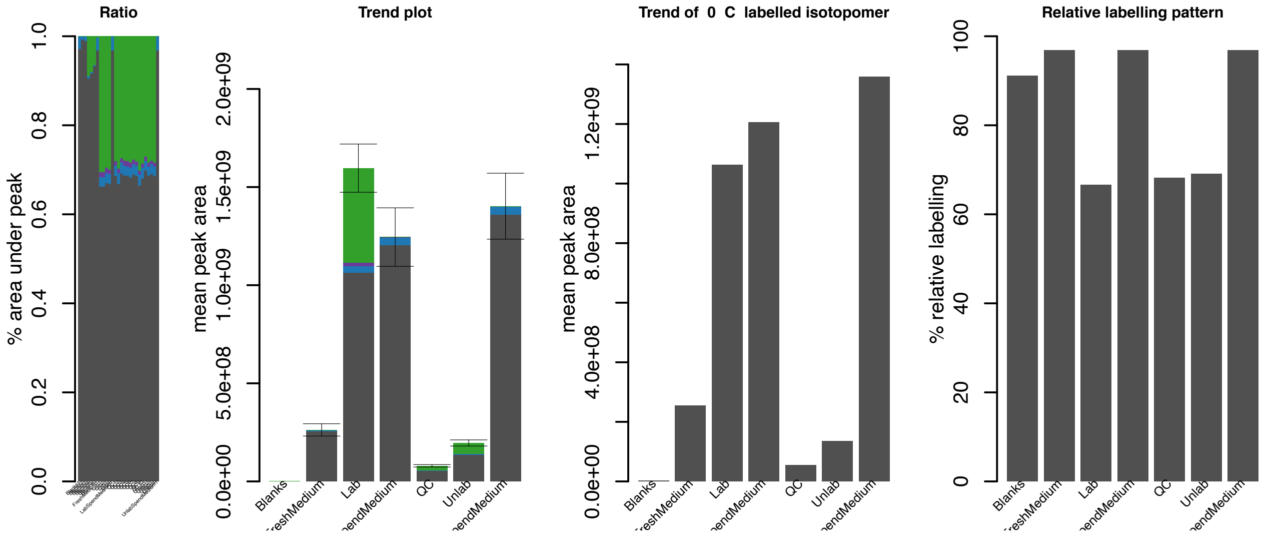

Succinate

Formula: C4H6O4 Mass: 118.027 Std.RT: 744.6 Ion: NEG

G1

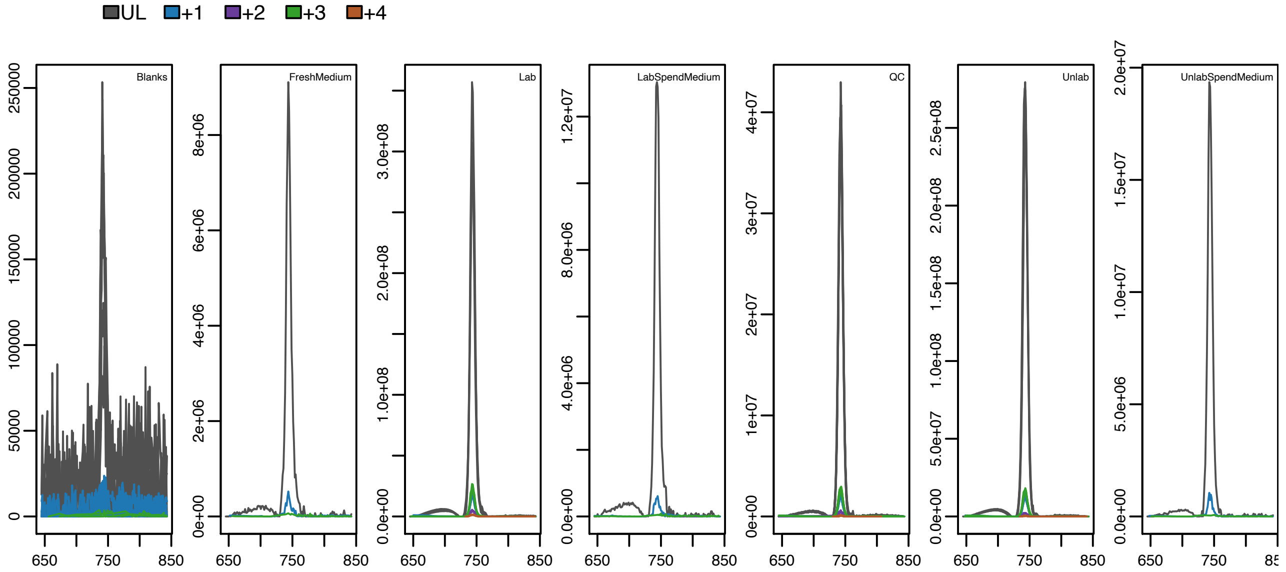

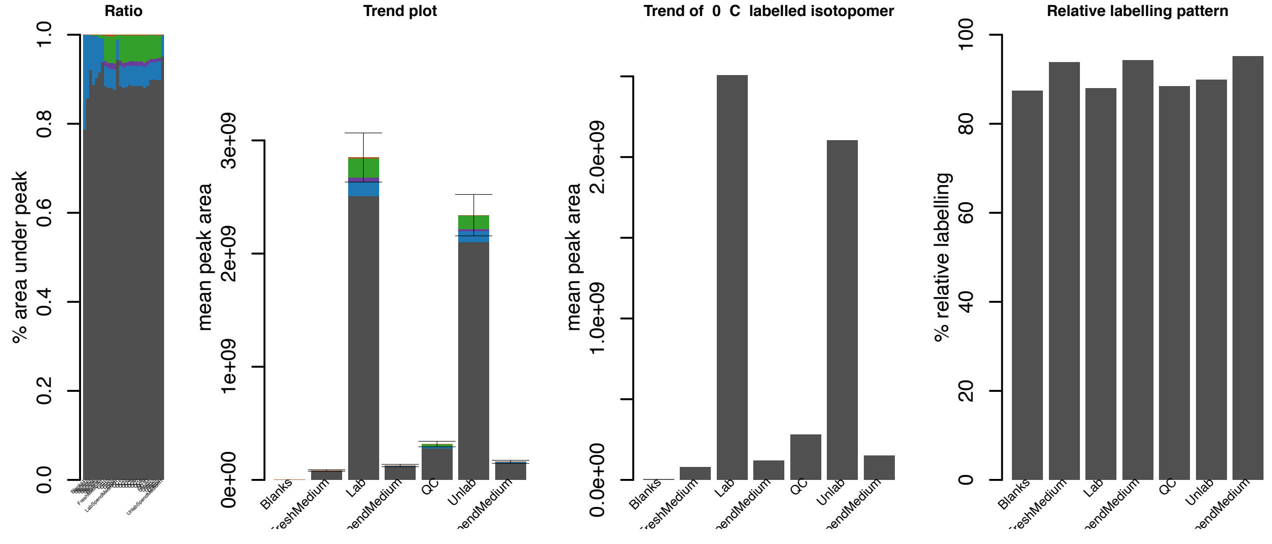

UMP

Formula: C9H13N2O9P Mass: 324.036 Std.RT: 694.8 Ion: NEG

G1

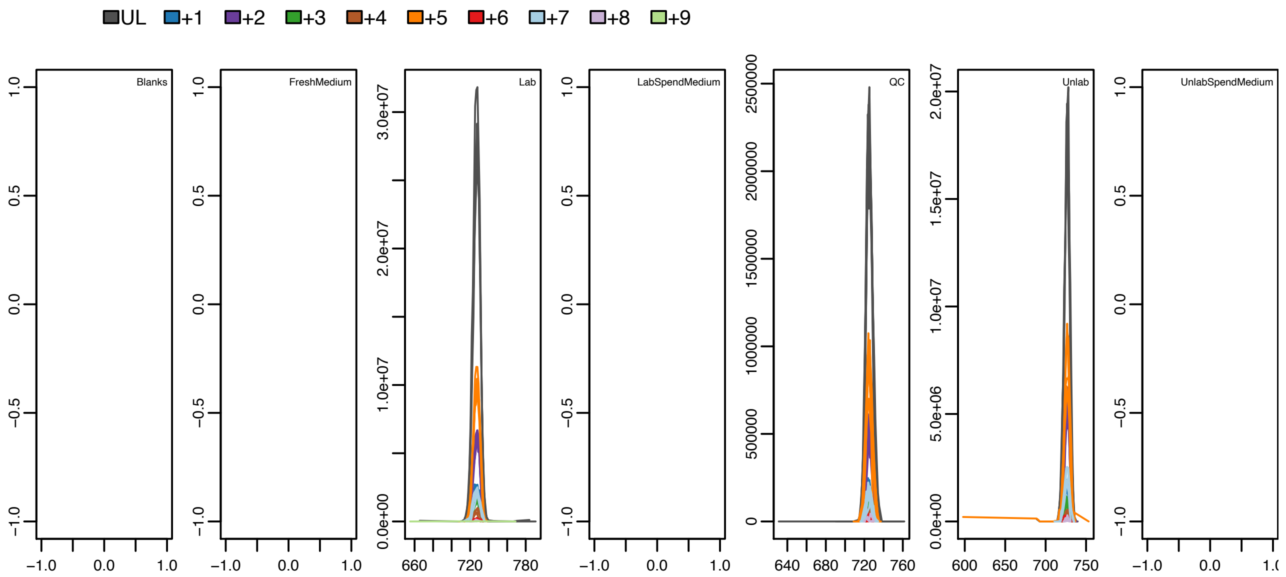

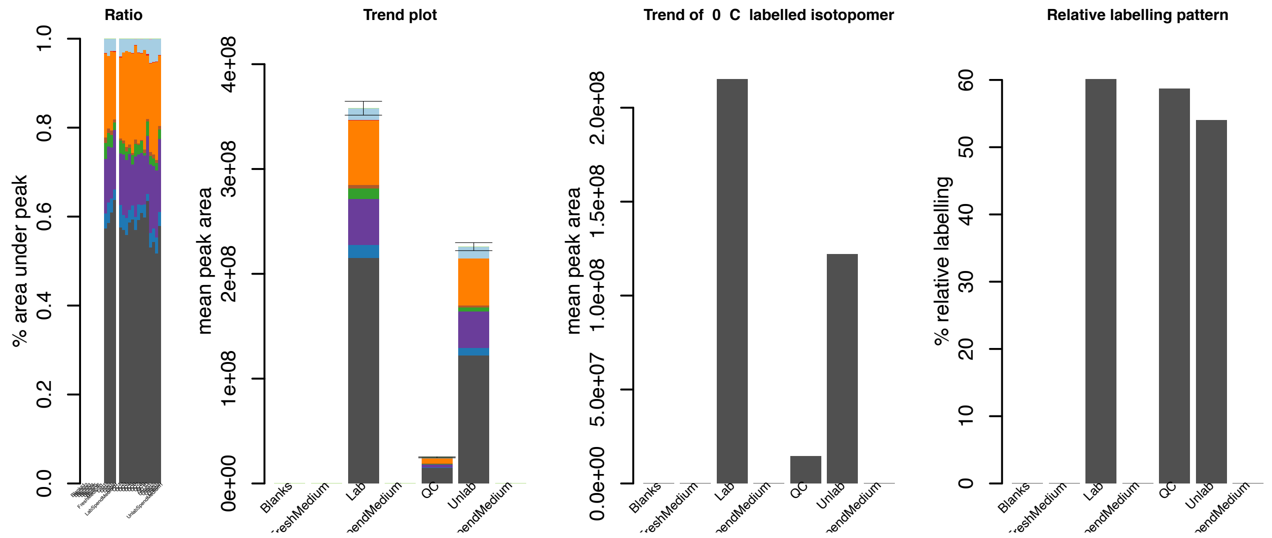

Uracil

G1

Formula: C4H4N2O2   Mass: 112.027 Std.RT: 483.6 Ion: NEG

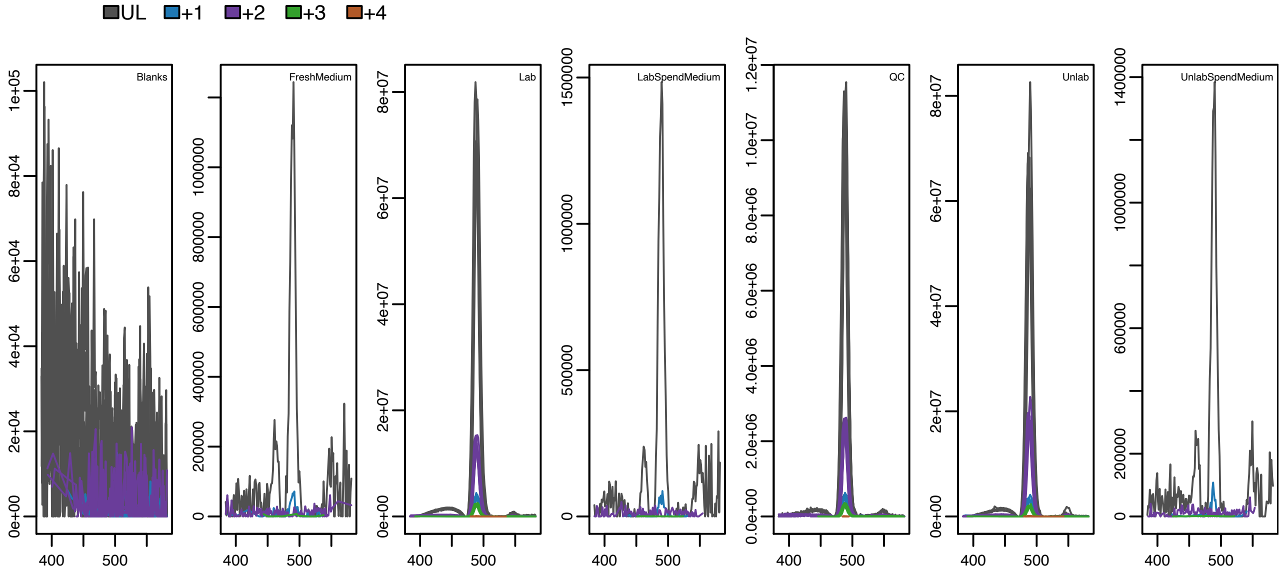

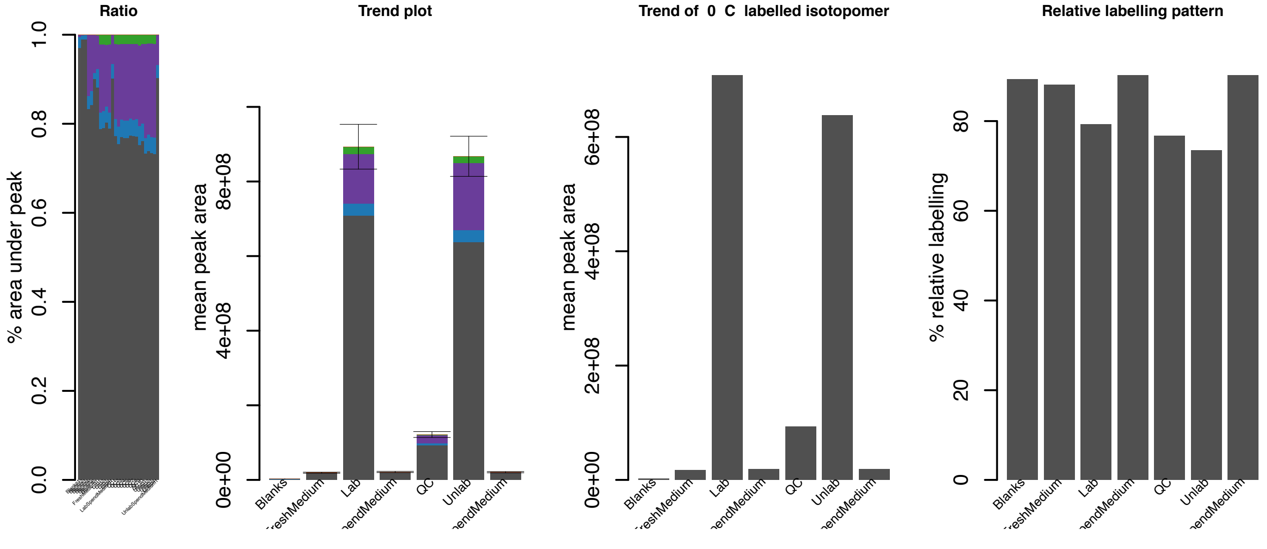

Uridine

Formula: C9H12N2O6 Mass: 244.07 Std.RT: 549 Ion: NEG

G1

UL +1 +2 +3 +4 +5 +6 +7 +8 +9

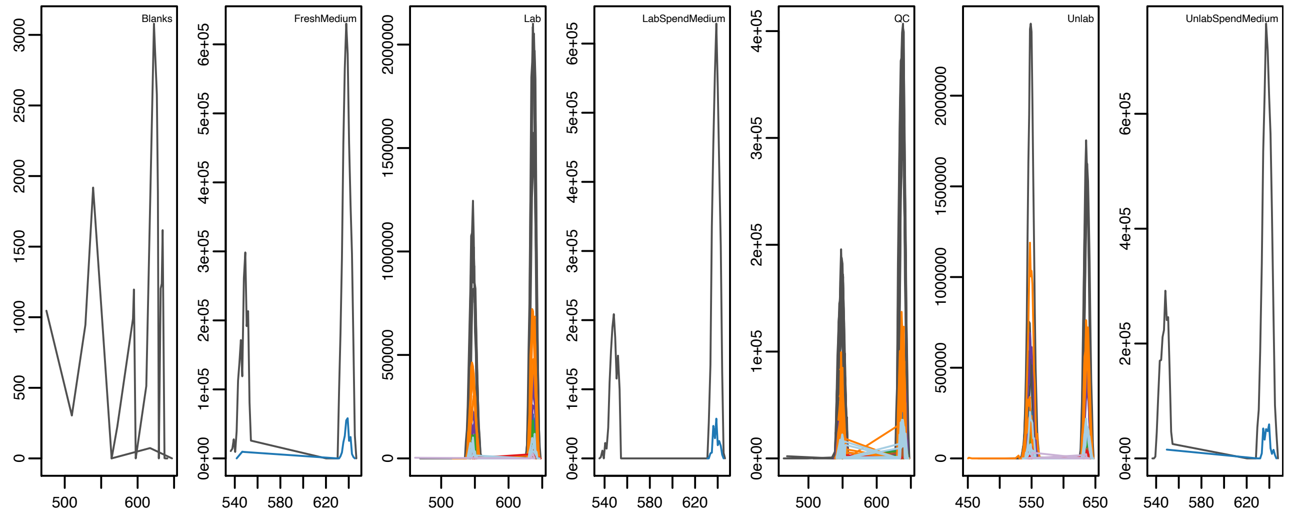

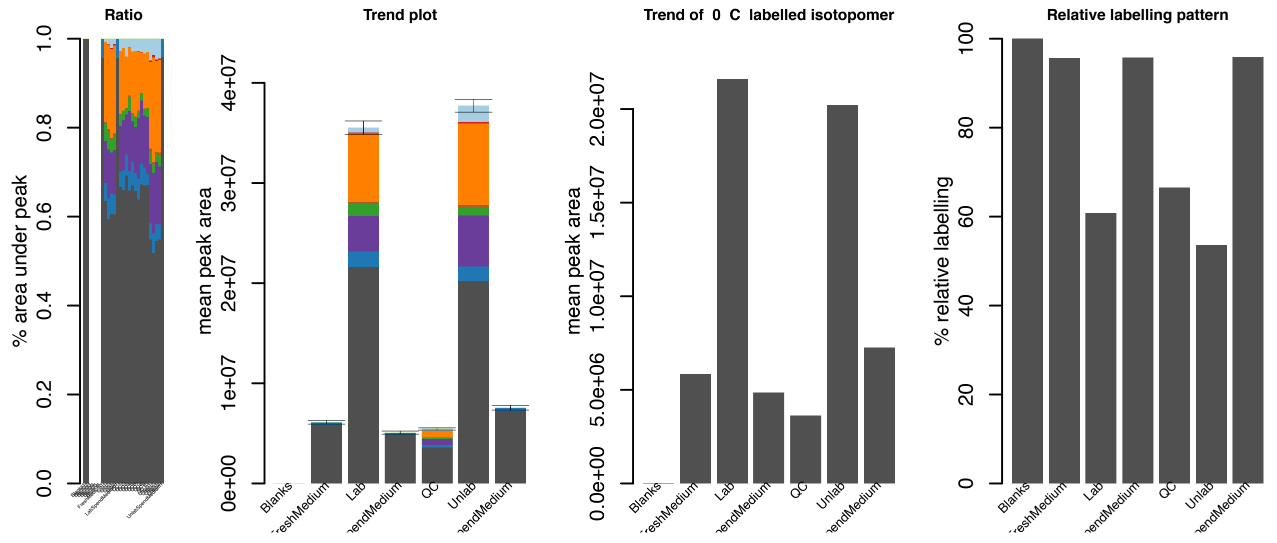

Xanthine

G1

Formula: C5H4N4O2 Mass: 152.033 Std.RT: 630.6 Ion: NEG

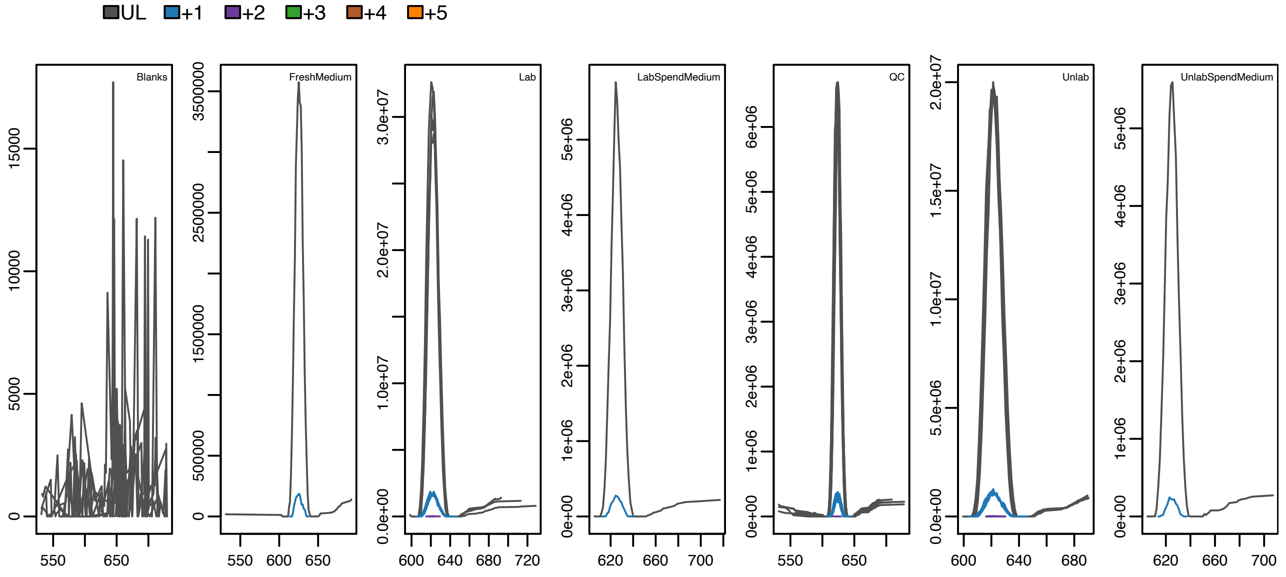

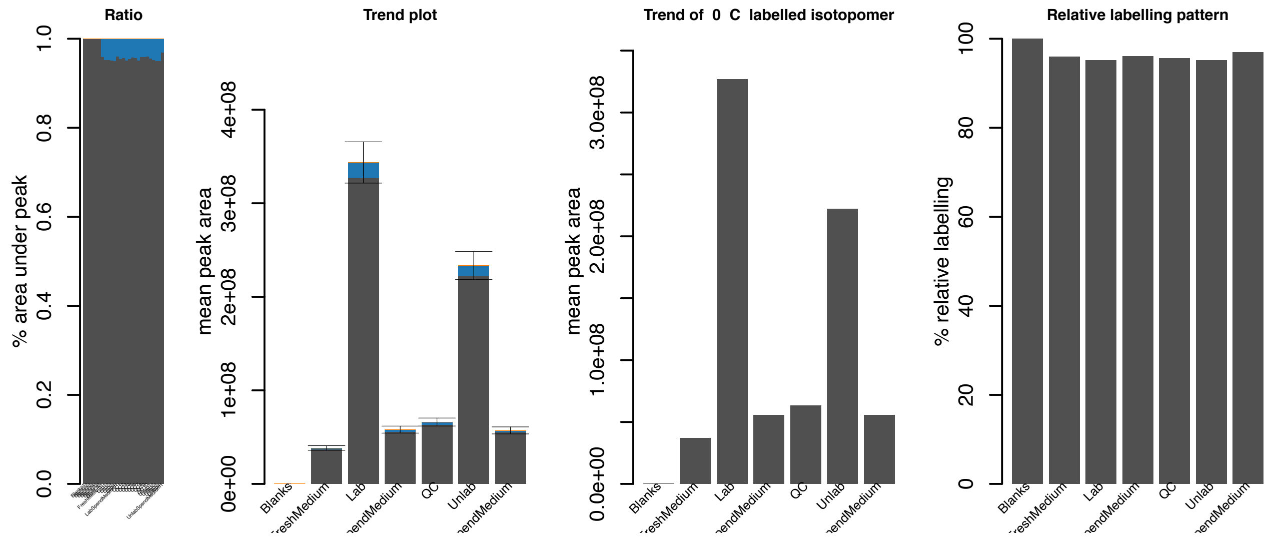

sn−Glycerol 3−phosphate

Formula: C3H9O6P   Mass: 172.014 Std.RT: 720.6 Ion: NEG

G1

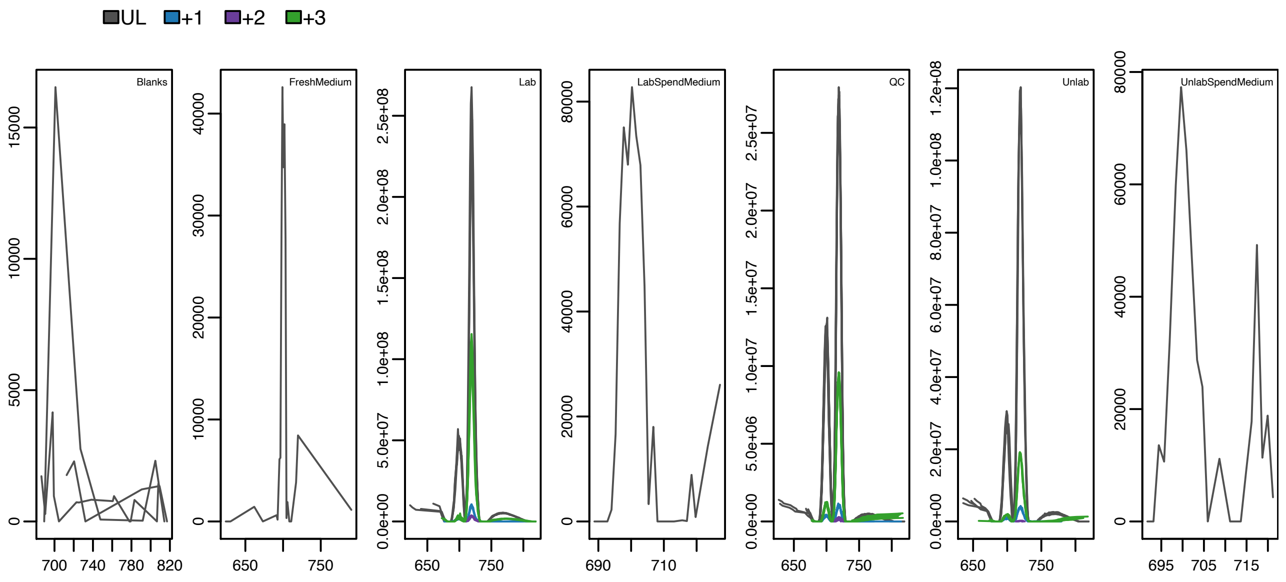

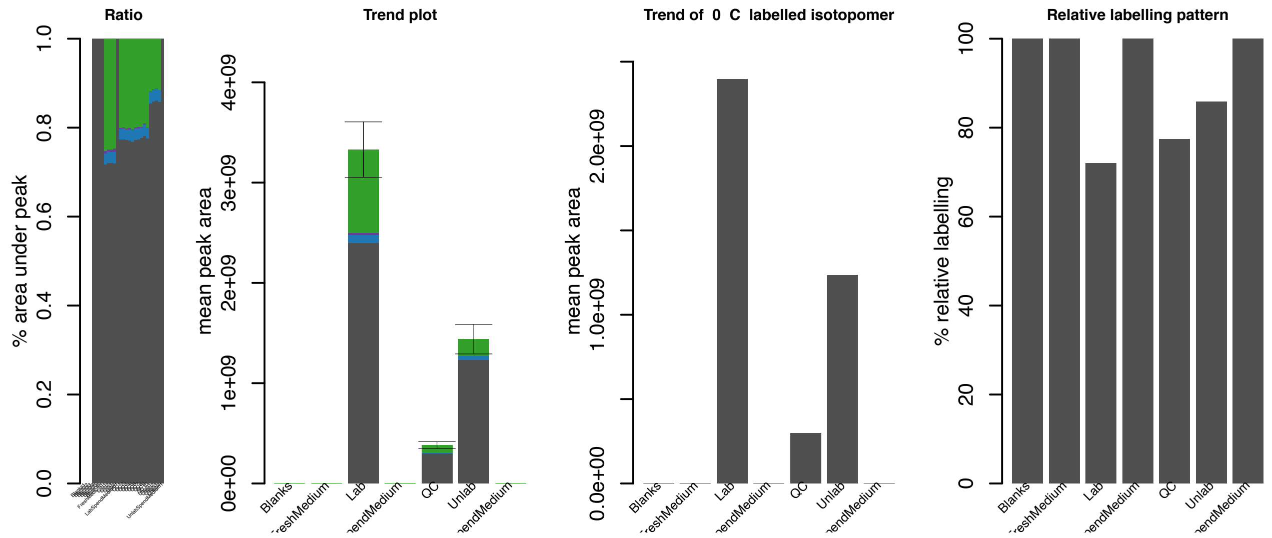

sn−glycero−3−Phosphocholine

G1

Formula: C8H20NO6P Mass: 257.103 Std.RT: 700.8 Ion: NEG

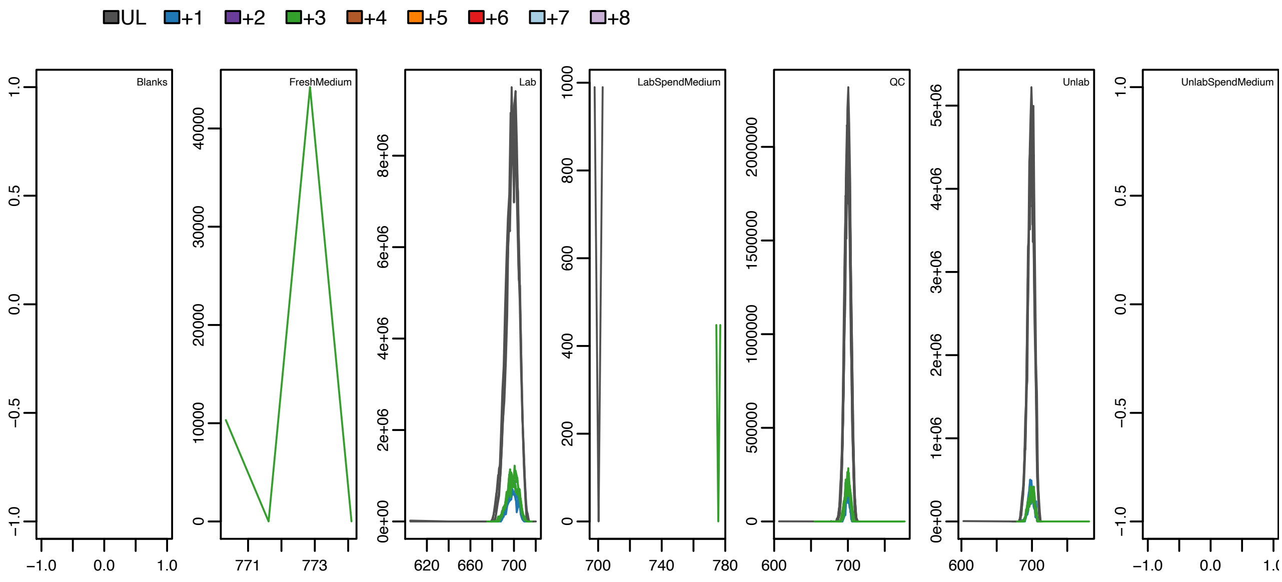

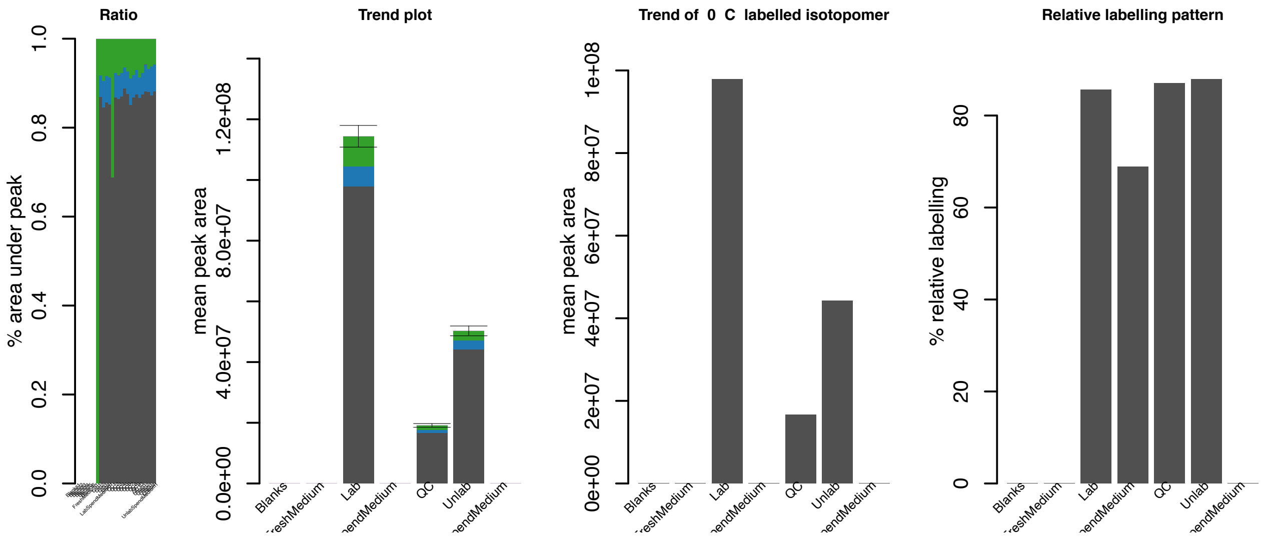

thymine

Formula: C5H6N2O2 Mass: 126.043 Std.RT: 418.2 Ion: NEG

G1

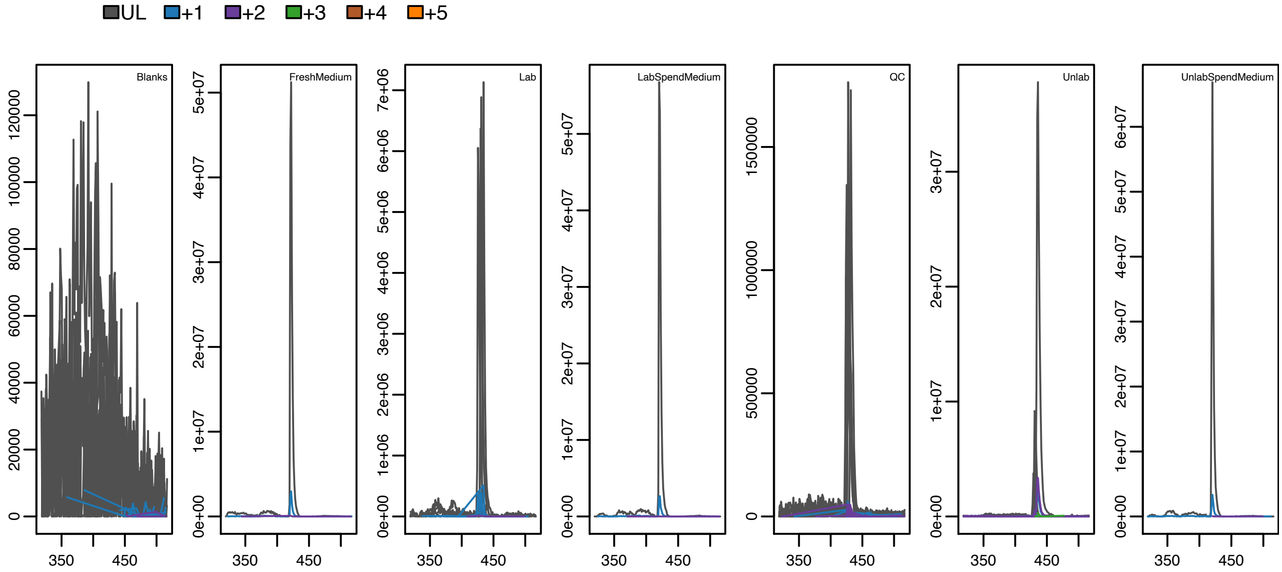

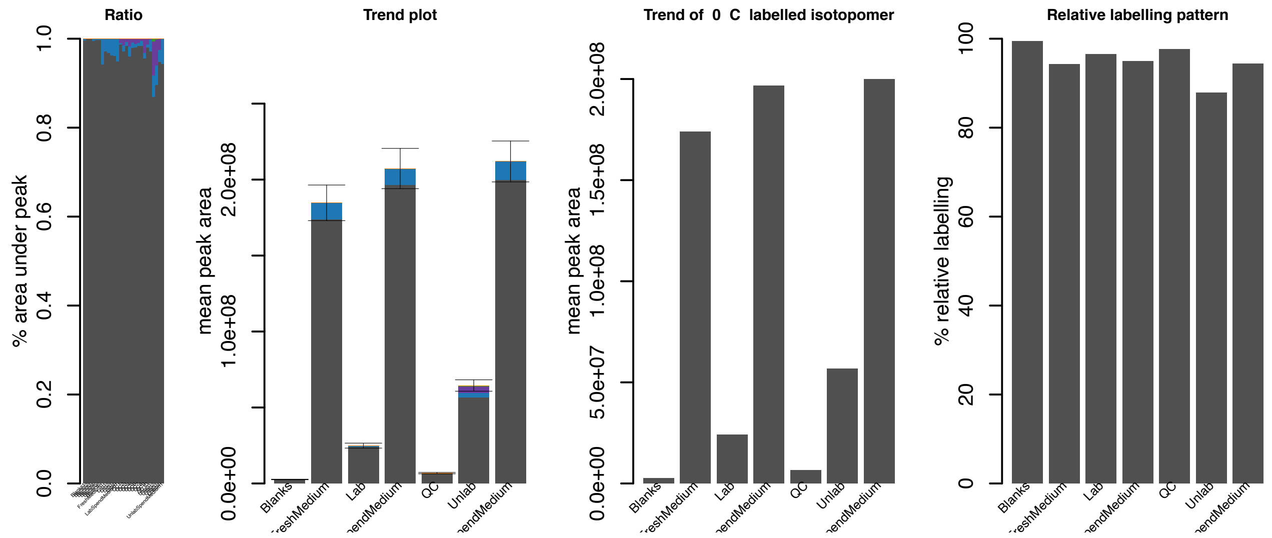

3'_5'−Cyclic AMP

Formula: C10H12N5O6P Mass: 329.053 Std.RT: 526.8 Ion: POS

G1

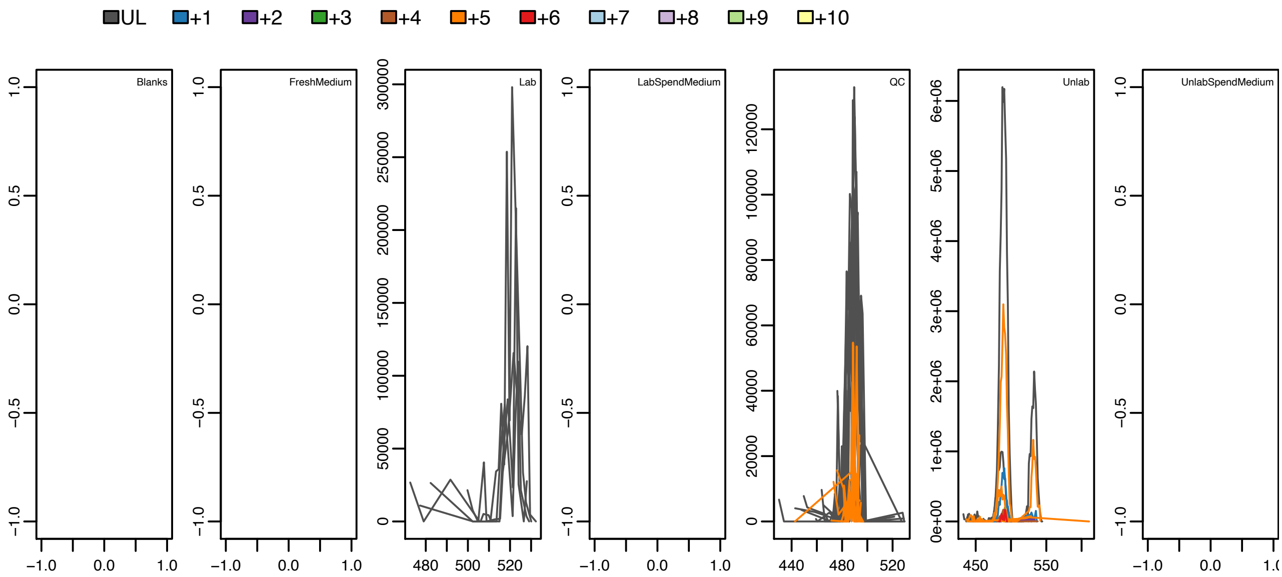

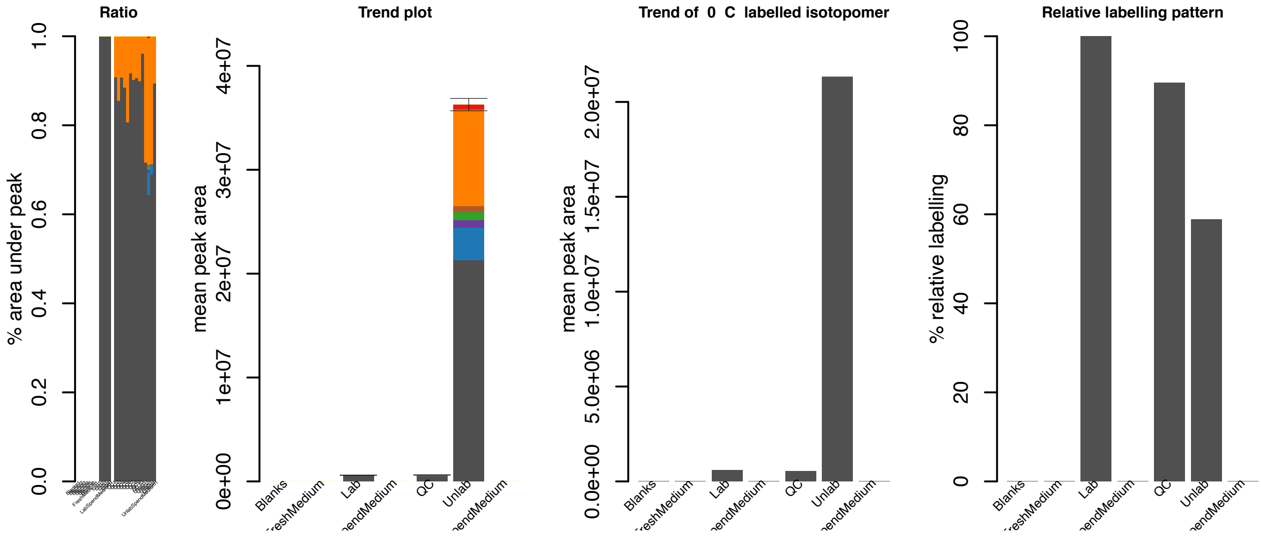

5'–Methylthioadenosine

Formula: C11H15N5O3S Mass: 297.09 Std.RT: 363 Ion: POS

G1

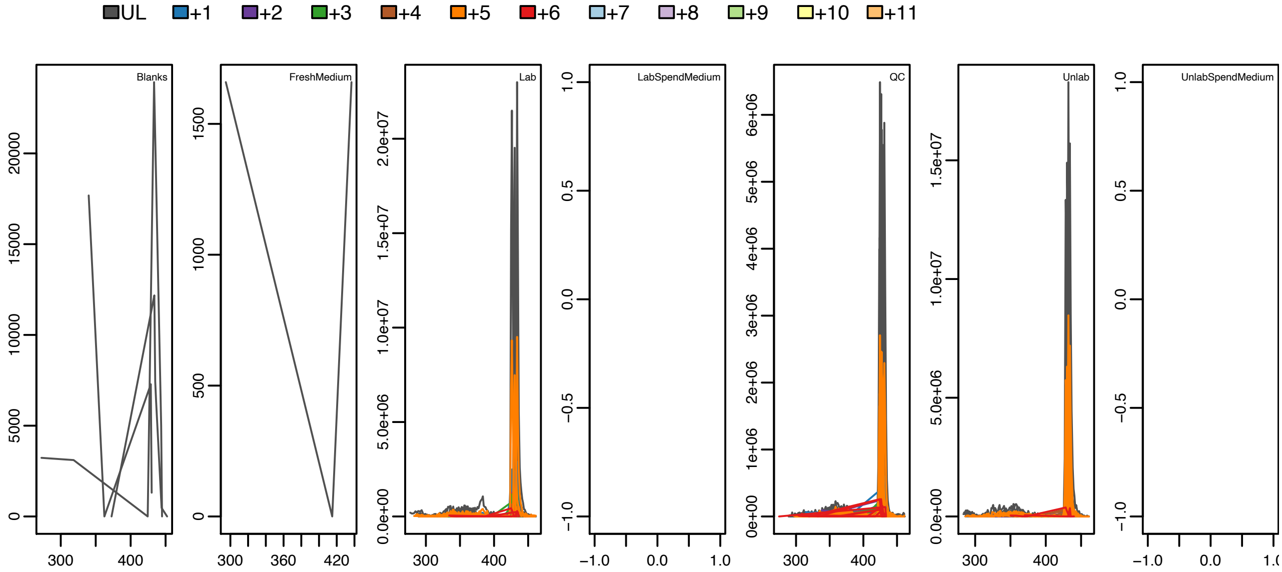

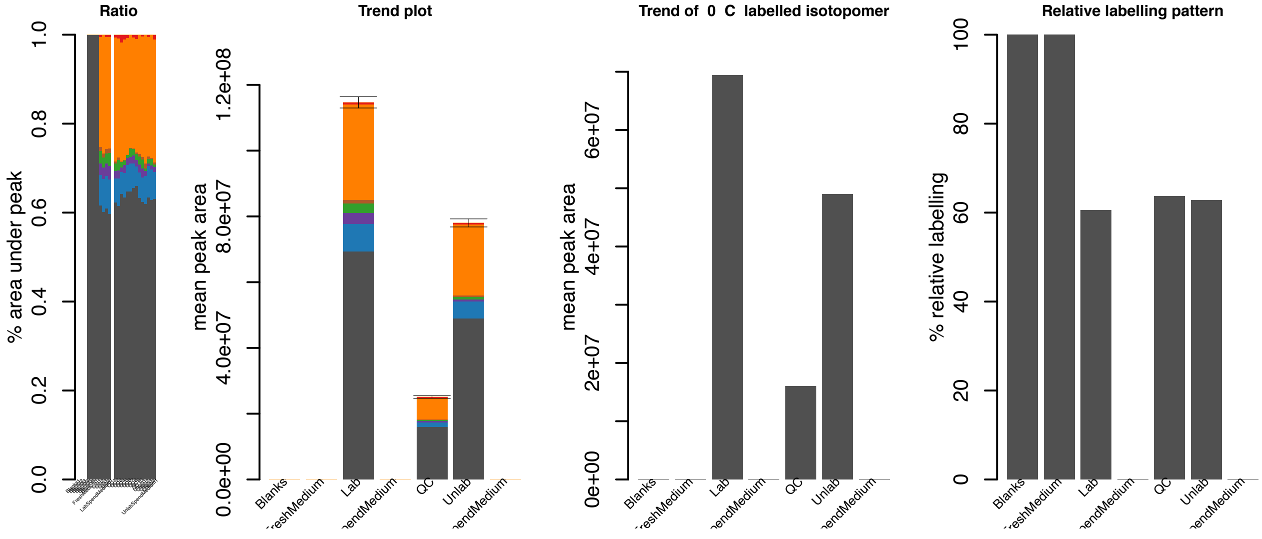

ADP

G1

Formula: C10H15N5O10P2 Mass: 427.029 Std.RT: 714 Ion: POS

UL +1 +2 +3 +4 +5 +6 +7 +8 +9 +10

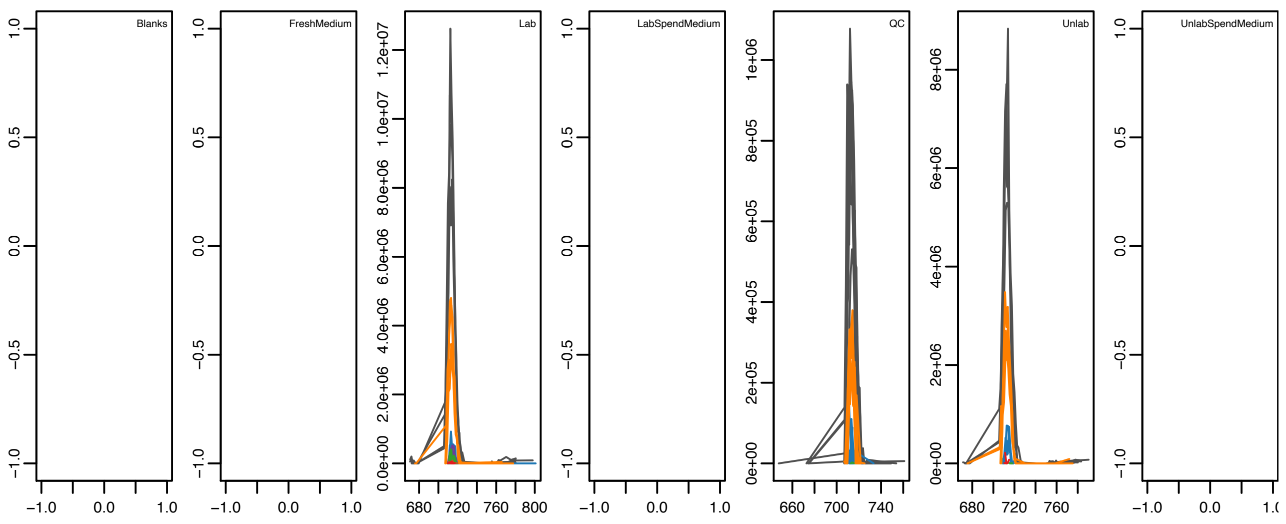

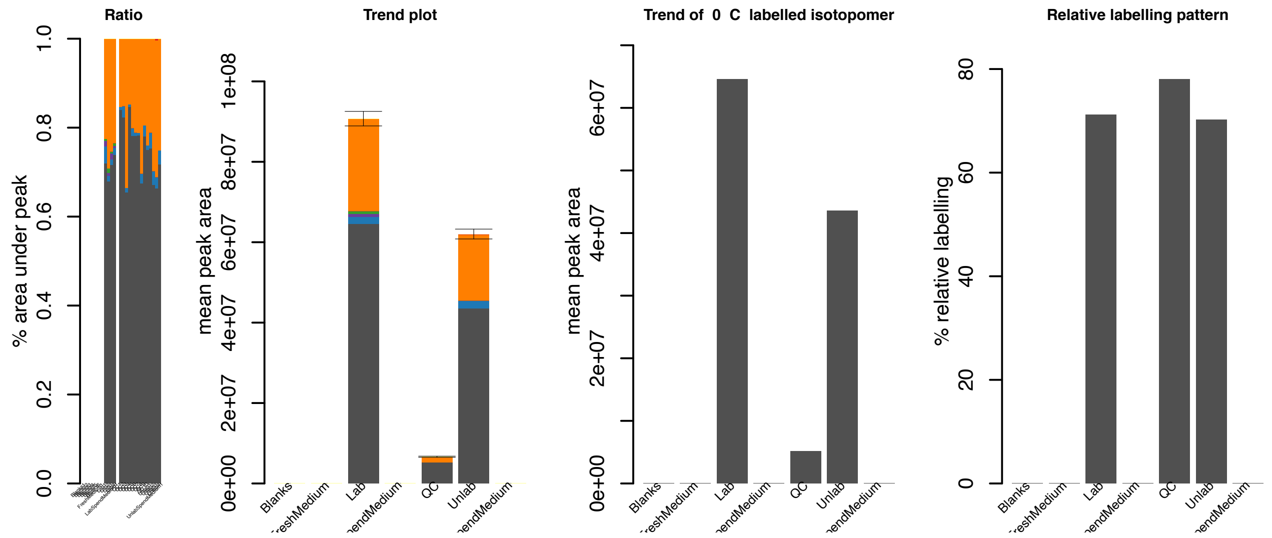

AMP

Formula: C10H14N5O7P Mass: 347.063 Std.RT: 670.8 Ion: POS

G1

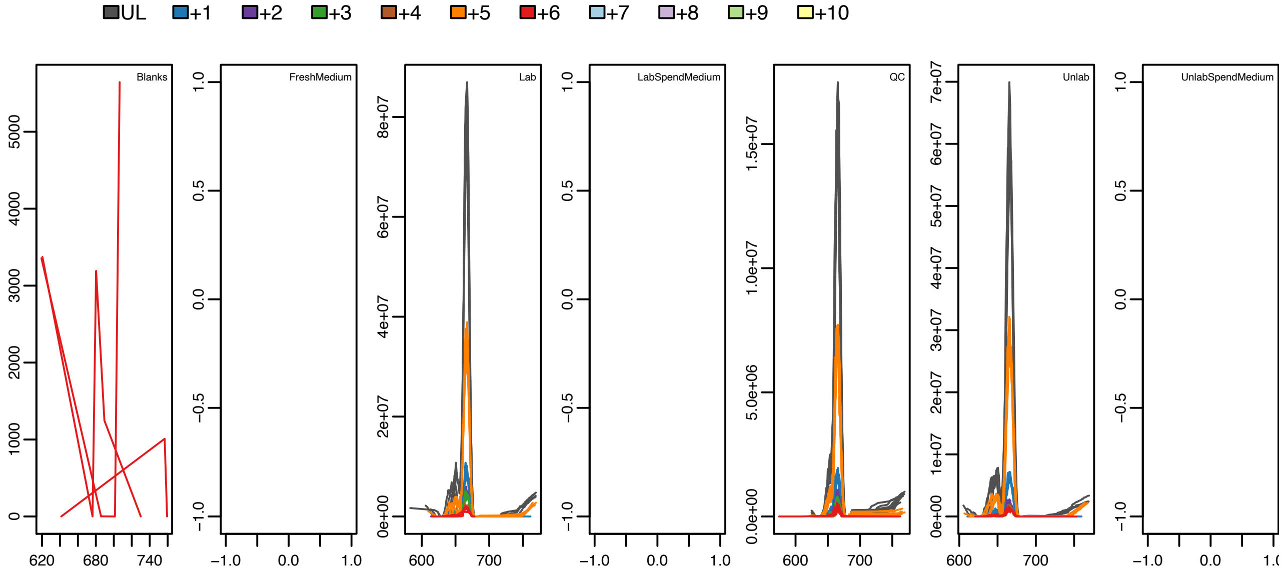

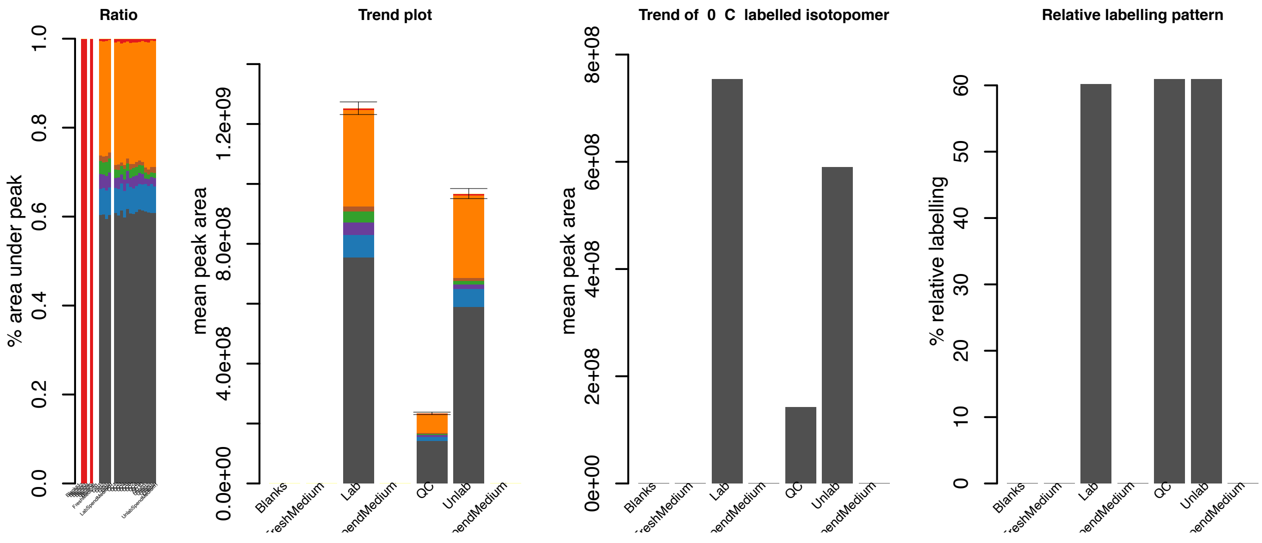

Acetylcholine

Formula: $C_7H_{15}NO_2$ Mass: 145.11 Std.RT: 823.2 Ion: POS

G1

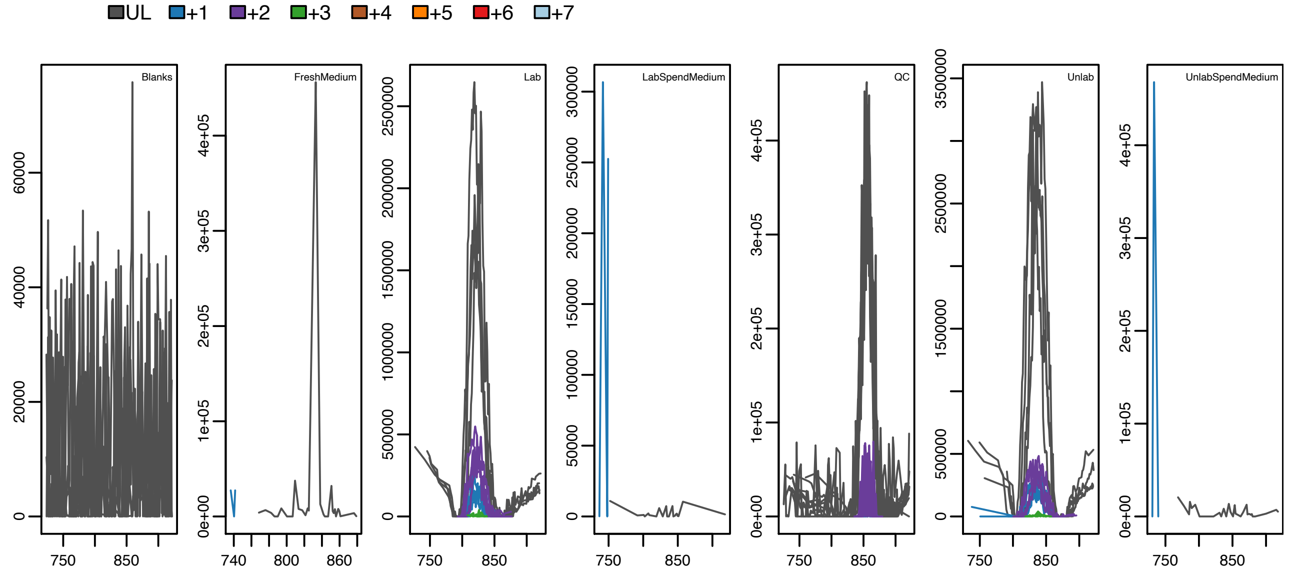

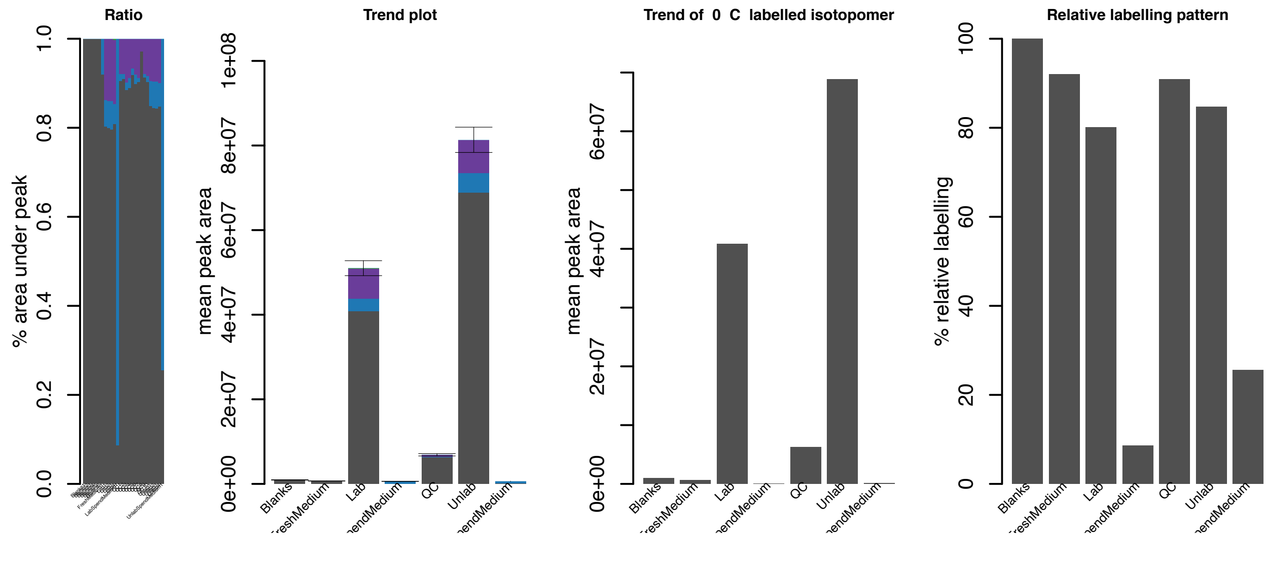

Adenine

Formula: C5H5N5 Mass: 135.054 Std.RT: 523.8 Ion: POS

G1

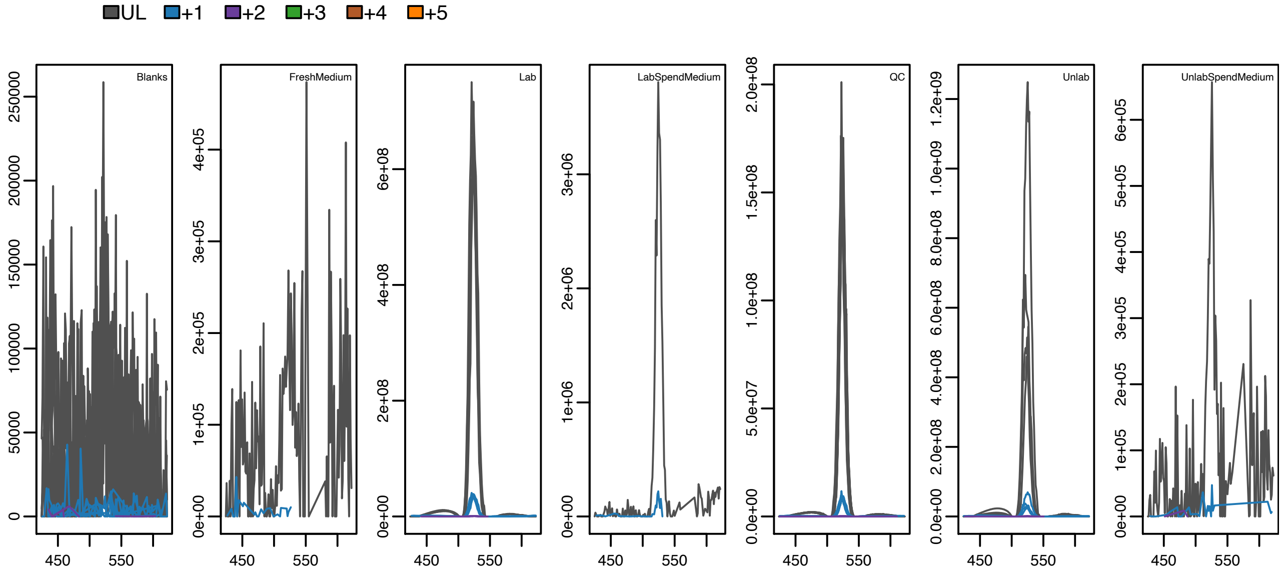

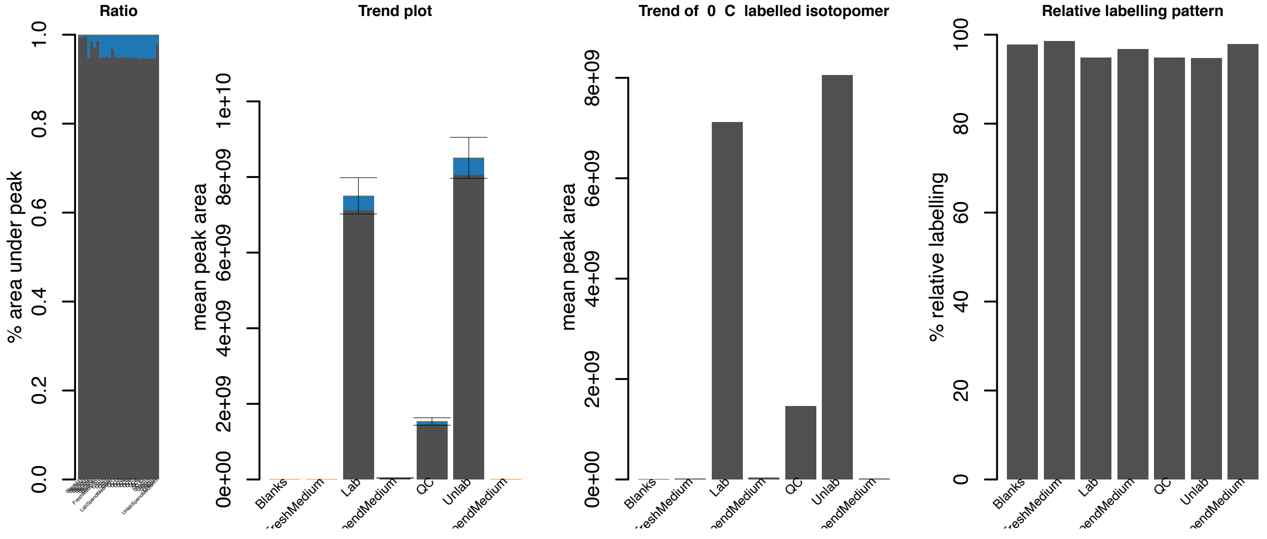

Adenosine

Formula: C10H13N5O4 Mass: 267.097 Std.RT: 494.4 Ion: POS

G1

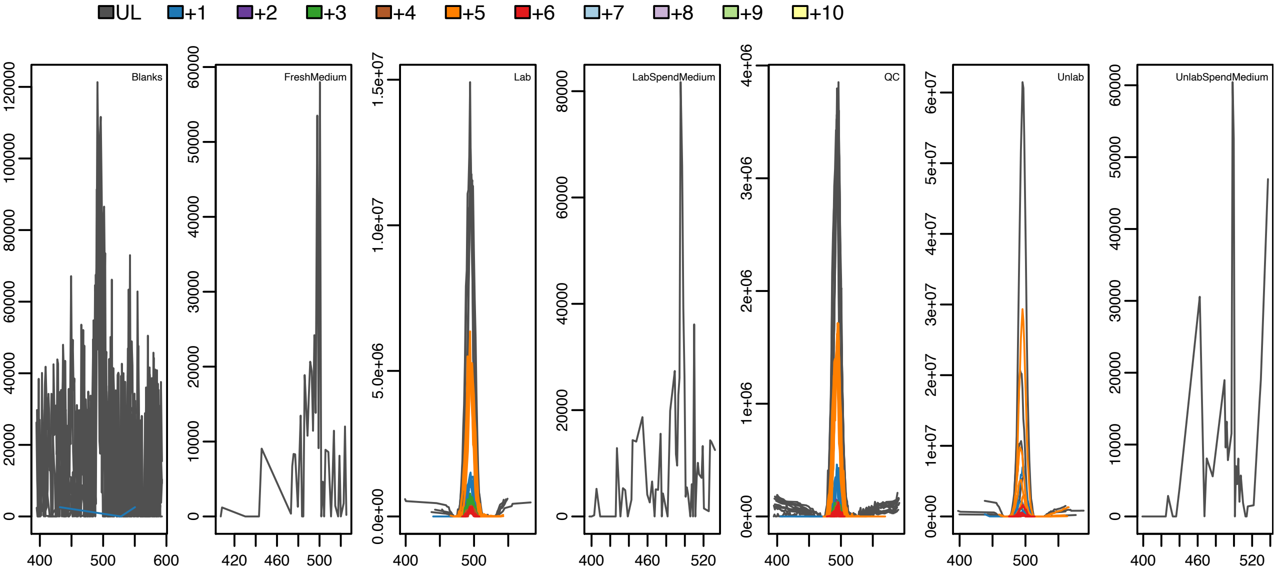

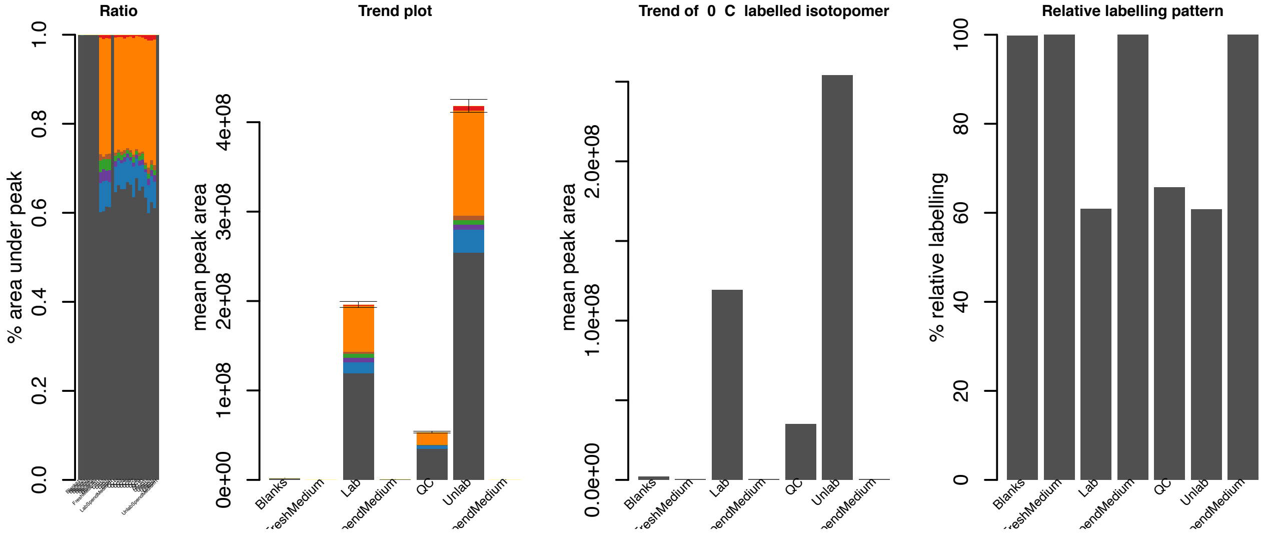

Adenosine 2'_5'−bisphosphate

Formula: C10H15N5O10P2 Mass: 427.029 Std.RT: 761.4 Ion: POS

G1

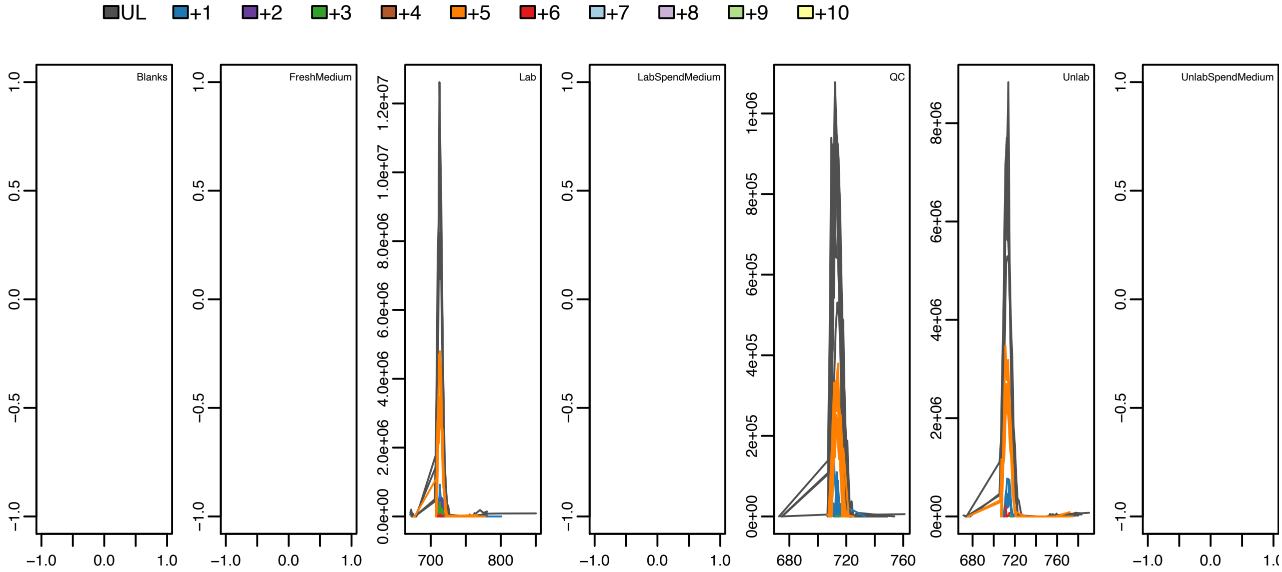

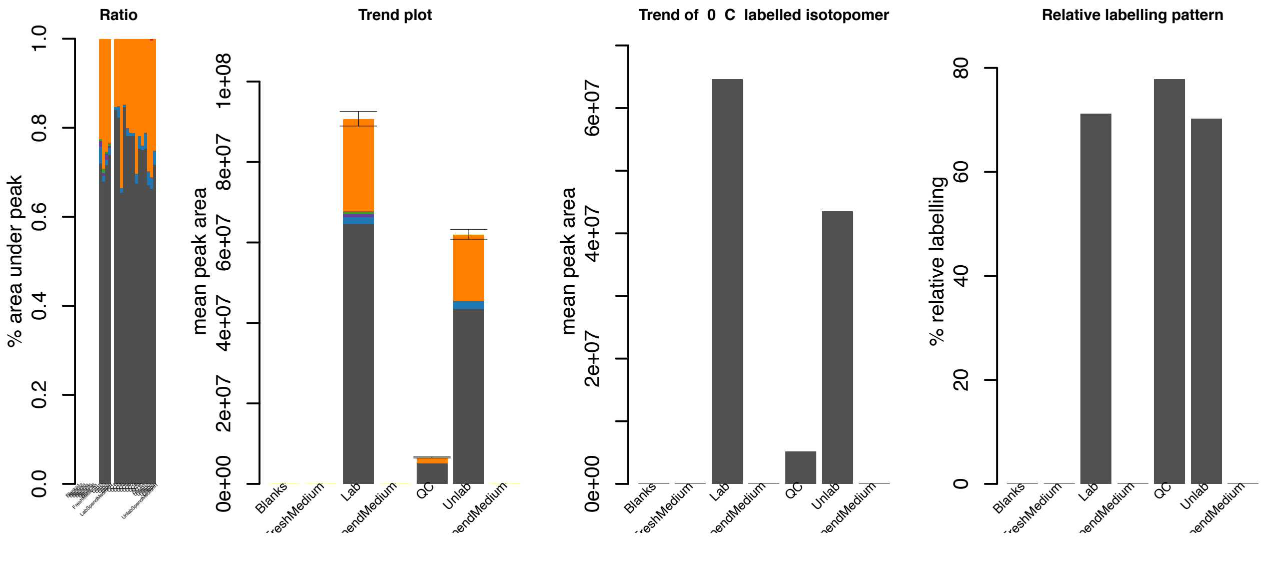

Choline

Formula: C5H13NO Mass: 103.1 Std.RT: 1069.8 Ion: POS

G1

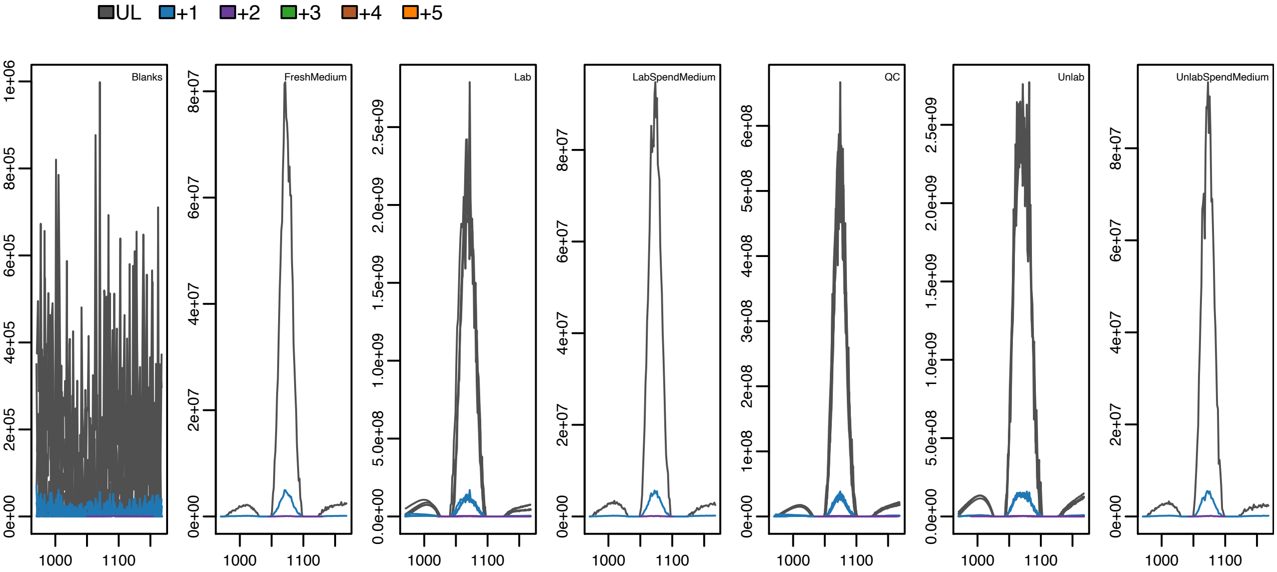

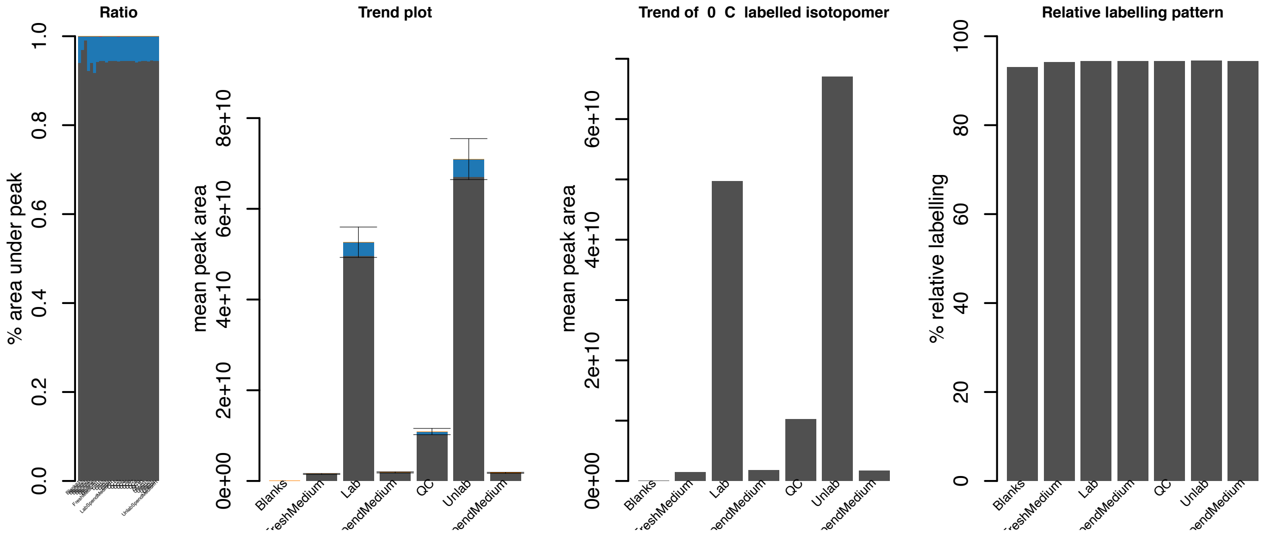

Choline phosphate

Formula: C5H14NO4P   Mass: 183.066 Std.RT: 706.2 Ion: POS

G1

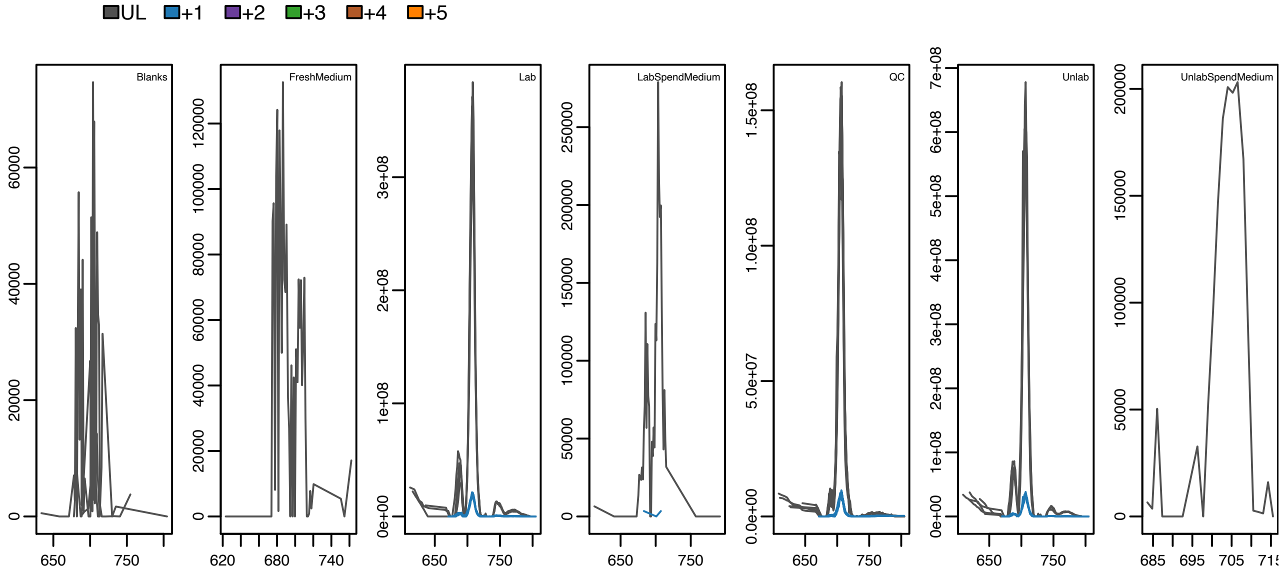

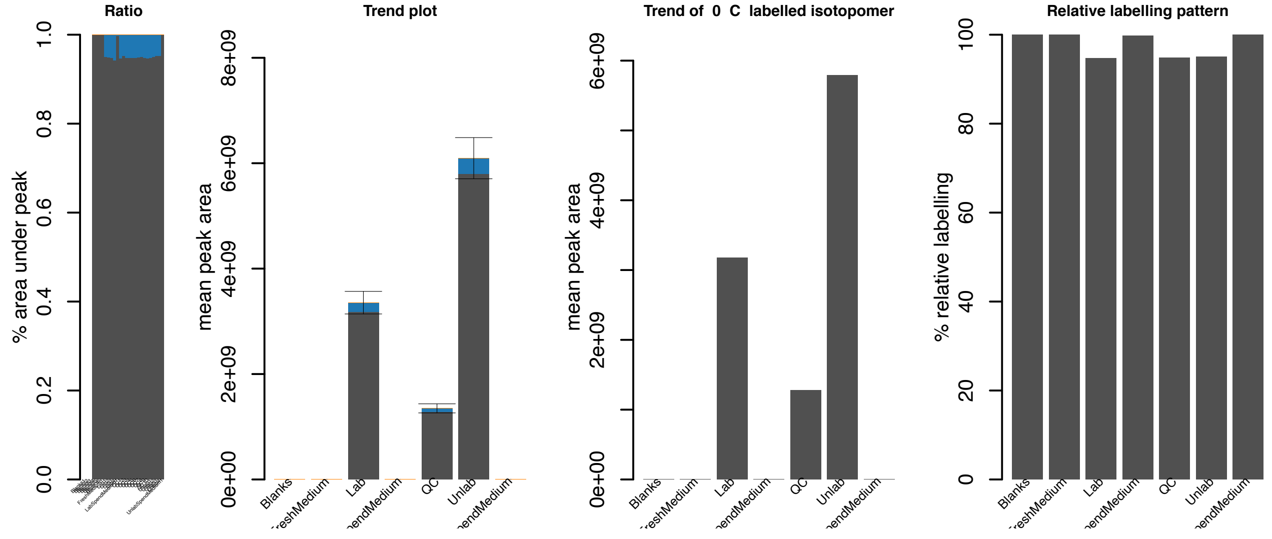

Cytidine

G1

Formula: C9H13N3O5 Mass: 243.086 Std.RT: 633.6 Ion: POS

UL +1 +2 +3 +4 +5 +6 +7 +8 +9

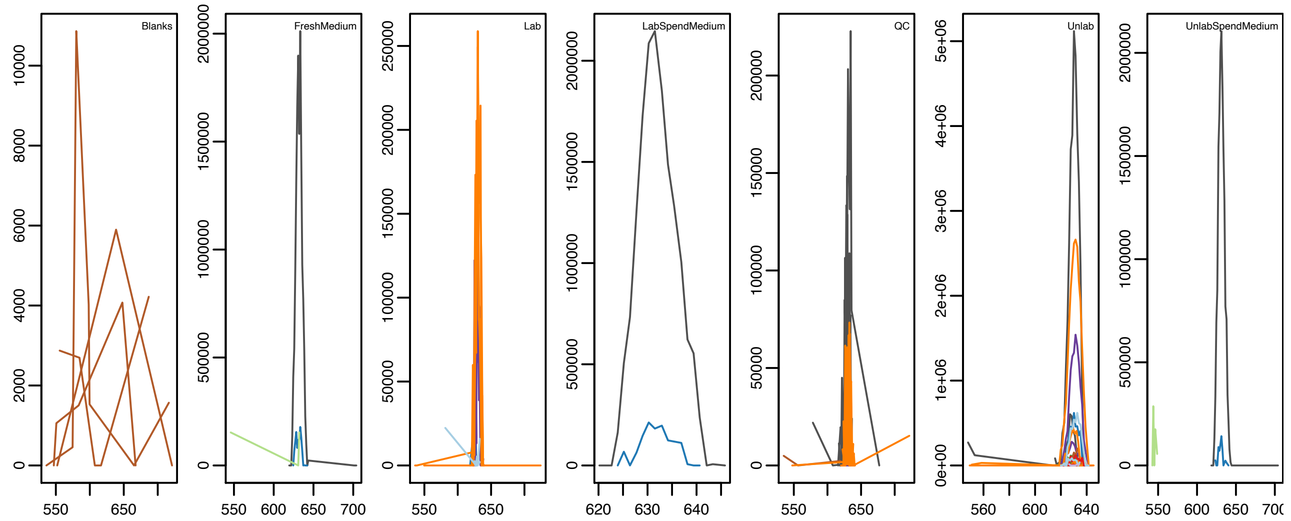

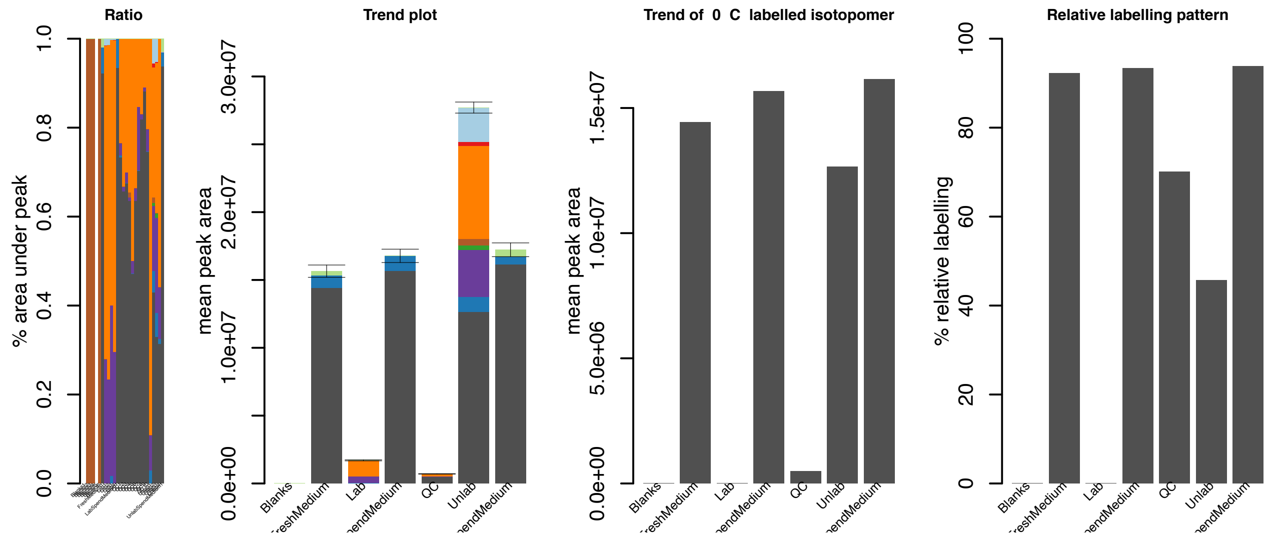

D−Glucosamine−6−phosphate

Formula: C6H14NO8P Mass: 259.046 Std.RT: 759 Ion: POS

G1

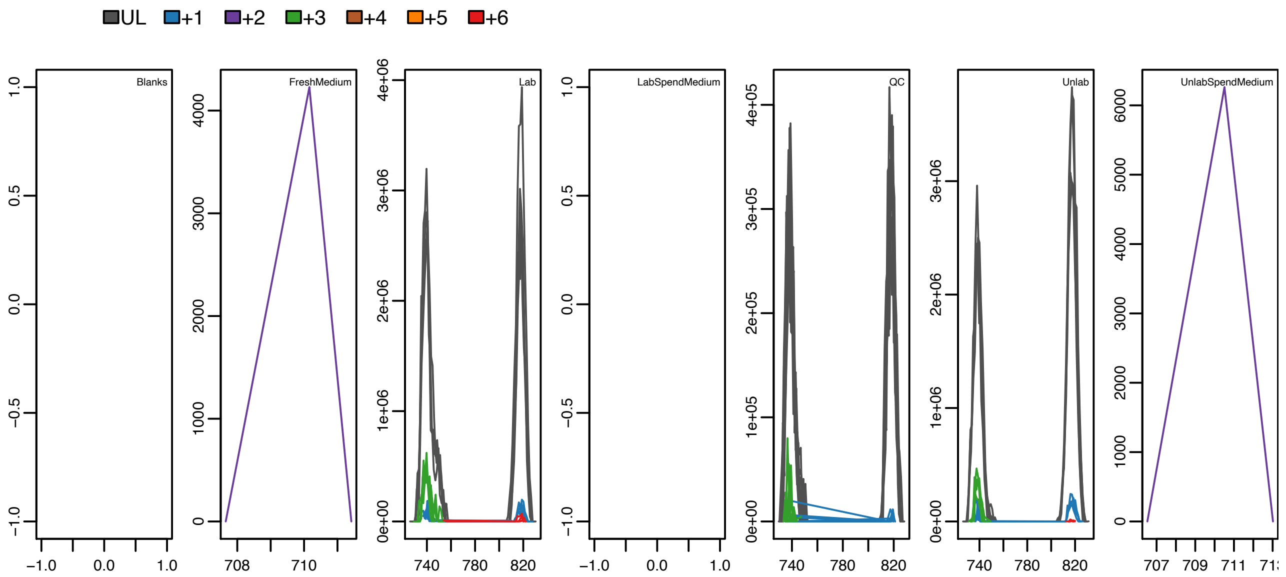

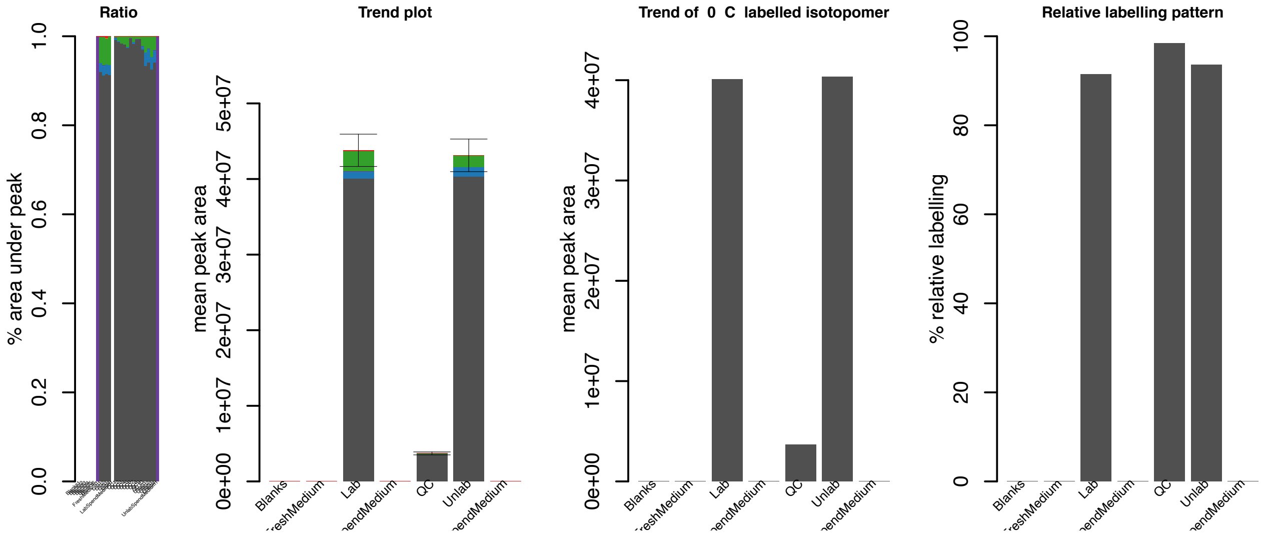

GDP

Formula: C10H15N5O11P2 Mass: 443.024 Std.RT: 816 Ion: POS

G1

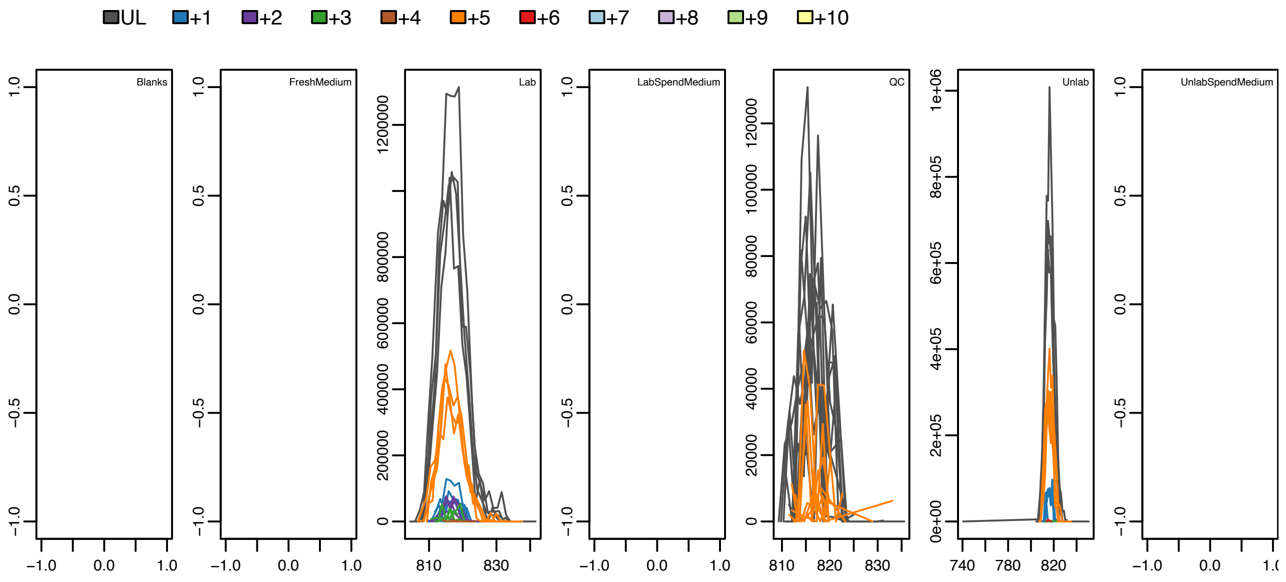

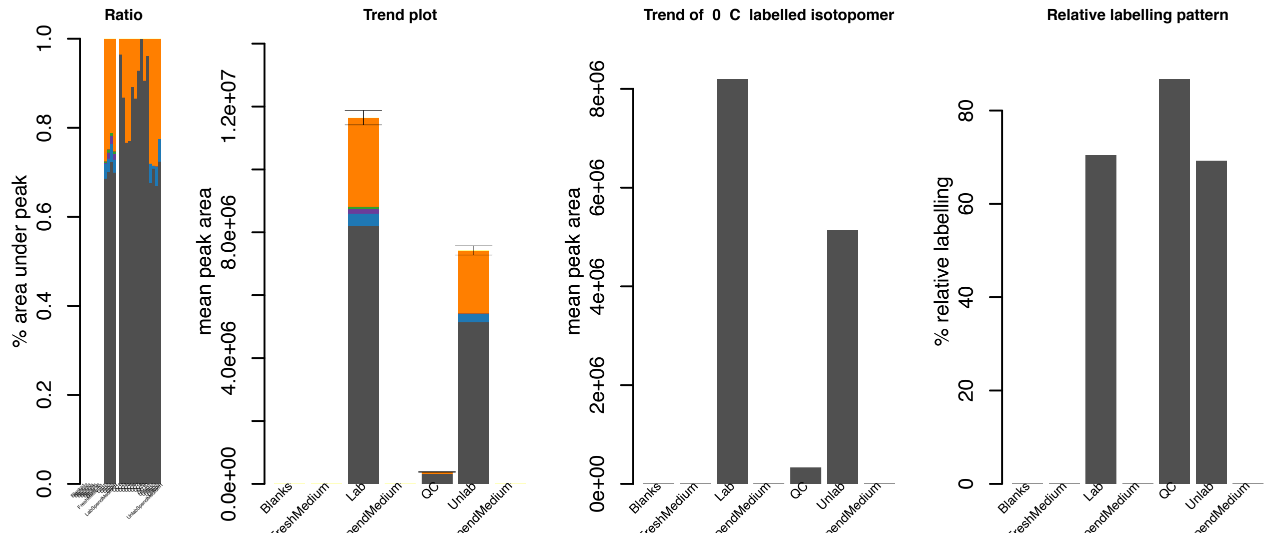

GMP

G1

Formula: C10H14N5O8P Mass: 363.058 Std.RT: 747 Ion: POS

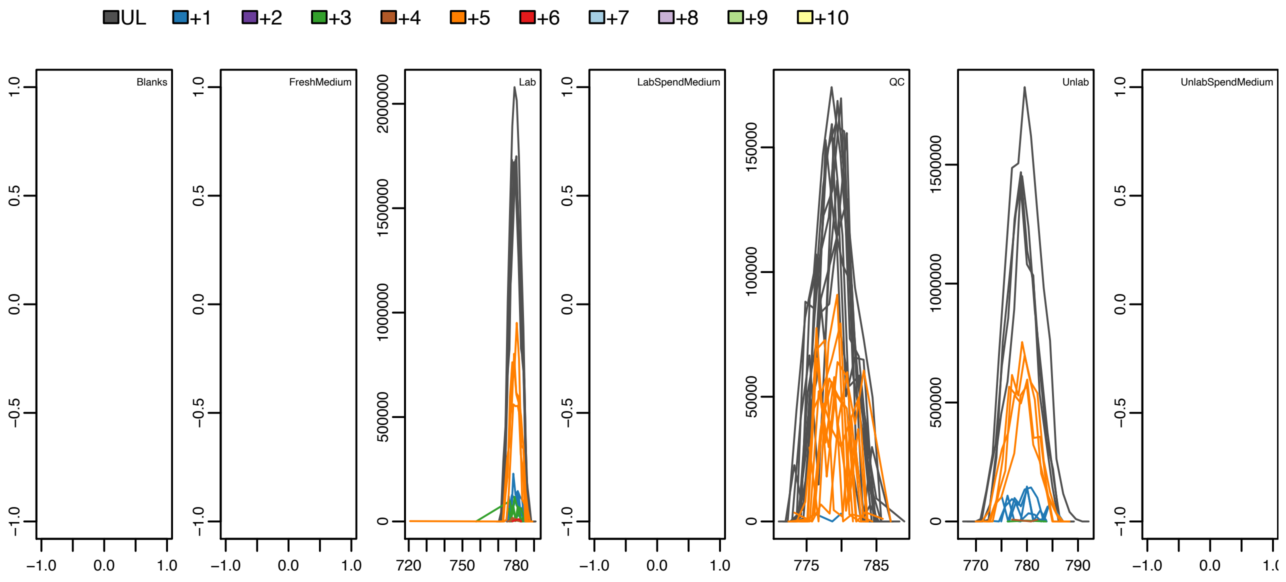

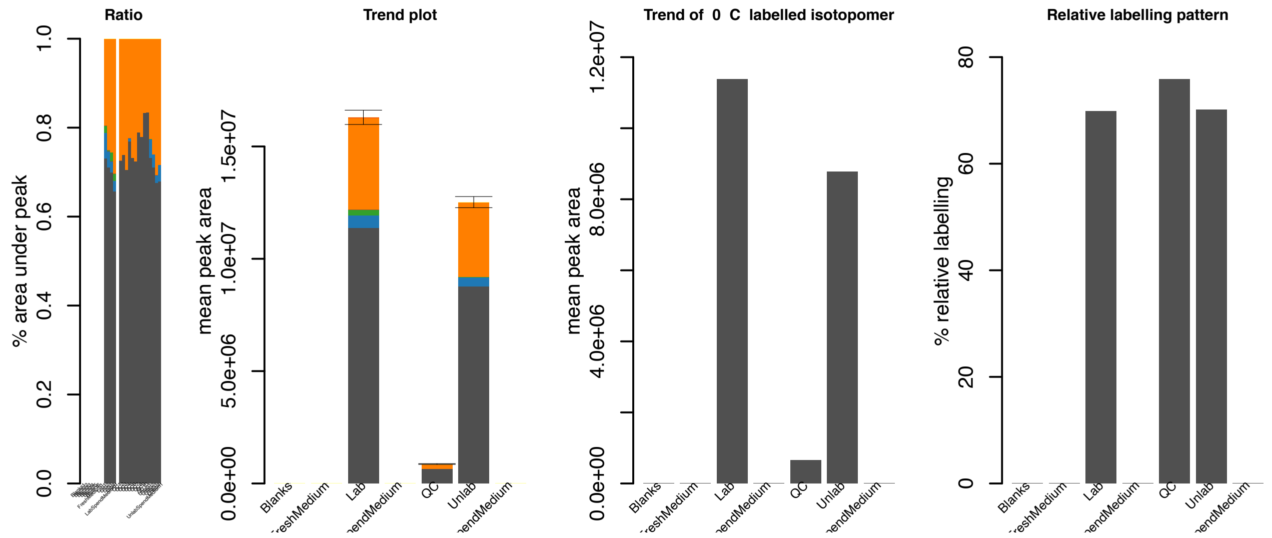

Glycine

Formula: $C_2H_5NO_2$ Mass: 75.032 Std.RT: 772.8 Ion: POS

G1

UL +1 +2

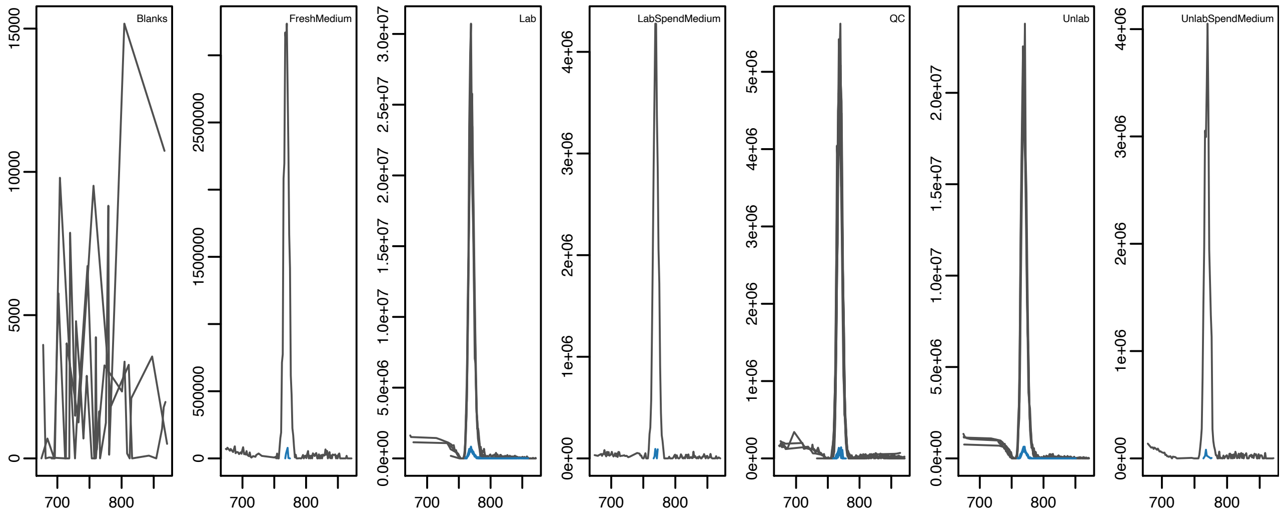

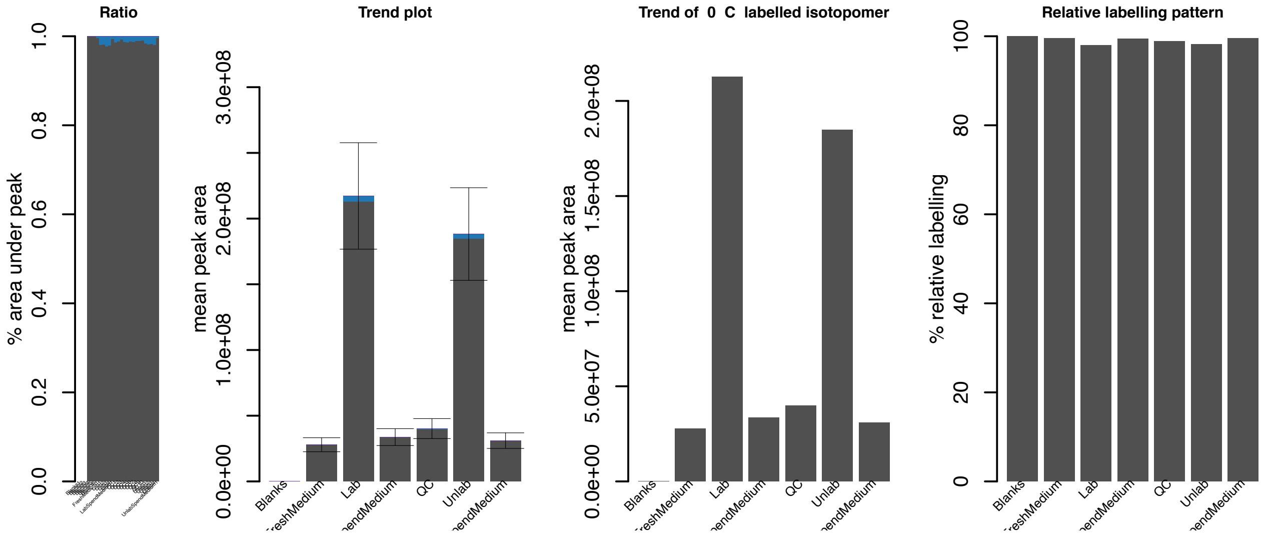

Guanine

Formula: C5H5N5O Mass: 151.049 Std.RT: 664.2 Ion: POS

G1

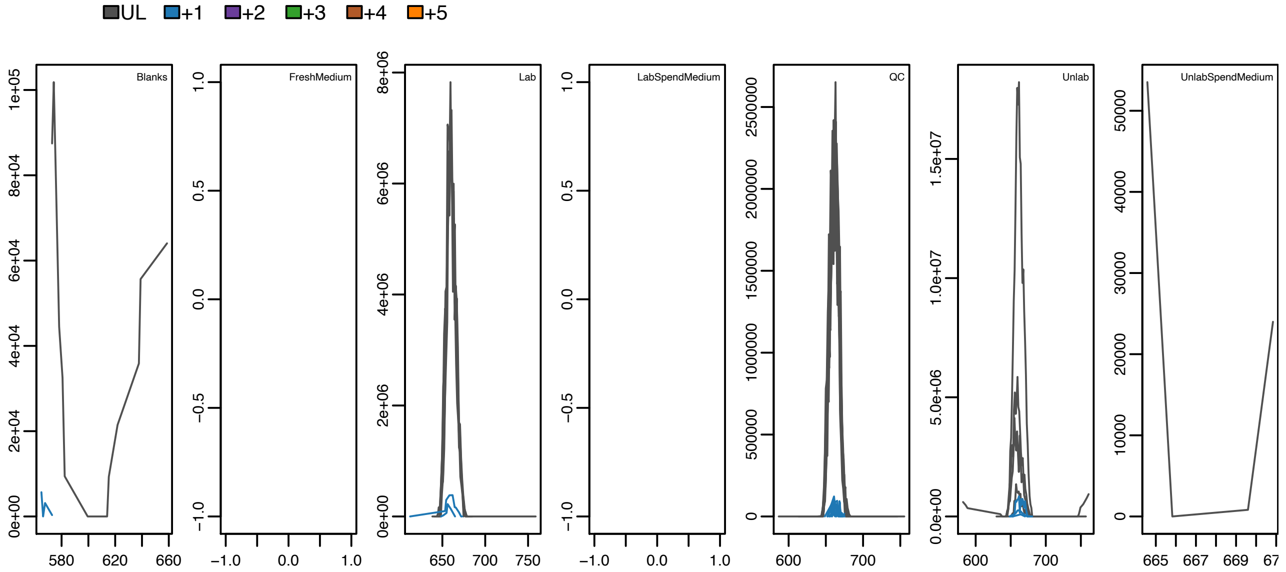

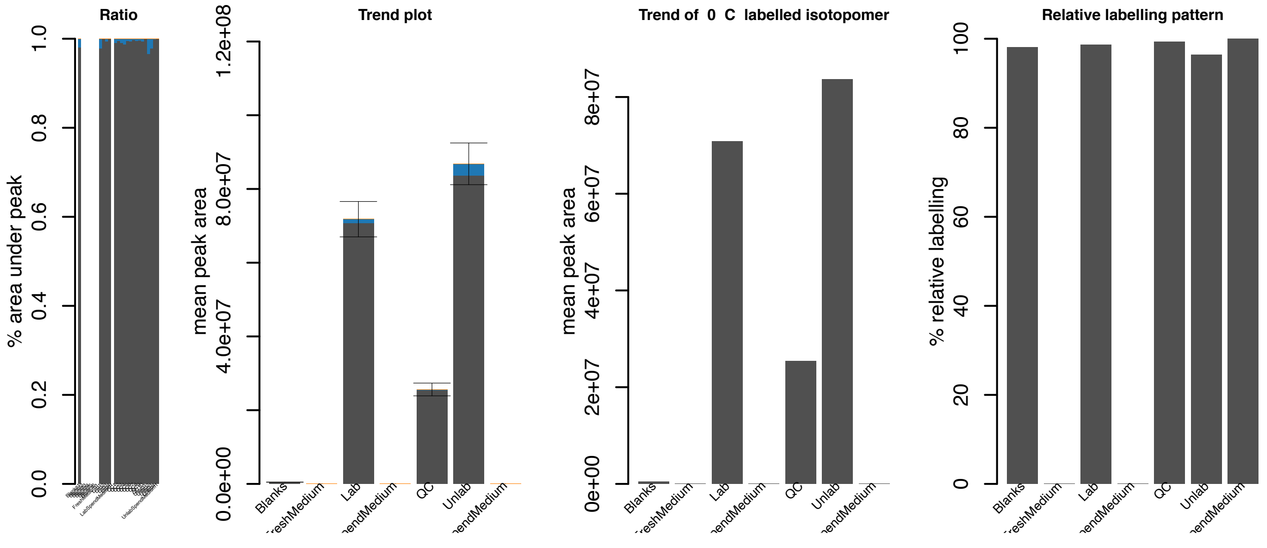

Hypoxanthine

Formula: C5H4N4O Mass: 136.039 Std.RT: 562.2 Ion: POS

G1

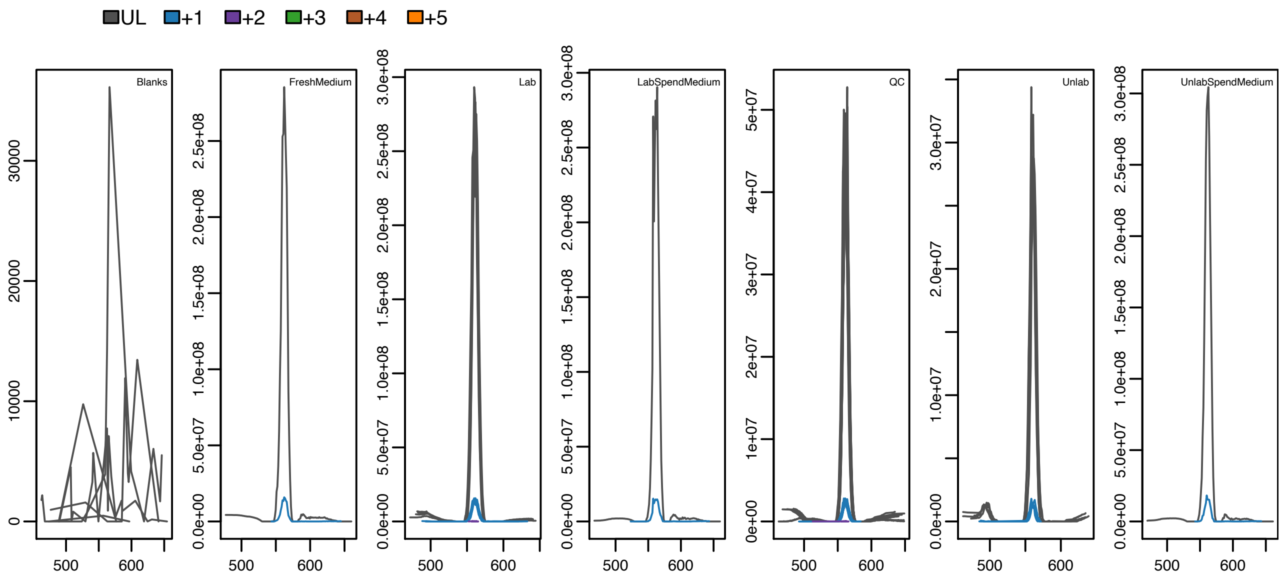

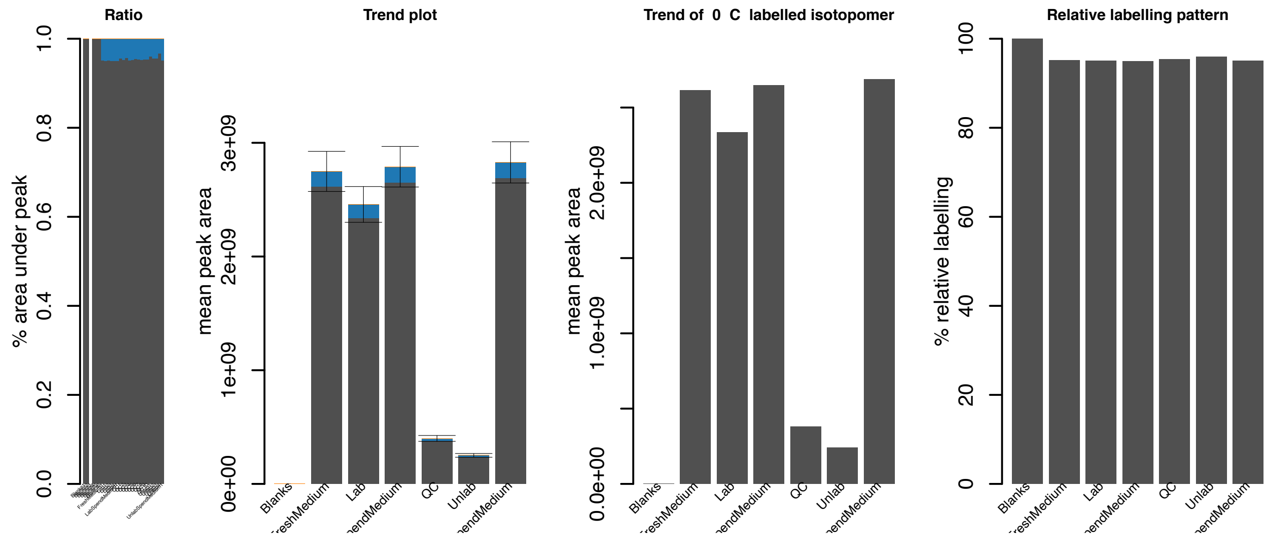

IMP

Formula: C10H13N4O8P Mass: 348.047 Std.RT: 732.6 Ion: POS

G1

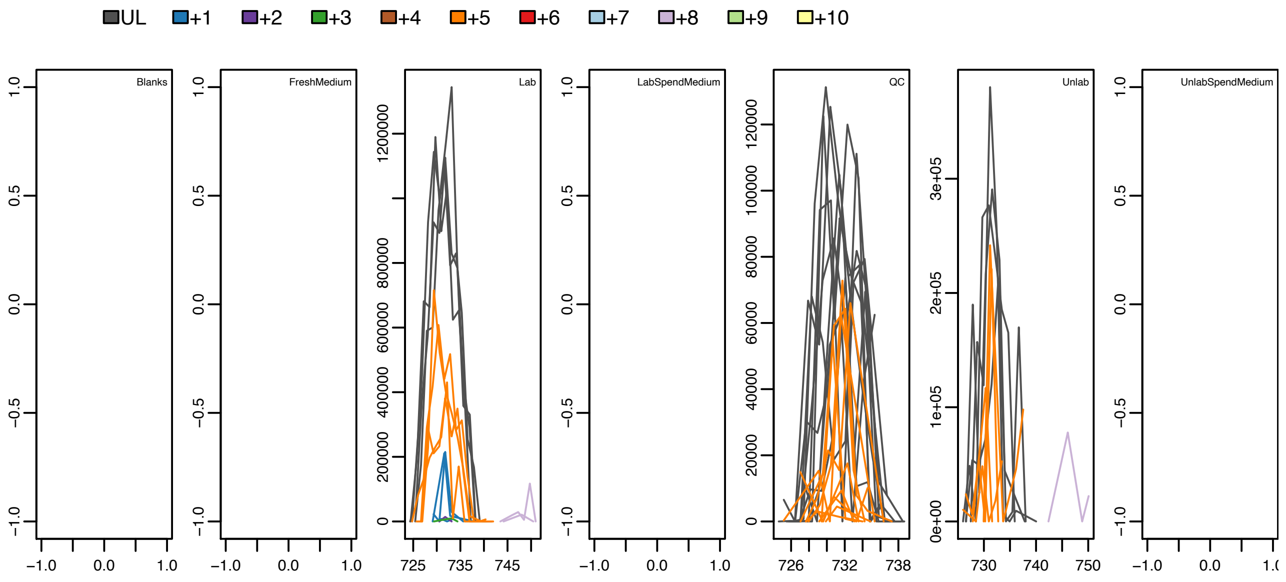

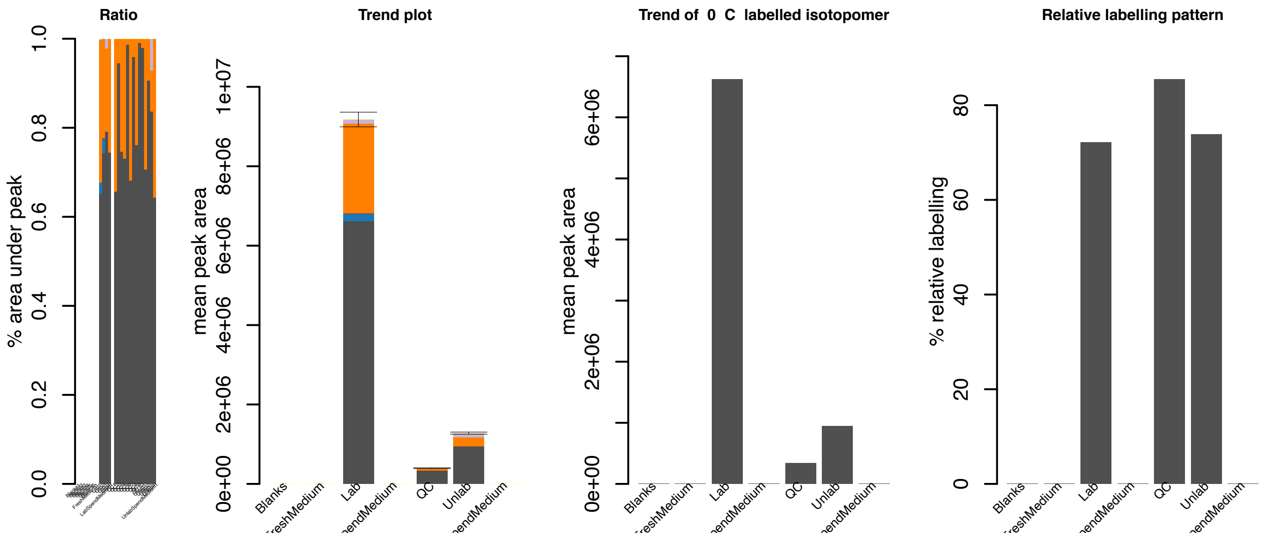

Imidazole−4−acetate

Formula: C5H6N2O2 Mass: 126.043 Std.RT: 625.2 Ion: POS

G1

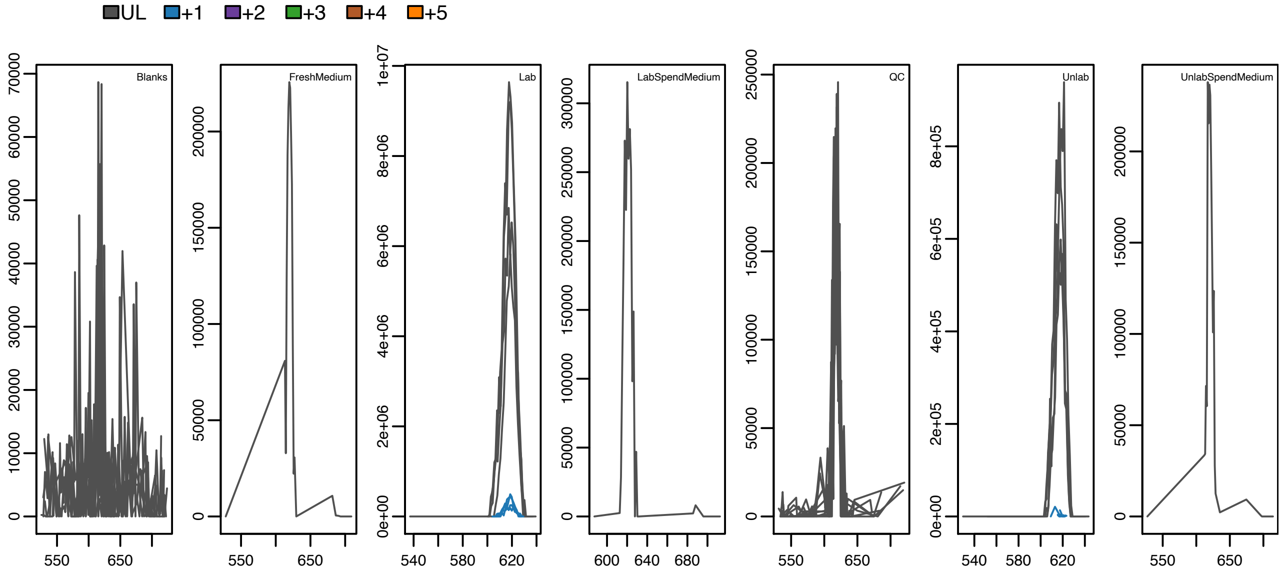

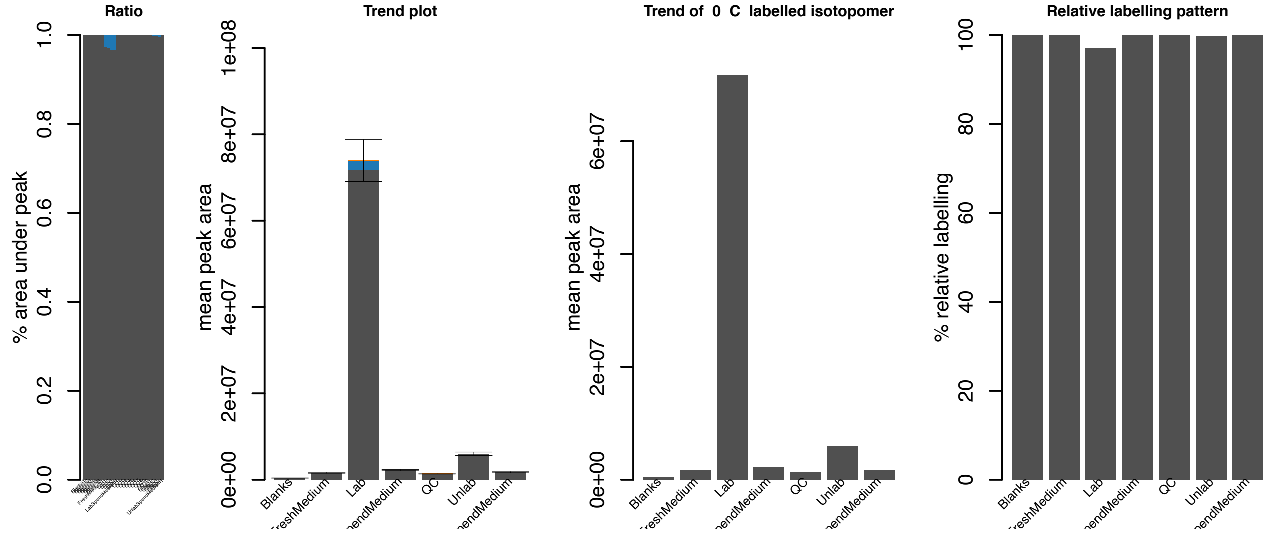

Isonicotinic acid

Formula: C6H5NO2 Mass: 123.032 Std.RT: 447.6 Ion: POS

G1

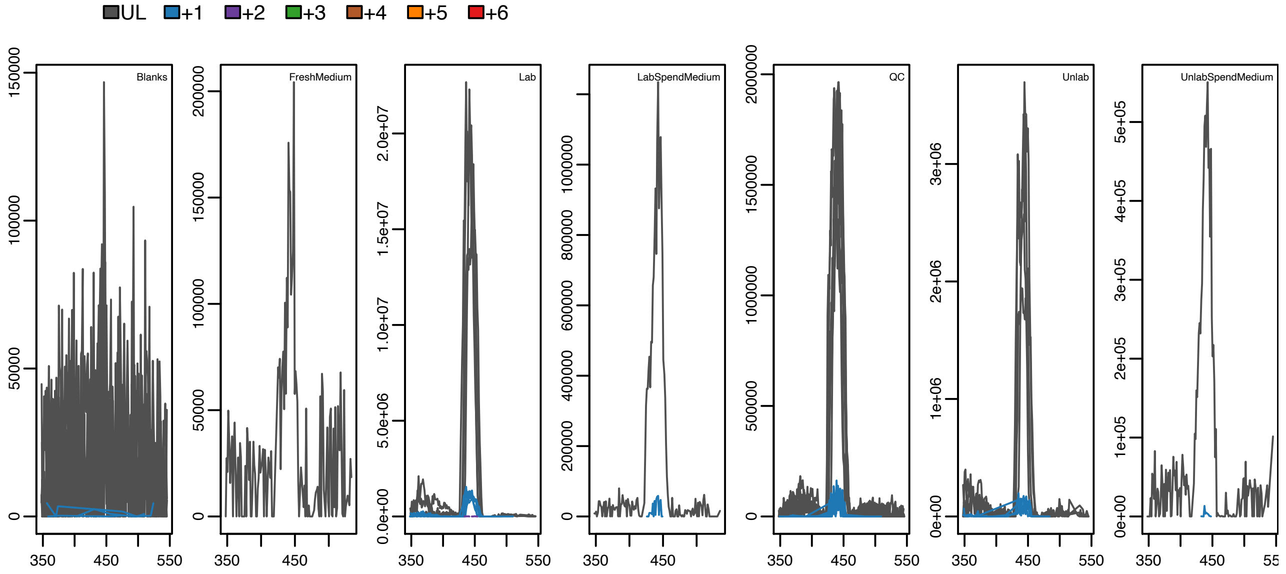

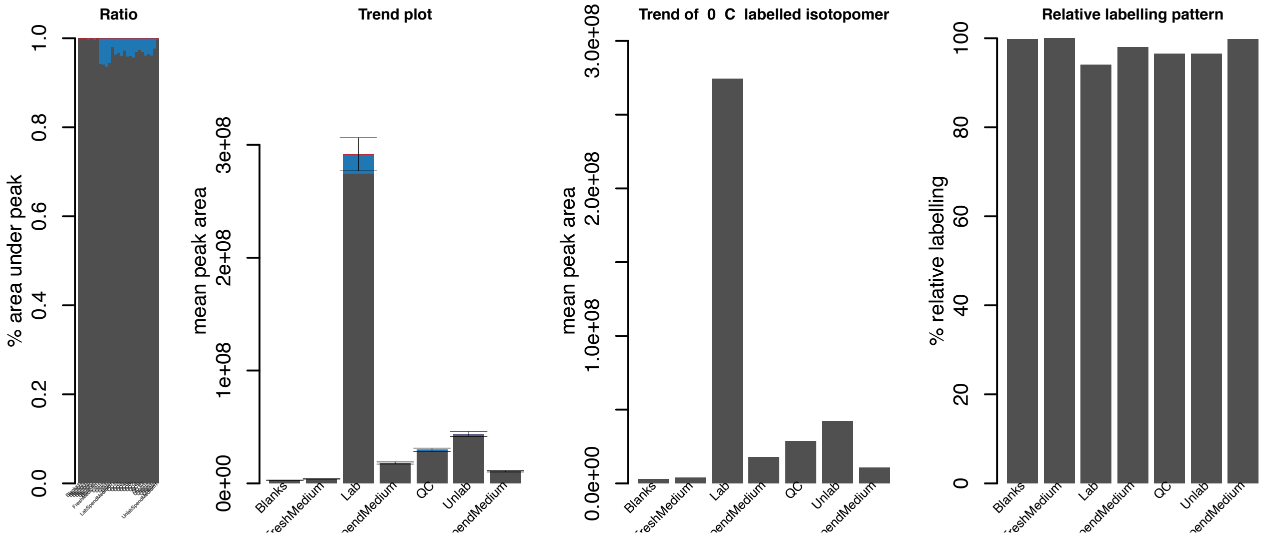

L−Alanine

Formula: C3H7NO2 Mass: 89.048 Std.RT: 737.4 Ion: POS

G1

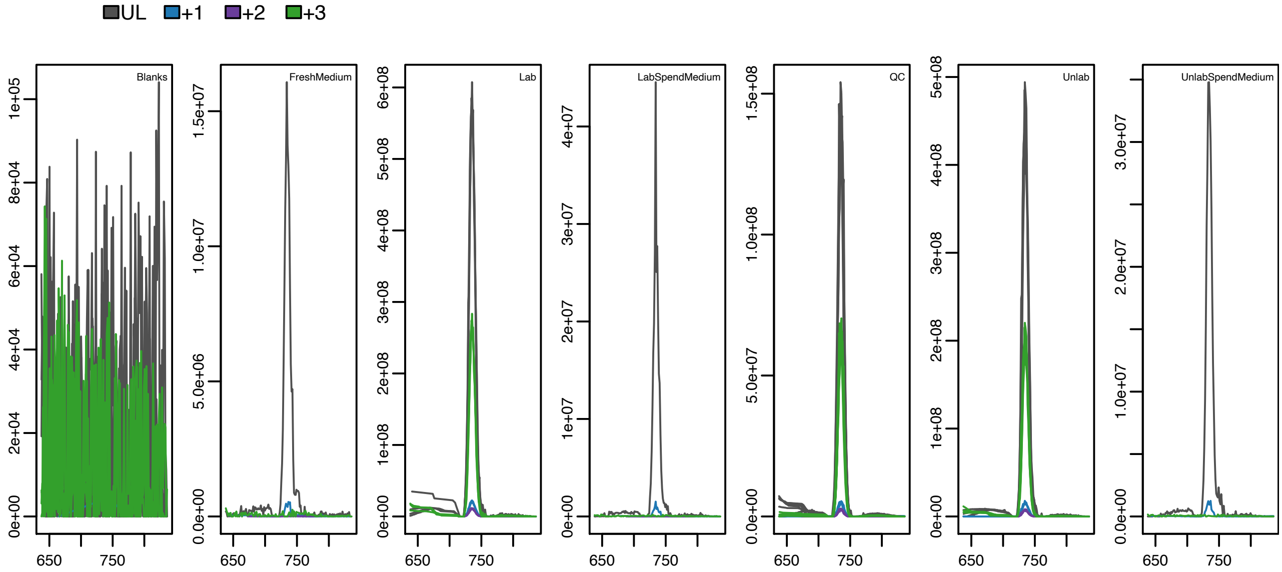

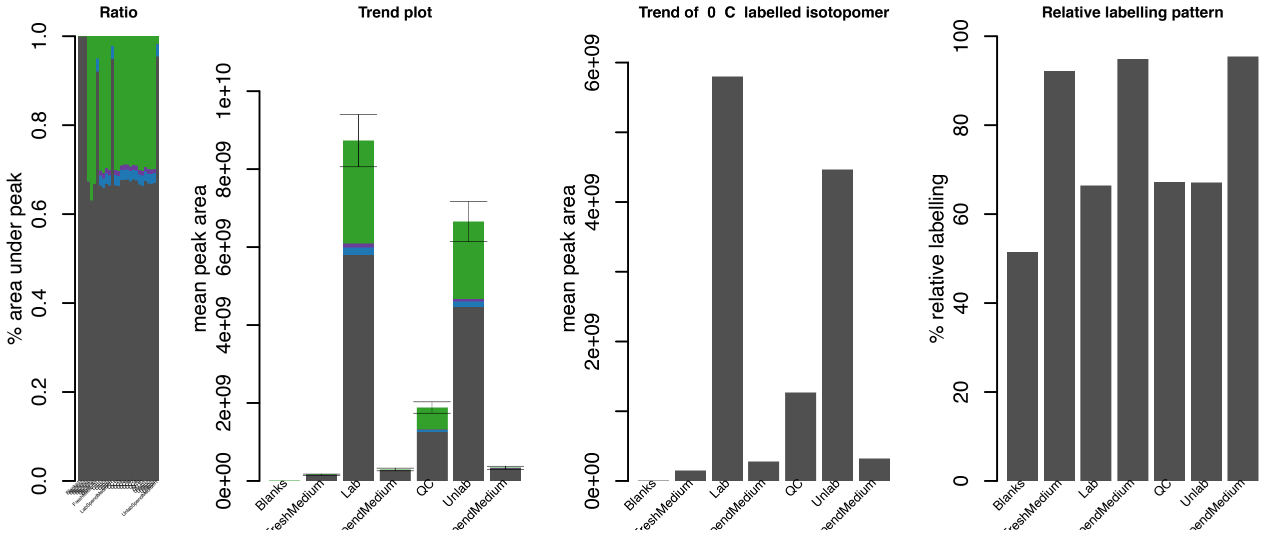

L−Arginine

Formula: $C6H14N4O2$ Mass: 174.112 Std.RT: 1209.6 Ion: POS

G1

UL +1 +2 +3 +4 +5 +6

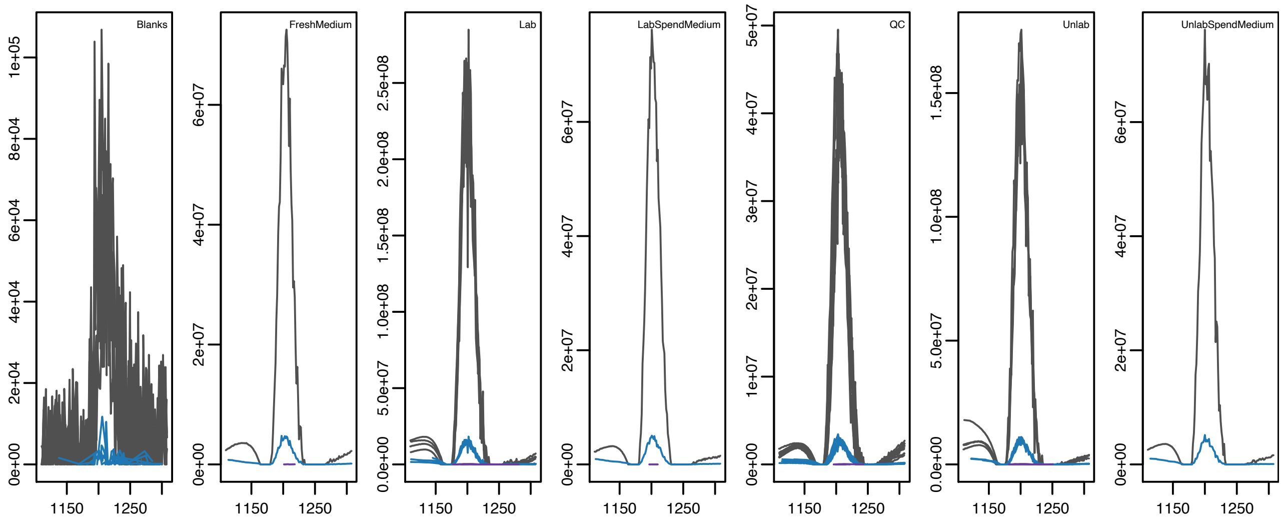

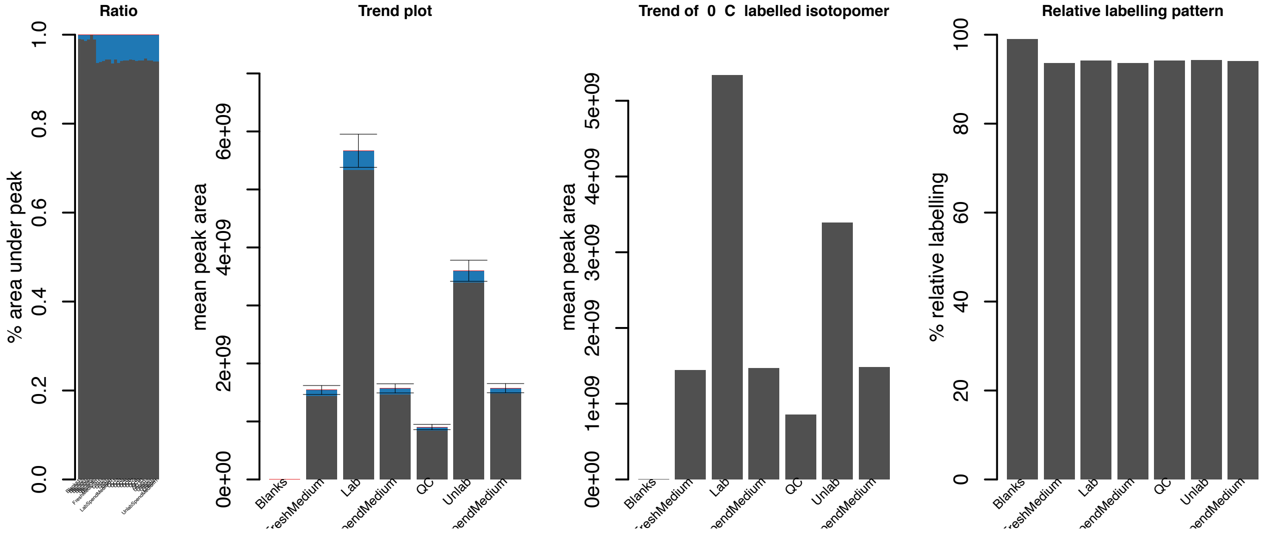

L−Asparagine

Formula: C4H8N2O3 Mass: 132.053 Std.RT: 754.8 Ion: POS

G1

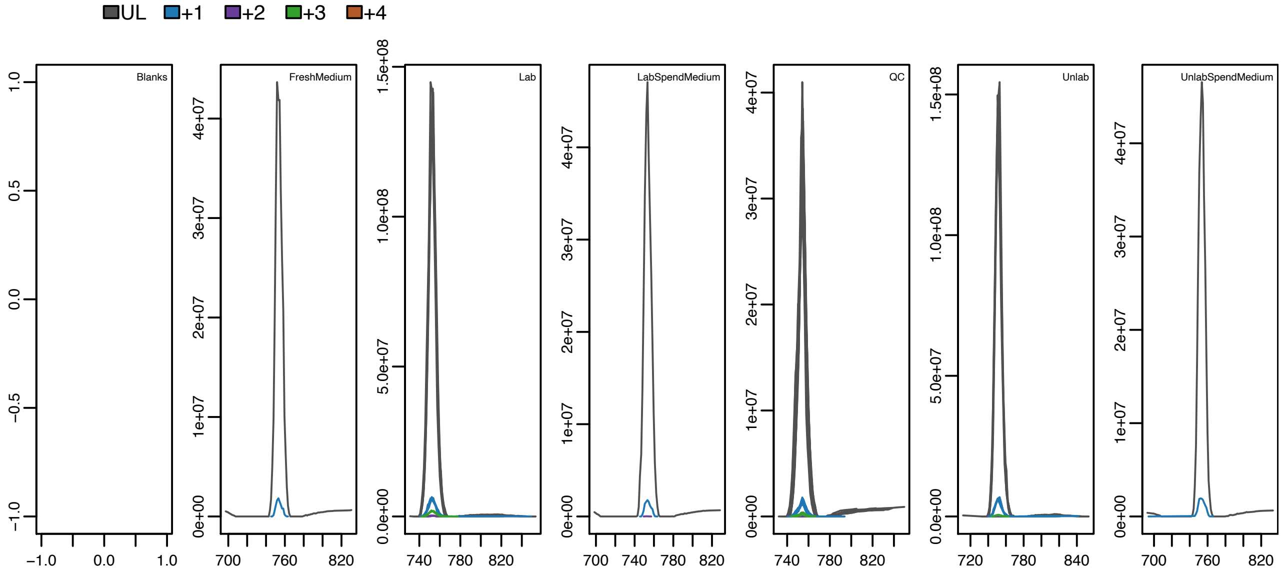

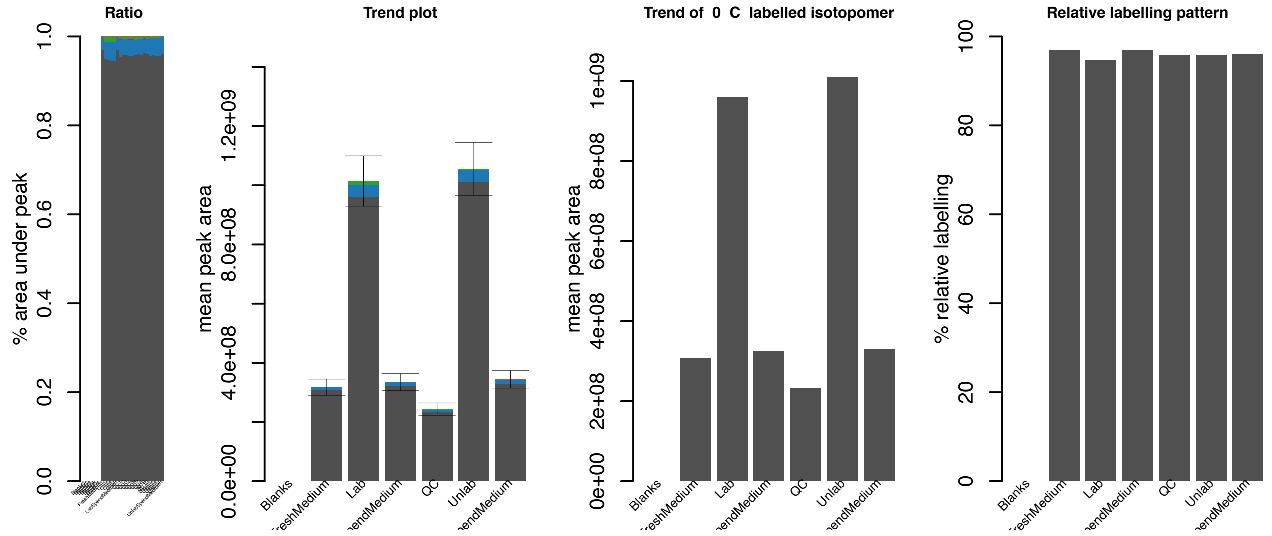

L−Carnitine

Formula: $C7H15NO3$ Mass: 161.105 Std.RT: 670.8 Ion: POS

G1

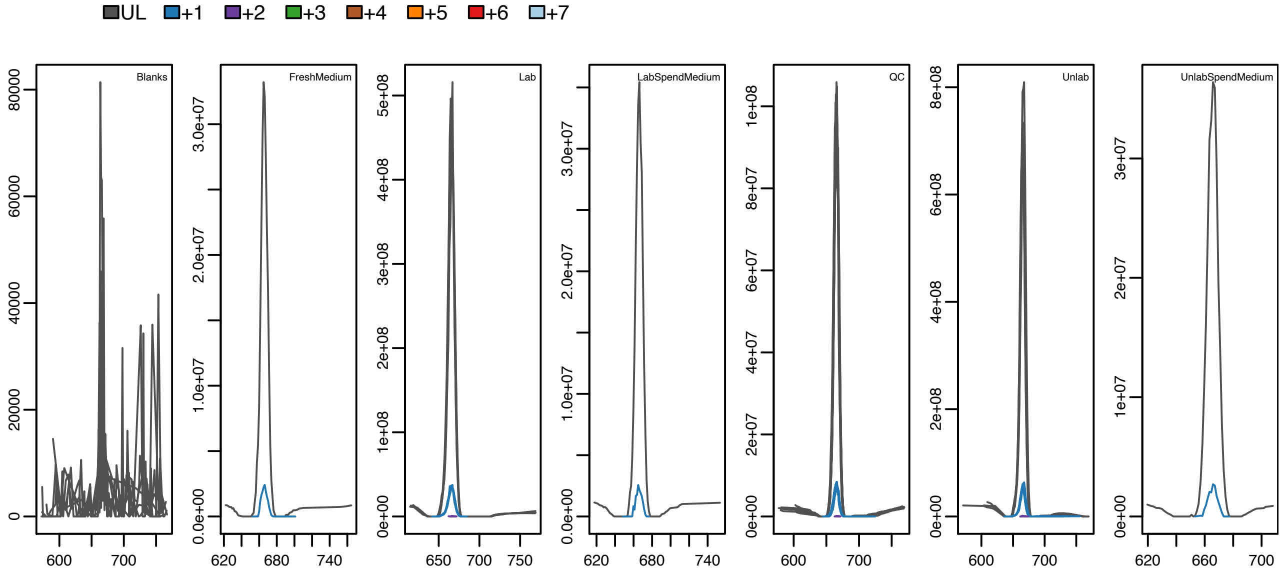

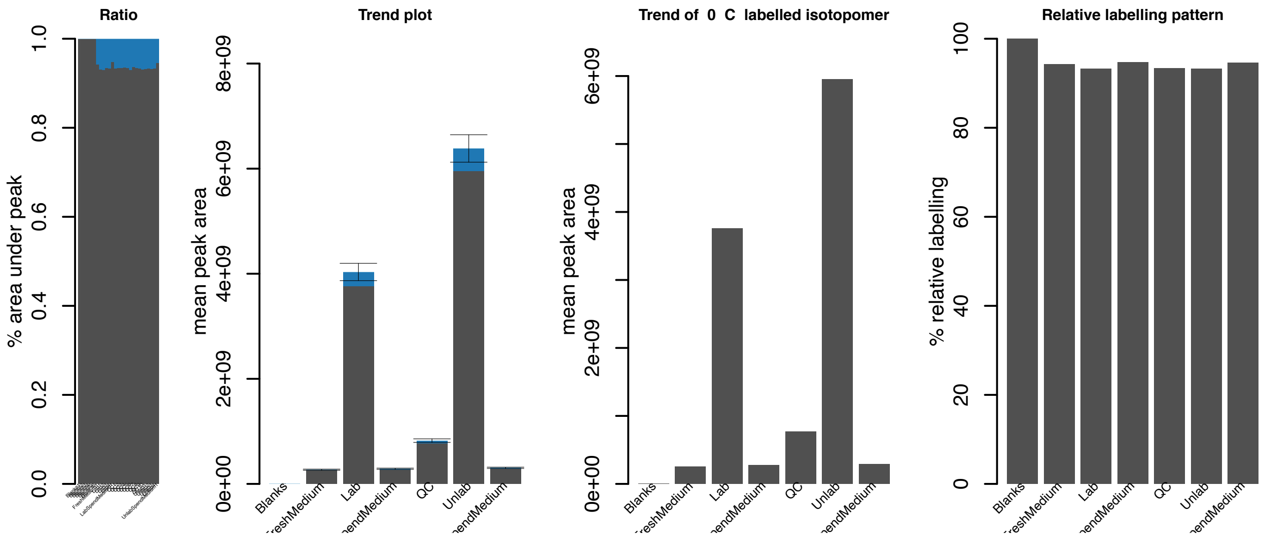

L−Citrulline

Formula: C6H13N3O3 Mass: 175.096 Std.RT: 767.4 Ion: POS

G1

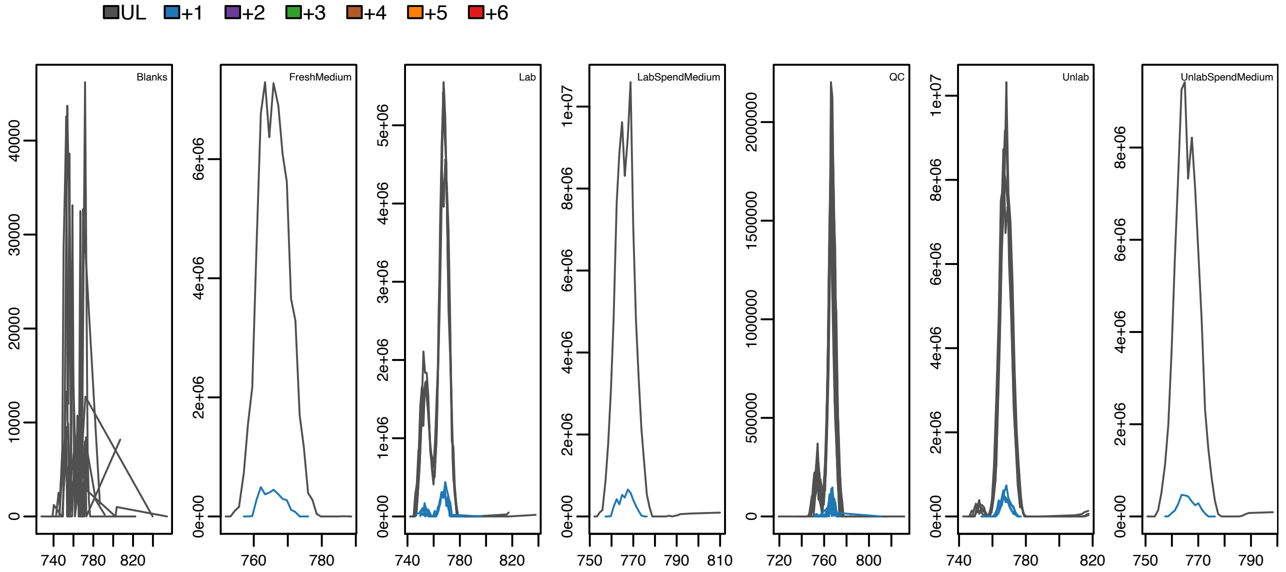

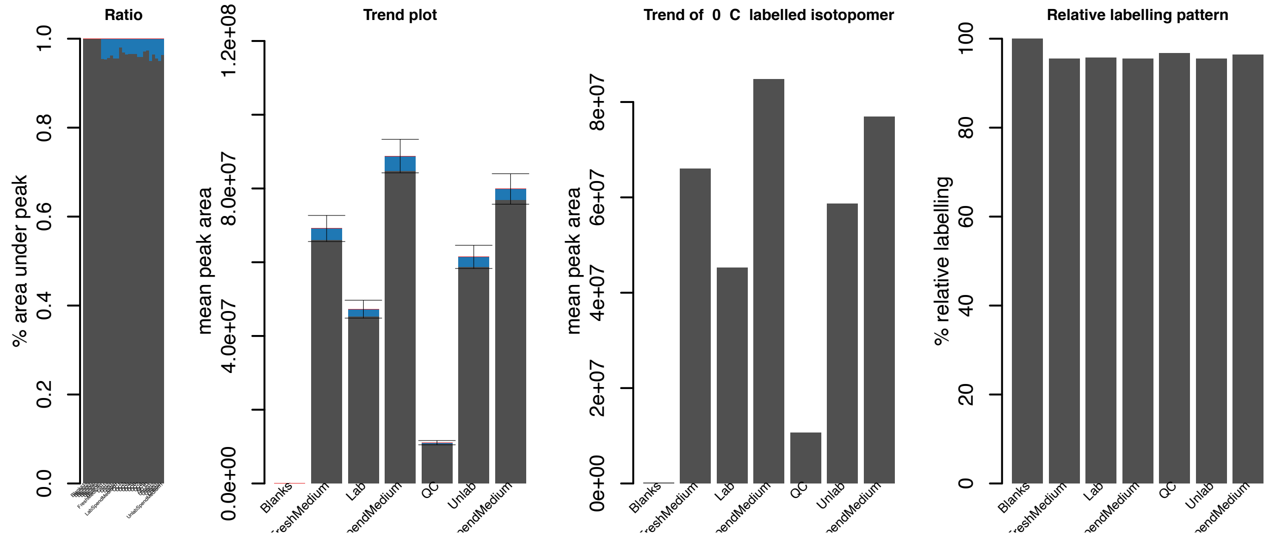

L−Cystathionine

Formula: C7H14N2O4S Mass: 222.067 Std.RT: 798.6 Ion: POS

G1

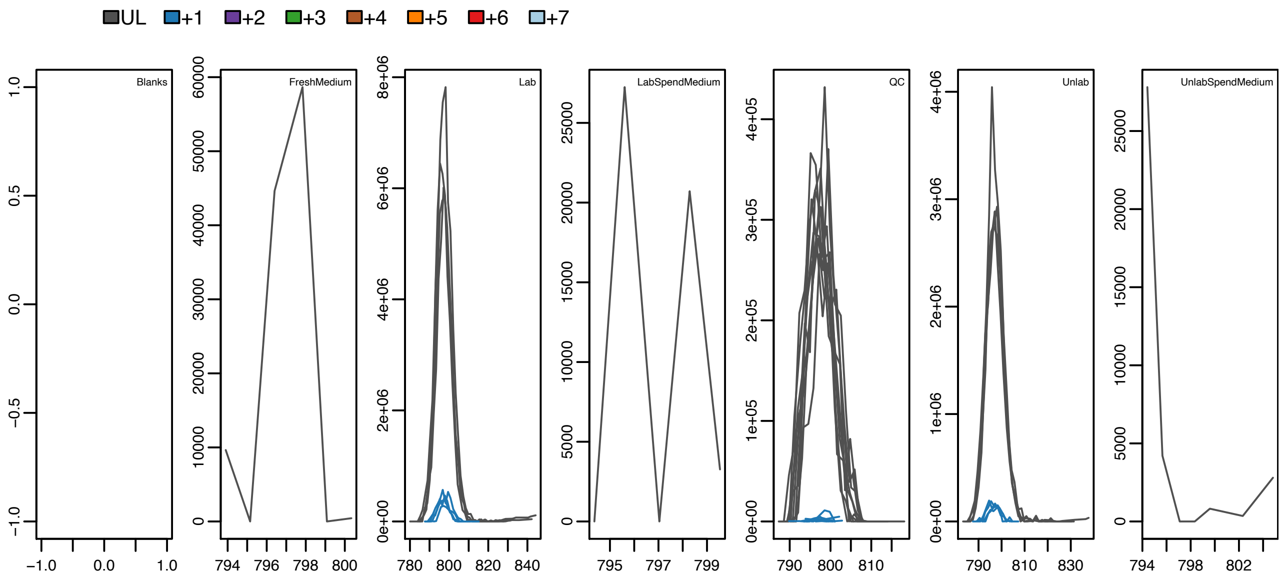

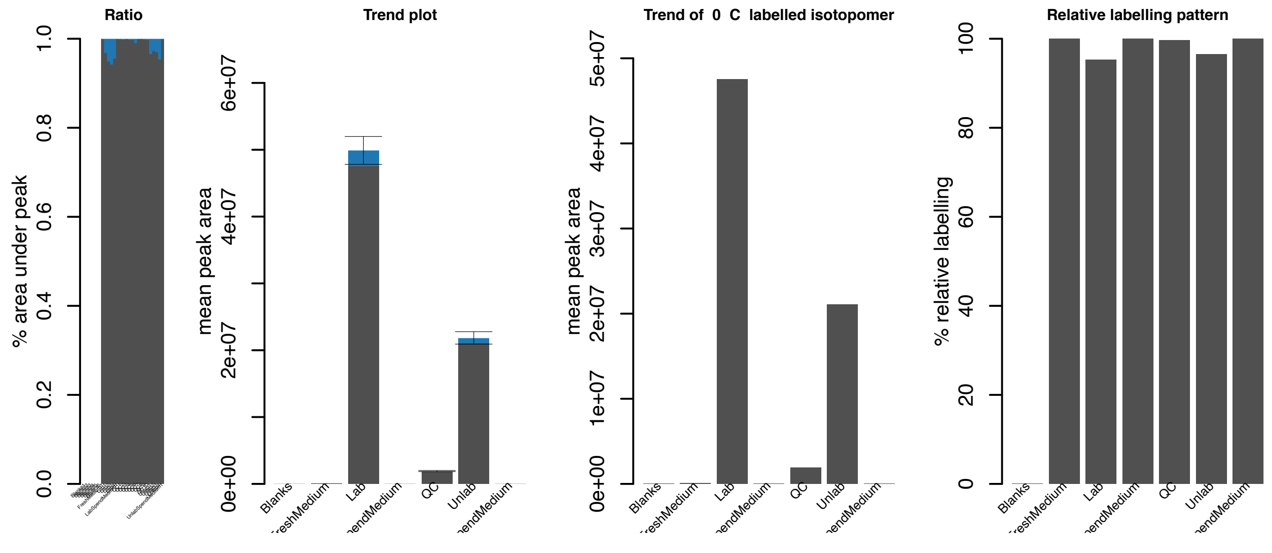

L−Glutamate

Formula: C5H9NO4 Mass: 147.053 Std.RT: 723.6 Ion: POS

G1

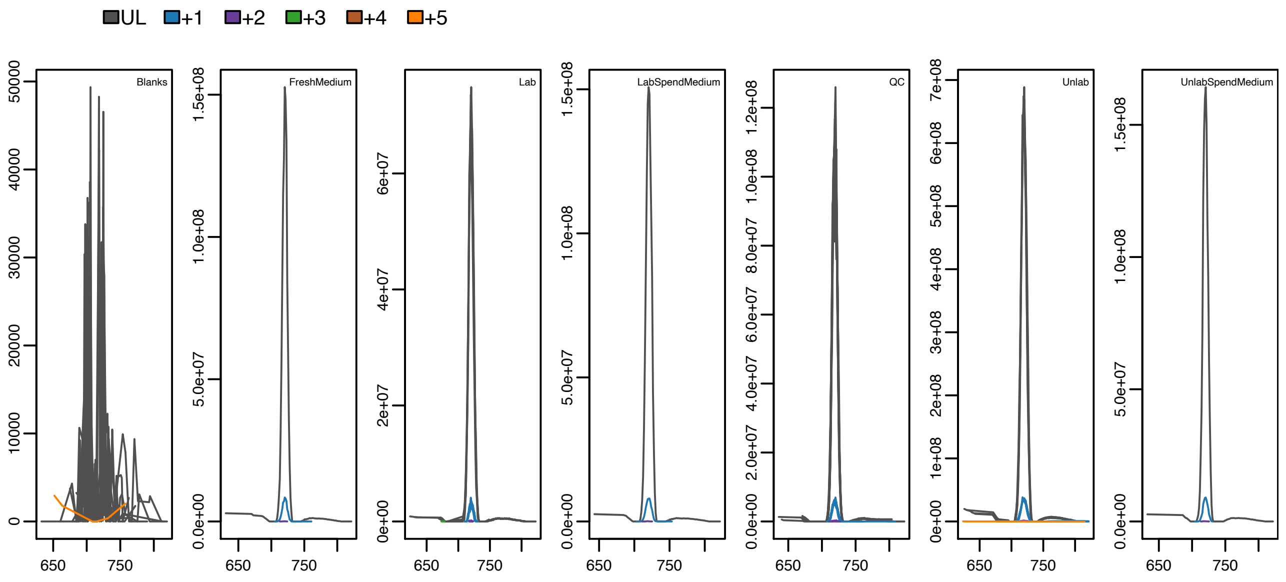

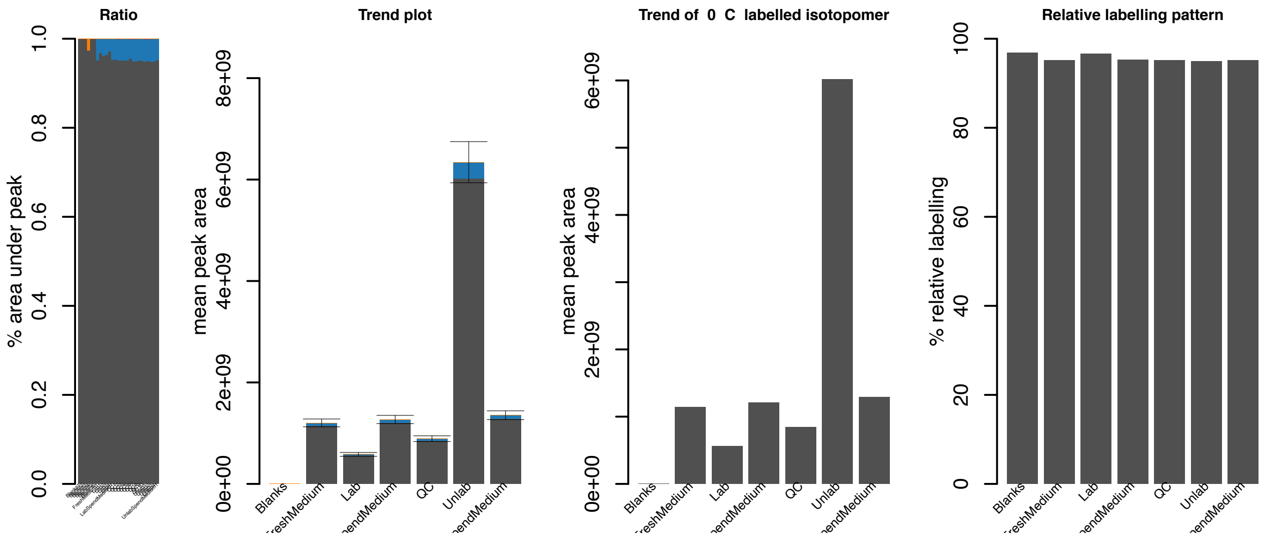

L−Glutamine

Formula: C5H10N2O3 Mass: 146.069 Std.RT: 739.2 Ion: POS

G1

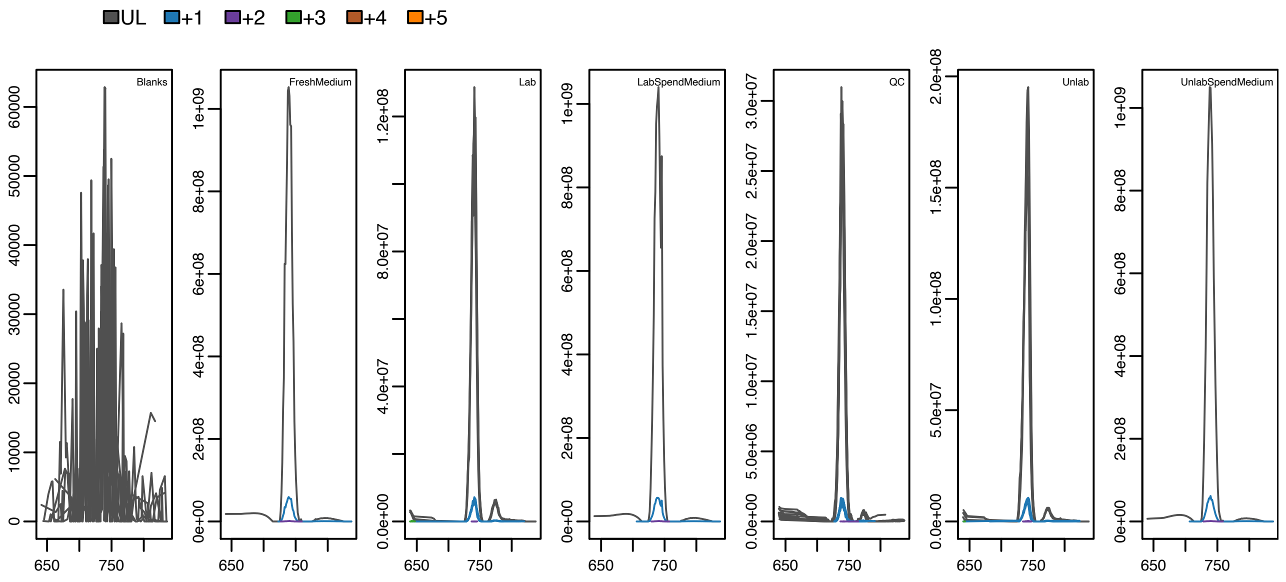

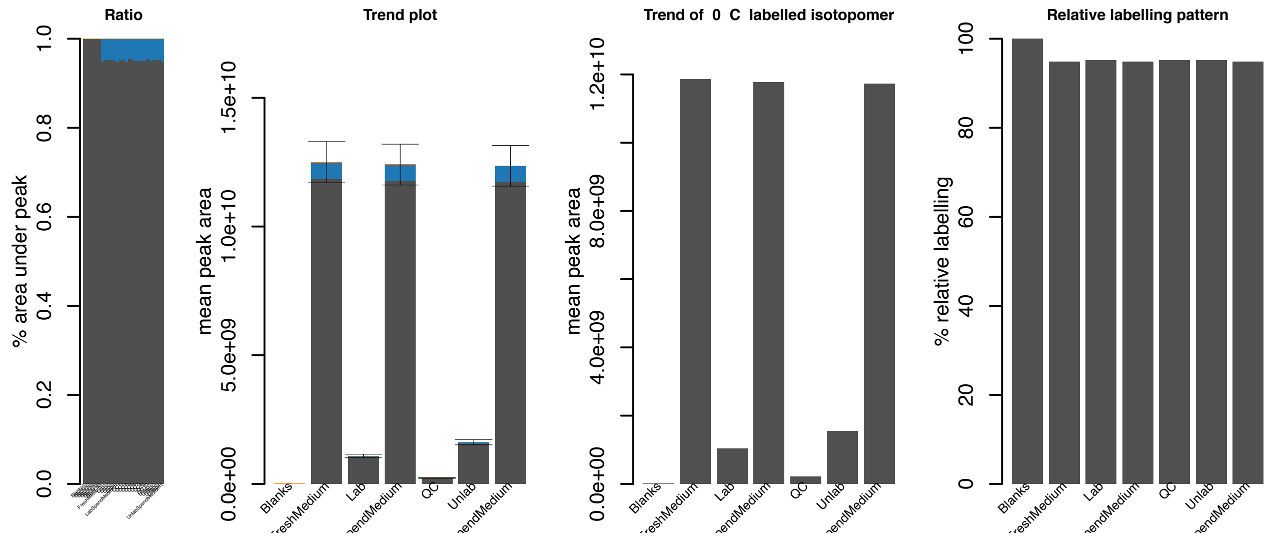

L−Leucine

Formula: C6H13NO2 Mass: 131.095 Std.RT: 578.4 Ion: POS

G1

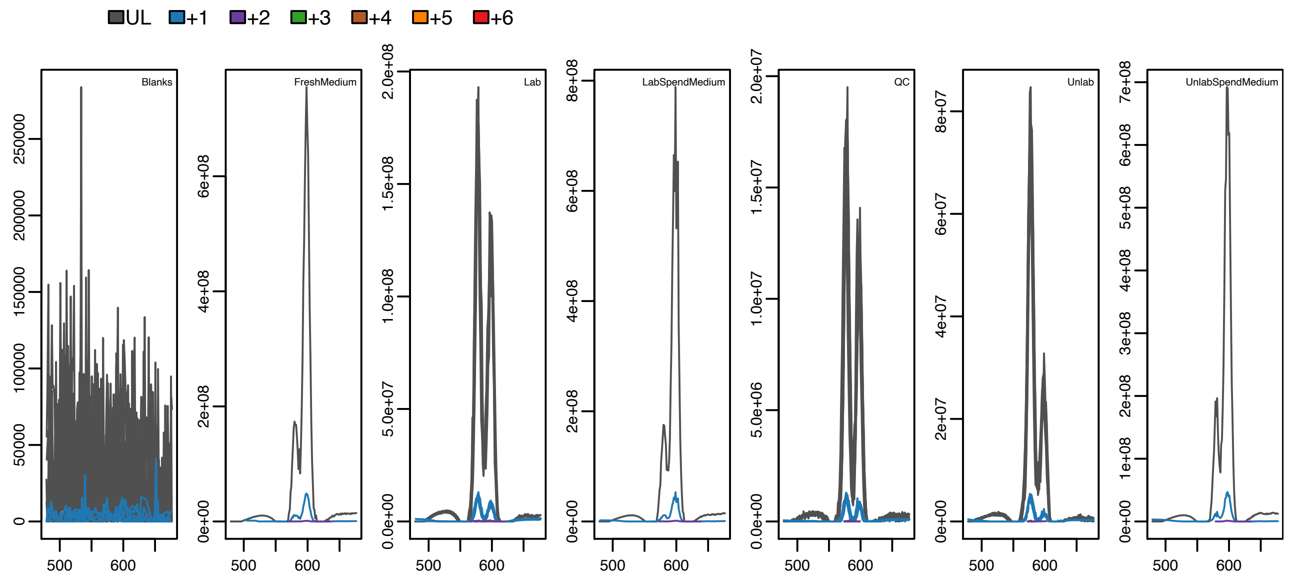

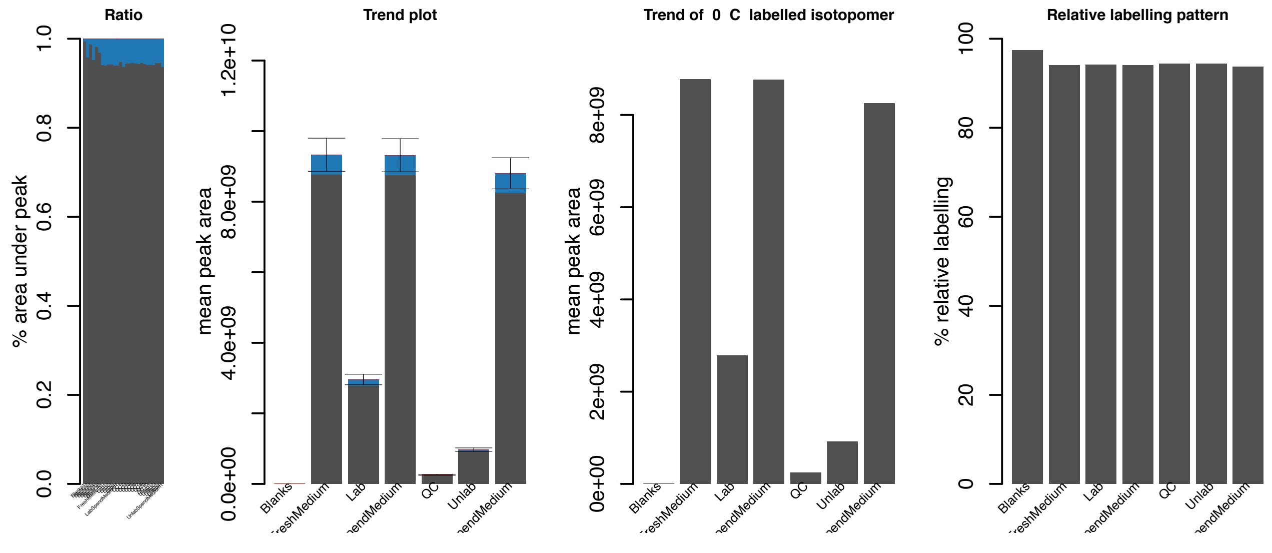

L−Serine

Formula: C3H7NO3 Mass: 105.043 Std.RT: 775.2 Ion: POS

G1

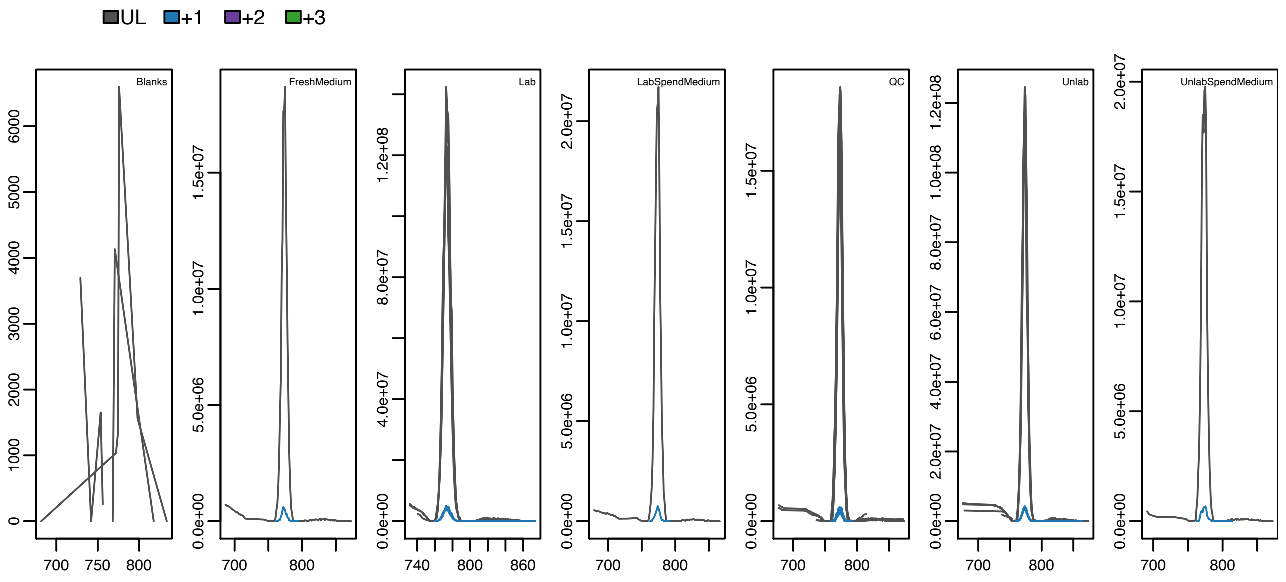

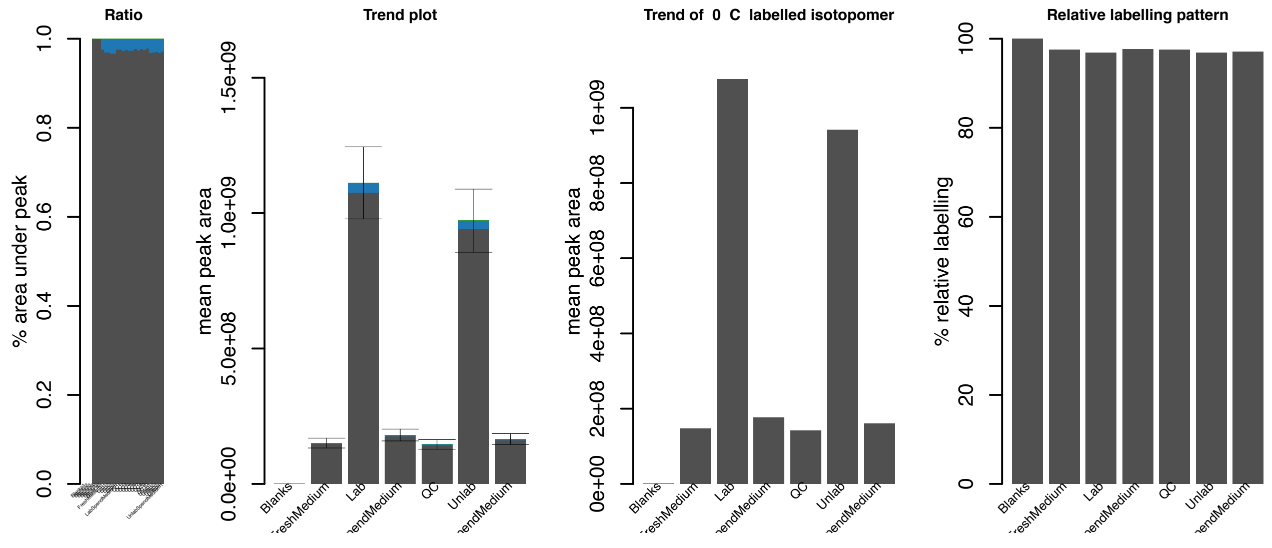

L−Threonine

Formula: C4H9NO3 Mass: 119.058 Std.RT: 728.4 Ion: POS

G1

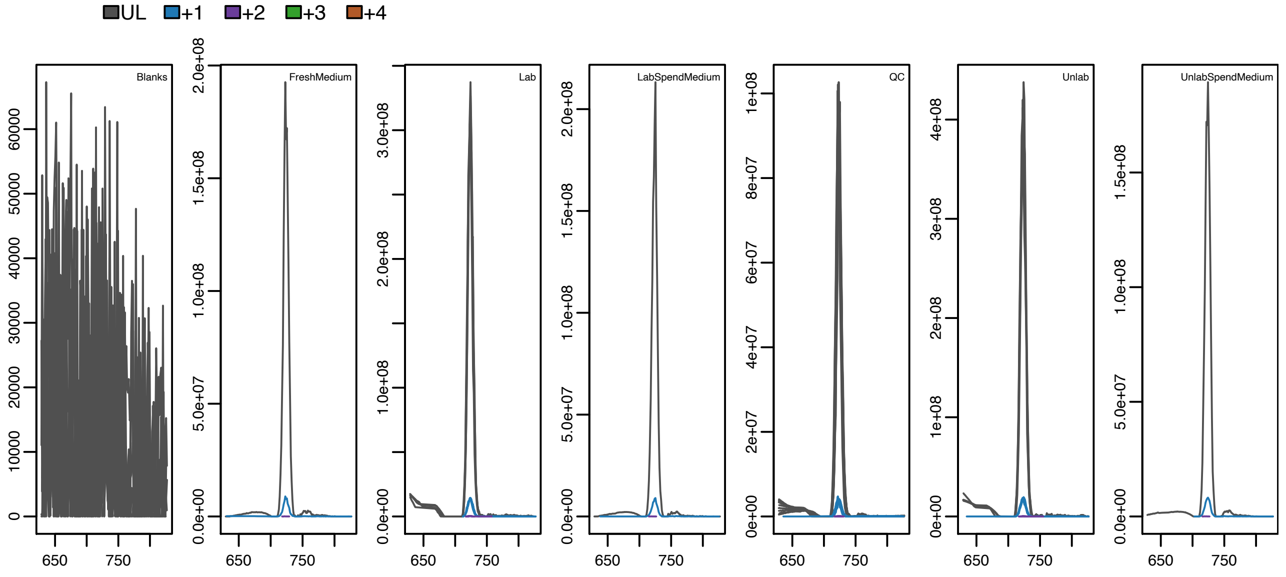

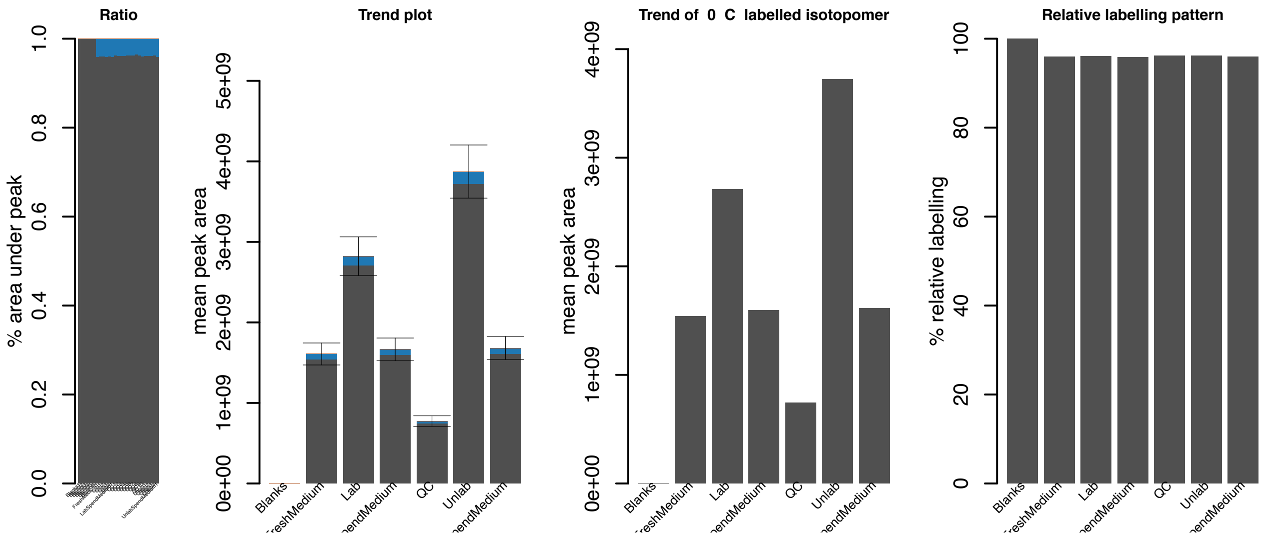

L−Tryptophan

Formula: C11H12N2O2   Mass: 204.09   Std.RT: 630   Ion: POS

G1

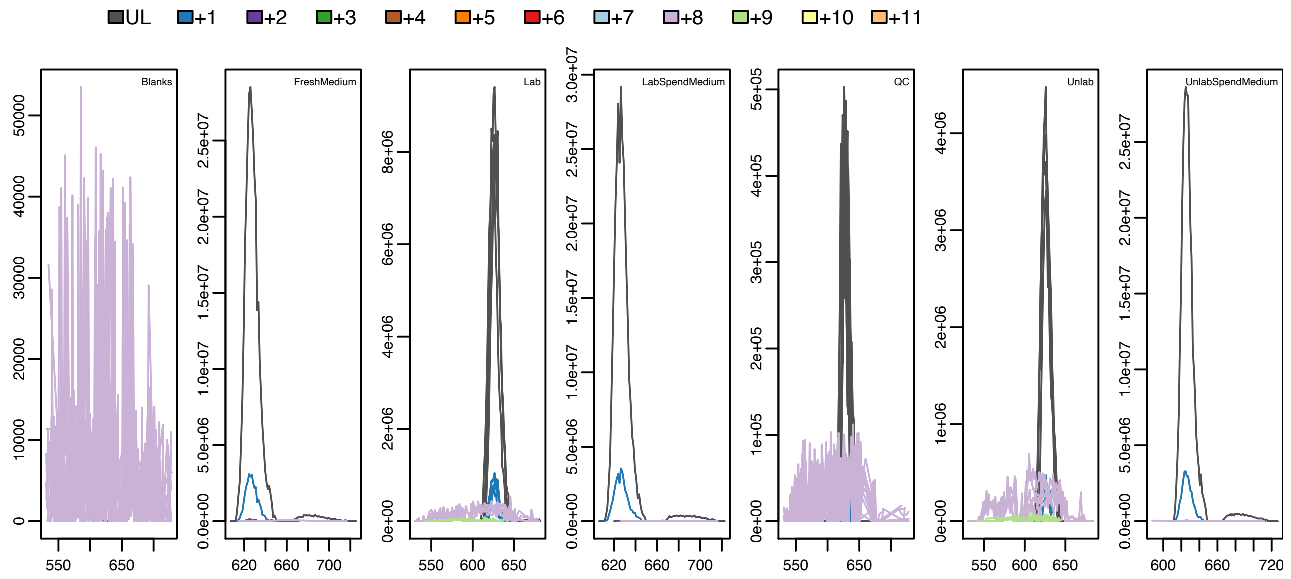

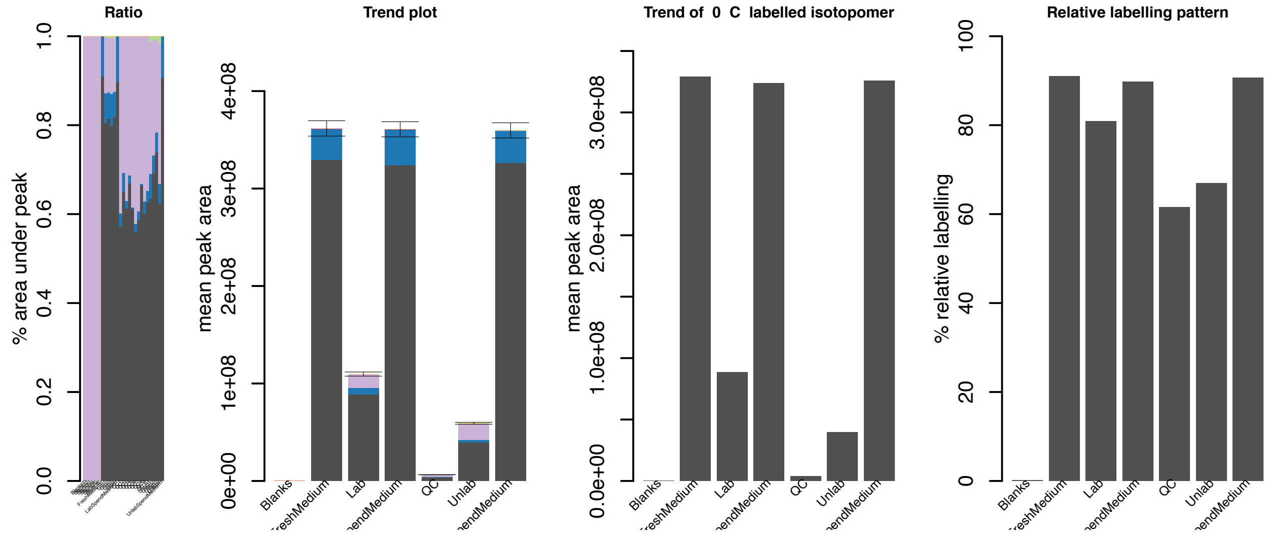

L−Tyrosine

Formula: C9H11NO3 Mass: 181.074 Std.RT: 680.4 Ion: POS

G1

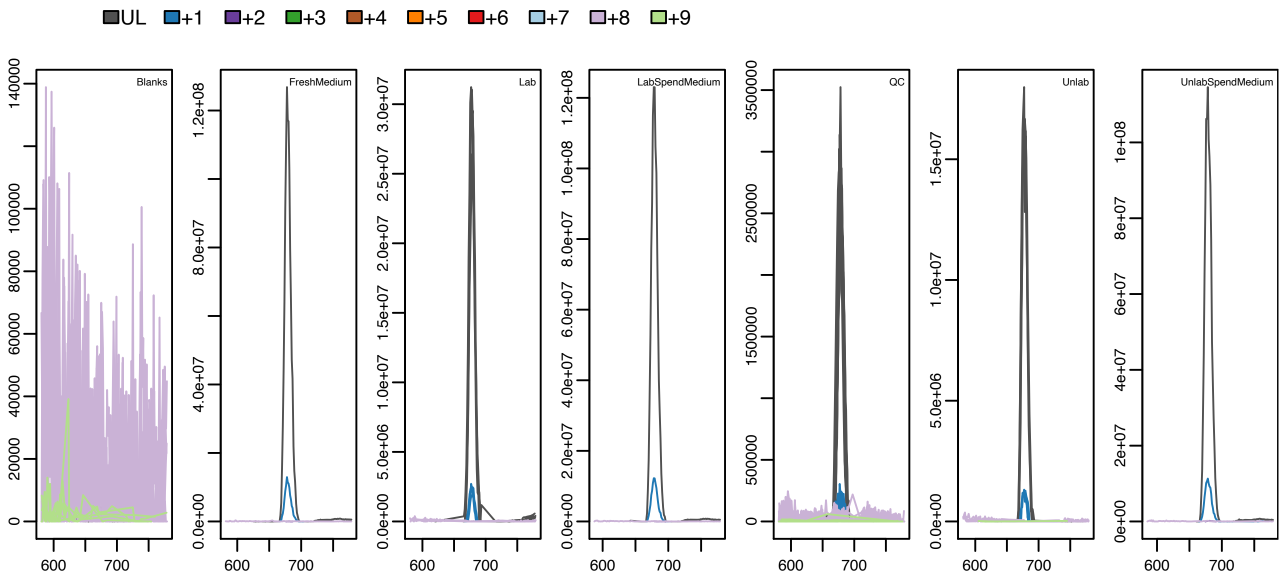

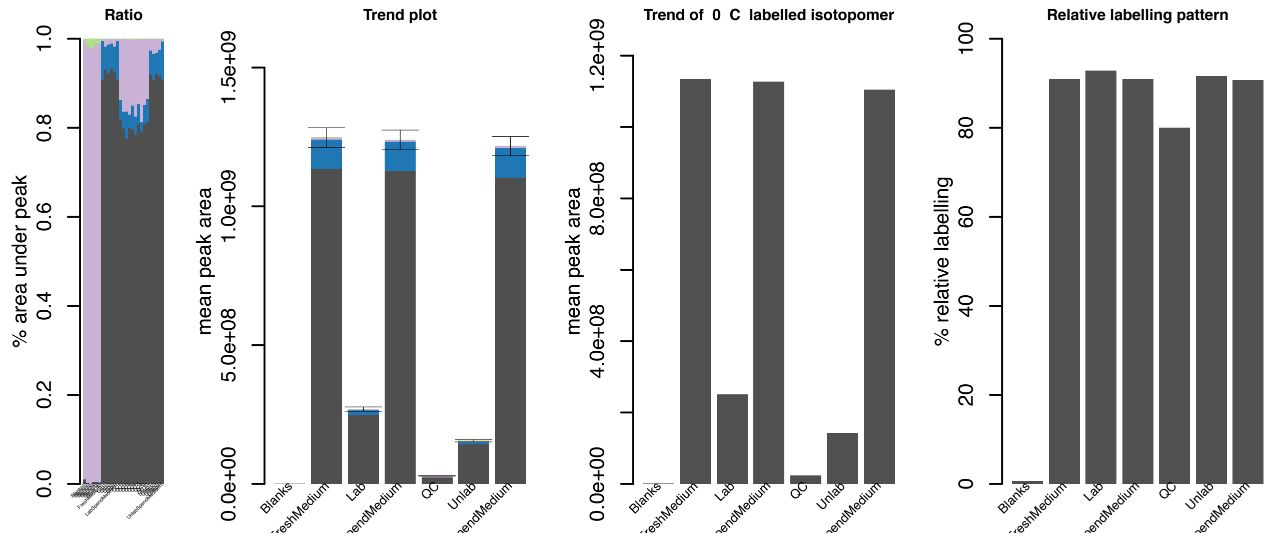

L−Valine

Formula: C5H11NO2 Mass: 117.079 Std.RT: 651 Ion: POS

G1

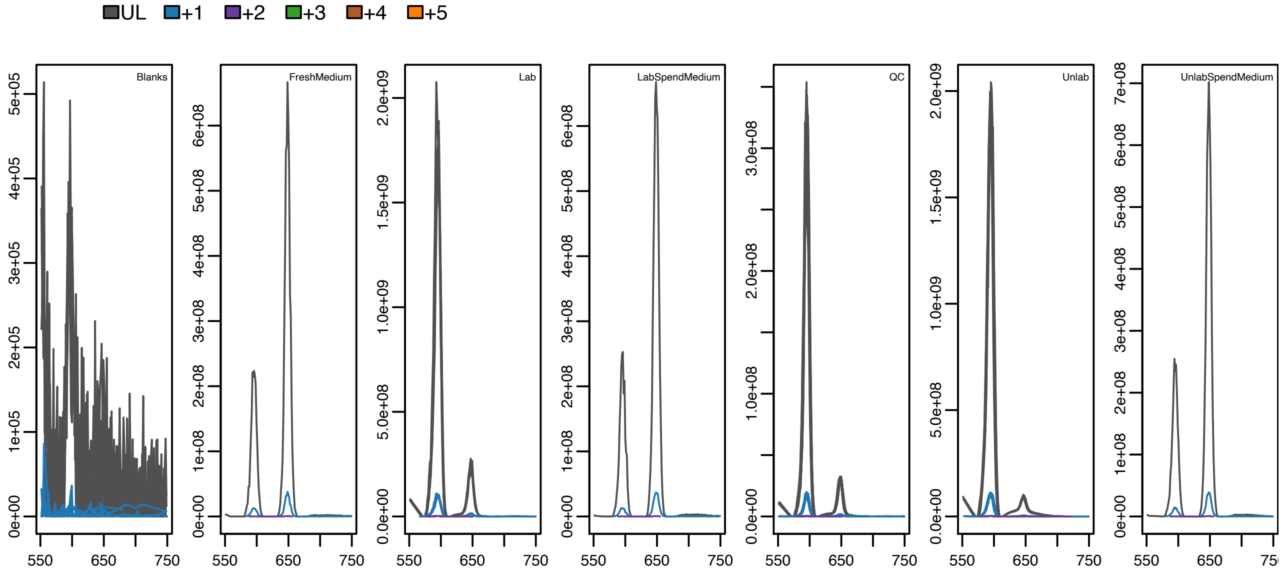

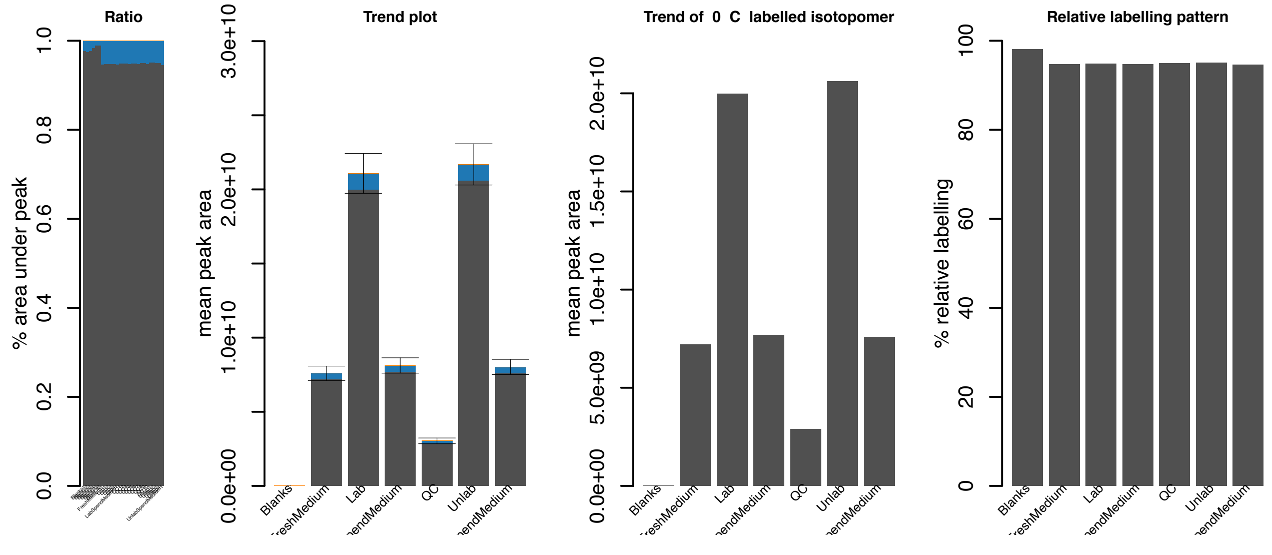

L−isoleucine

Formula: C6H13NO2   Mass: 131.095 Std.RT: 623.4 Ion: POS

G1

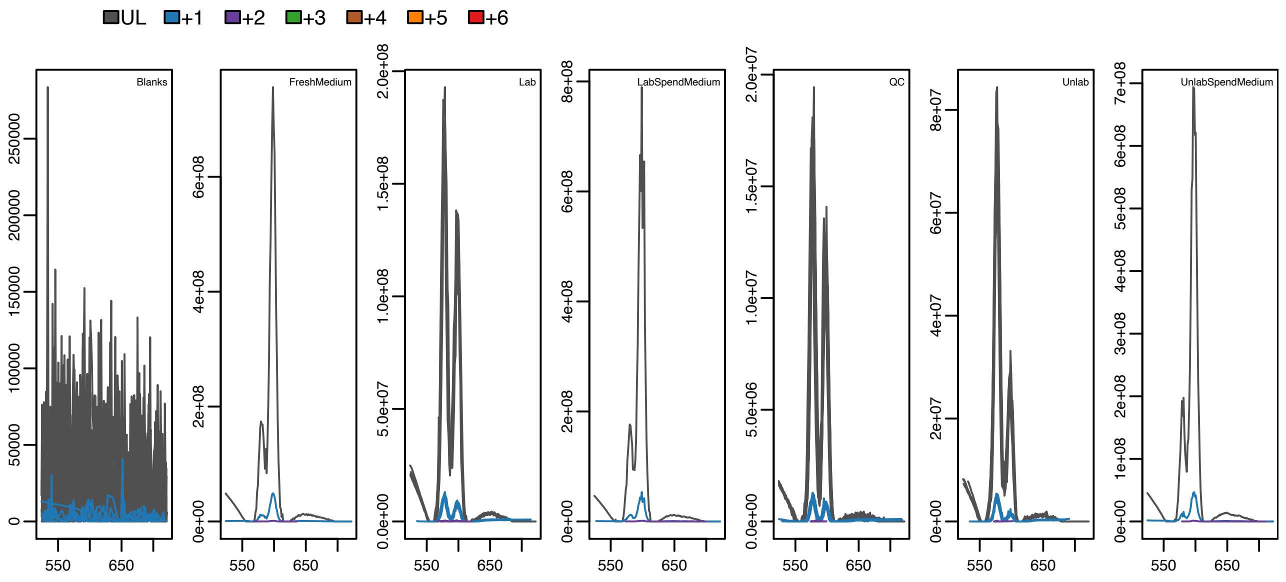

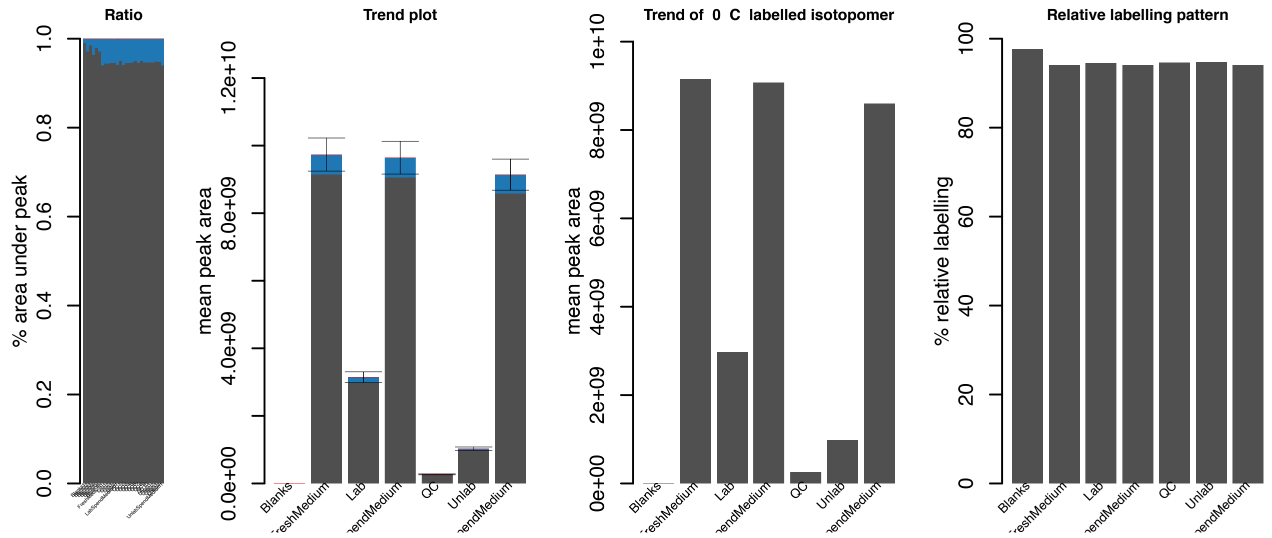

N−Acetylornithine

Formula: C7H14N2O3 Mass: 174.1 Std.RT: 735 Ion: POS

G1

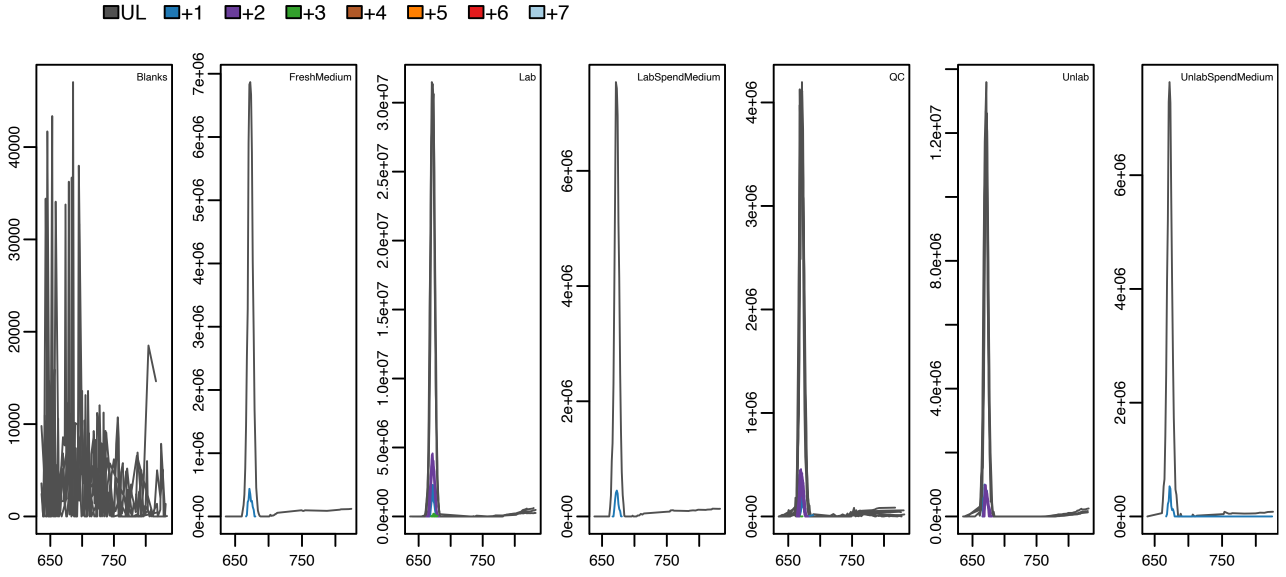

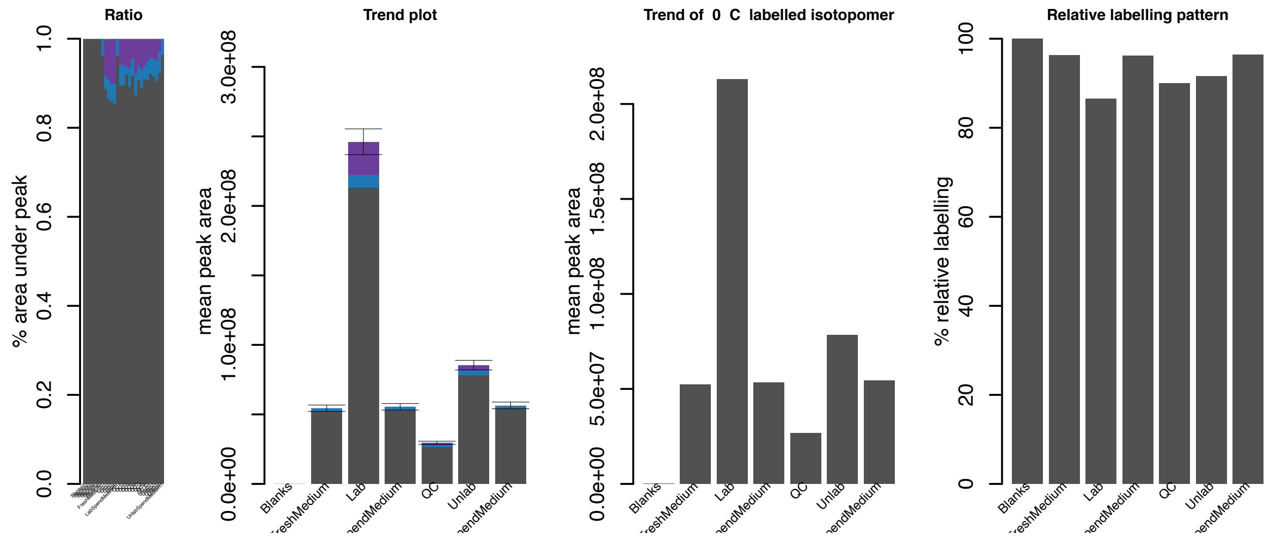

N2−Acetyl−L−lysine

Formula: C8H16N2O3   Mass: 188.116 Std.RT: 727.2 Ion: POS

G1

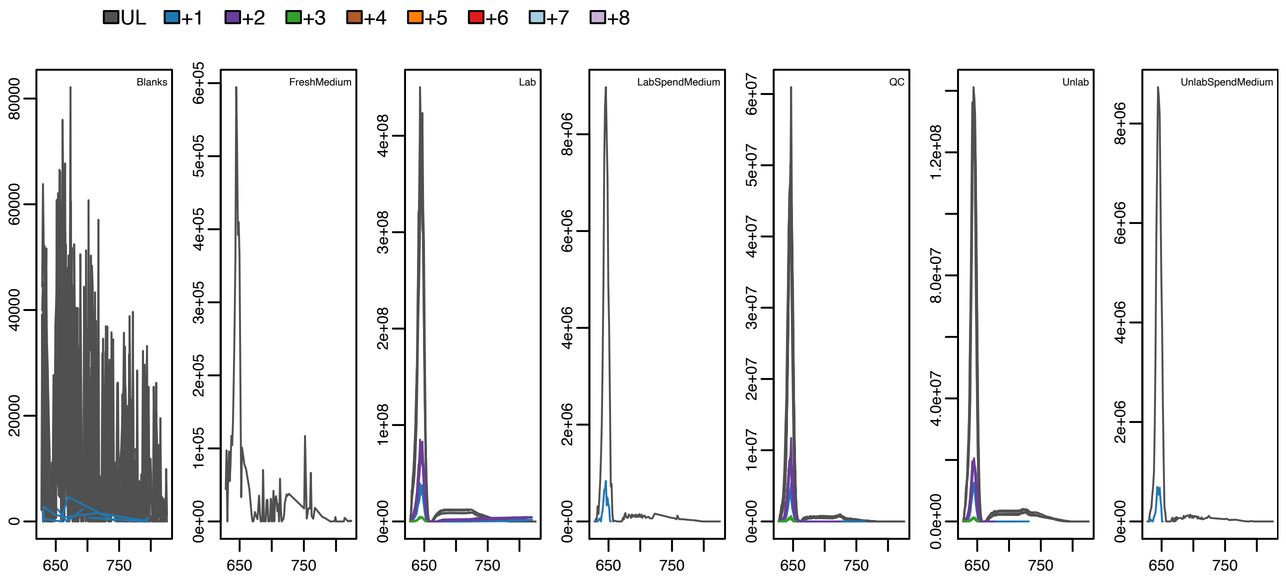

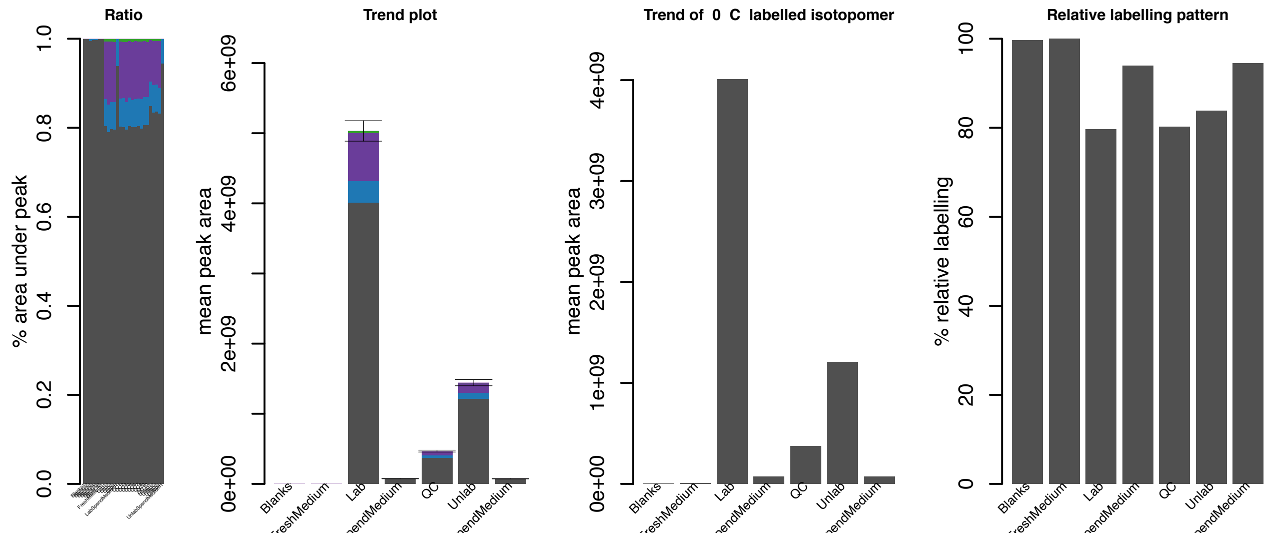

NAD+

Formula: C21H27N7O14P2 Mass: 663.109 Std.RT: 678 Ion: POS

G1

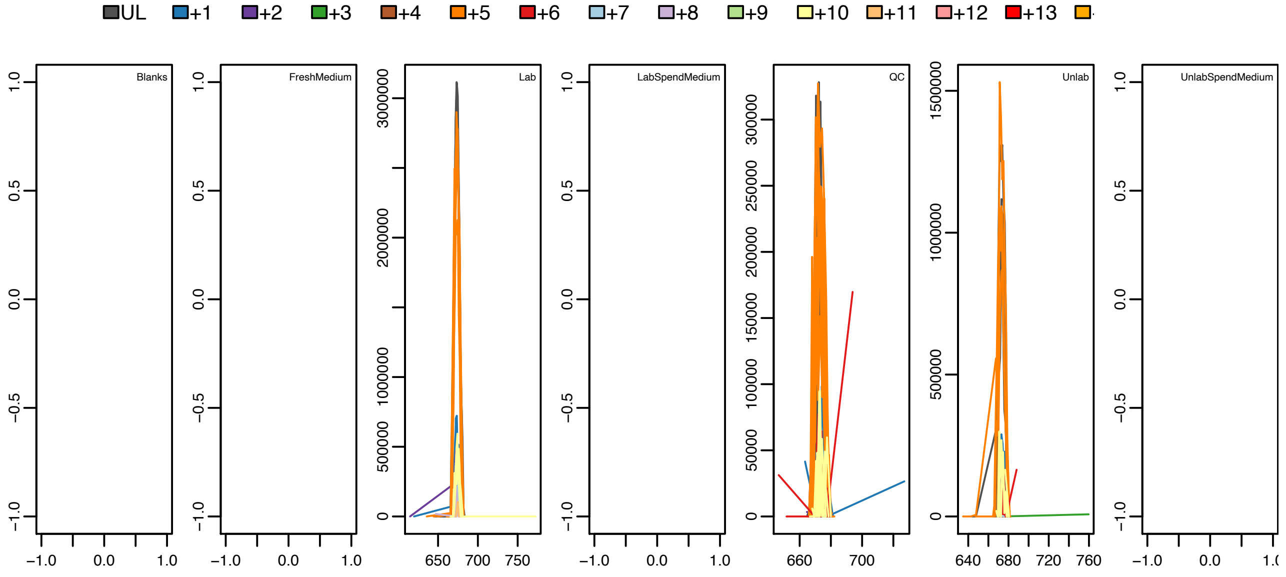

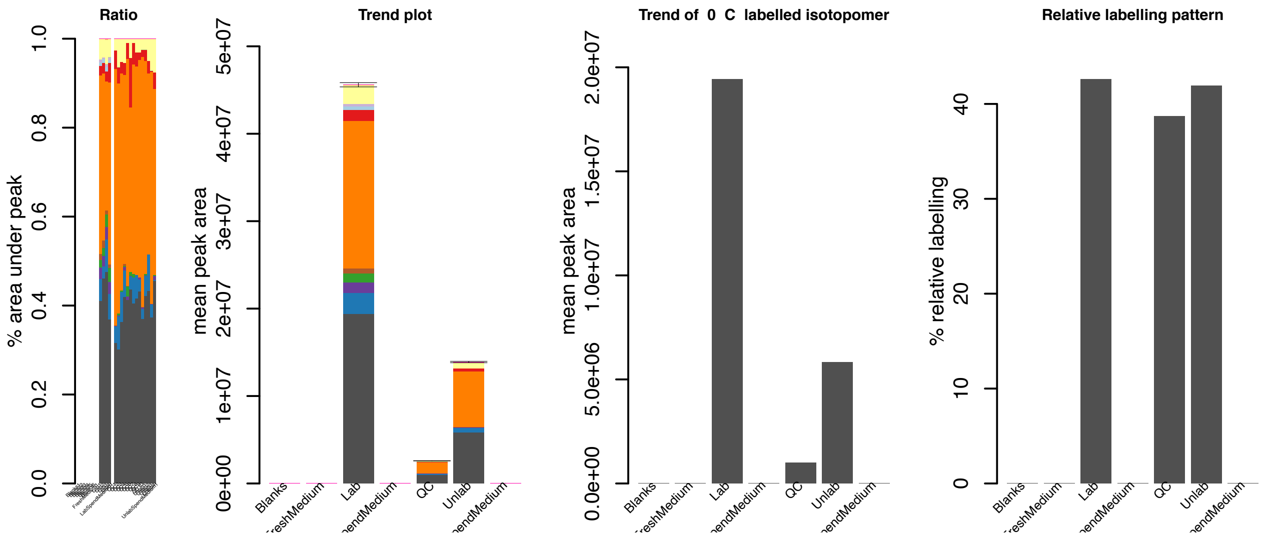

Nicotinamide

Formula: C6H6N2O Mass: 122.048 Std.RT: 389.4 Ion: POS

G1

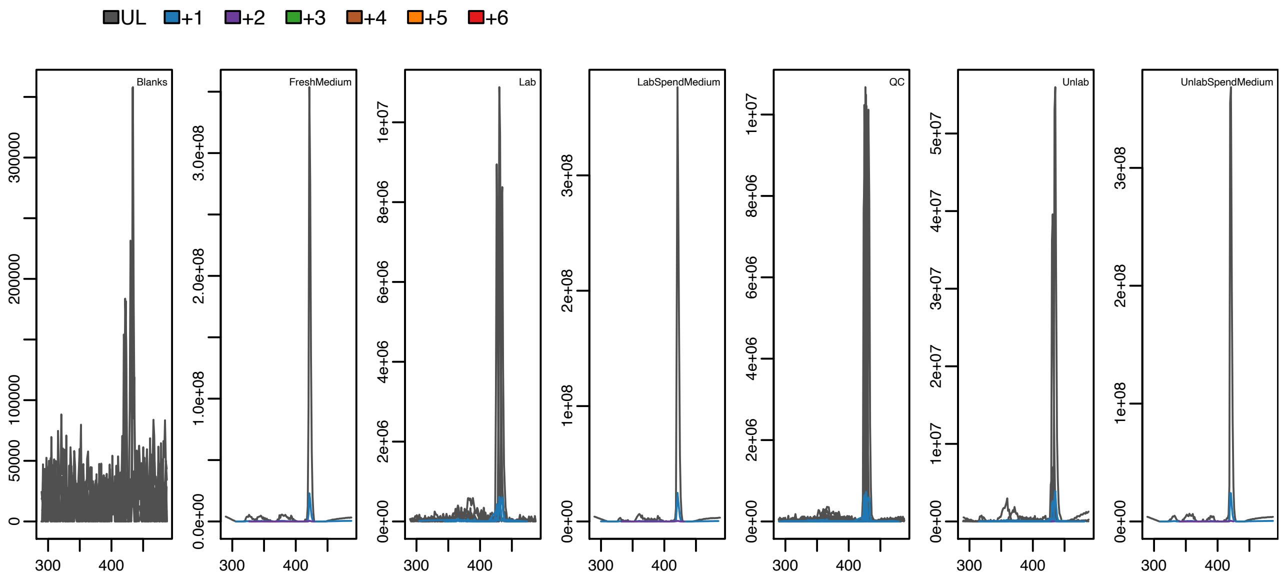

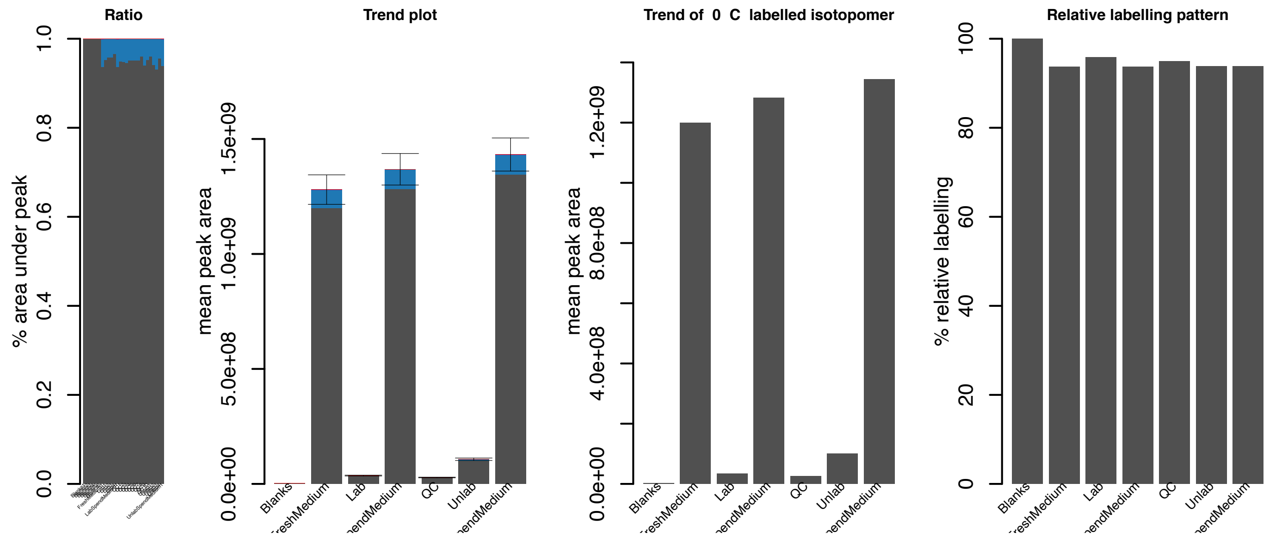

Nicotinate

G1

Formula: C6H5NO2 Mass: 123.032 Std.RT: 435 Ion: POS

UL +1 +2 +3 +4 +5 +6

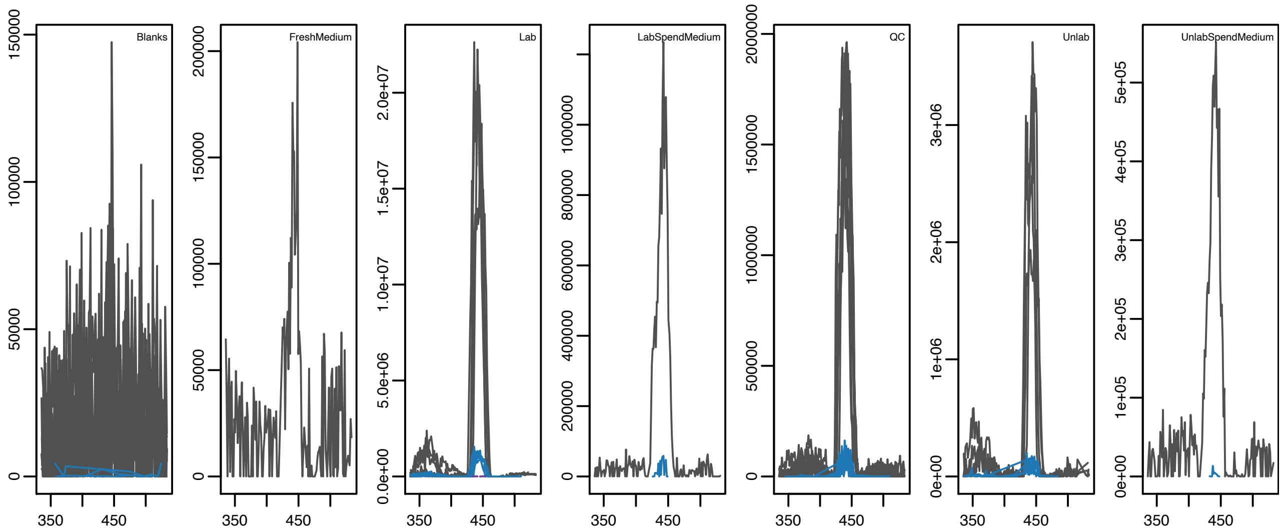

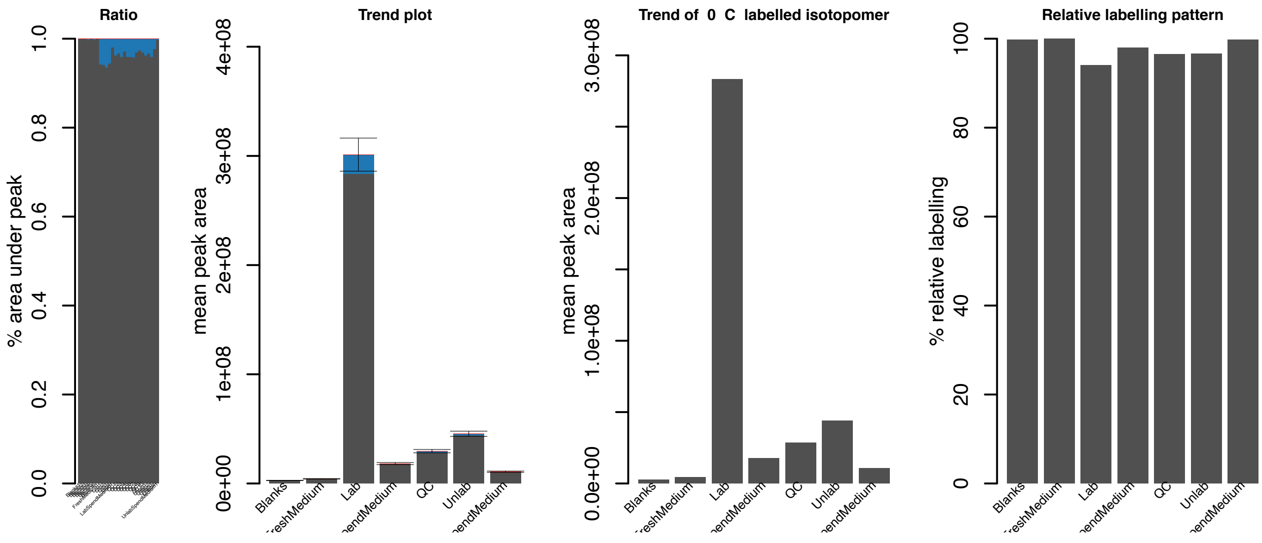

O−Acetyl−L−serine

Formula: C5H9NO4 Mass: 147.053 Std.RT: 701.4 Ion: POS

G1

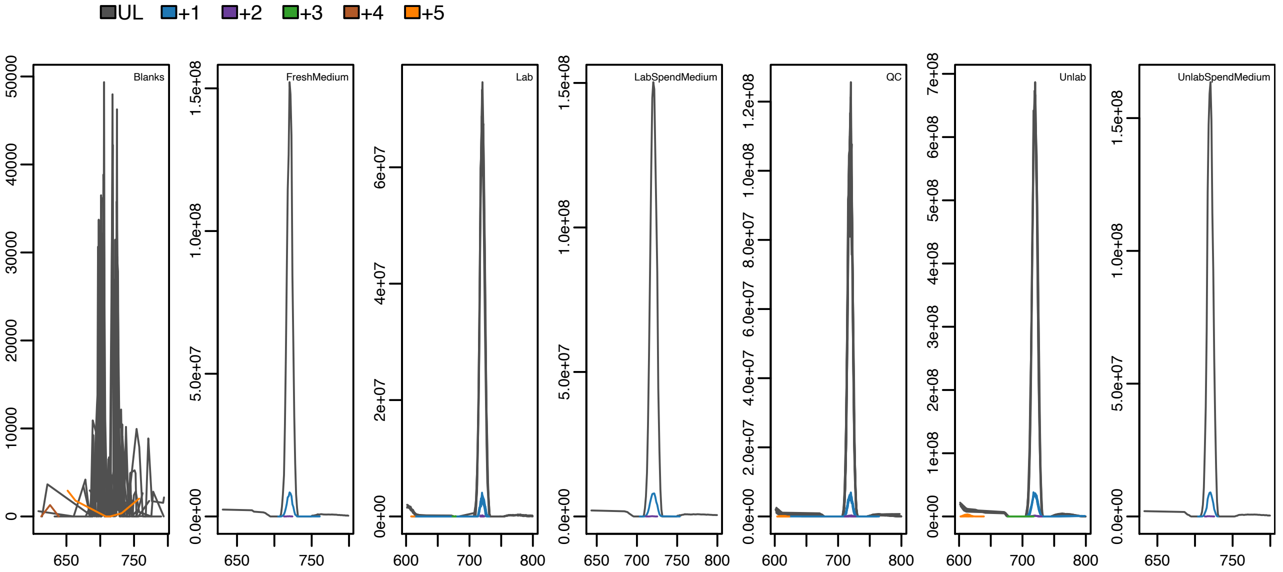

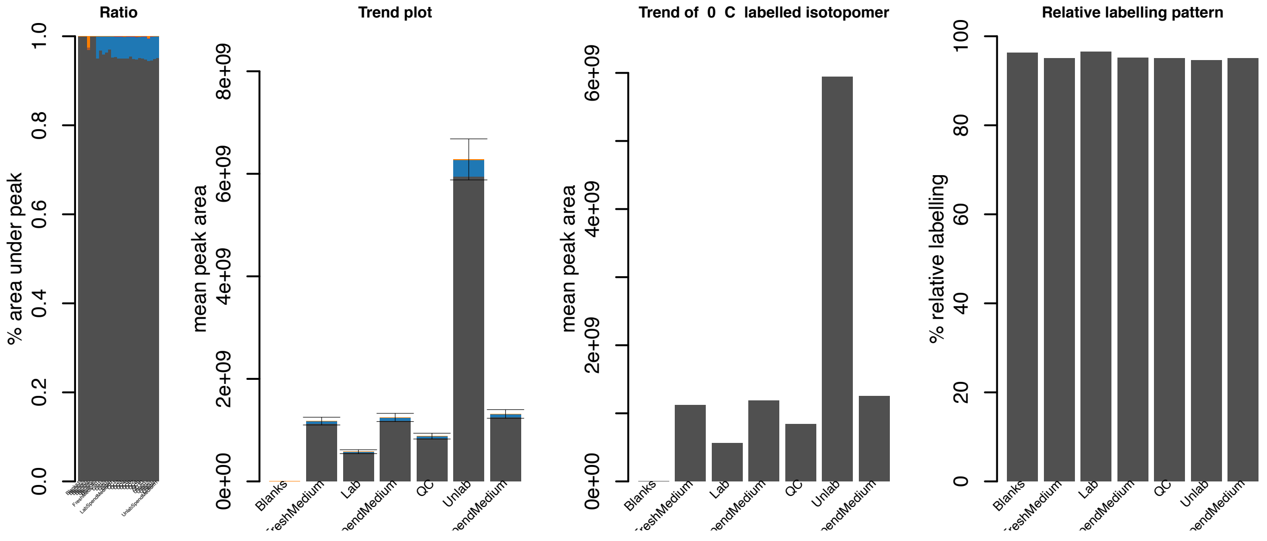

O−Acetylcarnitine

Formula: C9H17NO4 Mass: 203.116 Std.RT: 573.6 Ion: POS

G1

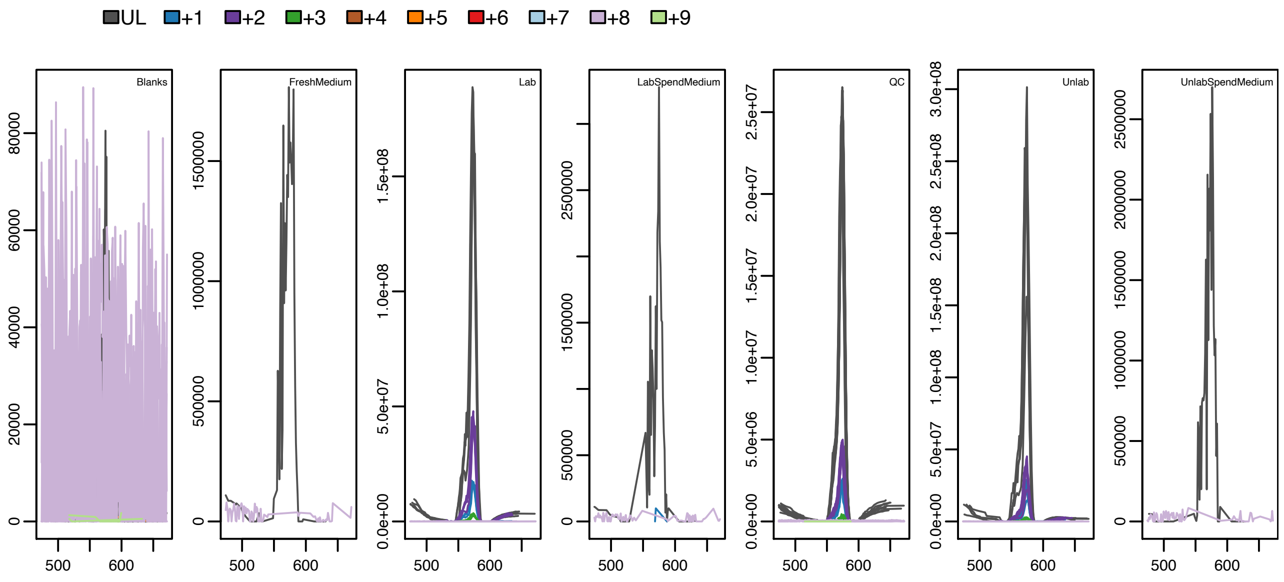

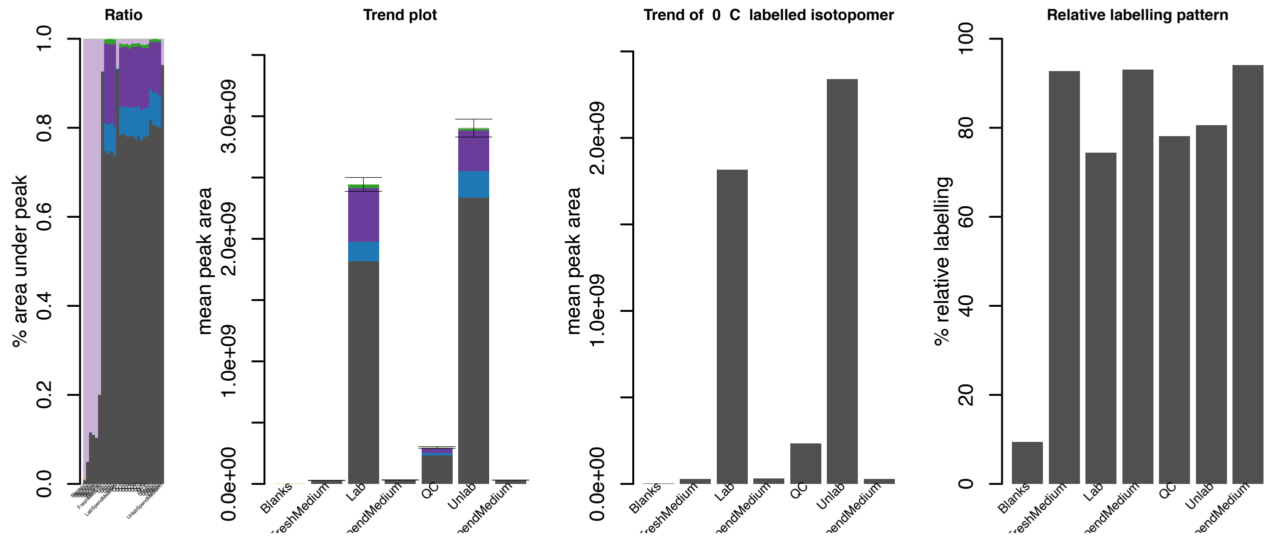

Pantothenate

Formula: C9H17NO5 Mass: 219.111 Std.RT: 487.2 Ion: POS

G1

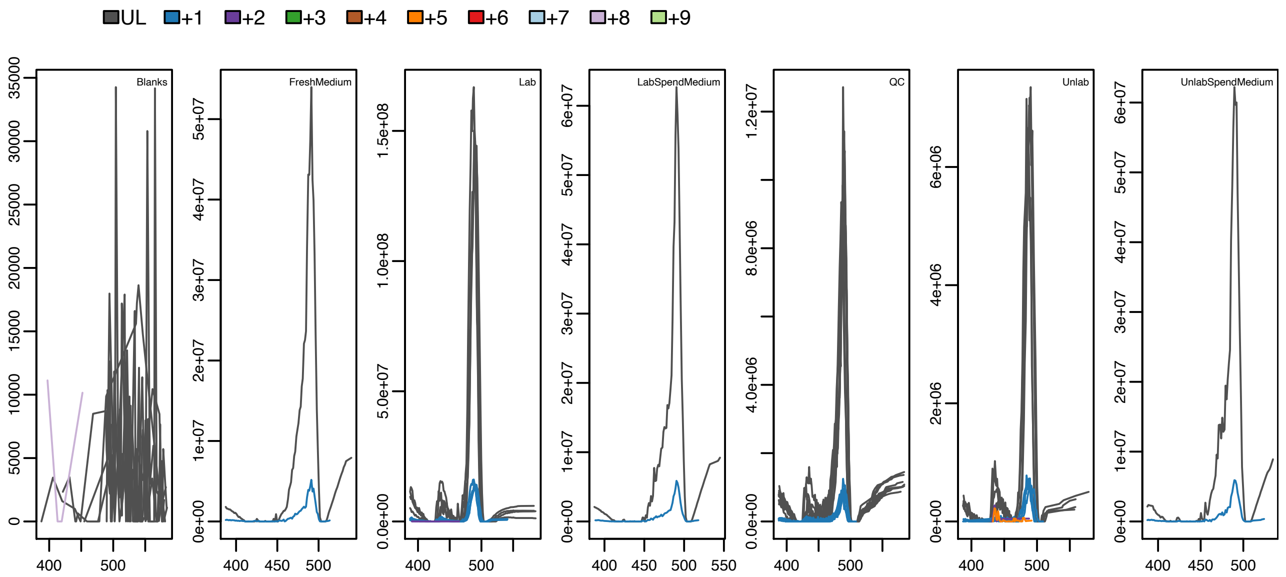

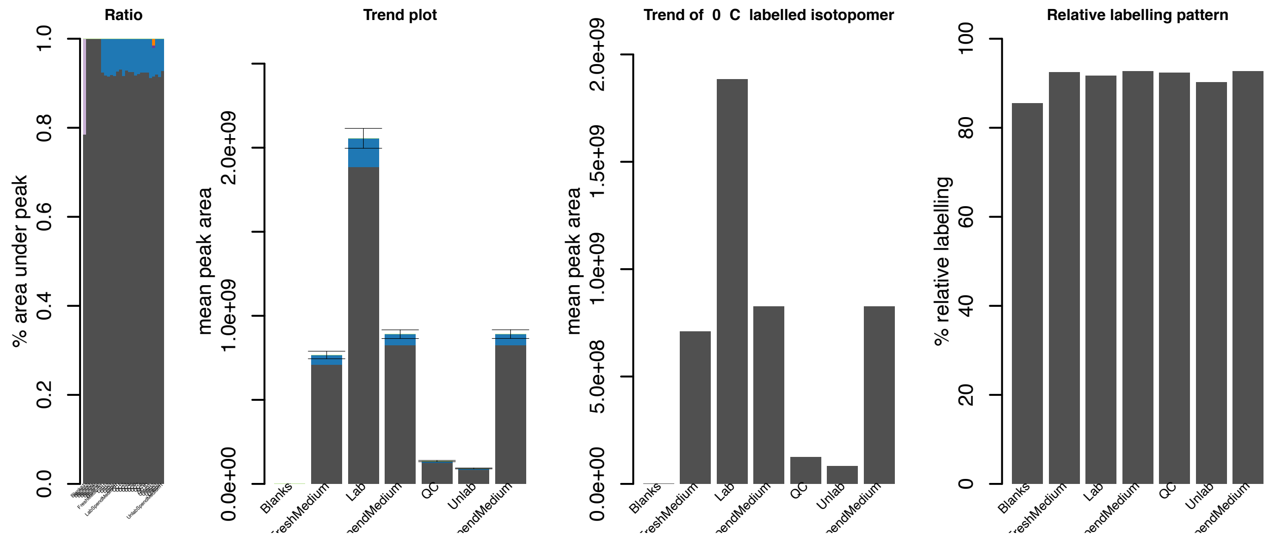

Picolinic acid

Formula: C6H5NO2   Mass: 123.032 Std.RT: 508.2 Ion: POS

G1

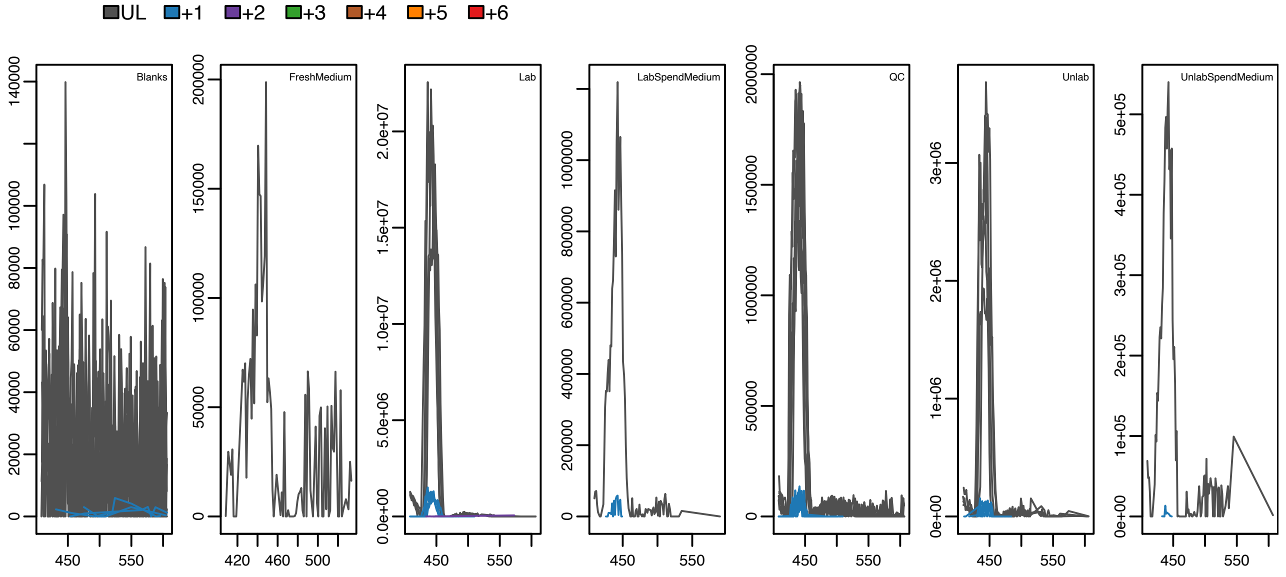

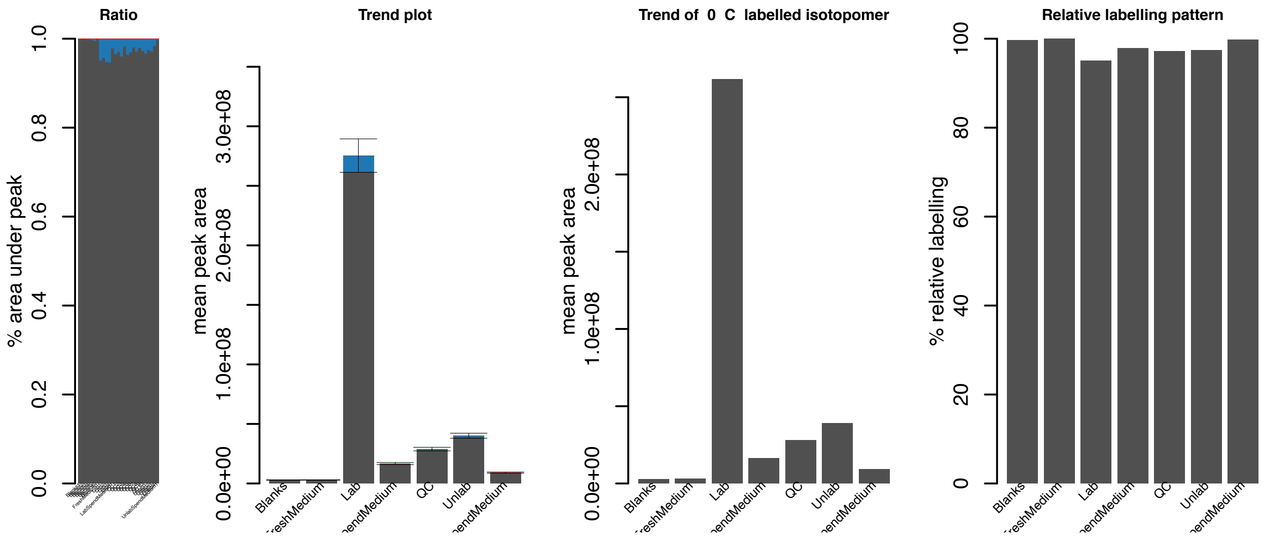

Pyridoxal

G1

Formula: C8H9NO3 Mass: 167.058 Std.RT: 454.8 Ion: POS

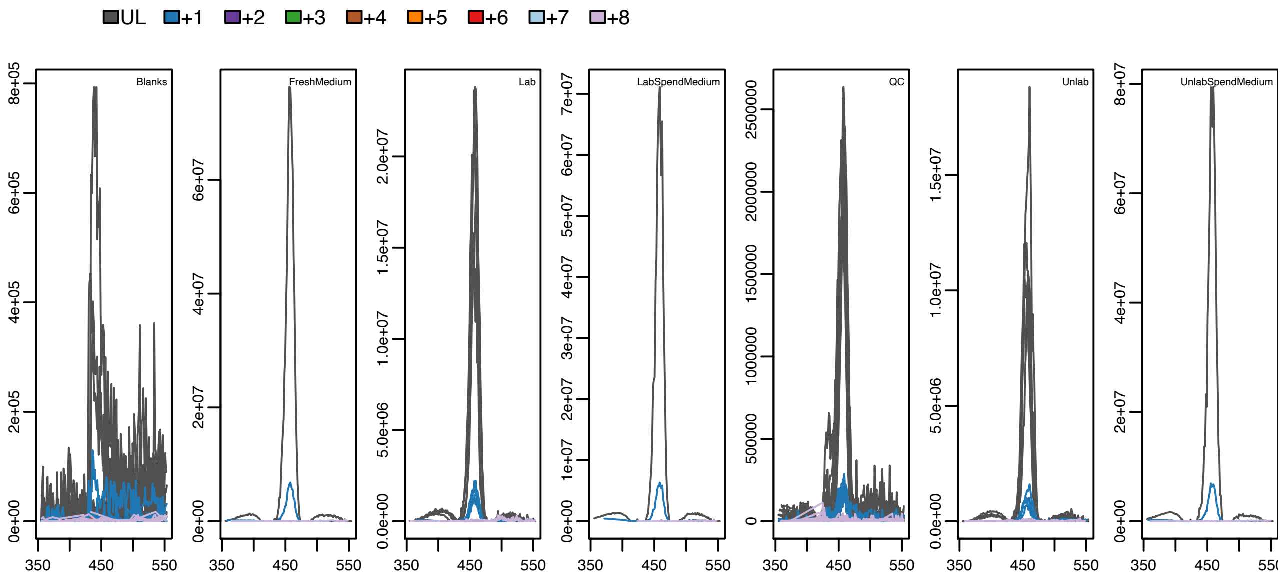

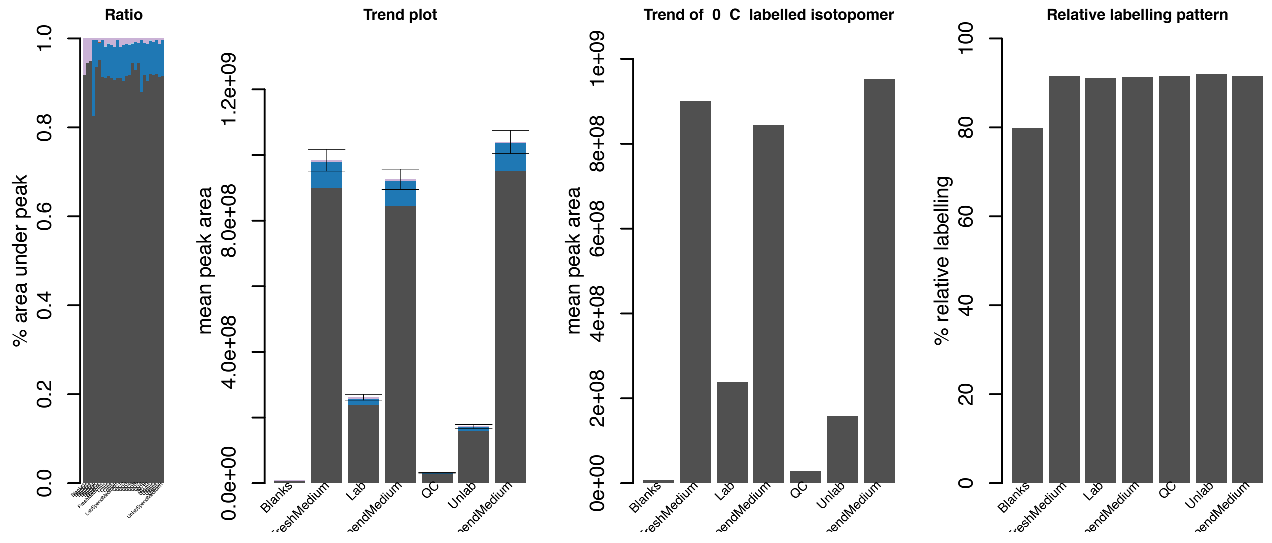

UMP

Formula: C9H13N2O9P Mass: 324.036 Std.RT: 694.8 Ion: POS

G1

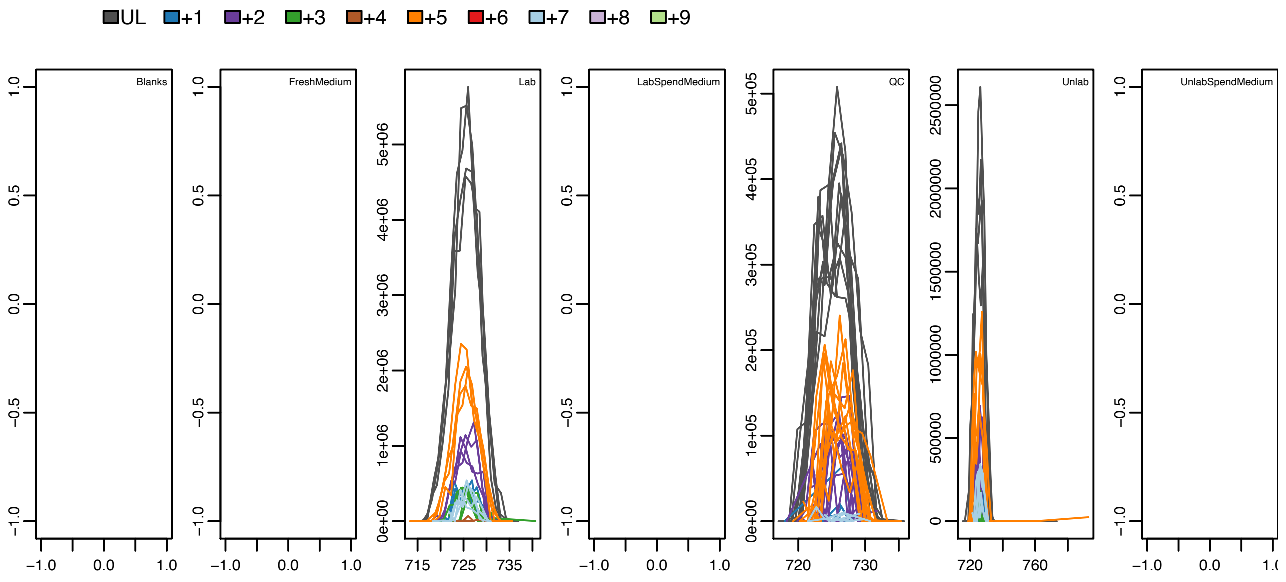

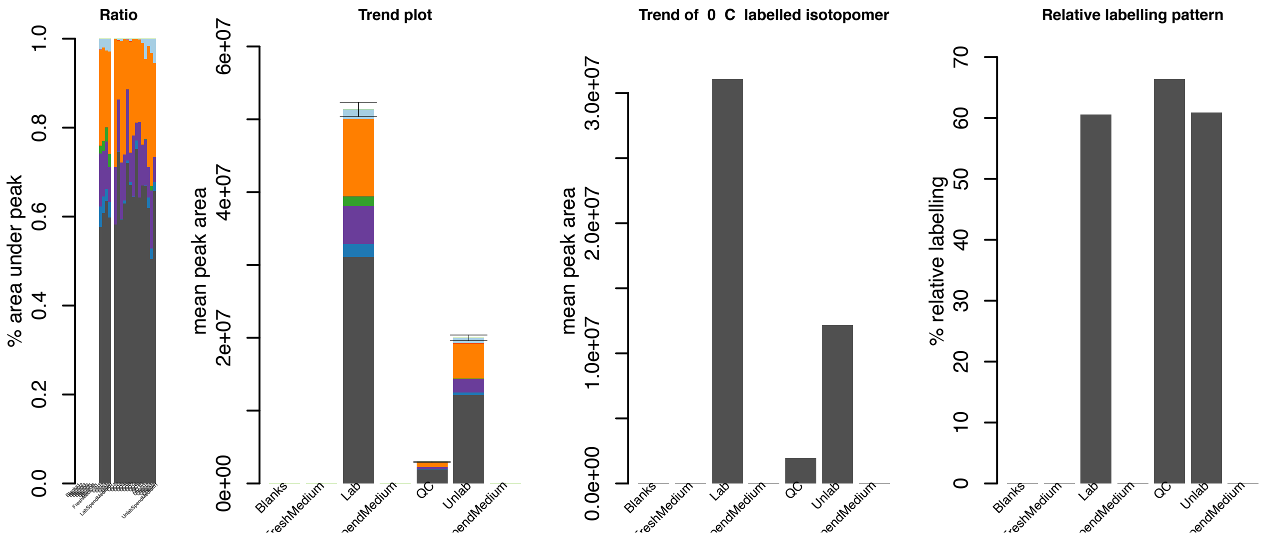

Uracil

Formula: C4H4N2O2   Mass: 112.027 Std.RT: 483.6 Ion: POS

G1

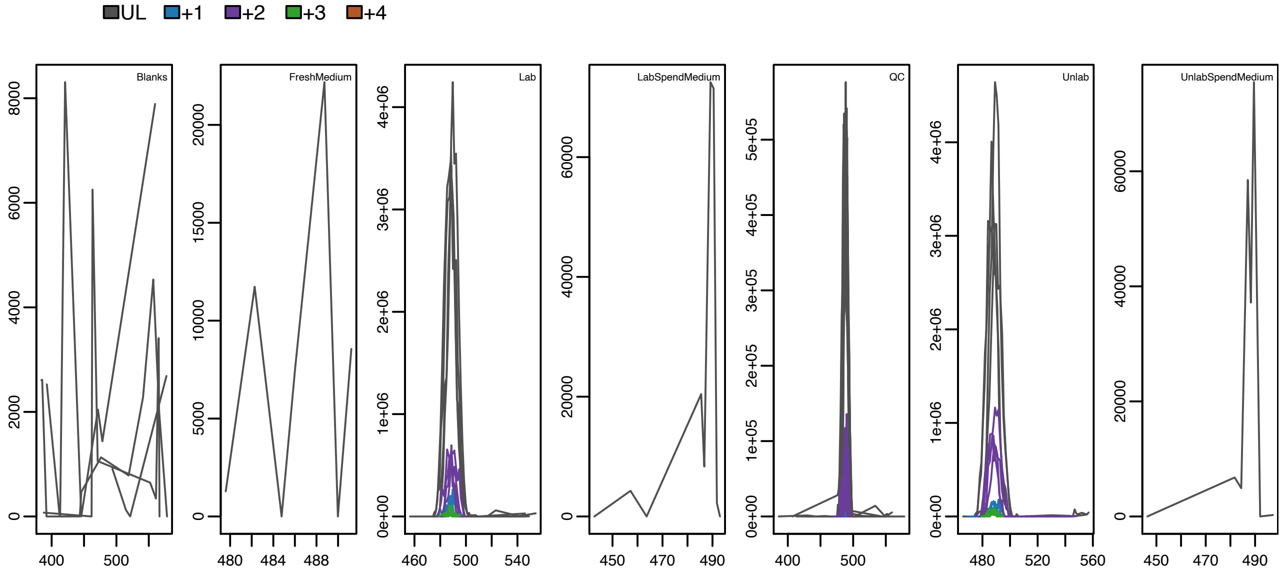

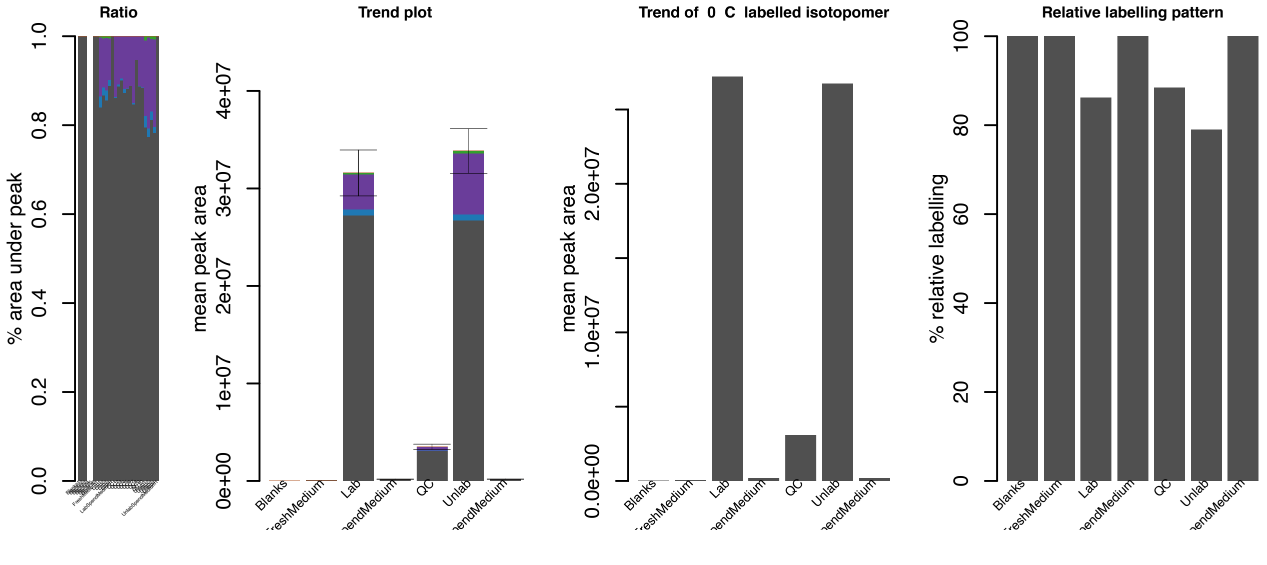

cytosine

Formula: C4H5N3O Mass: 111.043 Std.RT: 612.6 Ion: POS

G1

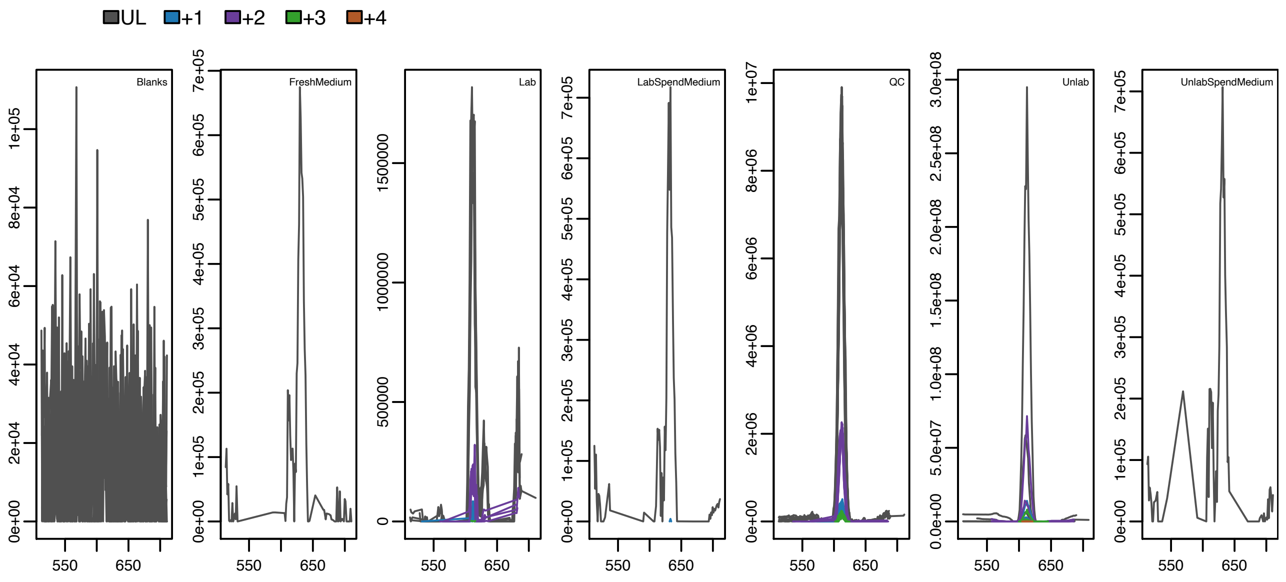

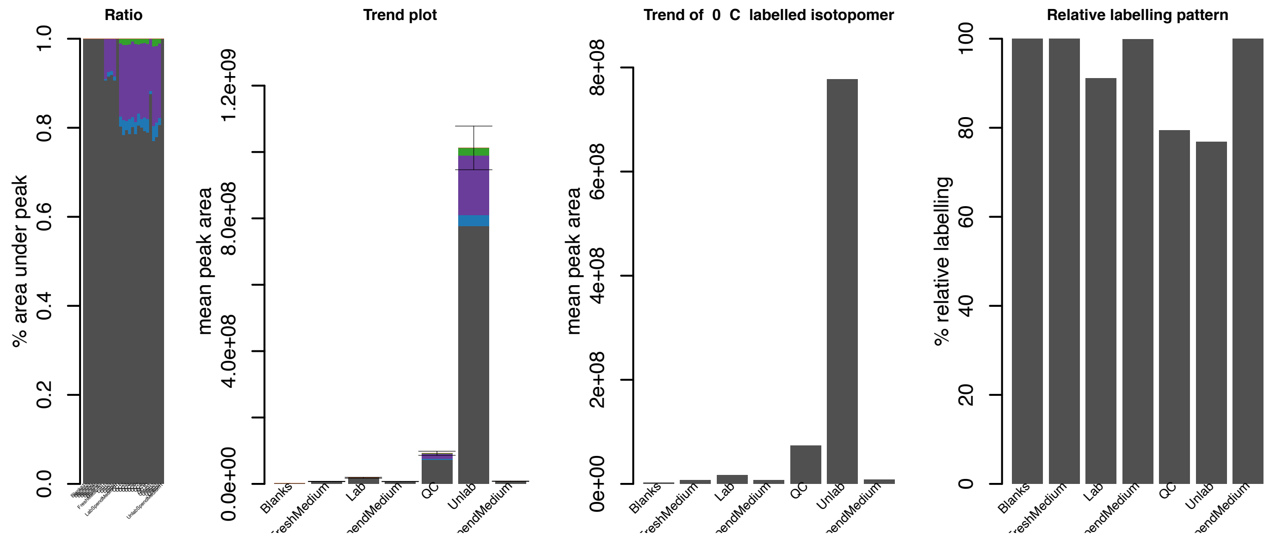

dAMP

Formula: C10H14N5O6P Mass: 331.068 Std.RT: 632.4 Ion: POS

G1

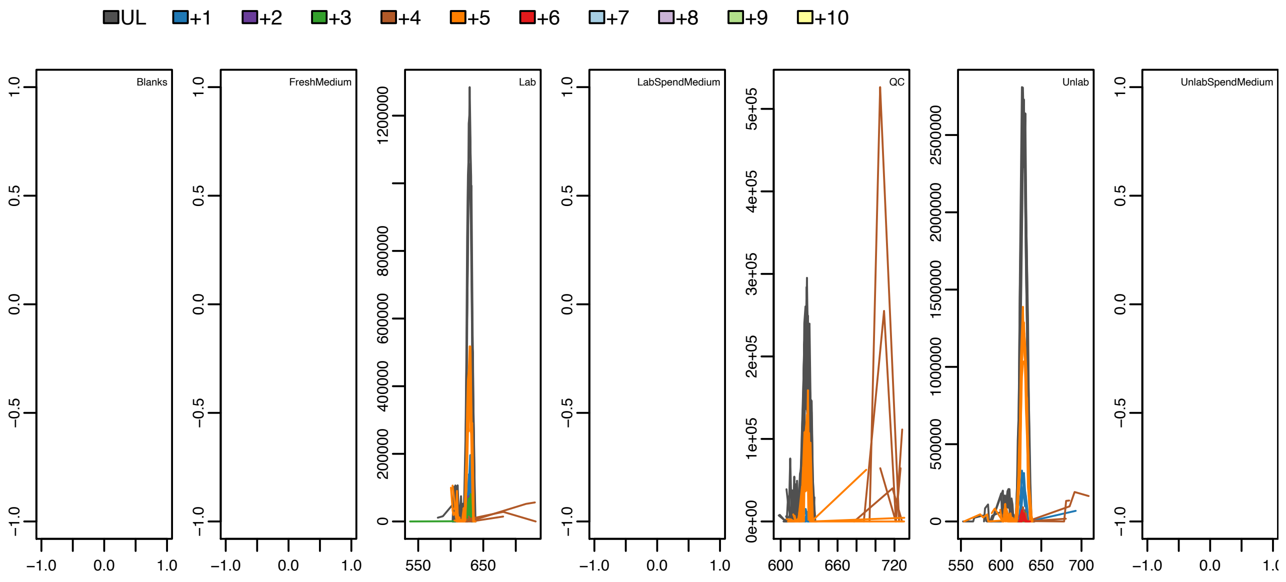

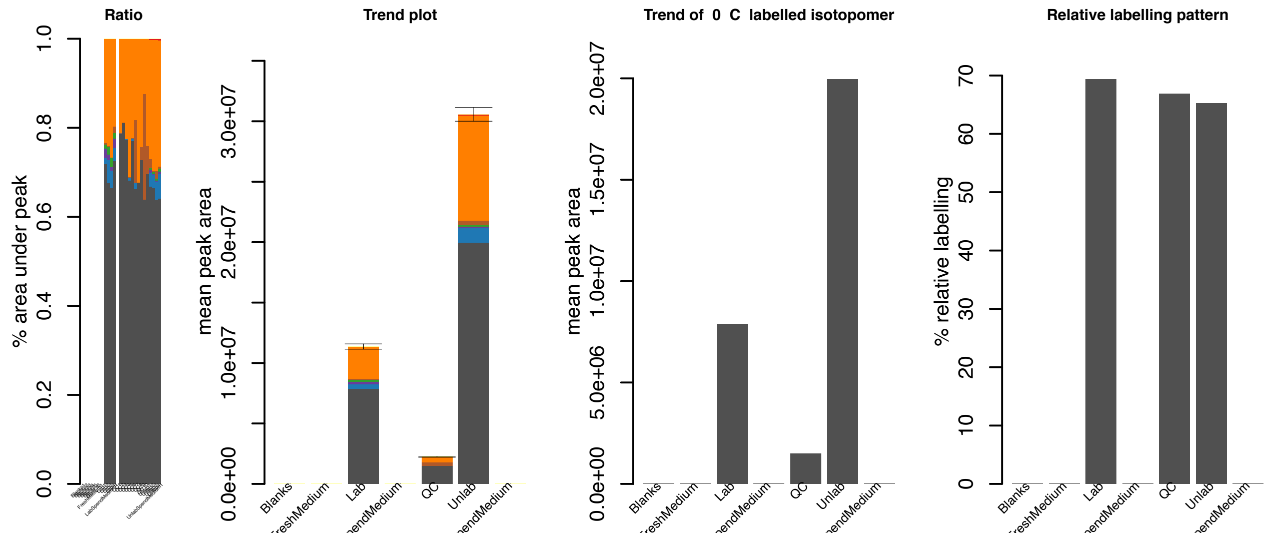

sn−Glycerol 3−phosphate

Formula: C3H9O6P Mass: 172.014 Std.RT: 720.6 Ion: POS

G1

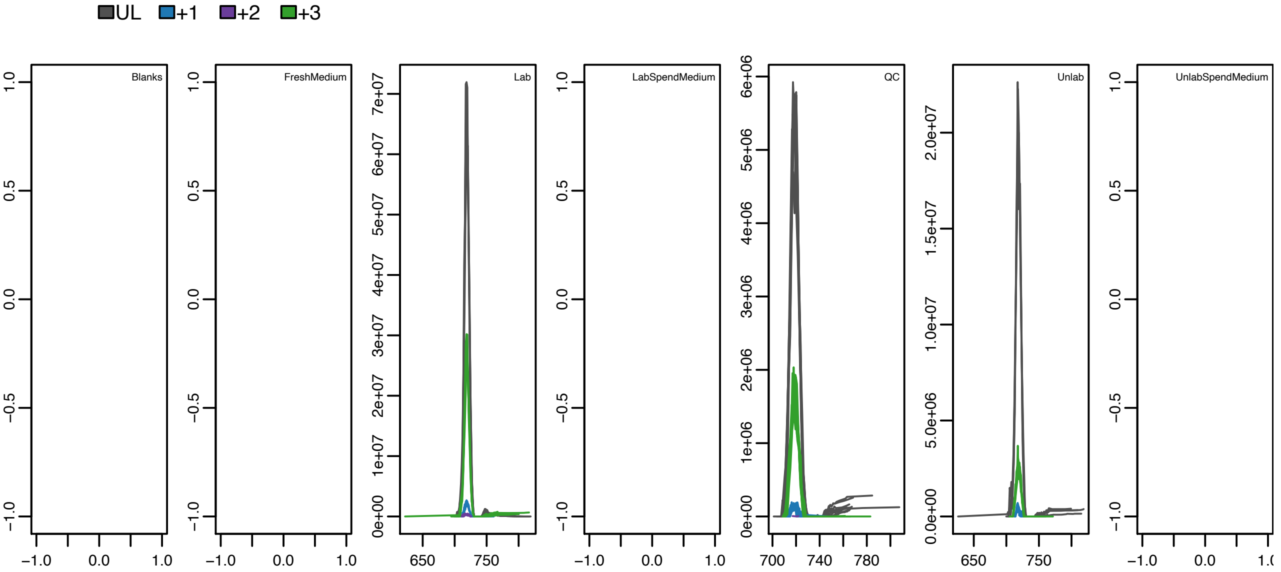

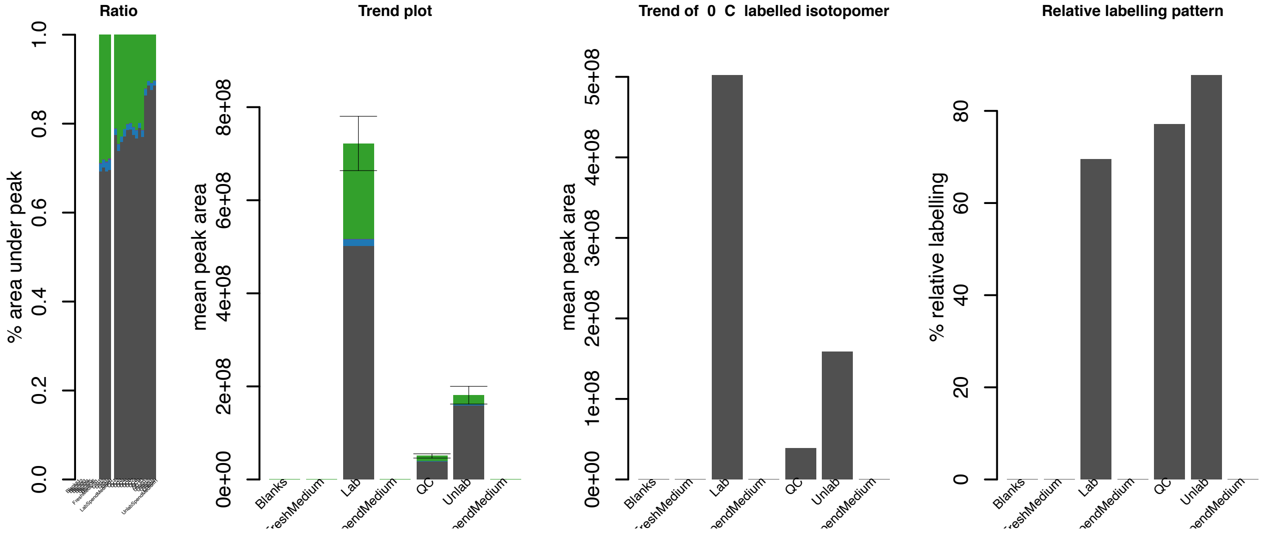

sn−glycero−3−Phosphocholine

Formula: C8H20NO6P Mass: 257.103 Std.RT: 700.8 Ion: POS

G1

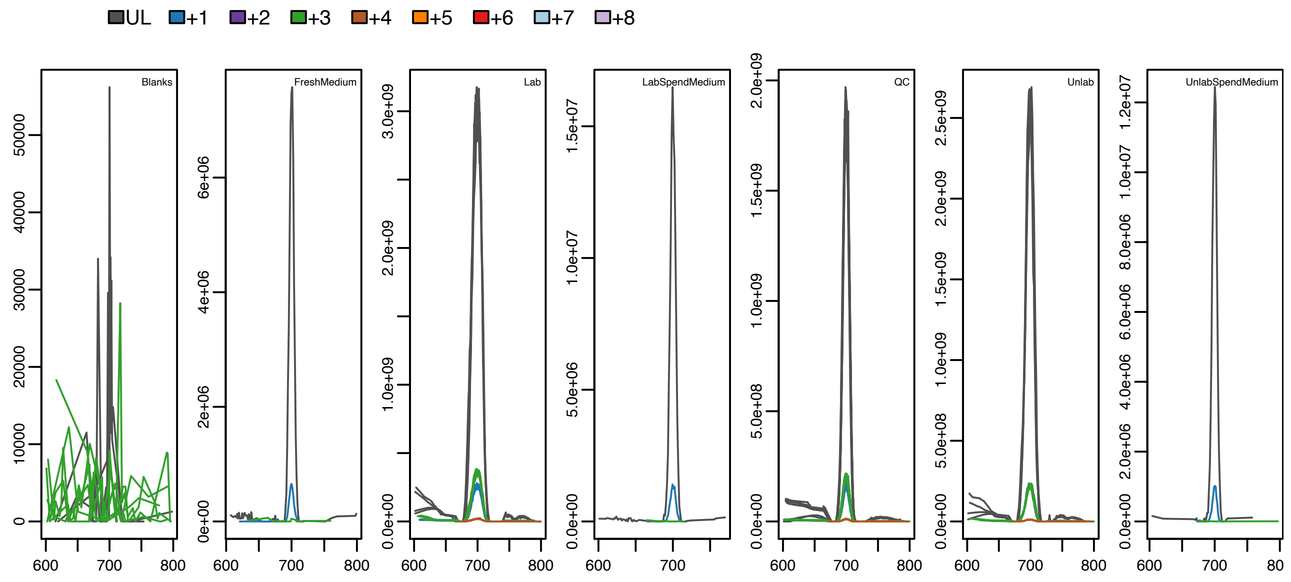

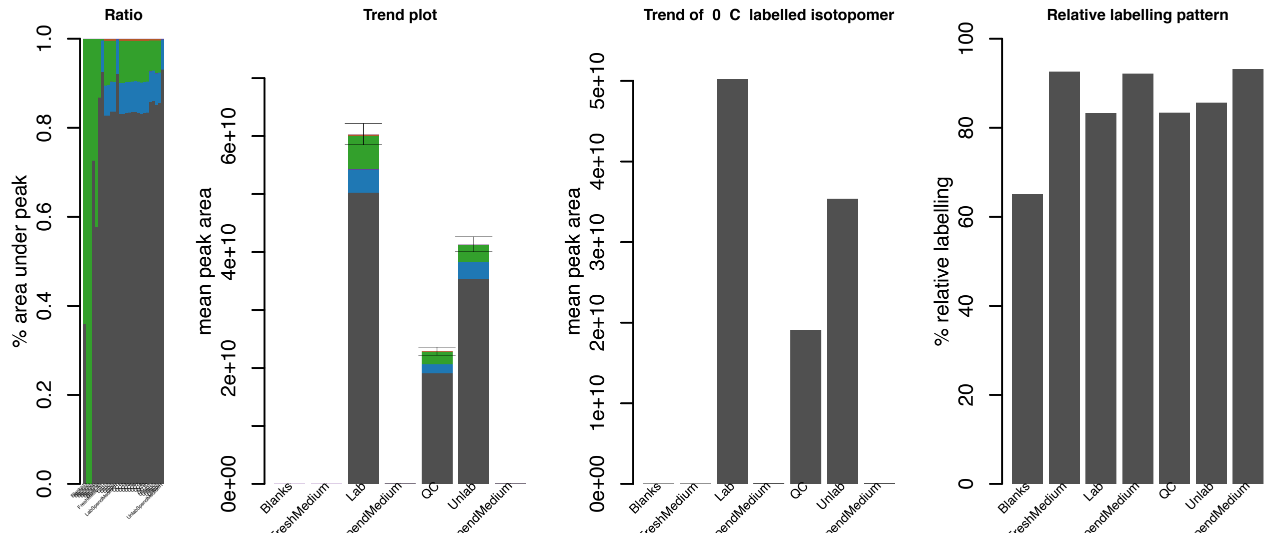

thymine

Formula: C5H6N2O2 Mass: 126.043 Std.RT: 418.2 Ion: POS

G1

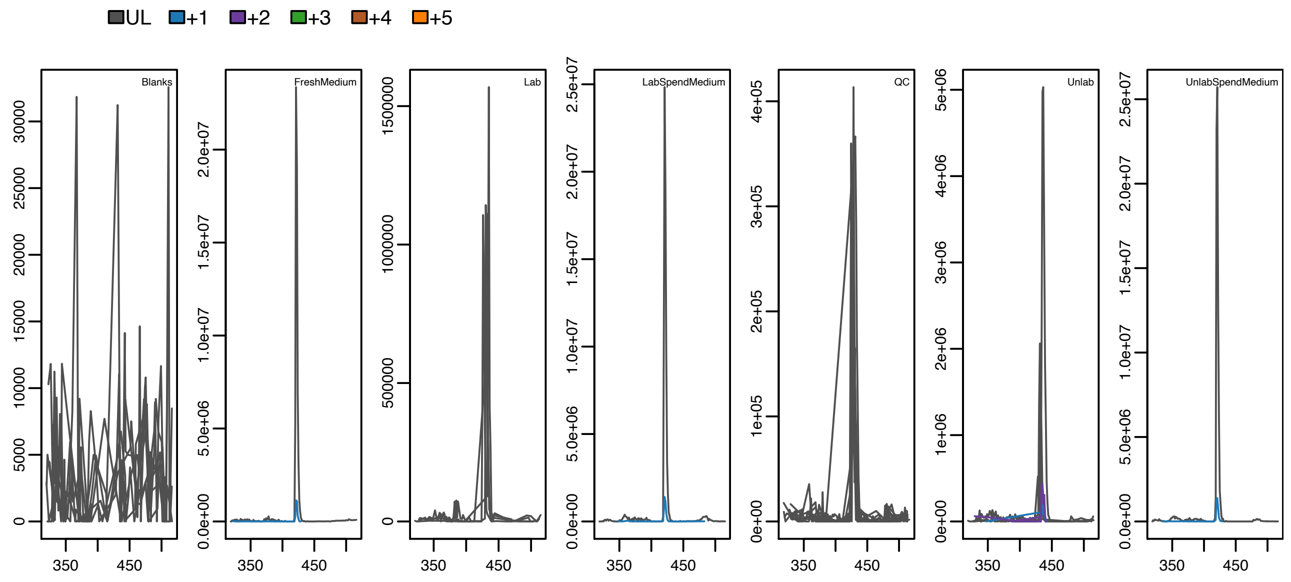

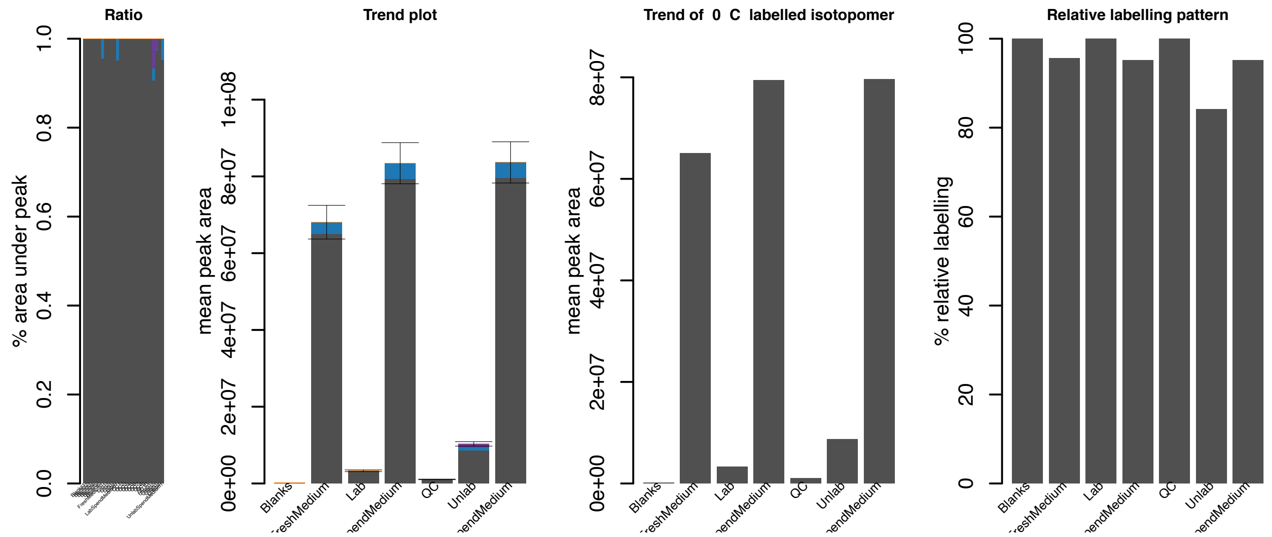

trans−4−Hydroxy−L−proline

Formula: C5H9NO3 Mass: 131.058 Std.RT: 720.6 Ion: POS

G1

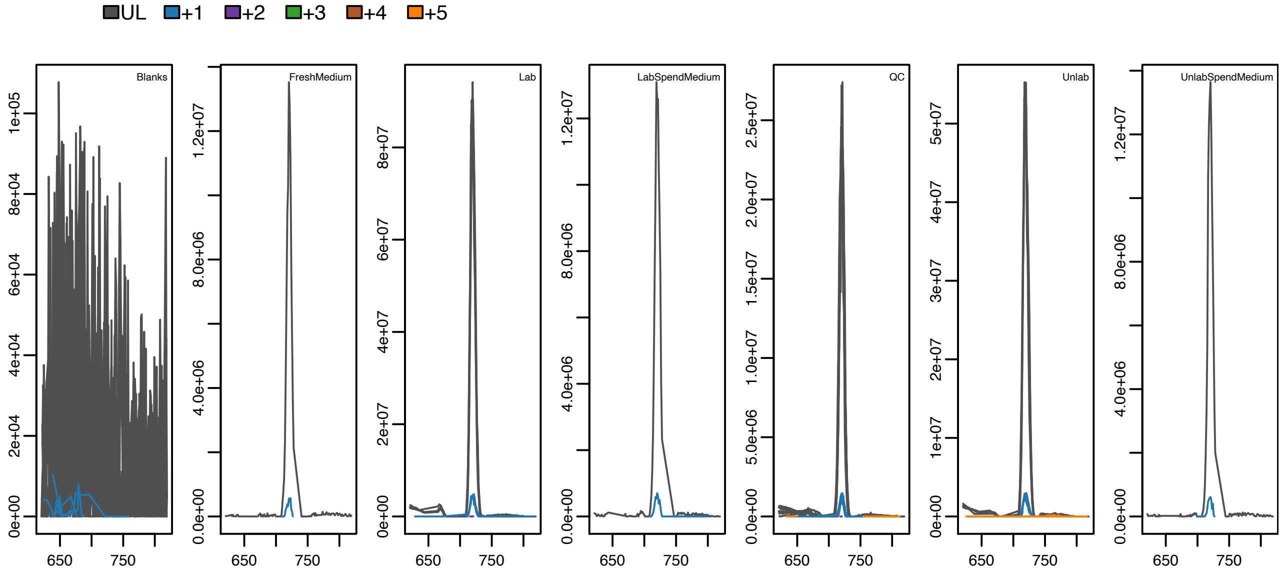

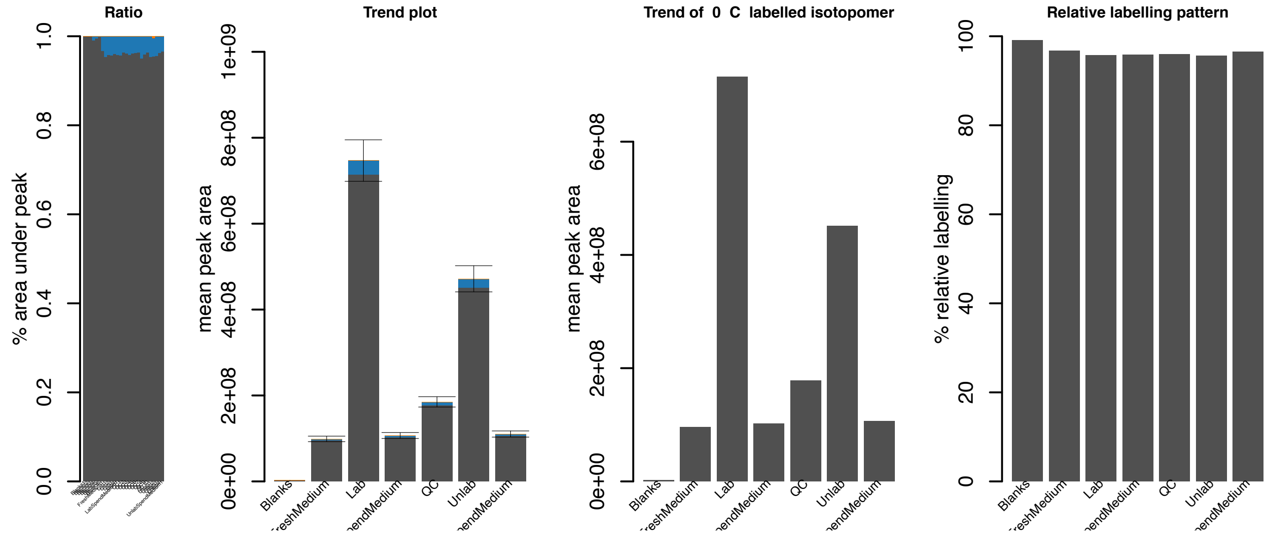

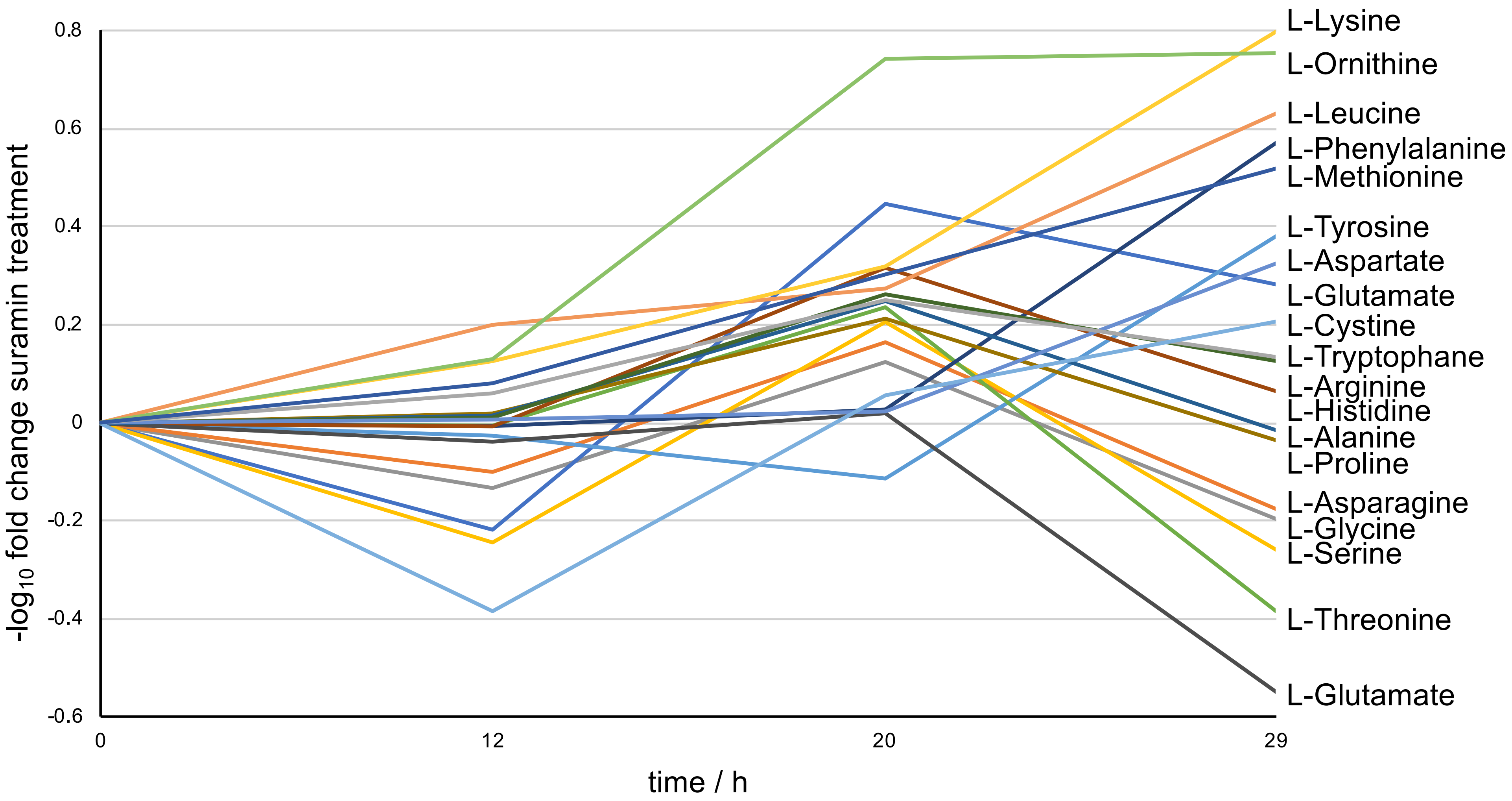
-log10 fold change suramin treatment
0.8
0.6
0.4
0.2
0
-0.2
-0.4
-0.6
0
12
20
29
time / h
L-Lysine
L-Ornithine
L-Leucine
L-Phenylalanine
L-Methionine
L-Tyrosine
L-Aspartate
L-Glutamate
L-Cystine
L-Tryptophane
L-Arginine
L-Histidine
L-Alanine
L-Proline
L-Asparagine
L-Glycine
L-Serine
L-Threonine
L-Glutamate

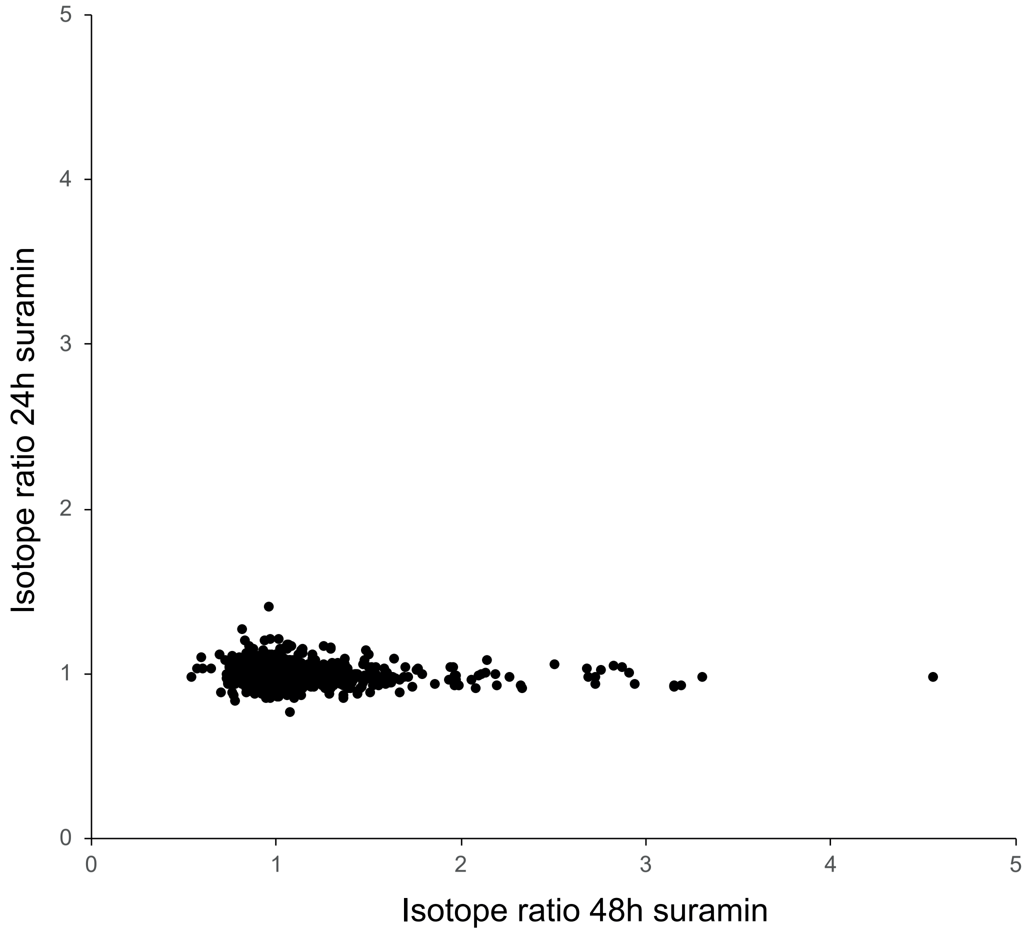
Isotope ratio 24h suramin
Isotope ratio 48h suramin
0
1
2
3
4
5

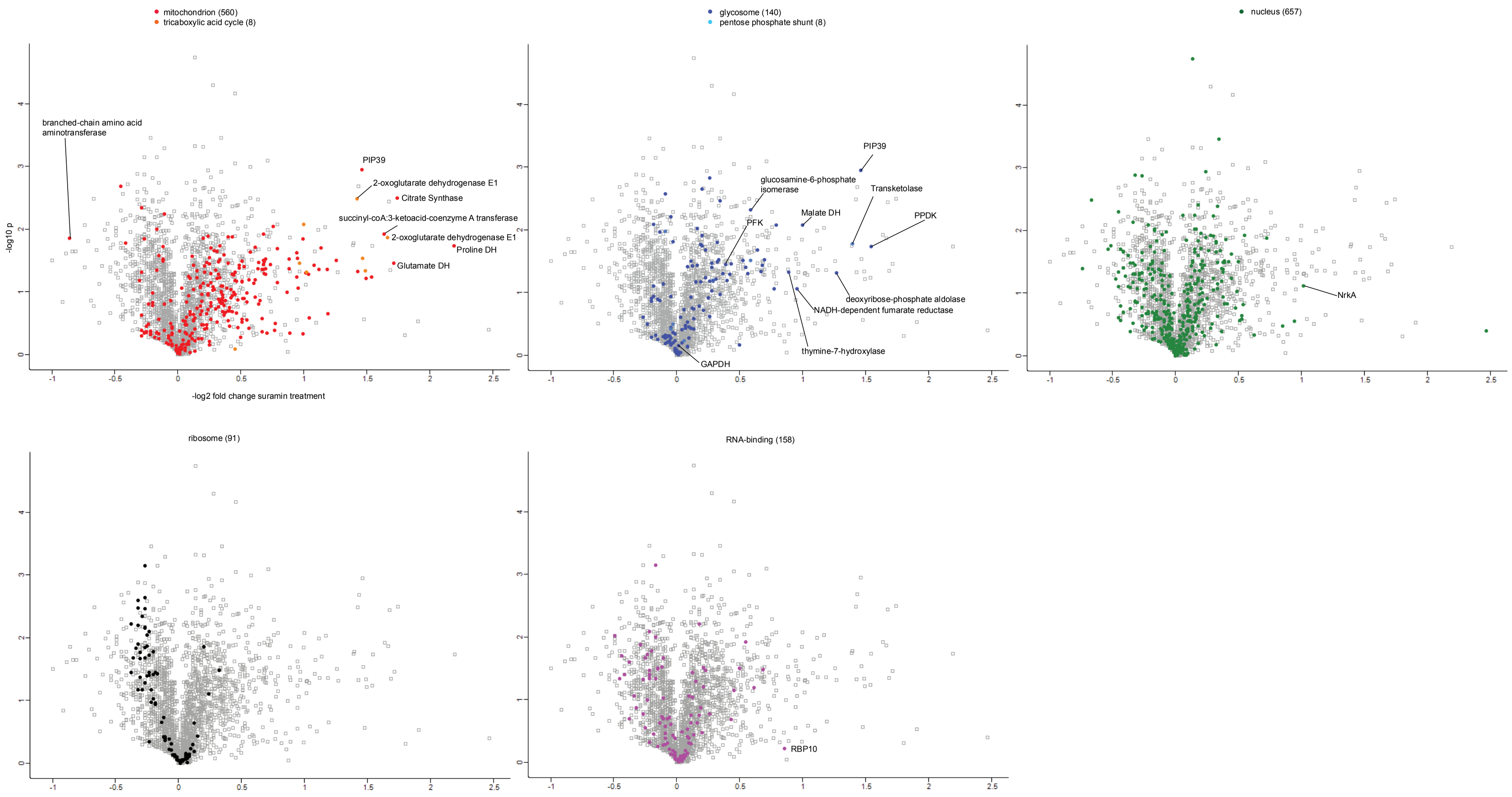
mitochondrion (560)
tricaboxylic acid cycle (8)
-log10 p
-log2 fold change suramin treatment
branched-chain amino acid aminotransferase
PIP39
2-oxoglutarate dehydrogenase E1
Citrate Synthase
succinyl-coA:3-ketoacid-coenzyme A transferase
2-oxoglutarate dehydrogenase E1
Proline DH
Glutamate DH
glycosome (140)
pentose phosphate shunt (8)
glucosamine-6-phosphate isomerase
PFK
Malate DH
Transketolase
PPDK
deoxyribose-phosphate aldolase
NADH-dependent fumarate reductase
thymine-7-hydroxylase
GAPDH
nucleus (657)
NrkA
ribosome (91)
RNA-binding (158)
RBP10

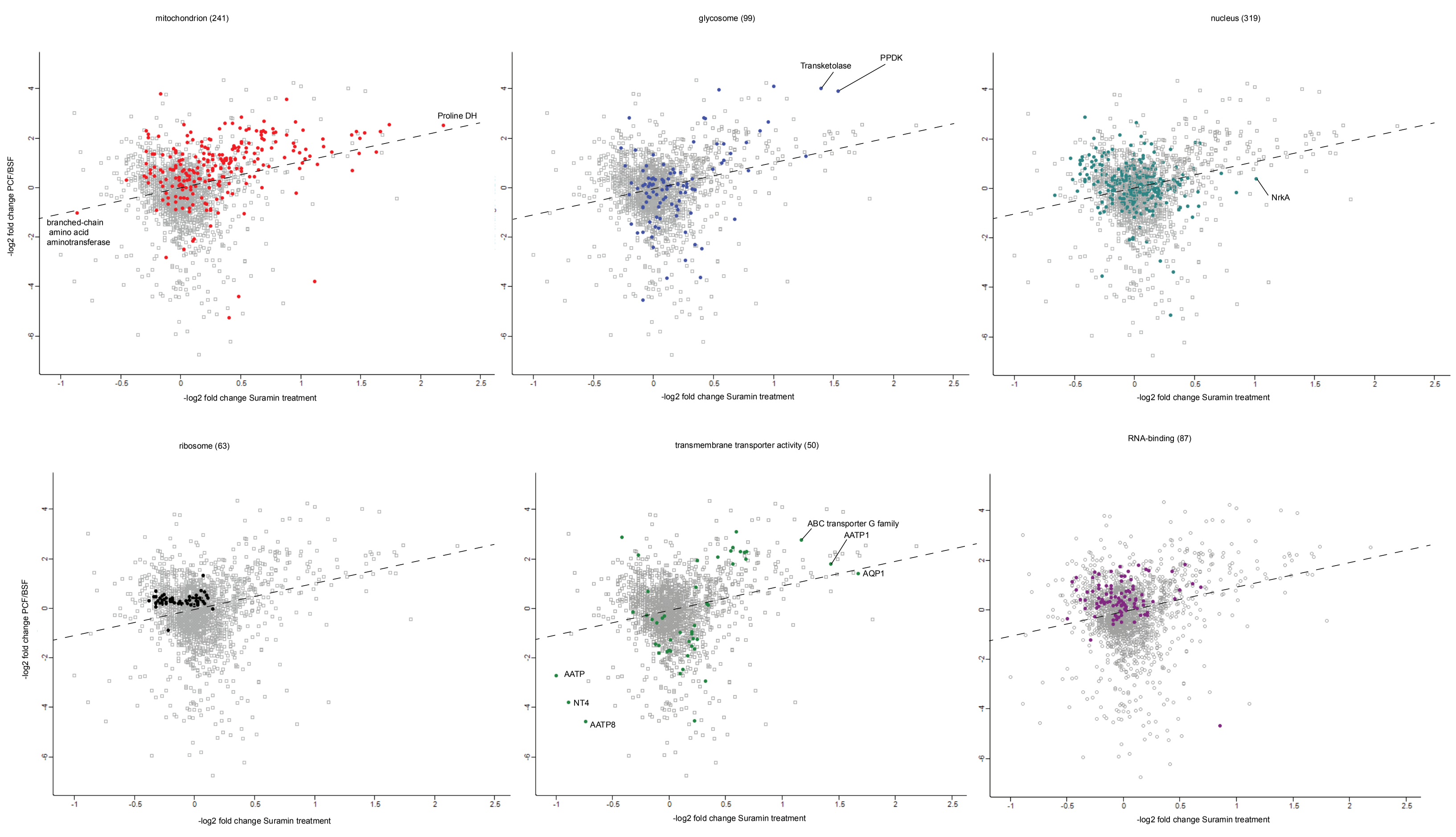
mitochondrion (241)
Proline DH
branched-chain amino acid aminotransferase
glycosome (99)
Transketolase
PPDK
nucleus (319)
NrkA
ribosome (63)
transmembrane transporter activity (50)
ABC transporter G family
AATP1
AQP1
AATP
NT4
AATP8
RNA-binding (87)
-log2 fold change PCF/BSF
-log2 fold change Suramin treatment

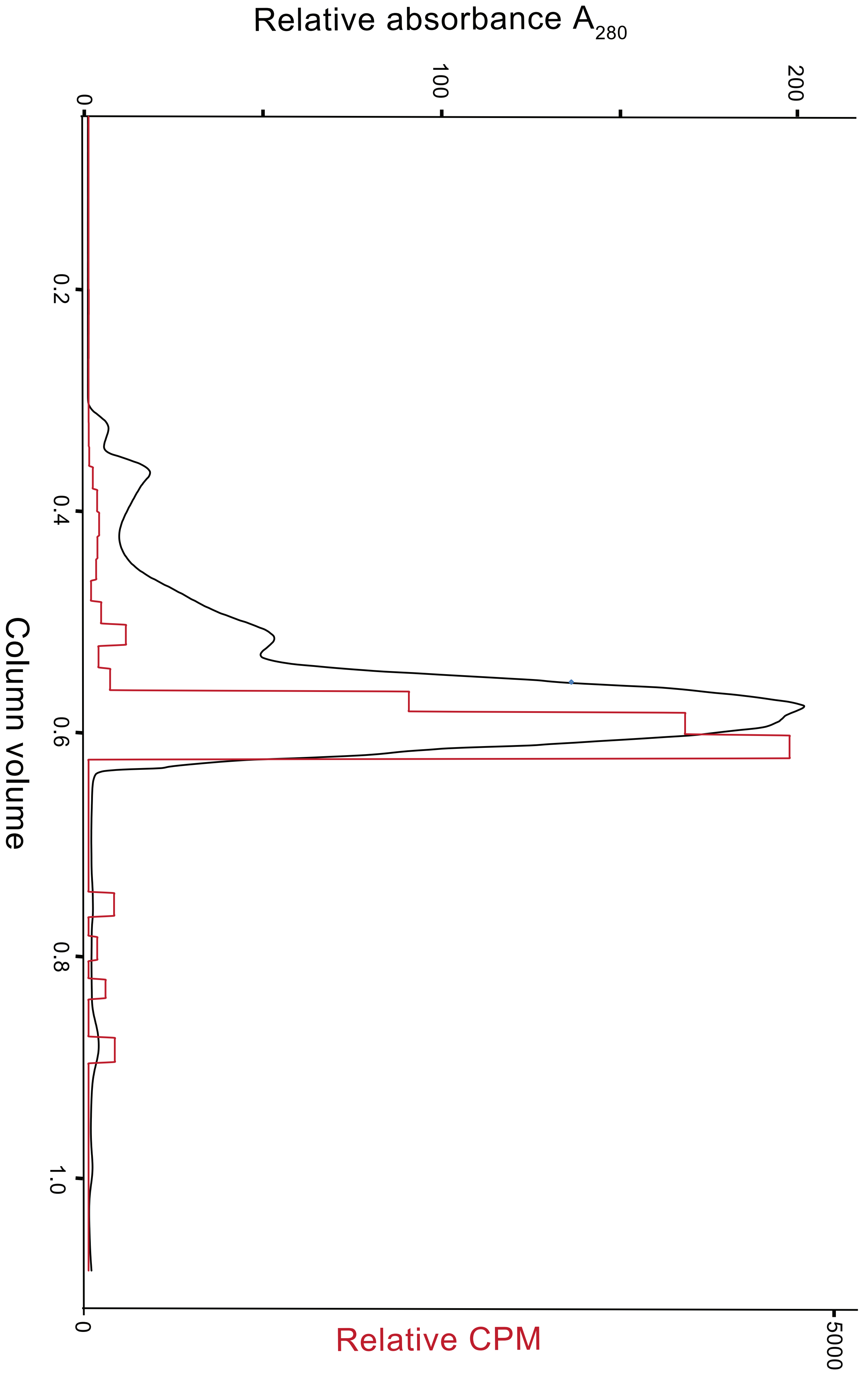

Relative absorbance A_{280}
0
100
200
0.2
0.4
0.6
0.8
1.0
Column volume
0
5000
Relative CPM

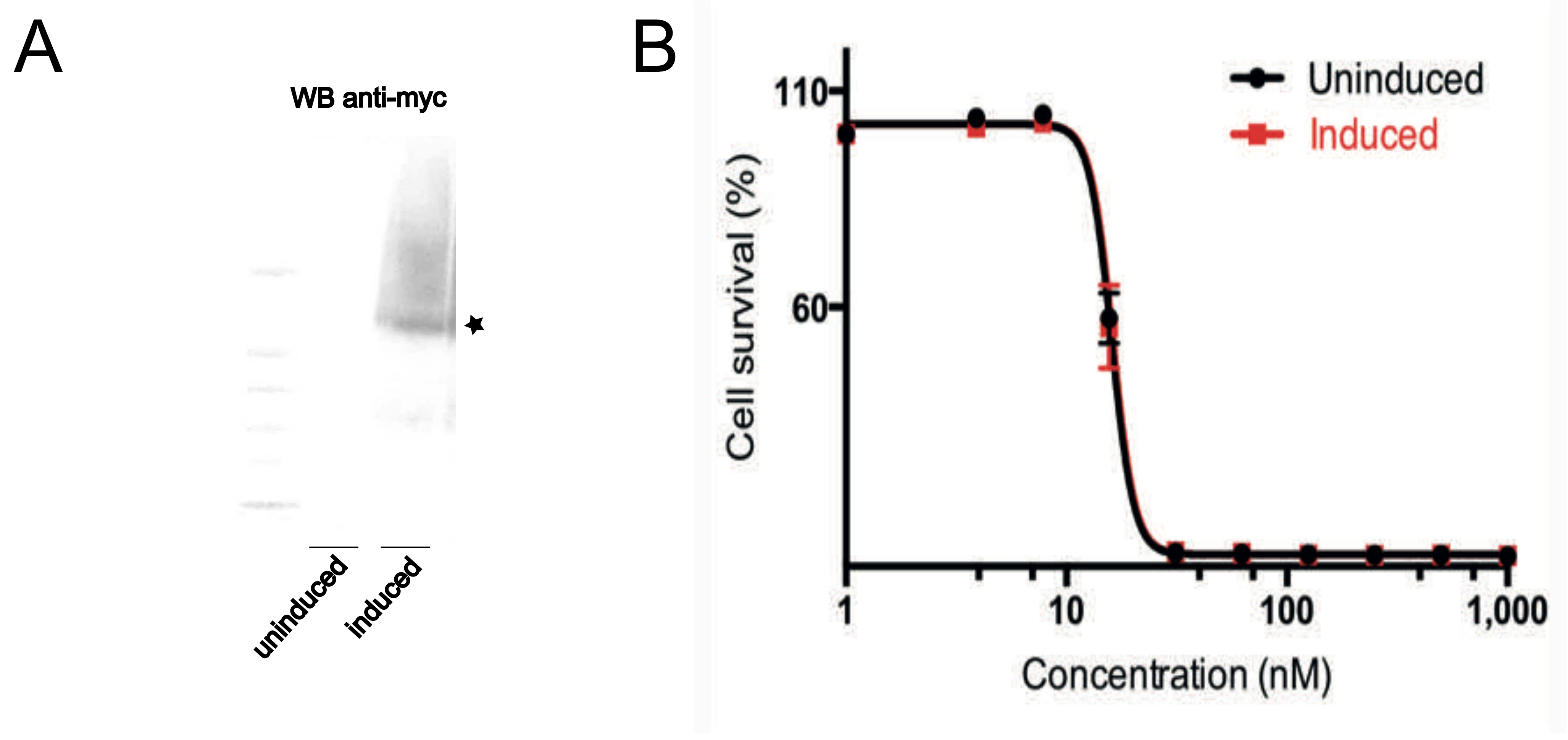
A
WB anti-myc
uninduced
induced
B
Uninduced
Induced
110
60
Cell survival (%)
1
10
100
1,000
Concentration (nM)
